# Mapping the human genetic architecture of COVID-19

**DOI:** 10.1038/s41586-021-03767-x

**Published:** 2021-07-08

**Authors:** COVID-19 Host Genetics Initiative, Mari E. K. Niemi, Juha Karjalainen, Rachel G. Liao, Benjamin M. Neale, Mark Daly, Andrea Ganna, Lea Davis, Sulggi Lee, James Priest, Alessandra Renieri, Vijay G. Sankaran, David van Heel, Patrick Deelen, J. Brent Richards, Tomoko Nakanishi, Les Biesecker, V. Eric Kerchberger, J. Kenneth Baillie, Francesca Mari, Anna Bernasconi, Stefano Ceri Baillie, Arif Canakoglu, Xiao Chang, Joseph R. Glessner, Hakon Hakonarson

**Affiliations:** 1 Institute for Molecular Medicine Finland (FIMM), University of Helsinki, Helsinki, Finland; 2 Broad Institute of MIT and Harvard, Cambridge, MA USA; 3 Analytic and Translational Genetics Unit, Massachusetts General Hospital, Boston, MA USA; 4 Massachusetts General Hospital, Broad Institute of MIT and Harvard, Cambridge, MA USA; 6 Yale University, New Haven, CT USA; 7 Icahn School of Medicine at Mount Sinai, New York, NY USA; 8 Stroke Pharmacogenomics and Genetics, Biomedical Research Institute Sant Pau (IIB Sant Pau), Sant Pau Hospital, Inmungen-CoV2, Barcelona, Spain; 9 Institute of Virology, Technical University Munich and Helmholtz Zentrum München, Munich, Germany; 10 Institute of Psychiatric Phenomics and Genomics, Medical Center of the University of Munich, Munich, Germany; 11 Department of Psychiatry, Medical Center of the University of Munich, Munich, Germany; 12 IRCCS, Istituto Giannina Gaslini, Genova, Italy; 13 Department of Neurosciences, Rehabilitation, Ophthalmology, Genetics, Maternal and Child Health, University of Genova, Genova, Italy; 14 Queen Mary University of London, London, UK; 15 Open Targets, Wellcome Genome Campus, Hinxton, UK; 16 Department of Complex Trait Genetics, Center for Neurogenomics and Cognitive Research, Amsterdam Neuroscience, Vrije Universiteit Amsterdam, Amsterdam, The Netherlands; 17 Lady Davis Institute, Jewish General Hospital, McGill University, Montreal, Quebec, Canada; 18 Medical Research Institute, Kangbuk Samsung Hospital, Sungkyunkwan University School of Medicine, Suwon, Republic of Korea; 19 Osaka University Graduate School of Medicine, Osaka, Japan; 20 Baylor College of Medicine, Houston, TX USA; 21 Mohammed Bin Rashid University of Medicine and Health Sciences, Dubai, United Arab Emirates; 22 MRC Integrative Epidemiology Unit (IEU), University of Bristol, Bristol, UK; 23 Department of Internal Medicine, Division of Cardiovascular Medicine, Michigan Medicine, Ann Arbor, MI USA; 24 Department of Human Genetics, University of Michigan Medical School, Ann Arbor, MI USA; 25 Department of Computational Medicine and Bioinformatics, University of Michigan Medical School, Ann Arbor, MI USA; 26 Seaver Autism Center for Research and Treatment, Department of Psychiatry, Icahn School of Medicine at Mount Sinai, New York, NY USA; 29 Program in Medical and Population Genetics, Broad Institute of MIT and Harvard, Cambridge, MA USA; 32 David Geffen School of Medicine at UCLA, Los Angeles, CA USA; 33 Institut Pasteur, Paris, France; 34 Harvard School of Public Health, Boston, MA USA; 35 Institute for Molecular Bioscience, The University of Queensland, Brisbane, Queensland, Australia; 38 Wellcome Sanger Institute, Wellcome Genome Campus, Hinxton, UK; 40 European Molecular Biology Laboratory, European Bioinformatics Institute (EMBL-EBI), Wellcome Genome Campus, Hinxton, UK; 42 Centre for Bioinformatics and Data Analysis, Medical University of Bialystok, Bialystok, Poland; 43 Trieste University, Trieste, Italy; 44 Vanderbilt University Medical Center, Nashville, TN USA; 45 University of California San Francisco, San Francisco, CA USA; 46 Stanford University, Stanford, CA USA; 47 University of Siena, Siena, Italy; 49 Boston Children’s Hospital, Broad Institute of MIT and Harvard, Cambridge, MA USA; 50 Blizard Institute, Queen Mary University of London, London, UK; 51 Department of Genetics, University Medical Centre Groningen, Groningen, The Netherlands; 52 Department of Genetics, University Medical Centre Utrecht, Utrecht, The Netherlands; 53 Department of Epidemiology, Biostatistics and Occupational Health, McGill University, Montreal, Quebec, Canada; 55 Department of Twin Research, King’s College London, London, UK; 56 Department of Human Genetics, McGill University, Montreal, Quebec Canada; 57 Kyoto-McGill International Collaborative School in Genomic Medicine, Graduate School of Medicine, Kyoto University, Kyoto, Japan; 59 National Institutes of Health, Bethesda, MD USA; 60 The Roslin Institute, University of Edinburgh, Edinburgh, UK; 61 Intensive Care Unit, Royal Infirmary of Edinburgh, Edinburgh, UK; 62 MRC Human Genetics Unit, Institute of Genetics and Molecular Medicine, University of Edinburgh, Western General Hospital, Edinburgh, UK; 63 Medical Genetics, University of Siena, Siena, Italy; 64 Genetica Medica, Azienda Ospedaliero-Universitaria Senese, Siena, Italy; 65 Med Biotech Hub and Competence Center, Department of Medical Biotechnologies, University of Siena, Siena, Italy; 66 Department of Electronics, Information and Bioengineering (DEIB), Politecnico di Milano, Milano, Italy; 67 Politecnico di Milano, Milan, Italy; 68 University of Michigan, Ann Arbor, MI USA; 69 Vanderbilt School of Medicine, Nashville, TN USA; 70 All India Institute of Medical Sciences Kalyani, Kalyani, India; 71 Hasso Plattner Institute, New York, NY USA; 72 Naina Tech, Hyderabad, India; 73 EMBL-European Bioinformatics Institute, Hinxton, UK; 74 University of Northampton, Northampton, UK; 75 University of Helsinki, Helsinki, Finland; 76 University of Miami, Miami, FL USA; 78 Ecole Centrale de Nantes, Inserm, Centre de Recherche en Transplantation et Immunologie, Nantes University, UMR1064, ITUN, Nantes, France; 79 University of Liège, Liège, Belgium; 80 Qatar Genome Program, Qatar Foundation Research, Development and Innovation, Qatar Foundation, Doha, Qatar; 82 Medical and Population Genetics and Cardiovascular Disease Initiative, Broad Institute of Harvard and MIT, Cambridge, Cambridge, MA USA; 83 Cardiovascular Research Center, Massachusetts General Hospital, Boston, MA USA; 84 Intensive Care Unit, Vall d’Hebron Hospital, Barcelona, Spain; 85 Institut de Biomedicina de València - CSIC, València, Spain; 86 Centro de Investigación Biomédica en Red en Enfermedades Neurodegenerativas (CIBERNED), València, Spain; 87 Unidad Mixta de Neurología y Genética, Instituto de Investigación Sanitaria La Fe, València, Spain; 88 Erasmus Medical Center, Rotterdam, The Netherlands; 89 National Genome Center, Copenhagen, Denmark; 90 University of Copenhagen, Copenhagen, Denmark; 91 Genomics PLC, Oxford, UK; 93 Institute for Community Medicine, University Medicine Greifswald, Greifswald, Germany; 94 Department of Population Medicine and Lifestyle Diseases Prevention, Medical University of Bialystok, Bialystok, Poland; 95 Genomics England, London, UK; 96 Junta de Andalucía, Seville, Spain; 97 Human Genetics Program of ICBM and Department of Basic-Clinical Oncology, University of Chile, Santiago, Chile; 98 Center for the Development of Scientific Research (CEDIC), Asunción, Paraguay; 100 Translational Bioinformatics Unit, Navarrabiomed, Complejo Hospitalario de Navarra (CHN), Universidad Pública de Navarra (UPNA), IdiSNA, Pamplona, Spain; 101 Mucosal & Salivary Biology Division, King’s College London Dental Institute, London, UK; 102 GENYO, Center for Genomics and Oncological Research Pfizer, University of Granada, Andalusian Regional Government, Granada, Spain; 103 University of Puerto Rico, San Juan, Puerto Rico; 104 National Laboratory of Genomics for Biodiversity (LANGEBIO), Advanced Genomics Unit, CINVESTAV, Irapuato, Mexico; 105 Queensland University of Technology, Brisbane, Queensland, Australia; 106 Clinical Research Unit of Nanoro, Institut de Recherche en Sciences de la Santé, CNRST, Ouagadougou, Burkina Faso; 107 McGill University, Montreal, Quebec, Canada; 108 Université de Montréal, Montreal, Quebec, Canada; 109 Fonds de la Recherche Scientifique (FNRS) & Centre de Génétique Humaine, Hôpital Erasme, Université Libre de Bruxelles, Brussels, Belgium; 112 University of Pecs Medical School, Pécs, Hungary; 113 Institute of Biomedicine and Cancer Research Laboratories, Western Cancer Centre FICAN West, University of Turku, Turku, Finland; 114 Institute of Biomedical Technologies, National Research Council, Segrate, Italy; 115 Immediate, Milan, Italy; 116 University of Cambridge, Cambridge, UK; 117 Genome Opinion, Seoul, Republic of Korea; 119 University of Groningen, Groningen, The Netherlands; 120 Universiti Malaysia Pahang, Gambang, Malaysia; 122 Vrije Universiteit Amsterdam, Amsterdam, The Netherlands; 123 University Medical Centre Groningen, University of Groningen, Groningen, The Netherlands; 124 MNM DIAGNOSTICS, Pozna?, Poland; 125 Institute for Systems Biology, Seattle, WA USA; 126 Sultan Idris Education University, Tanjung Malim, Malaysia; 127 Hospital Kulim, Kedah, Malaysia; 128 AbbVie, Lake Buff, IL USA; 129 Root Deep Insight, Boston MA, USA; 130 23andMe, Sunnyvale, CA USA; 131 GSK, Stevenage, UK; 132 Department of Pharmacology, Feinberg School of Medicine, Northwestern University, Chicago, IL USA; 133 Department of Medicine, Northwestern University, Chicago, IL USA; 134 Washington DC Veterans Affairs Medical Center, Hospital Medicine, Washington, DC USA; 135 Department of Medicine, George Washington University, Washington, DC USA; 136 Section of Hospital Medicine, Department of Medicine, University of Chicago, Chicago, IL USA; 137 Section of Hematology and Oncology, Department of Medicine, University of Chicago, Chicago, IL USA; 138 College of Pharmacy, University of Illinois at Chicago, Chicago, IL USA; 144 Department of Pharmacology, George Washington University, Washington, DC USA; 148 Department of Neurology, Amsterdam UMC, Amsterdam Neuroscience, Amsterdam, The Netherlands; 149 Department of Intensive Care, Amsterdam UMC, Amsterdam, The Netherlands; 150 Department of Infectious Diseases, Amsterdam UMC, Amsterdam, The Netherlands; 151 Department of Clinical Epidemiology, Biostatistics and Bioinformatics, Amsterdam UMC, Amsterdam, The Netherlands; 152 Experimental Immunology, Amsterdam UMC, Amsterdam, The Netherlands; 153 Department of Pulmonology, Amsterdam UMC, Amsterdam, The Netherlands; 154 Department of Pathology, Amsterdam UMC, Amsterdam, The Netherlands; 155 Department of Anesthesiology, Amsterdam UMC, Amsterdam, The Netherlands; 156 Amsterdam UMC Biobank Core Facility, Amsterdam UMC, Amsterdam, The Netherlands; 157 Department of Radiology, Amsterdam UMC, Amsterdam, The Netherlands; 158 Department of Medical Microbiology, Amsterdam UMC, Amsterdam, The Netherlands; 159 Department of Clinical Chemistry, Amsterdam UMC, Amsterdam, The Netherlands; 160 Amsterdam UMC Biobank, Amsterdam UMC, Amsterdam, The Netherlands; 161 Core Facility Genomics, Amsterdam UMC, Amsterdam, The Netherlands; 162 Ancestry, Lehi, UT USA; 163 GIGA-Institute, University of Liège, Liège, Belgium; 164 CHC Mont-Légia, Liège, Belgium; 165 BHUL (Liège Biobank), CHU of Liège, Liège, Belgium; 167 Stanley Center for Psychiatric Research, Broad Institute of MIT and Harvard, Cambridge, MA USA; 168 Centre de Génétique Humaine, Hôpital Erasme, Université Libre de Bruxelles, Brussels, Belgium; 169 Service de Médecine Interne, Hôpital Erasme, Université Libre de Bruxelles, Brussels, Belgium; 170 CHU of Liège, University of Liège, Liège, Belgium; 174 McGill Genome Centre, McGill University, Montréal, Québec, Canada; 177 Department of Emergency Medicine, McGill University, Montreal, Quebec Canada; 178 Emergency Department, Jewish General Hospital, McGill University, Montreal, Quebec, Canada; 179 McGill AIDS Centre, Department of Microbiology and Immunology, Lady Davis Institute for Medical Research, Jewish General Hospital, McGill University, Montreal, Quebec, Canada; 180 McGill Centre for Viral Diseases, Department of Infectious Disease, Lady Davis Institute, Jewish General Hospital, Montreal, Quebec, Canada; 181 Research Centre of the Centre Hospitalier de l’Université de Montréal, Montreal, Quebec, Canada; 182 Department of Medicine, Research Centre of the Centre Hospitalier de l’Université de Montréal, Montreal, Quebec Canada; 183 Department of Medicine, Université de Montréal, Montreal, Quebec Canada; 184 Department of Medicine and Human Genetics, McGill University, Montreal, Quebec Canada; 185 Department of Intensive Care, Research Centre of the Centre Hospitalier de l’Université de Montréal, Montreal, Quebec Canada; 186 Division of Infectious Diseases, Research Centre of the Centre Hospitalier de l’Université de Montréal, Montréal, Quebec, Canada; 187 Division of Genetic Medicine, Department of Medicine, Vanderbilt University Medical Center, Nashville, TN USA; 188 Vanderbilt Genetics Institute, Vanderbilt University Medical Center, Nashville, TN USA; 189 Institute of Human Genetics, University Hospital Bonn, Medical Faculty University of Bonn, Bonn, Germany; 190 Institute of Genomic Statistics and Bioinformatics, University Hospital Bonn, Medical Faculty University of Bonn, Bonn, Germany; 191 Department of Gastroenterology, Hepatology and Infectious Diseases, University Hospital Düsseldorf, Medical Faculty Heinrich Heine University, Düsseldorf, Germany; 192 Institute of Human Genetics, Medical Faculty, RWTH Aachen University, Aachen, Germany; 193 Clinic for Cardiology, Angiology and Internal Intensive Medicine, Medical Clinic I, RWTH Aachen University, Aachen, Germany; 194 Department of Pneumology and Intensive Care Medicine, Faculty of Medicine, RWTH Aachen University, Aachen, Germany; 195 Department of Pneumology, Hannover Medical School, Hannover, Germany; 196 Department of Gastroenterology, Hepatology and Endocrinology, Hannover Medical School, Hannover, Germany; 197 Hannover Unified Biobank, Hannover Medical School, Hannover, Germany; 198 Department I of Internal Medicine, Faculty of Medicine and University Hospital of Cologne, University of Cologne, Cologne, Germany; 199 Center for Molecular Medicine Cologne (CMMC), University of Cologne, Cologne, Germany; 200 German Center for Infection Research (DZIF), Partner Site Bonn-Cologne, Cologne, Germany; 202 Cologne Center for Genomics (CCG), University of Cologne, Cologne, Germany; 203 Department of Anesthesiology and Intensive Care Medicine, University Hospital Essen, University Duisburg-Essen, Essen, Germany; 204 Department of Child and Adolescent Psychiatry, University Hospital Essen, University of Duisburg-Essen, Essen, Germany; 205 Department of Infectious Diseases, University Hospital Essen, University Duisburg-Essen, Essen, Germany; 206 Department of Pneumology, Allergology and Respiratory Medicine, University Hospital Saarland, Homburg/Saar, Germany; 207 Center of Human and Molecular Biology, Department of Human Genetics, University Hospital Saarland, Homburg/Saar, Germany; 208 Department of Genetics & Epigenetics, Saarland University, Saarbrücken, Germany; 209 Eurac Research, Institute for Biomedicine (affiliated to the University of Lübeck), Bolzano, Italy; 210 University of Colorado Anschutz Medical Campus, Aurora, CO USA; 211 Department of Genetics and Development, Institute for Genomic Medicine, Columbia University, New York, NY USA; 212 Department of Medicine, Institute for Genomic Medicine, Columbia University, New York, NY USA; 213 Department of Biomedical Informatics, Columbia University, New York, NY USA; 214 Department of Pediatrics, Columbia University, New York, NY USA; 215 Department of Medicine, Columbia University, New York, NY USA; 216 Institute for Genomic Medicine, Columbia University, New York, NY USA; 217 Department of Biostatistics, Mailman School of Public Health, Columbia University, New York, NY USA; 218 Department of Pathology and Cell Biology, Columbia University, New York, NY USA; 219 Medical Research Institute, Kangbuk Samsung Hospital, Sungkyunkwan University School of Medicine, Seoul, Republic of Korea; 220 Department of Clinical Research Design and Evaluation, SAIHST, Sungkyunkwan University, Seoul, Republic of Korea; 221 Division of Gastroenterology, Department of Medicine, Kangbuk Samsung Hospital, Sungkyunkwan University, School of Medicine, Seoul, Republic of Korea; 222 Department of Biochemistry, College of Medicine, Ewha Womans University, Seoul, Republic of Korea; 223 Department of Internal Medicine, Seoul National University Hospital, Seoul National University College of Medicine, Seoul, Republic of Korea; 224 Department of Periodontology, Section of Dentistry, Seoul National University Bundang Hospital, Seongnam, Republic of Korea; 225 Department of Internal Medicine, Seoul National University Bundang Hospital, Seoul National University College of Medicine, Seongnam, Republic of Korea; 226 Department of Physical & Rehabilitation Medicine, Kangbuk Samsung Hospital, Sungkyunkwan University School of Medicine, Seoul, Republic of Korea; 227 Department of Clinical Research Design & Evaluation, SAIHST, Sungkyunkwan University, Seoul, Republic of Korea; 228 Biomedical Institute for Convergence at SKKU, Sungkyunkwan University School of Medicine, Suwon, Republic of Korea; 229 Department of Public Health Service, Seoul National University Bundang Hospital, Seongnam, Republic of Korea; 230 Department of Rehabilitation Medicine, Seoul National University College of Medicine, Seoul, Republic of Korea; 231 Korea Research Environment Open NETwork, Korea Institute of Science and Technology Information, Daejeon, Republic of Korea; 232 Global Science Experimental Data Hub Center, Korea Institute of Science and Technology Information, Daejeon, Republic of Korea; 233 Division of Infectious Diseases, Department of Medicine, Kangbuk Samsung Hospital, Sungkyunkwan University School of Medicine, Seoul, Republic of Korea; 234 Center for Cohort Studies, Kangbuk Samsung Hospital, Sungkyunkwan University School of Medicine, Seoul, Republic of Korea; 235 Department of Occupational and Environmental Medicine, Sungkyunkwan University School of Medicine, Seoul, Republic of Korea; 236 Department of Laboratory Medicine, Seoul National University Bundang Hospital, Seoul National University College of Medicine, Seongnam, Republic of Korea; 237 Institute of Clinical Molecular Biology, Christian-Albrechts-University, Kiel, Germany; 238 Novo Nordisk Foundation Center for Protein Research, Disease Systems Biology, Faculty of Health and Medical Sciences, University of Copenhagen, Copenhagen, Denmark; 239 Institut de Biotecnologia i de Biomedicina, Universitat Autònoma de Barcelona, Barcelona, Spain; 240 ICREA, Barcelona, Spain; 241 Research Group for Evolutionary Immunogenomics, Max Planck Institute for Evolutionary Biology, Plön, Germany; 242 Research Unit for Evolutionary Immunogenomics, Department of Biology, University of Hamburg, Hamburg, Germany; 243 Department of Gastroenterology, Hospital Universitario Ramón y Cajal, University of Alcalá, Instituto Ramón y Cajal de Investigación Sanitaria (IRYCIS), Madrid, Spain; 244 Centro de Investigación Biomédica en Red en Enfermedades Hepáticas y Digestivas (CIBEREHD), Instituto de Salud Carlos III (ISCIII), Madrid, Spain; 245 Vall d’Hebron Institut de Recerca (VHIR), Vall d’Hebron Hospital Universitari, Barcelona, Spain; 246 Charite Universitätsmedizin Berlin, Berlin, Germany; 249 Hospital Universitario Clinico San Cecilio, Granada, Spain; 250 Instituto de Investigación Ibs.Granada, Granada, Spain; 251 Emergency Department, University Hospital Regensburg, Regensburg, Germany; 252 Department for Infectious Diseases and Infection Control, University Hospital Regensburg, Regensburg, Germany; 253 Medical University of Innsbruck, Department of Medicine and Christian Doppler Laboratory on Iron and Phosphate Biology, Innsbruck, Austria; 254 Institute of Clinical Medicine, University of Oslo, Oslo, Norway; 255 Department of Microbiology, Oslo University Hospital, Oslo, Norway; 256 Hospital Clinic, University of Barcelona and IDIBAPS, Barcelona, Spain; 257 European Foundation for the Study of Chronic Liver Failure (EF-CLIF), Barcelona, Spain; 259 Cologne Excellence Cluster on Cellular Stress Responses in Aging-Associated Diseases (CECAD), University of Cologne, Cologne, Germany; 260 Center for Molecular Medicine Cologne (CMMC), University of Cologne, Cologne, Germany; 261 Genomes for Life-GCAT labGermans Trias i Pujol Research Institute (IGTP), Badalona, Spain; 262 IRCCS Humanitas Research Hospital, Milan, Italy; 263 Department of Biomedical Sciences, Humanitas University, Pieve Emanuele, Milan, Italy; 264 Institute of Transfusionsmedicine, University Hospital Schleswig-Holstein (UKSH), Kiel, Germany; 265 Klinik für Innere Medizin I, Universitätsklinikum Schleswig-Holstein, Kiel Campus, Kiel, Germany; 266 Zentrum für Humangenetik Regensburg, Regensburg, Germany; 267 University Hospital Schleswig-Holstein (UKSH), Kiel Campus, Kiel, Germany; 268 Section for Gastroenterology, Department of Transplantation Medicine, Division for Cancer Medicine, Surgery and Transplantation, Oslo University Hospital Rikshospitalet, Oslo, Norway; 269 Research Institute for Internal Medicine, Division of Surgery, Inflammatory Diseases and Transplantation, Oslo University Hospital Rikshospitalet and University of Oslo, Oslo, Norway; 270 Norwegian PSC Research Center, Department of Transplantation Medicine, Division of Surgery, Inflammatory Diseases and Transplantation, Oslo University Hospital Rikshospitalet, Oslo, Norway; 272 Division of Rheumatology, Inflammation and Immunity, Brigham and Women’s Hospital and Harvard Medical School, Boston, MA USA; 273 Division of Genetics, Department of Medicine, Brigham and Women’s Hospital, Boston, MA USA; 274 Department of Biomedical Informatics, Harvard Medical School, Boston, MA USA; 275 Center for Data Sciences, Brigham and Women’s Hospital, Boston, MA USA; 276 Randaberg Municipality, Randaberg, Norway; 277 Department of Quality and Health Technology, Faculty of Health Sciences, University of Stavanger, Stavanger, Norway; 278 Department of Genetics and Bioinformatics (HDGB), Division of Health Data and Digitalization, Norwegian Institute of Public Health, Oslo, Norway; 279 Centre for Genetics and Genomics Versus Arthritis, Centre for Musculoskeletal Research, Manchester Academic Health Science Centre, The University of Manchester, Manchester, UK; 280 Department of Intensive Care, Hospital Universitario Ramón y Cajal, Instituto Ramón y Cajal de Investigación Sanitaria (IRYCIS), University of Alcalá, Madrid, Spain; 281 Osakidetza Basque Health Service, Donostialdea Integrated Health Organisation, Clinical Biochemistry Department, San Sebastian, Spain; 282 Research Center and Memory Clinic, Fundació ACE, Institut Català de Neurociències Aplicades, Universitat Internacional de Catalunya, Barcelona, Spain; 283 Networking Research Center on Neurodegenerative Diseases (CIBERNED), Instituto de Salud Carlos III, Madrid, Spain; 284 Department of Acute Medicine, Oslo University Hospital, Oslo, Norway; 285 Fondazione IRCCS Ca’ Granda Ospedale Maggiore Policlinico, Milan, Italy; 286 European Reference Network on Hepatological Diseases (ERN RARE LIVER), San Gerardo Hospital, Monza, Italy; 287 Division of Gastroenterology, Center for Autoimmune Liver Diseases, Department of Medicine and Surgery, University of Milan Bicocca, Milan, Italy; 288 German Center for Neurodegenerative Diseases (DZNE Bonn), Bonn, Germany; 289 Division of Neurogenetics and Molecular Psychiatry, Department of Psychiatry and Psychotherapy, Medical Faculty, University of Cologne, Cologne, Germany; 290 Department of Psychiatry, Glenn Biggs Institute for Alzheimer’s and Neurodegenerative Diseases, San Antonio, TX USA; 291 Department of Neurodegenerative Diseases and Geriatric Psychiatry, University Hospital Bonn, Bonn, Germany; 292 Liver Unit, Department of Internal Medicine, Hospital Universitari Vall d’Hebron, Vall d’Hebron Barcelona Hospital Campus, Barcelona, Spain; 293 Department of Anesthesiology and Intensive Care, University Hospital of North Norway, Tromsø, Norway; 294 Klinik für Innere Medizin I, Universitätsklinikum Schleswig-Holstein, Kiel Campus, Kiel, Germany; 295 Gastroenterology Unit, Fondazione IRCCS Casa Sollievo della Sofferenza, San Giovanni Rotondo, Italy; 296 Department of Infectious Diseases, Oslo University Hospital, Oslo, Norway; 297 Microbiology Department, Hospital Universitari Vall d’Hebron, Barcelona, Spain; 298 Universitat Autònoma de Barcelona, Bellaterra, Spain; 299 Department of Respiratory Diseases, Hospital Universitario Ramón y Cajal, Instituto Ramón y Cajal de Investigación Sanitaria (IRYCIS), Madrid, Spain; 300 Department of Respiratory Medicine and Allergology, University Hospital, Goethe University, Frankfurt am Main, Germany; 301 Department of Infectious Diseases, Hospital Universitario Ramón y Cajal, Instituto Ramón y Cajal de Investigación Sanitaria (IRYCIS), University of Alcalá, Madrid, Spain; 302 Department of Internal Medicine II, Technical University of Munich, School of Medicine, University Hospital rechts der Isar, Munich, Germany; 303 Division of Clinical Infectious Diseases, Research Center Borstel, Borstel, Germany; 304 German Center for Infection Research (DZIF) Clinical Tuberculosis Unit, Borstel, Germany; 305 Respiratory Medicine & International Health, University of Lübeck, Lübeck, Germany; 306 Osakidetza Basque Health Service, Basurto University Hospital, Respiratory Service, Bilbao, Spain; 307 Department of Clinical and Molecular Medicine, Faculty of Medicine and Health Science, Norwegian University of Science and Technology, Trondheim, Norway; 308 Clinic Ålesund Hospital, Department of Medicine, Møre & Romsdal Hospital Trust, Ålesund, Norway; 309 Department of Anesthesiology, Hospital Universitario Ramón y Cajal, Instituto Ramón y Cajal de Investigación Sanitaria (IRYCIS), University of Alcalá, Madrid, Spain; 310 Spain Hospital Clinic, University of Barcelona and IDIBAPS, Barcelona, Spain; 311 Osakidetza Basque Health Service, Galdakao Hospital, Respiratory Service, Galdakao, Spain; 312 IBMDR - E.OOspedali Galliera, Genova, Italy; 313 Liver ICU, Hospital Clinic Barcelona, Barcelona, Spain; 314 Biocruces Bizkaia Health Research Institute, Barakaldo, Spain; 315 Histocompatibilidad y Biologia Molecular, Centro de Transfusion de Madrid, Madrid, Spain; 316 University of Milan, Milan, Italy; 317 Fondazione Grigioni per il Morbo di Parkinson, Milan, Italy; 318 Department of Anesthesiology, Intensive Care Medicine and Pain Therapy, University Hospital Frankfurt, Frankfurt am Main, Germany; 319 German Center for Infection Research (DZIF), Medical Faculty and University Hospital Cologne, University of Cologne, Partner Site Bonn-Cologne, Cologne, Germany; 321 Ikerbasque, Basque Foundation for Science, Bilbao, Spain; 322 Department of Liver and Gastrointestinal Diseases, Biodonostia Health Research Institute, Donostia University Hospital, University of the Basque Country (UPV/EHU), San Sebastian, Spain; 323 Infectious Diseases Service, Osakidetza, Biocruces Bizkaia Health Research Institute, Barakaldo, Spain; 324 Medical Department, Drammen Hospital, Vestre Viken Hospital Trust, Drammen, Norway; 325 Research Center Borstel, BioMaterialBank Nord, Borstel, Germany; 326 German Center for Lung Research (DZL), Airway Research Center North (ARCN), Giessen, Germany; 327 Popgen 2.0 Network (P2N), Kiel, Germany; 329 Department of Liver and Gastrointestinal Diseases, Biodonostia Health Research Institute, Donostia University Hospital, University of the Basque Country (UPV/EHU), CIBERehd, San Sebastian, Spain; 330 Department of Infectious Diseases, Oslo University Hospital, Oslo, Norway; 331 Department of Clinical Science, University of Bergen, Bergen, Norway; 332 Biodonostia Health Research Institute, Donostia University Hospital, San Sebastian, Spain; 333 Germans Trias i Pujol Research Institute (IGTP), Badalona, Spain; 335 ISGlobal, Barcelona, Spain; 336 CIBER Epidemiología y Salud Pública (CIBERESP), Madrid, Spain; 337 Universitat Pompeu Fabra (UPF), Barcelona, Spain; 338 Hospital del Mar Medical Research Institute (IMIM), Barcelona, Spain; 339 Osakidetza Basque Health Service, Donostialdea Integrated Health Organization, San Sebastian, Spain; 340 Department of Internal Medicine, Infectious Diseases, University H.ospital Frankfurt and Goethe University Frankfurt, Frankfurt am Main, Germany; 341 Humanitas Gavazzeni-Castelli, Bergamo, Italy; 344 School of Biological Sciences, Monash University, Clayton, Victoria, Australia; 345 Munich Clinic Schwabing, Academic Teaching Hospital, Ludwig-Maximilians-University (LMU), Munich, Germany; 346 Department of Anesthesiology, Hospital Universitario Ramón y Cajal, Instituto Ramón y Cajal de Investigación Sanitaria (IRYCIS), Madrid, Spain; 349 Clinical Trials Centre Cologne, ZKS Köln, Cologne, Germany; 351 Institute of Human Genetics, University of Bonn School of Medicine, University Hospital Bonn, Bonn, Germany; 352 Institute of Clinical Molecular Biology, Christian-Albrechts-University of Kiel, Kiel, Germany; 353 UKSH Schleswig-Holstein, Kiel, Germany; 354 Institute of Immunology, Christian-Albrechts-University of Kiel, Kiel, Germany; 355 Institute of Medical Virology, University Hospital Frankfurt, Goethe University, Frankfurt am Main, Germany; 356 German Centre for Infection Research (DZIF), External Partner Site Frankfurt, Frankfurt am Main, Germany; 357 Department of Neurology, Bezirksklinikum Regensburg, University of Regensburg, Regensburg, Germany; 359 Charite Universitätsmedizin Berlin, Berlin Institute of Health, Berlin, Germany; 360 German Center for Infection Research (DZIF), Partner Site Munich, Munich, Germany; 361 Department of Infectious Diseases, University Hospital of North Norway, Tromsø, Norway; 362 Faculty of Health Sciences, UIT The Arctic University of Norway, Tromsø, Norway; 363 Catalan Institute of Oncology (ICO), Barcelona, Spain; 364 Bellvitge Biomedical Research Institute (IDIBELL), Barcelona, Spain; 365 Universitat de Barcelona (UB), Barcelona, Spain; 366 deCODE genetics, Reykjavik, Iceland; 368 Mucosal Immunology Lab, Unidad de Excelencia Instituto de Biomedicina y Genética Molecular de Valladolid (IBGM), Universidad de Valladolid-CSIC, Valladolid, Spain; 369 Centro de Investigaciones Biomédicas en Red de Enfermedades Hepáticas y Digestivas (CIBERehd), Madrid, Spain; 370 Valladolid University Hospital, Valladolid, Spain; 371 Estonian Genome Centre, Institute of Genomics, University of Tartu, Tartu, Estonia; 372 SYNLAB Estonia, University of Tartu, Tartu, Estonia; 373 University of Tartu, Tartu, Estonia; 374 Kuressaare Hospital, Kuressaare, Estonia; 375 Institute of Biomedicine and Translational Medicine, University of Tartu, Tartu, Estonia; 377 West Tallinn Central Hospital, Tallinn, Estonia; 378 University of Tartu, Tartu University Hospital, Tartu, Estonia; 379 Estonian Health Insurance Fund, Tallinn, Estonia; 380 Tartu University Hospital, Tartu, Estonia; 381 FinnGen, Helsinki, Finland; 382 Finnish Institute for Health and Welfare (THL), Helsinki, Finland; 383 University of Helsinki, Faculty of Medicine, Clinical and Molecular Metabolism Research Program, Helsinki, Finland; 384 Institute of Molecular and Clinical Ophthalmology Basel (IOB), Basel, Switzerland; 385 Department of Ophthalmology, University of Basel, Basel, Switzerland; 386 Infectious Diseases Service, Department of Medicine, University Hospital and University of Lausanne, Lausanne, Switzerland; 388 Centre for Primary Care and Public Health, University of Lausanne, Lausanne, Switzerland; 389 Division of Infectious Diseases and Hospital Epidemiology, Cantonal Hospital St Gallen, St Gallen, Switzerland; 390 Division of Intensive Care, Geneva University Hospitals and the University of Geneva Faculty of Medicine, Geneva, Switzerland; 391 Infectious Disease Service, Department of Internal Medicine, Geneva University Hospital, Geneva, Switzerland; 392 Clinique de Médecine et spécialités, Infectiologie, HFR-Fribourg, Fribourg, Switzerland; 393 Infectious Diseases Division, University Hospital Centre of the Canton of Vaud, Hospital of Valais, Sion, Switzerland; 394 Functional Host Genomics of Infectious Diseases, University Hospital and University of Lausanne, Lausanne, Switzerland; 395 Registry COVID, University Hospital and University of Lausanne, Lausanne, Switzerland; 396 Pneumonia Prediction using Lung Ultrasound, University Hospital and University of Lausanne, Lausanne, Switzerland; 397 Center for Primary Care and Public Health (Unisanté), University of Lausanne, Lausanne, Switzerland; 398 COVID-19 Risk Prediction in Swiss ICUs-Trial, Division of Infectious Diseases and Hospital Epidemiology, Cantonal Hospital St Gallen, St Gallen, Switzerland; 399 GCAT-Genomes for Life, Germans Trias i Pujol Health Sciences Research Institute (IGTP), Badalona, Spain; 400 Catalan Institute of Oncology, Bellvitge Biomedical Research Institute, Consortium for Biomedical Research in Epidemiology and Public Health, University of Barcelona, Barcelona, Spain; 401 Barcelona Supercomputing Center, Centro Nacional de Supercomputación (BSC-CNS), Life & Medical Sciences, Barcelona, Spain; 404 University of Siena, DIISM-SAILAB, Siena, Italy; 405 Université Côte d’Azur, Inria, CNRS, I3S, Maasai, Nice, France; 408 Division of Infectious Diseases and Immunology, Department of Medical Sciences and Infectious Diseases, Fondazione IRCCS Policlinico San Matteo, Pavia, Italy; 409 Department of Internal Medicine and Therapeutics, University of Pavia, Pavia, Italy; 410 Department of Infectious and Tropical Diseases, University of Brescia and ASST Spedali Civili Hospital, Brescia, Italy; 411 Chirurgia Vascolare, Ospedale Maggiore di Crema, Crema, Italy; 412 III Infectious Diseases Unit, ASST-FBF-Sacco, Milan, Italy; 413 Department of Biomedical and Clinical Sciences Luigi Sacco, University of Milan, Milan, Italy; 414 Department of Specialized and Internal Medicine, Tropical and Infectious Diseases Unit, Azienda Ospedaliera Universitaria Senese, Siena, Italy; 415 Unit of Respiratory Diseases and Lung Transplantation, Department of Internal and Specialist Medicine, University of Siena, Siena, Italy; 416 Department of Emergency and Urgency, Medicine, Surgery and Neurosciences, Unit of Intensive Care Medicine, Siena University Hospital, Siena, Italy; 417 Department of Medical, Surgical and Neurosciences and Radiological Sciences, Unit of Diagnostic Imaging, University of Siena, Siena, Italy; 418 Rheumatology Unit, Department of Medicine, Surgery and Neurosciences, University of Siena, Policlinico Le Scotte, Siena, Italy; 419 Department of Specialized and Internal Medicine, Infectious Diseases Unit, San Donato Hospital Arezzo, Arezzo, Italy; 420 Department of Emergency, Anesthesia Unit, San Donato Hospital, Arezzo, Italy; 421 Department of Specialized and Internal Medicine, Pneumology Unit and UTIP, San Donato Hospital, Arezzo, Italy; 422 Department of Emergency, Anesthesia Unit, Misericordia Hospital, Grosseto, Italy; 423 Department of Specialized and Internal Medicine, Infectious Diseases Unit, Misericordia Hospital, Grosseto, Italy; 424 Department of Preventive Medicine, Azienda USL Toscana Sud Est, Arezzo, Italy; 425 Clinical Chemical Analysis Laboratory, Misericordia Hospital, Grosseto, Italy; 426 Territorial Scientific Technician Department, Azienda USL Toscana Sud Est, Arezzo, Italy; 427 Clinical Chemical Analysis Laboratory, San Donato Hospital, Arezzo, Italy; 428 Department of Health Sciences, Clinic of Infectious Diseases, ASST Santi Paolo e Carlo, University of Milan, Milan, Italy; 429 Department of Anesthesia and Intensive Care, University of Modena and Reggio Emilia, Modena, Italy; 430 HIV/AIDS Department, National Institute for Infectious Diseases, IRCCS, Lazzaro Spallanzani, Rome, Italy; 431 Infectious Diseases Clinic, Department of Medicine, Azienda Ospedaliera di Perugia, Perugia, Italy; 432 Infectious Diseases Clinic, Santa Maria Hospital, University of Perugia, Perugia, Italy; 433 Department of Infectious Diseases, Treviso Hospital, Treviso, Italy; 434 Clinical Infectious Diseases, Mestre Hospital, Venezia, Italy; 435 Infectious Diseases Clinic, ULSS1, Belluno, Italy; 436 Medical Genetics and Laboratory of Medical Genetics Unit, A.O.R.N “Antonio Cardarelli”, Naples, Italy; 437 Department of Molecular Medicine and Medical Biotechnology, University of Naples Federico II, Naples, Italy; 438 CEINGE Biotecnologie Avanzate, Naples, Italy; 439 IRCCS SDN, Naples, Italy; 440 Unit of Respiratory Physiopathology, AORN dei Colli, Monaldi Hospital, Naples, Italy; 441 Division of Medical Genetics, Fondazione IRCCS Casa Sollievo della Sofferenza Hospital, San Giovanni Rotondo, Italy; 442 Department of Medical Sciences, Fondazione IRCCS Casa Sollievo della Sofferenza Hospital, San Giovanni Rotondo, Italy; 443 Infectious Diseases Clinic, Policlinico San Martino Hospital, IRCCS for Cancer Research, Genova, Italy; 444 Microbiology, Fondazione Policlinico Universitario Agostino Gemelli IRCCS, Catholic University of Medicine, Rome, Italy; 445 Department of Laboratory Sciences and Infectious Diseases, Fondazione Policlinico Universitario AGemelli IRCCS, Rome, Italy; 446 Department of Cardiovascular Diseases, University of Siena, Siena, Italy; 447 Otolaryngology Unit, University of Siena, Siena, Italy; 448 Department of Internal Medicine, ASST Valtellina e Alto Lario, Sondrio, Italy; 449 First Aid Department, Luigi Curto Hospital, Polla, Italy; 450 U.O.C. Laboratorio di Genetica Umana, Genova, Italy; 451 Infectious Diseases Clinics, University of Modena and Reggio Emilia, Modena, Italy; 452 Department of Respiratory Diseases, Azienda Ospedaliera di Cremona, Cremona, Italy; 453 U.O.C. Medicina, ASST Nord Milano, Ospedale Bassini, Milan, Italy; 454 Department of Cardiovascular, Neural and Metabolic Sciences, Istituto Auxologico Italiano, IRCCS, San Luca Hospital, Milan, Italy; 455 Department of Medicine and Surgery, University of Milano-Bicocca, Milan, Italy; 456 Center for Cardiac Arrhythmias of Genetic Origin, Istituto Auxologico Italiano, IRCCS, Milan, Italy; 457 Laboratory of Cardiovascular Genetics, Istituto Auxologico Italiano, IRCCS, Milan, Italy; 458 Unit of Infectious Diseases, ASST Papa Giovanni XXIII Hospital, Bergamo, Italy; 459 Department of Cardiology, Institute of Montescano, Istituti Clinici Scientifici Maugeri, IRCCS, Pavia, Italy; 460 Department of Cardiac Rehabilitation, Institute of Tradate (VA), Istituti Clinici Scientifici Maugeri, IRCCS, Pavia, Italy; 461 Cardiac Rehabilitation Unit, Fondazione Salvatore Maugeri, IRCCS, Scientific Institute of Milan, Milan, Italy; 462 IRCCS CMondino Foundation, Pavia, Italy; 463 Medical Genetics Unit, Meyer Children’s University Hospital, Florence, Italy; 464 Department of Medicine, Pneumology Unit, Misericordia Hospital, Grosseto, Italy; 465 Department of Preventive Medicine, Azienda USL Toscana Sud Est, Arezzo, Italy; 466 Department of Anesthesia and Intensive Care Unit, ASST Fatebenefratelli Sacco, Luigi Sacco Hospital, Polo Universitario, University of Milan, Milan, Italy; 467 Health Management, Azienda USL Toscana Sudest, Arezzo, Italy; 468 Department of Mathematics, University of Pavia, Pavia, Italy; 469 Independent researcher, Milan, Italy; 470 Scuola Normale Superiore, Pisa, Italy; 471 CNR-Consiglio Nazionale delle Ricerche, Istituto di Biologia e Biotecnologia Agraria (IBBA), Milano, Italy; 473 Veos Digital, Milan, Italy; 475 Core Research Laboratory, ISPRO, Florence, Italy; 478 Division of Infectious Diseases and Immunology, Fondazione IRCCS Policlinico San Matteo, Pavia, Italy; 479 Department of Molecular and Translational Medicine, University of Brescia, Brescia, Italy; 480 Clinical Chemistry Laboratory, Cytogenetics and Molecular Genetics Section, Diagnostic Department, ASST Spedali Civili di Brescia, Brescia, Italy; 481 Department of Medical and Surgical Sciences for Children and Adults, University of Modena and Reggio Emilia, Modena, Italy; 482 Department of Molecular Medicine, University of Padova, Padua, Italy; 483 Laboratory of Regulatory and Functional Genomics, Fondazione IRCCS Casa Sollievo della Sofferenza, San Giovanni Rotondo, Italy; 484 Clinical Trial Office, Fondazione IRCCS Casa Sollievo della Sofferenza Hospital, San Giovanni Rotondo, Italy; 485 Department of Health Sciences, University of Genova, Genova, Italy; 486 Oncologia Medica e Ufficio Flussi Sondrio, Sondrio, Italy; 487 Local Health Unit, Pharmaceutical Department of Grosseto, Toscana Sud Est Local Health Unit, Grosseto, Italy; 488 Independent researcher, Milan, Italy; 489 Direzione Scientifica, Istituti Clinici Scientifici Maugeri IRCCS, Pavia, Italy; 490 Fondazione per la ricerca Ospedale di Bergamo, Bergamo, Italy; 491 Allelica, New York, NY USA; 493 School of Basic and Medical Biosciences, Faculty of Life Sciences and Medicine, King’s College London, London, UK; 494 Medical and Population Genomics, Wellcome Sanger Institute, Hinxton, UK; 495 Bradford Institute for Health Research, Bradford Teaching Hospitals National Health Service (NHS) Foundation Trust, Bradford, UK; 497 Institute of Population Health Sciences, Queen Mary University of London, London, UK; 498 Genes & Health, Blizard Institute, Queen Mary University of London, London, UK; 499 Institute of Population Health Sciences, Queen Mary University of London, London, UK; 500 Department of Biostatistics, University of Michigan, Ann Arbor, MI USA; 501 Heart Institute (InCor), University of Sao Paulo Med School, São Paulo, Brazil; 502 Genentech, San Francisco, CA USA; 503 DNA Link Inc., Seoul, Republic of Korea; 504 Seoul National University Hospital Gangnam Center, Seoul, Republic of Korea; 505 Division of Infectious Diseases, Department of Internal Medicine, Chungnam National University School of Medicine, Daejeon, Republic of Korea; 506 East Kent Hospitals NHS Foundation Trust, Canterbury, UK; 507 Department of Internal Medicine, School of Medicine, Kyungpook National University, Daegu, Republic of Korea; 508 Division of Infectious Diseases, Department of Internal Medicine, Incheon Medical Center, Incheon, Republic of Korea; 509 Department of Infectious Diseases, Keimyung University Dongsan Hospital, Keimyung University School of Medicine, Daegu, Republic of Korea; 510 Department of Internal Medicine, Pusan National University School of Medicine and Medical Research Institute, Pusan National University Hospital, Busan, Republic of Korea; 511 Division of Infectious Diseases, Department of Internal Medicine, Myongji Hospital, Goyang, Republic of Korea; 512 Institute for Health Promotion, Graduate School of Public Health, Yonsei University, Seoul, Republic of Korea; 513 Division of Cardiovascular Medicine, Stanford University, Stanford, CA USA; 514 Department of Medicine, Stanford University, Stanford, CA USA; 515 Department of Genetics, Stanford University, Stanford, CA USA; 516 Department of Biomedical Data Science, Stanford University, Stanford, CA USA; 518 Department of Pathology, Stanford University, Stanford, CA USA; 519 Illumina, San Diego, CA USA; 520 Computational Biology, Drug Discovery Sciences, Takeda Pharmaceuticals, Boston, MA USA; 521 Department of Computational Biology, Swiss Institute of Bioinformatics (SIB), University of Lausanne, Lausanne, Switzerland; 523 Royal Victoria Hospital, Belfast, UK; 524 Chelsea & Westminster NHS Foundation Trust, London, UK; 525 Northampton General Hospital NHS Trust, Northampton, UK; 526 Wrexham Maelor Hospital, Wrexham, UK; 527 University College Dublin, St Vincent’s University Hospital, Dublin, Ireland; 528 University Hospitals Coventry & Warwickshire NHS Trust, Coventry, UK; 529 Watford General Hospital, Watford, UK; 530 NIHR Health Protection Research Unit, Institute of Infection, Veterinary and Ecological Sciences, Faculty of Health and Life Sciences, University of Liverpool, Liverpool, UK; 531 Queen Alexandra Hospital (Hampshire), Portsmouth Hospital Trust, Portsmouth, UK; 532 Princess Royal Hospital, Brighton & Sussex Universities Hospitals NHS Trust, Brighton, UK; 533 Bassettlaw Hospital, Doncaster and Bassetlaw, Worksop, UK; 534 Darent Valley Hospital, Dartford & Gravesham NHS Trust, Dartford, UK; 535 High Containment Laboratories, University of Birmingham, Birmingham, UK; 536 Queen Elizabeth the Queen Mother Hospital, Margate, UK; 537 John Radcliffe Hospital, Oxford University Hospitals NHS Foundation Trust, Oxford, UK; 538 Royal Albert Edward Infirmary (Wigan), Wrightington, Wigan and Leigh, Wigan, UK; 539 Manchester Royal Infirmary, Manchester University Hospitals NHS Foundation Trust, Manchester, UK; 540 Furness General Hospital, Morecambe Bay NHS Foundation Trust, Barrow-in-Furness, UK; 541 Castle Hill Hospital, Hull University Teaching Hospital Trust, Hull, UK; 542 Hillingdon Hospital, Hillingdon Hospital, London, UK; 543 St Thomas Hospital, Guys and St Thomas Foundation Trust, London, UK; 544 University Hospitals Coventry and Warwickshire, Coventry, UK; 545 St Michaels Hospital (Bristol), University Hospitals Bristol and Weston NHS Foundation Trust, Bristol, UK; 546 Stepping Hill Hospital, Stockport NHS Foundation Trust, Manchester, UK; 547 Royal Liverpool Hospital, Liverpool University Hospitals NHS Foundation Trust, Liverpool, UK; 548 Bristol Royal Hospital (Children’s), University Hospitals Bristol and Weston NHS Foundation Trust, Bristol, UK; 549 Scarborough Hospital, York Teaching Hospitals NHS Foundation Trust, York, UK; 550 Liverpool Heart & Chest Hospital, Liverpool Heart & Chest NHS Foundation Trust, Liverpool, UK; 551 James Paget University Hospital, James Paget University Hospitals NHS Foundation Trust, Great Yarmouth, UK; 552 The James Cook University Hospital, South Tees NHS Foundation Trust, Middlesbrough, UK; 553 Aberdeen Royal Infirmary, Grampian, Aberdeen, UK; 554 University of Edinburgh, Edinburgh, UK; 555 Royal Devon and Exeter Hospital, Royal Devon and Exeter NHS Foudation Trust, Exeter, UK; 556 Worcestershire Royal Hospital, Worcestershire Acute Hospitals NHS Trust, Worcester, UK; 557 Conquest Hospital, Hastings, East Sussex Healthcare NHS Trust, Seaford, UK; 558 Dorset County Hospital, Dorset County Hospital NHS Foundation Trust, Dorchester, UK; 559 Royal Bournemouth General Hospital, University Hospitals Dorset NHS Foundation Trust, Bournemouth, UK; 560 Harrogate Hospital, Harrogate and District NHS Foundation Trust, Harrogate, UK; 561 Burnley General Teaching Hospital, East Lancashire Hospitals NHS Hospitals, Burnley, UK; 562 Torbay Hospital, Torbay & South Devon NHS Foundation Trust, Torquay, UK; 563 Royal Hallamshire Hospital, Sheffield Teaching Hospitals NHS Foundation Trust, Sheffield, UK; 564 St Georges Hospital (Tooting), St Georges University Hospitals NHS Foundation Trust, London, UK; 565 Blackpool Victoria Hospital, Blackpool Teaching Hospitals NHS Foundation Trust, Blackpool, UK; 566 The Royal London Hospital, Barts Health NHS Trust, London, UK; 567 Salford Royal NHS Foundation Trust, Salford Royal NHS Foundation Trust, Manchester, UK; 568 University Hospital of North Durham, County Durham and Darlington Foundation Trust, Durham, UK; 569 Norfolk and Norwich University Hospital, Norfolk and Norwich University Hospital NHS Foundation Trust, Norwich, UK; 572 Fairfield General Hospital, Pennine Acute Hospitals NHS Trust, Manchester, UK; 573 Hereford County Hospital, Wye Valley NHS Trust, Hereford, UK; 574 Southampton General Hospital, University Hospital Southampton NHS Foundation Trust, Southampton, UK; 575 Northampton General Hospital, Northampton General Hospital NHS Trust, Northampton, UK; 576 University Hospital of Wales, Cardiff and Vale University Health Board, Cardiff, UK; 577 University of Bristol, Bristol, UK; 578 Leighton Hospital, Mid Cheshire Hospitals NHS Foundation Trust, Crewe, UK; 579 Diana Princess of Wales Hospital (Grimsby), North Lincolnshire & Goole, Grimsby, UK; 580 Manor Hospital, Walsall Healthcare NHS Trust, Walsall, UK; 581 Addenbrookes Hospital, Cambridge University Hospital NHS Foundation Trust, Cambridge, UK; 582 West Suffolk Hospital, West Suffolk Hospital NHS Foundation Trust, Bury St Edmunds, UK; 583 Basingstoke and North Hampshire Hospital, Hampshire Hospitals NHS Foundation Trust, Basingstoke, UK; 584 North Cumbria Integrated Care NHS Foundation Trust, Carlisle, UK; 585 Warwick Hospital, South Warwickshire NHS Foundation Trust, Warwick, UK; 586 Birmingham Women’s and Children’s Hospital, Birmingham Women’s and Children’s Hospital NHS Foundation Trust, Birmingham, UK; 587 Nottingham City Hospital, Nottingham University Hospitals NHS Trust, Nottingham, UK; 588 Glangwili Hospital Child Health Section, Hywel Dda University Health Board, Carmarthen, UK; 589 Alder Hey Children’s Hospital, Alder Hey Children’s NHS Foundation Trust, Liverpool, UK; 590 Bronglais General Hospital, Hywel Dda University Health Board, Aberystwyth, UK; 591 Worthing Hospital, Western Sussex Hospitals NHS Foundation Trust, Worthing, UK; 592 Rotheram District General Hospital, The Rotheram NHS Foundation Trust, Rotherham, UK; 593 Royal Free Hospital, Royal Free London NHS Foundation Trust, London, UK; 594 Homerton Hospital, Homerton University Hospital NHS Foundation Trust, London, UK; 595 Airedale Hospital, Airedale NHS Foundation Trust, Keighley, UK; 596 Basildon Hospital, Basildon and Thurrock University Hospitals NHS Foundation Trust, Basildon, UK; 597 The Christie NHS Foundation Trust, Manchester, UK; 598 Queen Elizabeth Hospital (Greenwich), Lewisham and Greenwich NHS Trust, London, UK; 599 The Whittington Hospital, Whittington Health NHS Trust, London, UK; 600 Sheffield Children’s Hospital, Sheffield Children’s NHS Foundation Trust, Sheffield, UK; 601 Royal United Hospital, Bath, Royal United Hospitals Bath NHS Foundation Trust, Bath, UK; 602 Western General Hospital, Edinburgh, UK; 603 Mid and South Essex NHS Foundation Trust, Basildon, UK; 604 Hinchingbrooke Hospital, North West Anglia NHS Foundation Trust, Peterborough, UK; 605 Royal Preston Hospital, Lancashire Teaching Hospitals NHS Foundation Trust, Preston, UK; 606 University Hospital (Coventry), University Hospitals Coventry and Warwickshire, Coventry, UK; 607 The Walton Centre, The Walton Centre, Liverpool, UK; 608 Hull Royal Infirmary, Hull University Teaching Hospital Trust, Hull, UK; 609 Darlington Memorial Hospital, County Durham and Darlington Foundation Trust, Darlington, UK; 610 Queen Elizabeth Hospital (Gateshead), Gateshead NHS Foundation Trust, Newcastle, UK; 611 Warrington Hospital, Warrington & Halton Hospitals NHS Foundation Trust, Warrington, UK; 612 University Hospitals Bristol and Weston NHS Foundation Trust, Bristol, UK; 613 St Mary’s Hospital (Isle of Wight), Isle of Wight NHS Trust, Isle of Wight, UK; 614 The Maidstone Hospital, Maidstone & Tunbridge Wells NHS Trust, Maidstone, UK; 615 Huddersfield Royal, Calderdale and Huddersfield NHS Foundation Trust, Huddersfield, UK; 616 Royal Surrey County Hospital, Guildford, UK; 617 Countess of Chester Hospital, Countess of Chester Hospital NHS Foundation Trust, Chester, UK; 618 Frimley Park Hospital, Frimley Health Foundation Trust, Frimley, UK; 620 Leeds General Infirmary, Leeds Teaching Hospitals, Leeds, UK; 621 North Middlesex Hospital, North Middlesex University Hospital NHS Trust, London, UK; 622 Arrowe Park Hospital, Wirral University Teaching Hospital NHS Foundation Trust, Wirral, UK; 623 Great Ormond Street Hospital, Great Ormond Street Hospital for Children NHS Foundation Trust, London, UK; 624 Royal Shrewsbury Hospital, Shrewsbury and Telford Hospital NHS Trust, Shrewsbury, UK; 625 East Surrey Hospital (Redhill), Surrey & Sussex Healthcare, Redhill, UK; 626 Burton Hospital, University Hospitals of Derby & Burton NHS Foundation Trust, Burton-on-Trent, UK; 627 Kent and Canterbury Hospital, East Kent Hospitals NHS Foundation Trust, Canterbury, UK; 628 Weston Area General Trust, University Hospitals Bristol and Weston NHS Foundation Trust, Bristol, UK; 629 Luton and Dunstable University Hospital, Luton, UK; 630 Glasgow Royal Infirmary, Greater Glasgow and Clyde, Glasgow, UK; 631 Derbyshire Healthcare, Derbyshire Healthcare NHS Foundation Trust, Derby, UK; 632 Macclesfield General Hospital, East Cheshire NHS Foundation Trust, Macclesfield, UK; 633 Chelsea and Westminster Hospital, Chelsea and Westminster NHS Trust, London, UK; 634 Institute of Microbiology and Infection, University of Birmingham, Birmingham, UK; 635 Prince Philip Hospital, Hwyel Dda University Health Board, Llanelli, UK; 636 George Eliot Hospital - Acute Services, George Eliot Hospital, Nuneaton, UK; 637 Kettering General Hospital, Kettering General Hospital NHS Foundation Trust, Kettering, UK; 638 Birmingham Heartlands Hospital, Birmingham, UK; 639 Russells Hall Hospital, The Dudley Group NHS Foundation Trust, Dudley, UK; 640 Harefield Hospital, Royal Brompton & Harefield Trust, London, UK; 641 Lister Hospital, East and North Hertfordshire NHS Trust, Stevenage, UK; 642 Musgrove Park Hospital (Taunton & Somerset), Somerset NHS Foundation Trust, Taunton, UK; 643 Queen’s Hospital, Havering (Romford), Barking, Havering and Redbridge University Hospitals NHS Trust, London, UK; 644 Southport & Formby District General Hospital, Southport and Ormskirk Hospital NHS Trust, Southport, UK; 645 New Cross Hospital, The Royal Wolverhampton NHS Trust, Wolverhampton, UK; 646 King’s College Hospital, London, UK; 647 The Royal Victoria Infirmary, Newcastle Hospitals NHS Trust, Newcastle, UK; 648 The Great Western Hospital, Great Western Hospitals NHS Foundation Trust, Swindon, UK; 649 Ninewells Hospital, Tayside, Dundee, UK; 650 Poole Hospital NHS Trust, Poole, UK; 651 Burton Hospital, University Hospitals of Derby & Burton NHS Foundation Trust, Derby, UK; 652 William Harvey Hospital, Ashford, East Kent Hospitals NHS Foundation Trust, Willesborough, UK; 653 King’s Mill Hospital, Sherwood Forest Hospitals NHS Foundation Trust, Sutton-in-Ashfield, UK; 654 Liverpool Women’s NHS Foundation Trust, Liverpool, UK; 655 Dewsbury Hospital, Mid Yorkshire Hospitals NHS Trust, Dewsbury, UK; 656 Northern Devon District Hospital, Northern Devon Healthcare NHS Trust, Barnstaple, UK; 657 Tameside General Hospital, Tameside and Glossop Integrated Care NHS Foundation Trust, Manchester, UK; 658 Sandwell General Hospital, Sandwell and West Birmingham Hospitals NHS Trust, Birmingham, UK; 659 Broomfield Hospital, Mid and South Essex University Hospitals Group, Broomfield, UK; 660 Wycombe Hospital, Buckingham Healthcare NHS Trust, Wycombe, UK; 661 University Hospital of North Tees, North Tees and Hartlepool NHS Trust, Stockton-on-Tees, UK; 662 Royal Manchester Children’s Hospital, Manchester University Hospitals NHS Foundation Trust, Manchester, UK; 663 Bedford Hospital, Bedford, UK; 664 Colchester General Hospital, East Suffolk and North Essex Foundation Trust, Colchester, UK; 665 Queen Elizabeth Hospital (Birmingham) and Heartlands, University Hospital Birmingham NHS Foundation Trust, Birmingham, UK; 666 Chesterfield Royal Hospital, Chesterfield Royal Hospital NHS Foundation Trust, Chesterfield, UK; 667 Princess Alexandra Hospital, The Princess Alexandra Hospital NHS Trust, Harlow, UK; 668 Watford General Hospital, West Hertfordshire Hospitals NHS Trust, Watford, UK; 669 Milton Keynes Hospital, Milton Keynes University Hospital NHS Foundation Trust, Milton Keynes, UK; 670 Royal Bolton General Hospital, Bolton Foundation Trust, Bolton, UK; 671 Royal Gwent (Newport), Aneurin Bevan University Health Board, Newport, UK; 672 The Royal Marsden Hospital (London), The Royal Marsden NHS Foundation Trust, London, UK; 673 Queen Victoria Hospital (East Grinstead), Queen Victoria Hospital NHS Foundation Trust, East Grinstead, UK; 674 County Hospital (Stafford), University Hospitals of North Midlands NHS Trust, Stafford, UK; 675 Whiston Hospital, St Helen’s & Knowlsey Hospitals NHS Trust, Prescot, UK; 676 Croydon University Hospital, London, UK; 677 Gloucester Royal, Gloucestershire Hospitals NHS Foundation Trust, Gloucester, UK; 678 Medway Maritime Hospital, Medway Maritime NHS Trust, Gillingham, UK; 679 Royal Papworth Hospital Everard, Royal Papworth Hospital NHS Foundation Trust, Cambridge, UK; 680 Derriford (Plymouth), University Hospital Plymouth NHS Trust, Plymouth, UK; 681 St Helier Hospital, Epsom and St Helier University Hospital NHS Trust, London, UK; 682 Royal Berkshire Hospital, Royal Berkshire Foundation Trust, London, UK; 683 Bradford Royal Infirmary, Bradford Teaching Hospitals NHS Foundation Trust, Bradford, UK; 684 Northwick Park, London North West University Hospital Trust, London, UK; 685 Ealing Hospital, London North West University Hospital Trust, London, UK; 686 Royal Cornwall Hospital (Tresliske), Royal Cornwall NHS Trust, Truro, UK; 687 Ashford Hospital, Ashford & St Peter’s Hospital, Stanwell, UK; 688 Leicester Royal Infirmary (includes Glenfield Site), University Hospitals of Leicester, Leicester, UK; 689 Grantham and District Hospital, United Lincolnshire Hospitals NHS Trust, Grantham, UK; 690 University Hospital Aintree, Liverpool University Hospitals NHS Foundation Trust, Liverpool, UK; 691 North Tyneside General Hospital, Northumbria Healthcare NHS Trust, North Shields, UK; 692 Queen Elizabeth Hospital (King’s Lynn), Queen Elizabeth Hospital King’s Lynn NHS Foundation Trust, King’s Lynn, UK; 693 The Crick Institute, London, UK; 695 William Harvey Research Institute, Barts and the London School of Medicine and Dentistry, Queen Mary University of London, London, UK; 696 Centre for Genomic and Experimental Medicine, Institute of Genetics and Molecular Medicine, University of Edinburgh, Western General Hospital, Edinburgh, UK; 697 Intensive Care National Audit & Research Centre, London, UK; 700 Wellcome Centre for Human Genetics, University of Oxford, Oxford, UK; 705 Centre for Inflammation Research, The Queen’s Medical Research Institute, University of Edinburgh, Edinburgh, UK; 706 Great Ormond Street Hospital for Children NHS Foundation Trust, London, UK; 707 Biostatistics Group, School of Life Sciences, Sun Yat-sen University, Guangzhou, China; 708 Centre for Global Health Research, Usher Institute of Population Health Sciences and Informatics, Edinburgh, UK; 709 Department of Medical Epidemiology and Biostatistics, Karolinska Institutet, Stockholm, Sweden; 710 Institute for Molecular Bioscience, The University of Queensland, Brisbane, Queensland, Australia; 711 School of Life Sciences, Westlake University, Hangzhou, China; 712 Westlake Laboratory of Life Sciences and Biomedicine, Westlake University, Hangzhou, China; 714 Centre for Medical Informatics, The Usher Institute, University of Edinburgh, Edinburgh, UK; 715 Liverpool Clinical Trials Centre, University of Liverpool, Liverpool, UK; 716 Centre for Health Informatics, Division of Informatics, Imaging and Data Science, School of Health Sciences, Faculty of Biology, Medicine and Health, University of Manchester, Manchester Academic Health Science Centre, Manchester, UK; 717 MRC Human Genetics Unit, MRC Institute of Genetics and Molecular Medicine, University of Edinburgh, Edinburgh, UK; 718 School of Informatics, University of Edinburgh, Edinburgh, UK; 719 Royal Hospital for Children, Glasgow, UK; 721 MRC-University of Glasgow Centre for Virus Research, Institute of Infection, Immunity and Inflammation, College of Medical, Veterinary and Life Sciences, University of Glasgow, Glasgow, UK; 722 Centre for Tropical Medicine and Global Health, Nuffield Department of Medicine, University of Oxford, Oxford, UK; 724 Department of Anaesthesia and Intensive Care, The Chinese University of Hong Kong, Prince of Wales Hospital, Hong Kong, China; 725 Department of Critical Care Medicine, Queen’s University and Kingston Health Sciences Centre, Kingston, Ontario, Canada; 726 Wellcome-Wolfson Institute for Experimental Medicine, Queen’s University Belfast, Belfast, UK; 727 Department of Intensive Care Medicine, Royal Victoria Hospital, Belfast, UK; 728 UCL Centre for Human Health and Performance, London, UK; 729 Clinical Research Centre at St Vincent’s University Hospital, University College Dublin, Dublin, Ireland; 730 National Heart and Lung Institute, Imperial College London, London, UK; 731 Imperial College Healthcare NHS Trust London, London, UK; 732 NIHR Health Protection Research Unit for Emerging and Zoonotic Infections, Institute of Infection, Veterinary and Ecological Sciences University of Liverpool, Liverpool, UK; 733 Respiratory Medicine, Alder Hey Children’s Hospital, Institute in The Park, University of Liverpool, Alder Hey Children’s Hospital, Liverpool, UK; 734 Department of Intensive Care Medicine, Guy’s and St Thomas NHS Foundation Trust, London, UK; 735 Department of Medicine, University of Cambridge, Cambridge, UK; 736 Airedale General Hospital, Keighley, UK; 737 Barts Health NHS Trust, London, UK; 738 Basildon Hospital, Basildon, UK; 739 BHRUT (Barking Havering) - Queens Hospital and King George Hospital, Romford, UK; 740 Bradford Royal Infirmary, Bradford, UK; 741 Bronglais General Hospital, Aberystwyth, UK; 742 Broomfield Hospital, Chelmsford, UK; 743 Calderdale Royal Hospital, Halifax, UK; 744 Charing Cross Hospital, St Mary’s Hospital and Hammersmith Hospital, London, UK; 745 Barnet Hospital, London, UK; 746 Birmingham Children’s Hospital, Birmingham, UK; 747 St John’s Hospital Livingston, Livingston, UK; 748 Aberdeen Royal Infirmary, Aberdeen, UK; 749 Addenbrooke’s Hospital, Cambridge, UK; 750 Aintree University Hospital, Liverpool, UK; 752 Arrowe Park Hospital, Wirral, UK; 753 Ashford and St Peter’s Hospital, Lyne, UK; 754 Basingstoke and North Hampshire Hospital, Basingstoke, UK; 755 Borders General Hospital, Melrose, UK; 756 Chesterfield Royal Hospital Foundation Trust, Chesterfield, UK; 757 Eastbourne District General Hospital, East Sussex, UK and Conquest Hospital, Eastbourne, UK; 758 Barnsley Hospital, Barnsley, UK; 759 Blackpool Victoria Hospital, Blackpool, UK; 760 East Surrey Hospital, Redhill, UK; 761 Good Hope Hospital, Birmingham, UK; 762 Hereford County Hospital, Hereford, UK; 763 Hull Royal Infirmary, Hull, UK; 765 Kent & Canterbury Hospital, Canterbury, UK; 766 Manchester Royal Infirmary, Manchester, UK; 767 Nottingham University Hospital, Nottingham, UK; 768 Pilgrim Hospital, Lincoln, UK; 769 Queen Elizabeth Hospital, Birmingham, UK; 770 Salford Royal Hospital, Manchester, UK; 771 Tameside General Hospital, Ashton-under-Lyne, UK; 772 The Tunbridge Wells Hospital and Maidstone Hospital, Maidstone, UK; 773 The Royal Oldham Hospital, Manchester, UK; 774 The Royal Papworth Hospital, Cambridge, UK; 775 University College Hospital, London, UK; 776 Withybush General Hospital, Haverfordwest, UK; 777 Wythenshawe Hospital, Manchester, UK; 778 Yeovil Hospital, Yeovil, UK; 779 Cumberland Infirmary, Carlisle, UK; 780 Darent Valley Hospital, Dartford, UK; 781 Dumfries and Galloway Royal Infirmary, Dumfries, UK; 782 Ealing Hospital, London, UK; 783 Fairfield General Hospital, Bury, UK; 784 George Eliot Hospital NHS Trust, Nuneaton, UK; 785 Glan Clwyd Hospital, Bodelwyddan, UK; 786 Glangwili General Hospital, Camarthen, UK; 787 The Great Western Hospital, Swindon, UK; 788 Guys and St Thomas’ Hospital, London, UK; 789 Harefield Hospital, London, UK; 790 Harrogate and District NHS Foundation Trust, Harrogate, UK; 792 James Paget University Hospital NHS Trust, Great Yarmouth, UK; 794 King’s Mill Hospital, Nottingham, UK; 795 Kingston Hospital, Kingston, UK; 796 Lincoln County Hospital, Lincoln, UK; 797 Liverpool Heart and Chest Hospital, Liverpool, UK; 798 Macclesfield District General Hospital, Macclesfield, UK; 799 Medway Maritime Hospital, Gillingham, UK; 800 Milton Keynes University Hospital, Milton Keynes, UK; 801 Morriston Hospital, Swansea, UK; 802 National Hospital for Neurology and Neurosurgery, London, UK; 803 Norfolk and Norwich University hospital (NNUH), Norwich, UK; 804 North Middlesex University Hospital NHS Trust, London, UK; 806 Northumbria Healthcare NHS Foundation Trust, North Shields, UK; 807 Peterborough City Hospital, Peterborough, UK; 808 Prince Charles Hospital, Merthyr Tydfil, UK; 809 Royal Sussex County Hospital, Brighton, UK; 810 Princess Royal Hospital, Haywards Heath, UK; 811 Princess of Wales Hospital, Llantrisant, UK; 812 Queen Alexandra Hospital, Portsmouth, UK; 813 Queen Elizabeth Hospital, London, UK; 815 Queen Victoria Hospital, East Grinstead, UK; 816 Queen’s Hospital Burton, Burton-On-Trent, UK; 817 Raigmore Hospital, Inverness, UK; 818 Rotherham General Hospital, Rotherham, UK; 819 Royal Blackburn Teaching Hospital, Blackburn, UK; 820 Royal Preston Hospital, Preston, UK; 821 Royal Surrey County Hospital, Guildford, UK; 822 Royal Albert Edward Infirmary, Wigan, UK; 823 The Royal Alexandra Children’s Hospital, Brighton, UK; 824 Royal Alexandra Hospital, Paisley, UK; 825 Royal Bolton Hospital, Bolton, UK; 826 University Hospitals Dorset NHS Foundation Trust, Dorchester, UK; 827 Royal Brompton Hospital, London, UK; 828 Imperial College London, London, UK; 829 Royal Cornwall Hospital, Truro, UK; 830 Royal Free Hospital, London, UK; 831 Royal Glamorgan Hospital, Pontyclun, UK; 832 Royal Gwent Hospital, Newport, UK; 833 Northern General Hospital, Sheffield, UK; 834 Royal Hampshire County Hospital, Winchester, UK; 835 Royal Manchester Children’s Hospital, Manchester, UK; 836 Royal Stoke University Hospital, Stoke-on-Trent, UK; 837 Salisbury District Hospital, Salisbury, UK; 838 Sandwell General Hospital, Birmingham, UK; 839 Scarborough General Hospital, Scarborough, UK; 840 Scunthorpe General Hospital, Scunthorpe, UK; 841 Southmead Hospital, Bristol, UK; 842 St George’s Hospital, London, UK; 843 St Mary’s Hospital, Newport, UK; 844 Stoke Mandeville Hospital, Aylesbury, UK; 845 Sunderland Royal Hospital, Sunderland, UK; 846 Alexandra Hospital, Redditch and Worcester Royal Hospital, Worcester, UK; 847 The Christie NHS Foundation Trust, Manchester, UK; 848 The Queen Elizabeth Hospital, King’s Lynn, UK; 849 The Royal Liverpool University Hospital, Liverpool, UK; 850 The Royal Marsden NHS Foundation Trust, London, UK; 851 Torbay Hospital, Torquay, UK; 852 University Hospital Monklands, Airdrie, UK; 853 University Hospital Lewisham, London, UK; 854 University Hospital North Durham, Darlington, UK; 855 University Hospital of North Tees, Stockton-on-Tees, UK; 856 University Hospital of Wales, Cardiff, UK; 857 University Hospital Wishaw, Wishaw, UK; 858 Victoria Hospital, Kirkcaldy, UK; 859 Warrington General Hospital, Warrington, UK; 860 West Cumberland Hospital, Whitehaven, UK; 861 Western Sussex Hospitals, Chichester, UK; 862 Whiston Hospital, Prescot, UK; 863 York Hospital, York, UK; 864 Ysbyty Gwynedd, Bangor, UK; 865 Countess of Chester Hospital, Chester, UK; 866 Croydon University Hospital, Croydon, UK; 867 Diana Princess of Wales Hospital, Grimsby, UK; 868 Dorset County Hospital, Dorchester, UK; 869 Forth Valley Royal Hospital, Falkirk, UK; 870 Furness General Hospital, Barrow-in-Furness, UK; 871 Alder Hey Children’s Hospital, Liverpool, UK; 872 Derriford Hospital, Plymouth, UK; 873 Glasgow Royal Infirmary, Glasgow, UK; 874 Glenfield Hospital, Leicester, UK; 875 Gloucestershire Royal Hospital, Gloucester, UK; 876 Golden Jubilee National Hospital, Clydebank, UK; 877 Great Ormond St Hospital and UCL Great Ormond St Institute of Child Health NIHR Biomedical Research Centre, London, UK; 878 Homerton University Hospital Foundation NHS Trust, London, UK; 879 James Cook University Hospital, Middlesbrough, UK; 880 John Radcliffe Hospital, Oxford, UK; 881 Leicester Royal Infirmary, Leicester, UK; 882 Lister Hospital, Stevenage, UK; 883 New Cross Hospital, Wolverhampton, UK; 884 Royal Victoria Infirmary, Newcastle Upon Tyne, UK; 885 Ninewells Hospital, Dundee, UK; 886 North Devon District Hospital, Barnstaple, UK; 887 North Manchester General Hospital, Manchester, UK; 888 Northwick Park Hospital, London, UK; 889 Prince Philip Hospital, Lianelli, UK; 890 Pinderfields General Hospital, Wakefield, UK; 891 Poole Hospital, Poole, UK; 892 Royal Shrewsbury Hospital, Shrewsbury, UK; 893 Princess Royal Hospital, Telford, UK; 899 Queen Elizabeth Hospital Gateshead, Gateshead, UK; 900 Queen Elizabeth University Hospital, Glasgow, UK; 901 Royal Berkshire NHS Foundation Trust, Reading, UK; 902 Royal Derby Hospital, Derby, UK; 903 Royal Devon and Exeter Hospital, Exeter, UK; 904 Royal Infirmary of Edinburgh, Edinburgh, UK; 905 Royal Lancaster Infirmary, Lancaster, UK; 906 Royal United Hospital, Bath, UK; 907 Russells Hall Hospital, Dudley, UK; 908 Sheffield Children’s Hospital, Sheffield, UK; 909 Southampton General Hospital, Southampton, UK; 910 Southend University Hospital, Westcliff-on-Sea, UK; 911 Southport and Formby District General Hospital, Ormskirk, UK; 912 St James’s University Hospital and Leeds General Infirmary, Leeds, UK; 913 Bristol Royal Infirmary, Bristol, UK; 914 Stepping Hill Hospital, Stockport, UK; 915 The Princess Alexandra Hospital, Harlow, UK; 916 University Hospital Crosshouse, Kilmarnock, UK; 917 University Hospital Hairmyres, East Kilbride, UK; 918 Craigavon Area Hospital, Craigavon, UK; 919 Warwick Hospital, Warwick, UK; 920 West Middlesex Hospital, Isleworth, UK; 922 Whittington Hospital, London, UK; 923 William Harvey Hospital, Ashford, UK; 932 Section of Molecular Virology, Imperial College London, London, UK; 933 Antimicrobial Resistance and Hospital Acquired Infection Department, Public Health England, London, UK; 934 Department of Infectious Disease, Imperial College London, London, UK; 935 National Infection Service, Public Health England, London, UK; 936 MRC-University of Glasgow Centre for Virus Research, Glasgow, UK; 937 Liverpool School of Tropical Medicine, Liverpool, UK; 938 Institute of Infection and Global Health, University of Liverpool, Liverpool, UK; 940 Virology Reference Department, National Infection Service, Public Health England, London, UK; 941 Department of Pharmacology, University of Liverpool, Liverpool, UK; 942 Nuffield Department of Medicine, University of Oxford, Oxford, UK; 944 Nottingham University Hospitals NHS Trust, Nottingham, UK; 945 Nuffield Department of Medicine, John Radcliffe Hospital, Oxford, UK; 946 ISARIC Global Support Centre, Centre for Tropical Medicine and Global Health, Nuffield Department of Medicine, University of Oxford, Oxford, UK; 947 Division of Infection and Immunity, University College London, London, UK; 948 Institute of Infection, Veterinary and Ecological Sciences, University of Liverpool, Liverpool, UK; 949 Centre for Clinical Infection and Diagnostics Research, Department of Infectious Diseases, School of Immunology and Microbial Sciences, King’s College London, London, UK; 950 Institute of Evolutionary Biology, University of Edinburgh, Edinburgh, UK; 951 Department of Pediatrics and Virology, Imperial College London, London, UK; 952 The Florey Institute for Host-Pathogen Interactions, Department of Infection, Immunity and Cardiovascular Disease, University of Sheffield, Sheffield, UK; 953 Division of Structural Biology, The Wellcome Centre for Human Genetics, University of Oxford, Oxford, UK; 955 Blood Borne Virus Unit, Virus Reference Department, National Infection Service, Public Health England, London, UK; 956 Department of Infection, Immunity and Cardiovascular Disease, University of Sheffield, Sheffield, UK; 957 Institute for Global Health, University College London, London, UK; 958 Molecular and Clinical Cancer Medicine, Institute of Systems, Molecular and Integrative Biology, University of Liverpool, Liverpool, UK; 959 Department of Child Life and Health, University of Edinburgh, Edinburgh, UK; 960 Section of Biomolecular Medicine, Division of Systems Medicine, Department of Metabolism, Digestion and Reproduction, Imperial College London, London, UK; 961 Department of Epidemiology and Biostatistics, School of Public Health, Faculty of Medicine, Imperial College London, London, UK; 962 National Phenome Centre, Department of Metabolism, Digestion and Reproduction, Imperial College London, London, UK; 963 Department of Molecular and Clinical Cancer Medicine, University of Liverpool, Liverpool, UK; 964 Institute of Translational Medicine, University of Liverpool, Liverpool, UK; 965 Intensive Care Unit, Royal Infirmary Edinburgh, Edinburgh, UK; 966 University of Liverpool, Liverpool, UK; 967 University of Glasgow, Glasgow, UK; 968 Edinburgh Clinical Research Facility, Western General Hospital, University of Edinburgh, Edinburgh, UK; 970 Department of Infectious Diseases, Leiden University Medical Center, Leiden, The Netherlands; 971 Cambridge University Hospitals NHS Foundation Trust, Cambridge, UK; 974 Genotek, Moscow, Russia; 975 Helix, San Mateo, CA USA; 976 Center for Genomic Medicine, Desert Research Institute, Reno, NV USA; 977 Renown Health, Reno, NV USA; 978 24Genetics, Boston, MA USA; 979 Hospital La Paz Institute for Health Research, Madrid, Spain; 980 Division of Pulmonary Medicine, Department of Medicine, Keio University School of Medicine, Tokyo, Japan; 981 Department of Statistical Genetics, Osaka University Graduate School of Medicine, Suita, Japan; 982 Laboratory of Statistical Immunology, Immunology Frontier Research Center (WPI-IFReC), Osaka University, Suita, Japan; 983 Integrated Frontier Research for Medical Science Division, Institute for Open and Transdisciplinary Research Initiatives, Osaka University, Suita, Japan; 984 Division of Health Medical Intelligence, Human Genome Center, the Institute of Medical Science, The University of Tokyo, Tokyo, Japan; 985 Laboratory of Viral Infection I, Department of Infection Control and Immunology, Ōmura Satoshi Memorial Institute & Graduate School of Infection Control Sciences, Kitasato University, Tokyo, Japan; 986 Department of Surgery, Keio University School of Medicine, Tokyo, Japan; 987 Department of Organoid Medicine, Keio University School of Medicine, Tokyo, Japan; 988 Department of Infectious Diseases, Keio University School of Medicine, Tokyo, Japan; 989 Department of Respiratory Medicine and Clinical Immunology, Osaka University Graduate School of Medicine, Suita, Japan; 990 Department of Immunopathology, Immunology Frontier Research Center (WPI-IFReC), Osaka University, Suita, Japan; 991 Institute of Research, Tokyo Medical and Dental University, Tokyo, Japan; 992 Department of Insured Medical Care Management, Tokyo Medical and Dental University Hospital of Medicine, Tokyo, Japan; 993 Genome Medical Science Project (Toyama), National Center for Global Health and Medicine, Chiba, Japan; 994 Division of Gastroenterology and Hepatology, Department of Medicine, Keio University School of Medicine, Tokyo, Japan; 995 M&D Data Science Center, Tokyo Medical and Dental University, Tokyo, Japan; 996 Department of Pathology and Tumor Biology Institute for the Advanced Study of Human Biology (WPI-ASHBi), Kyoto University, Kyoto, Japan; 997 Department of Medicine, Center for Hematology and Regenerative Medicine, Karolinska Institute, Stockholm, Sweden; 999 Department of Emergency and Critical Care Medicine, Keio University School of Medicine, Tokyo, Japan; 1000 Department of Anesthesiology, Keio University School of Medicine, Tokyo, Japan; 1001 Department of Laboratory Medicine, Keio University School of Medicine, Tokyo, Japan; 1002 Division of Infection Control and Prevention, Osaka University Hospital, Suita, Japan; 1003 Department of Biomedical Ethics and Public Policy, Osaka University Graduate School of Medicine, Suita, Japan; 1004 Center for Genomic Medicine, Kyoto University Graduate School of Medicine, Kyoto, Japan; 1005 Department of Pulmonary Medicine, Faculty of Medicine, University of Tsukuba, Tsukuba, Japan; 1006 Department of Neurosurgery, Faculty of Medicine, The University of Tokyo, Tokyo, Japan; 1007 Laboratory of Immune Regulation, Department of Microbiology and Immunology, Osaka University Graduate School of Medicine, Suita, Japan; 1009 Medical Innovation Promotion Center, Tokyo Medical and Dental University, Tokyo, Japan; 1010 Clinical Research Center, Tokyo Medical and Dental University Hospital of Medicine, Tokyo, Japan; 1011 Department of Medical Informatics, Tokyo Medical and Dental University Hospital of Medicine, Tokyo, Japan; 1012 Respiratory Medicine, Tokyo Medical and Dental University, Tokyo, Japan; 1013 Clinical Laboratory, Tokyo Medical and Dental University Hospital of Medicine, Tokyo, Japan; 1014 Department of Pathology and Tumor Biology, Kyoto University, Kyoto, Japan; 1015 Department of Respiratory Medicine, Graduate School of Medicine, Faculty of Medicine, Juntendo University, Tokyo, Japan; 1016 Department of Emergency and Disaster Medicine, Graduate School of Medicine, Faculty of Medicine, Juntendo University, Tokyo, Japan; 1017 Department of Cardiovascular Biology and Medicine, Graduate School of Medicine, Faculty of Medicine, Juntendo University, Tokyo, Japan; 1018 Department of Respiratory Medicine, Tokyo Women’s Medical University, Tokyo, Japan; 1019 Department of General Medicine, Tokyo Women’s Medical University, Tokyo, Japan; 1020 Department of Respiratory Medicine, Saitama Cardiovascular and Respiratory Center, Saitama, Japan; 1021 Kawasaki Municipal Ida Hospital, Kanagawa, Japan; 1022 Saitama Medical Center, Internal Medicine, Japan Community Healthcare Organization (JCHO), Saitama, Japan; 1023 Saitama City Hospital, Saitama, Japan; 1024 Division of Infection Control, Eiju General Hospital, Tokyo, Japan; 1025 Department of Pulmonary Medicine, Eiju General Hospital, Tokyo, Japan; 1026 Department of Respiratory Medicine, Osaka Saiseikai Nakatsu Hospital, Osaka, Japan; 1027 Division of Respirology, Rheumatology, and Neurology, Department of Internal Medicine Kurume University School of Medicine, Fukuoka, Japan; 1028 Department of Infection Control, Osaka Saiseikai Nakatsu Hospital, Osaka, Japan; 1029 Department of Infectious Diseases, Tosei General Hospital, Aichi, Japan; 1030 Fukujuji Hospital, Kiyose, Japan; 1031 Department of Emergency and Critical Care Medicine, Tokyo Women’s Medical University Medical Center East, Tokyo, Japan; 1032 Department of Medicine, Tokyo Women’s Medical University Medical Center East, Tokyo, Japan; 1033 Department of Pediatrics, Tokyo Women’s Medical University Medical Center East, Tokyo, Japan; 1034 Japan Community Healthcare Organization Kanazawa Hospital, Kanazawa, Japan; 1035 Division of Pulmonary Medicine, Department of Internal Medicine, Federation of National Public Service Personnel Mutual Aid Associations, Tachikawa Hospital, Tachikawa, Japan; 1036 Department of Respiratory Medicine, Japan Organization of Occupational Health and Safety, Kanto Rosai Hospital, Kawasaki, Japan; 1037 Department of General Internal Medicine, Japan Organization of Occupational Health and Safety, Kanto Rosai Hospital, Kawasaki, Japan; 1038 Department of Emergency and Critical Care Medicine, Kansai Medical University General Medical Center, Kirakata, Japan; 1039 Department of Respiratory Medicine, Kitasato University, Kitasato Institute Hospital, Tokyo, Japan; 1040 Ishikawa Prefectural Central Hospital, Kanazawa, Japan; 1041 Internal Medicine, Sano Kosei General Hospital, Sano, Japan; 1042 Saiseikai Yokohamashi Nanbu Hospital, Yokohama, Japan; 1043 Kanagawa Cardiovascular and Respiratory Center, Yokohama, Japan; 1044 Saiseikai Utsunomiya Hospital, Utsunomiya, Japan; 1045 Department of Respiratory Medicine, KKR Sapporo Medical Center, Sapporo, Japan; 1046 Internal Medicine, Internal Medicine Center, Showa University Koto Toyosu Hospital, Tokyo, Japan; 1047 Department of Respiratory Medicine, Toyohashi Municipal Hospital, Toyohashi, Japan; 1048 Keiyu Hospital, Yokohama, Japan; 1049 Department of Rheumatology, National Hospital Organization Hokkaido Medical Center, Sapporo, Japan; 1050 Department of Respiratory Medicine, National Hospital Organization Tokyo Medical Center, Tokyo, Japan; 1051 Department of Allergy, National Hospital Organization Tokyo Medical Center, Tokyo, Japan; 1052 Department of General Internal Medicine and Infectious Diseases, National Hospital Organization Tokyo Medical Center, Tokyo, Japan; 1053 Japanese Red Cross Musashino Hospital, Musashino, Japan; 1054 Department of Respiratory Medicine, Tohoku University Graduate School of Medicine, Sendai, Japan; 1055 Division of Respiratory Medicine, Department of Internal Medicine, Nihon University School of Medicine, Tokyo, Japan; 1056 Department of Emergency and Critical Care Medicine, St Marianna University School of Medicine, Kawasaki, Japan; 1057 Division of General Internal Medicine, Department of Internal Medicine, St Marianna University School of Medicine, Kawasaki, Japan; 1058 National Hospital Organization Kanazawa Medical Center, Kanazawa, Japan; 1059 Division of Infectious Diseases and Respiratory Medicine, Department of Internal Medicine, National Defense Medical College, Tokorozawa, Japan; 1060 Department of Emergency and Critical Care Medicine, Faculty of Medicine, Fukuoka University, Fukuoka, Japan; 1061 Department of Infection Control, Fukuoka University Hospital, Fukuoka, Japan; 1062 Tokyo Saiseikai Central Hospital, Tokyo, Japan; 1063 Department of Internal Medicine, Fukuoka Tokushukai Hospital, Kasuga, Japan; 1064 Department of Infectious Disease and Clinical Research Institute, National Hospital Organization Kyushu Medical Center, Fukuoka, Japan; 1065 Department of Respirology, National Hospital Organization Kyushu Medical Center, Fukuoka, Japan; 1067 Matsumoto City Hospital, Matsumoto, Japan; 1068 Uji-Tokushukai Medical Center, Uji, Japan; 1069 Department of Respiratory Medicine, Nagoya University Graduate School of Medicine, Nagoya, Japan; 1070 Department of Respiratory Medicine, Fujisawa City Hospital, Fujisawa, Japan; 1071 Sapporo City General Hospital, Sapporo, Japan; 1072 Department of Emergency and Critical Care Medicine, Chiba University Graduate School of Medicine, Chiba, Japan; 1073 Division of Respiratory Medicine, Social Welfare Organization Saiseikai Imperial Gift Foundation, Saiseikai Kumamoto Hospital, Kumamoto, Japan; 1074 Department of Anesthesiology and Intensive Care Medicine, Kyoto Prefectural University of Medicine, Kyoto, Japan; 1075 Ome Municipal General Hospital, Ome, Japan; 1076 Hanwa Daini Hospital, Osaka, Japan; 1077 Department of Respiratory Internal Medicine, St Marianna University School of Medicine, Yokohama-City Seibu Hospital, Yokohama, Japan; 1078 Division of Hematology, Department of Internal Medicine, St Marianna University Yokohama-City Seibu Hospital, Yokohama, Japan; 1079 Division of Pulmonary Medicine, Department of Medicine, Tokai University School of Medicine, Tokai University School of Medicine, Tokyo, Japan; 1080 Division of Pulmonary Medicine, Department of Medicine, Tokai University School of Medicine, Tokyo, Japan; 1081 National Hospital Organization Kumamoto Medical Center, Kumamoto, Japan; 1082 Department of Respiratory Medicine, Tokyo Medical University Hospital, Tokyo, Japan; 1083 Department of Respiratory Medicine, Japanese Red Cross Medical Center, Tokyo, Japan; 1084 JA Toride Medical Hospital, Toride, Japan; 1085 Japan Organization of Occupational Health and Safety Okayama Rosai Hospital, Okayama, Japan; 1086 Emergency and Disaster Medicine, Graduate School of Medicine, Gifu University School of Medicine, Gifu, Japan; 1087 Niigata University, Niigata, Japan; 1088 National Hospital Organization Kyoto Medical Center, Kyoto, Japan; 1089 Research Institute for Diseases of the Chest, Graduate School of Medical Sciences, Kyushu University, Fukuoka, Japan; 1090 Department of Medicine and Biosystemic Science, Kyushu University Graduate School of Medical Sciences, Fukuoka, Japan; 1091 Department of Emergency and Critical Care Medicine, Tsukuba University, Tsukuba, Japan; 1092 Department of Nephrology, Faculty of Medicine, University of Tsukuba, Tsukuba, Japan; 1093 Department of Hematology, Faculty of Medicine, University of Tsukuba, Tsukuba, Japan; 1094 National Hospital Organization Tokyo Hospital, Tokyo, Japan; 1095 Fujioka General Hospital, Fujioka, Japan; 1096 Division of Respiratory Medicine and Allergology, Department of Medicine, School of Medicine, Showa University, Tokyo, Japan; 1097 Department of Pulmonary Medicine, Fukushima Medical University, Fukushima, Japan; 1098 Kansai Electric Power Hospital, Osaka, Japan; 1099 Kumamoto City Hospital, Kumamoto, Japan; 1100 Department of Emergency and Critical Care Medicine, Tokyo Metropolitan Police Hospital, Tokyo, Japan; 1101 Department of Respiratory Medicine, International University of Health and Welfare, Shioya Hospital, Narita, Japan; 1102 Department of Clinical Laboratory, International University of Health and Welfare, Shioya Hospital, Narita, Japan; 1103 National Hospital Organization Saitama Hospital, Saitama, Japan; 1104 Department of Respiratory Medicine, Gunma University Graduate School of Medicine, Maebashi, Japan; 1105 Department of Orthopedic Surgery, Tokyo Medical University, Ibaraki Medical Center, Tokyo, Japan; 1106 Department of Internal Medicine, Kiryu Kosei General Hospital, Kiryu, Japan; 1107 Daini Osaka Police Hospital, Osaka, Japan; 1109 Department of Epidemiology, University Medical Centre Groningen, University of Groningen, Groningen, The Netherlands; 1112 Department of Psychiatry, University Medical Center Groningen, Groningen, The Netherlands; 1113 Department of Genetics, University Medical Center Groningen, Groningen, The Netherlands; 1114 Centre for Heart Lung Innovation, University of British Columbia, Vancouver, British Columbia, Canada; 1115 Division of Respiratory Medicine, Faculty of Medicine, University of British Columbia, Vancouver, British Columbia, Canada; 1116 Institut Universitaire de Cardiologie et de Pneumologie de Québec, Université Laval, Quebec, Quebec, Canada; 1117 Department of Genetics and Genomic Sciences, Icahn School of Medicine at Mount Sinai, New York, NY USA; 1118 University of Washington, Global Health, Seattle, WA USA; 1119 Gossamer Bio, San Diego, CA USA; 1120 Department of Pathology and Medical Biology, University Medical Centre Groningen, University of Groningen, Groningen, The Netherlands; 1121 GRIAC Research Institute, University Medical Centre Groningen, University of Groningen, Groningen, The Netherlands; 1122 Department of Pulmonary Diseases, University Medical Centre Groningen, University of Groningen, Groningen, The Netherlands; 1123 Center for Genomic Medicine, Massachusetts General Hospital, Boston, MA USA; 1124 Harvard Medical School, Cambridge, MA USA; 1125 Program in Medical and Population Genetics, Broad Institute, Boston, MA USA; 1126 Channing Division of Network Medicine, Department of Medicine, Brigham and Women’s Hospital, Boston, MA USA; 1127 Brigham and Women’s Hospital, Boston, MA USA; 1128 Psychiatric and Neurodevelopmental Genetics Unit, Center for Genomic Medicine, Massachusetts General Hospital, Boston, MA USA; 1129 Department of Neurology, Massachusetts General Hospital, Boston, MA USA; 1130 Division of General Internal Medicine, Massachusetts General Hospital and Department of Medicine, Boston, MA USA; 1132 Department of Human Genetics, University of Michigan, Ann Arbor, MI USA; 1133 Mount Sinai Clinical Intelligence Center, Department of Genetics and Genomic Sciences, Icahn School of Medicine at Mount Sinai, New York, NY USA; 1135 Sema4, a Mount Sinai venture, Stamford, CT USA; 1137 Mount Sinai Clinical Intelligence Center, Charles Bronfman Institute for Personalized Medicine, New York, NY USA; 1139 Department of Human Genetics, David Geffen School of Medicine at UCLA, Los Angeles, CA USA; 1140 Icahn Institute of Data Science and Genomics Technology, Icahn School of Medicine, New York, NY USA; 1141 Mount Sinai Clinical Intelligence Center, Icahn School of Medicine, New York, NY USA; 1142 Department of Genetic and Genomic Sciences, Icahn School of Medicine at Mount Sinai, New York, NY USA; 1143 Charles Bronfman Institute for Personalized Medicine, Icahn School of Medicine at Mount Sinai, New York, NY USA; 1144 Institute for Genomic Health, Icahn School of Medicine at Mount Sinai, New York, NY USA; 1146 The Mindich Child Health and Development Institute, Icahn School of Medicine at Mount Sinai, New York, NY USA; 1147 Pamela Sklar Division of Psychiatric Genomics, Icahn School of Medicine at Mount Sinai, New York, NY USA; 1148 Department of Psychiatry, Icahn School of Medicine at Mount Sinai, New York, NY USA; 1149 Icahn School of Medicine at Mount Sinai, New York, NY USA; 1150 Department of Psychiatry, Department of Genetic and Genomic Sciences, Icahn School of Medicine at Mount Sinai, New York, NY USA; 1151 Department of Environmental Medicine and Public Health, Icahn School of Medicine at Mount Sinai, New York, NY USA; 1152 Department of Human Genetics, Center for Autism Research and Treatment, Institute for Precision Health, University of California Los Angeles, Los Angeles, CA USA; 1153 The Hasso Plattner Institute of Digital Health at Mount Sinai, Icahn School of Medicine at Mount Sinai, New York, NY USA; 1154 BioMe Phenomics Center, >Icahn School of Medicine at Mount Sinai, New York, NY USA; 1155 Department of Medicine, Icahn School of Medicine at Mount Sinai, New York, NY USA; 1157 Regeneron Genetics Center, Tarrytown, NY USA; 1158 Phenomic Analytics & Clinical Data Core, Geisinger Health System, Danville, PA USA; 1159 Department of Population Health Sciences, Geisinger Health System, Danville, PA USA; 1160 Department of Molecular and Functional Genomics, Geisinger Health System, Danville, PA USA; 1162 Department of Genetics, University of Pennsylvania Perelman School of Medicine, Philadelphia, PA USA; 1163 Department of Biomedical Data Science, Stanford University, Stanford, CA USA; 1166 Department of Psychiatry, University of North Carolina at Chapel Hill, Chapel Hill, USA; 1167 Department of Nutrition, University of North Carolina at Chapel Hill, Chapel Hill, USA; 1168 Institute of Neuroscience and Physiology, University of Gothenburg, Gothenburg, Sweden; 1169 Department of Medical Sciences, University of Turin, Turin, Italy; 1170 Department of Clinical and Biological Sciences, University of Turin, Orbassano, Italy; 1171 Department of Pediatrics, Department of Microbiology, Immunology and Molecular Genetics, University of California Los Angeles, Los Angeles, CA USA; 1172 University of Genova, Genova, Italy; 1173 Hopital Mont-Godinne, Yvoir, Belgium; 1174 Department of Molecular Medicine, University of Pavia, Pavia, Italy; 1175 Department of Public Health and Pediatric Sciences, University of Turin, Turin, Italy; 1176 Qatar Biobank for Medical Research, Qatar Foundation Research, Development and Innovation, Qatar Foundation, Doha, Qatar; 1177 Latvian Biomedical Research and Study Centre, Riga, Latvia; 1178 Department of Neuroscience, Karolinska Institutet, Stockholm, Sweden; 1179 Max Planck Institute for Evolutionary Anthropology, Leipzig, Germany; 1180 Anaesthesiology and Intensive Care Medicine, Department of Surgical Sciences, Uppsala University, Uppsala, Sweden; 1181 Integrative Physiology, Department of Medical Cell Biology, Uppsala University, Uppsala, Sweden; 1182 Hedenstierna Laboratory, CIRRUS, Anaesthesiology and Intensive Care Medicine, Department of Surgical Sciences, Uppsala University, Uppsala, Sweden; 1183 Department of Computer Science, School of Engineering, University of California Los Angeles, Los Angeles, CA USA; 1184 University of California Los Angeles, Los Angeles, CA USA; 1185 Department of Psychiatry and Biobehavioral Sciences, David Geffen School of Medicine at University of California Los Angeles, Los Angeles, CA USA; 1186 Division of Immunology, Allergy, and Rheumatology, University of California Los Angeles, Los Angeles, CA USA; 1187 Department of Psychiatry, University of California Los Angeles, Los Angeles, CA USA; 1188 Department of Neurology, University of California Los Angeles, Los Angeles, CA USA; 1189 Department of Computational Medicine, University of California Los Angeles, Los Angeles, CA USA; 1190 Department of Pathology and Laboratory Medicine, University of California Los Angeles, Los Angeles, CA USA; 1191 Bioinformatics IDP, UCLA, Los Angeles, CA USA; 1192 Department of Neurology, David Geffen School of Medicine at UCLA, Los Angeles, CA USA; 1193 Department of Urology, David Geffen School of Medicine at UCLA, Los Angeles, CA USA; 1195 Queen Mary University, London, UK; 1196 UCL Great Ormond Street Institute of Child Health, London, UK; 1197 University of Cambridge, Cambridge, UK; 1199 Big Data Institute, Nuffield Department of Population Health, Li Ka Shing Centre for Health Information and Discovery, University of Oxford, Oxford, UK; 1201 Experimental Medicine Division, Nuffield Department of Medicine, John Radcliffe Hospital, University of Oxford, Oxford, UK; 1202 Public Health England, Field Service, Addenbrooke’s Hospital, Cambridge, UK; 1203 Public Health England, Data and Analytical Services, National Infection Service, London, UK; 1204 Program in Bioinformatics and Integrative Genomics, Harvard Medical School, Boston, MA USA; 1205 Program in Biological and Biomedical Sciences, Harvard Medical School, Boston, MA USA; 1207 Department of Clinical Research and Leadership, George Washington University, Washington, DC USA; 1208 Department of Human Genetics, The Wellcome Sanger Institute, Wellcome Genome Campus, Hinxton, Cambridge, UK; 1209 Strangeways Research Laboratory, The National Institute for Health Research Blood and Transplant Unit in Donor Health and Genomics, University of Cambridge, Cambridge, UK; 1210 Department of Haematology, University of Cambridge, Cambridge Biomedical Campus, Cambridge, UK; 1211 British Heart Foundation Cardiovascular Epidemiology Unit, Department of Public Health and Primary Care, University of Cambridge, Cambridge, UK; 1212 British Heart Foundation Centre of Research Excellence, University of Cambridge, Cambridge, UK; 1213 The National Institute for Health Research Blood and Transplant Research Unit in Donor Health and Genomics, University of Cambridge, Cambridge, UK; 1214 Health Data Research UK Cambridge, Wellcome Genome Campus and University of Cambridge, Cambridge, UK; 1215 Department of Human Genetics, Wellcome Sanger Institute, Hinxton, UK; 1216 Department of Epidemiology, Emory University Rollins School of Public Health, North Druid Hills, GA USA; 1217 Atlanta CA Health Care System, North Druid Hills, GA USA; 1218 Center for Population Genomics, MAVERIC, VA Boston Healthcare System, Boston, MA USA; 1219 MAVERIC, VA Boston Healthcare System, Boston, MA USA; 1220 Stanford University, Stanford, CA USA; 1221 Palo Alto VA Healthcare System, Stanford, CA USA; 1222 Department of Biostatistics, Boston University School of Public Health, Boston, MA USA; 1223 Department of Haematology, Central Hospital of Bolzano (SABES-ASDAA), Bolzano, Italy; 1224 Laboratory of Clinical Pathology, Hospital of Bressanone (SABES-ASDAA), Bressanone, Italy; 1226 University of Alcalá, Centro de Investigación Biomédica en Red en Enfermedades Respiratorias (CIBERES), Madrid, Spain; 1227 Center for Applied Genomics, The Children’s Hospital of Philadelphia, Philadelphia, PA USA; 1228 Division of Human Genetics, Department of Pediatrics, The Perelman School of Medicine, University of Pennsylvania, Philadelphia, PA USA; 1229 Faculty of Medicine, University of Iceland, Reykjavik, Iceland; 1231 Infectious Disease Unit, Hospital of Massa, Massa, Italy; 1232 Department of Clinical Medicine, Public Health, Life and Environment Sciences, University of L’Aquila, L’Aquila, Italy; 1233 UOSD Laboratorio di Genetica Medica - ASL Viterbo, San Lorenzo, Italy; 1234 Unit of Infectious Diseases, S. M. Annunziata Hospital, Florence, Italy; 1235 Infectious Disease Unit, Hospital of Lucca, Lucca, Italy; 1236 Department of Clinical and Experimental Medicine, Infectious Diseases Unit, University of Pisa, Pisa, Italy; 1238 Clinic of Infectious Diseases, Catholic University of the Sacred Heart, Rome, Italy; 1239 Department of Diagnostic and Laboratory Medicine, Institute of Biochemistry and Clinical Biochemistry, Fondazione Policlinico Universitario A. Gemelli IRCCS, Catholic University of the Sacred Heart, Rome, Italy; 1240 Private University in the Principality of Liechtenstein, Triesen, Liechtenstein; 1241 Digestive Diseases Unit, Virgen del Rocio University Hospital, Institute of Biomedicine of Seville, University of Seville, Seville, Spain; 1242 Department of Biochemistry, University Hospital Vall d’Hebron, Barcelona, Spain; 1243 University of Sevilla, Sevilla, Spain; 1244 Instituto de Biomedicina de Sevilla, Sevilla, Spain; 1245 Hospital Universitario Virgen del Rocío de Sevilla, Sevilla, Spain; 1246 Consejo Superior de Investigaciones científicas, Madrid, Spain; 1247 Humanitas Clinical and Research Center, IRCCS, Milan, Italy; 1248 Immunohematology Department, Banc de Sang i Teixits, Autonomous University of Barcelona, Barcelona, Spain; 1249 August Pi i Sunyer Biomedical Research Institute, Hospital Clinic, University of Barcelona, Barcelona, Spain; 1250 Department of Pathophysiology and Transplantation, Università degli Studi di Milano, Milan, Italy; 1251 Internal Medicine Department, Virgen del Rocio University Hospital, Sevilla, Spain; 1252 Department of Biomedical Sciences, Humanitas University, Milan, Italy; 1253 Department Emergency, Anesthesia and Intensive Care, University Milano-Bicocca, Monza, Italy; 1254 Department of Medical Sciences, Università degli Studi di Torino, Turin, Italy; 1255 Department of Medical Microbiology, Clinic of Laboratory Medicine, St Olav’s Hospital, Trondheim, Norway; 1256 Department of Infectious Diseases, St Olav’s Hospital, Trondheim University Hospital, Trondheim, Norway; 1257 Department of Clinical and Molecular Medicine, NTNU, Trondheim, Norway; 1258 Department of Research, St Olav’s Hospital, Trondheim University Hospital, Trondheim, Norway; 1259 Institute of Parasitology and Biomedicine Lopez-Neyra, Granada, Spain; 1260 Institute for Cardiogenetics, University of Lübeck, Lübeck, Germany; 1261 German Research Center for Cardiovascular Research, partner site Hamburg-Lübeck-Kiel, Lübeck, Germany; 1262 University Heart Center Lübeck, Lübeck, Germany; 1263 Department of Research, Ostfold Hospital Trust, Gralum, Norway; 1264 Pediatric Departement, Centro Tettamanti- European Reference Network (ERN) PaedCan, EuroBloodNet, MetabERN-University of Milano-Bicocca-Fondazione MBBM/Ospedale San Gerardo, Milan, Italy; 1265 Geminicenter for Sepsis Research, Institute of Circulation and Medical Imaging (ISB), NTNU, Trondheim, Norway; 1266 Clinic of Anesthesia and Intensive Care, St Olav’s Hospital, Trondheim University Hospital, Trondheim, Norway; 1267 Clinic of Medicine and Rehabilitation, Levanger Hospital, Nord-Trondelag Hospital Trust, Levanger, Norway; 1268 Stefan-Morsch-Stiftung, Birkenfeld, Germany; 1269 Center of Bioinformatics, Biostatistics, and Bioimaging, School of Medicine and Surgery, University of Milano Bicocca, Milan, Italy; 1270 Phase 1 Research Centre, ASST Monza, School of Medicine and Surgery, University of Milano-Bicocca, Milan, Italy; 1271 Pneumologia ASST-Monza, University of Milano-Bicocca, Milano, Italy; 1272 School of Medicine and Surgery, University of Milano-Bicocca, Milano, Italy; 1273 Infectious Diseases Unit, San Gerardo Hospital, Monza, Italy; 1274 SODIR-VHIR research group, Barcelona, Spain; 1275 Bioinformatics area, Fiundación progreso y Salud, Andalucia, Spain; 1276 Present Address: Program in Metabolism, Broad Institute of MIT and Harvard, Cambridge, MA USA; 1277 Present Address: Program in Medical and Population Genetics, Broad Institute of MIT and Harvard, Cambridge, MA USA; 1278 Present Address: Diabetes Unit, Center for Genomic Medicine, Massachusetts General Hospital, Boston, MA USA; 1279 Present Address: Harvard Medical School, Boston, MA USA

**Keywords:** Genetics, Genome-wide association studies, SARS-CoV-2, Viral infection

## Abstract

The genetic make-up of an individual contributes to the susceptibility and response to viral infection. Although environmental, clinical and social factors have a role in the chance of exposure to SARS-CoV-2 and the severity of COVID-19^1,2^, host genetics may also be important. Identifying host-specific genetic factors may reveal biological mechanisms of therapeutic relevance and clarify causal relationships of modifiable environmental risk factors for SARS-CoV-2 infection and outcomes. We formed a global network of researchers to investigate the role of human genetics in SARS-CoV-2 infection and COVID-19 severity. Here we describe the results of three genome-wide association meta-analyses that consist of up to 49,562 patients with COVID-19 from 46 studies across 19 countries. We report 13 genome-wide significant loci that are associated with SARS-CoV-2 infection or severe manifestations of COVID-19. Several of these loci correspond to previously documented associations to lung or autoimmune and inflammatory diseases^3–7^. They also represent potentially actionable mechanisms in response to infection. Mendelian randomization analyses support a causal role for smoking and body-mass index for severe COVID-19 although not for type II diabetes. The identification of novel host genetic factors associated with COVID-19 was made possible by the community of human genetics researchers coming together to prioritize the sharing of data, results, resources and analytical frameworks. This working model of international collaboration underscores what is possible for future genetic discoveries in emerging pandemics, or indeed for any complex human disease.

## Abstract (web summary)

A global network of researchers was formed to investigate the role of human genetics in SARS-CoV-2 infection and COVID-19 severity; this paper reports 13 genome-wide significant loci and potentially actionable mechanisms in response to infection.

## Main

The COVID-19 pandemic, caused by infection with SARS-CoV-2, has resulted in an enormous health and economic burden worldwide. One of the most remarkable features of SARS-CoV-2 infection is the variation in consequences, which range from asymptomatic to life-threatening, viral pneumonia and acute respiratory distress syndrome^8^. Although established host factors correlate with disease severity (for example, increasing age, being a man and higher body-mass index^1^), these risk factors alone do not explain all of the variability in disease severity observed across individuals.

Genetic factors contributing to COVID-19 susceptibility and severity may provide new biological insights into disease pathogenesis and identify mechanistic targets for therapeutic development or drug repurposing, as treating the disease remains a highly important goal despite the recent development of vaccines. Further supporting this line of inquiry, rare loss-of-function variants in genes involved in the type I interferon response may be involved in severe forms of COVID-19^9–11^. At the same time, several genome-wide association studies that investigate the contribution of common genetic variation^12–15^ to COVID-19 have provided robust support for the involvement of several genomic loci associated with COVID-19 severity and susceptibility, with the strongest and most robust finding for severity being at the 3p21.31 locus^12–16^. However, much remains unknown about the genetic basis of susceptibility to SARS-CoV-2 and severity of COVID-19.

**Fig. 1:**
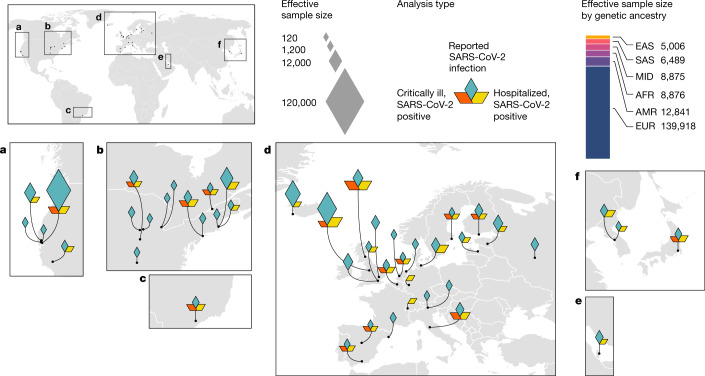
Geographical overview of the contributing studies to the COVID-19 HGI and composition by major ancestry groups. Populations are defined as African (AFR), admixed American (AMR), East Asian (EAS), European (EUR), Middle Eastern (MID) and South Asian (SAS).

The COVID-19 Host Genetics Initiative (COVID-19 HGI) (https://www.covid19hg.org/)^17^ is an international, open-science collaboration to share scientific methods and resources with research groups across the world with the goal to robustly map the host genetic determinants of SARS-CoV-2 infection and the severity of the resulting COVID-19 disease. Here, we report the latest results of meta-analyses of 46 studies from 19 countries (Fig. 1) for COVID-19 host genetic effects.

## Meta-analyses of COVID-19

**Extended Data Fig. 1:**
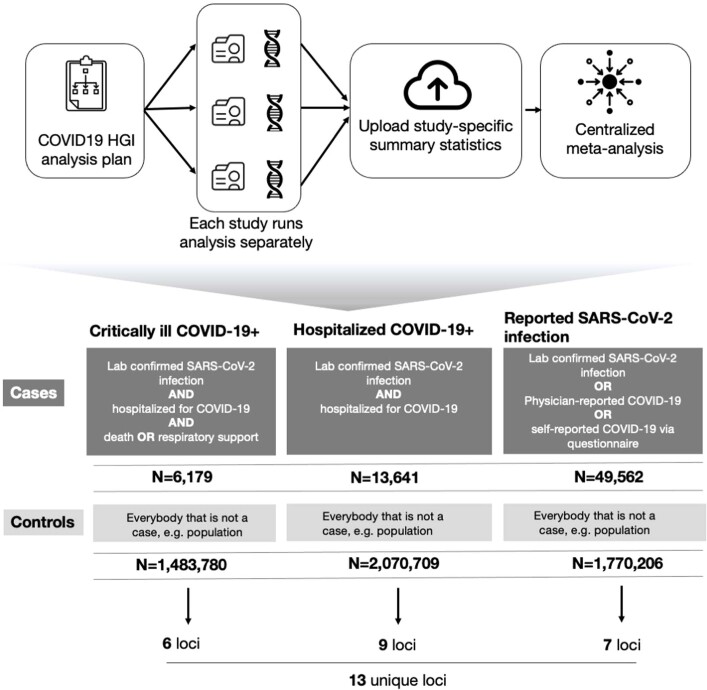
Analytical summary of the COVID-19 HGI meta-analysis. Using the analytical plan set by the COVID-19 HGI, each individual study runs their analyses and uploads the results to the Initiative, who then runs the meta-analysis. There are three main analyses that each study can contribute summary statistics to: critically ill COVID-19, hospitalized COVID-19 and reported SARS-CoV-2 infection. The phenotypic criteria used to define cases are listed in the dark grey boxes, along with the numbers of cases (*N*) included in the final all-ancestries meta-analysis. Controls were defined in the same way across all three analyses as everybody that is not a case—for example, population controls (light grey box). Sensitivity analyses—not reported in this extended data figure—also included mild and/or asymptomatic cases of COVID-19 as control individuals. Sample number (*N*) of control individuals differed between the analyses due to the difference in the number of studies contributing data to these.

**Extended Data Fig. 2:**
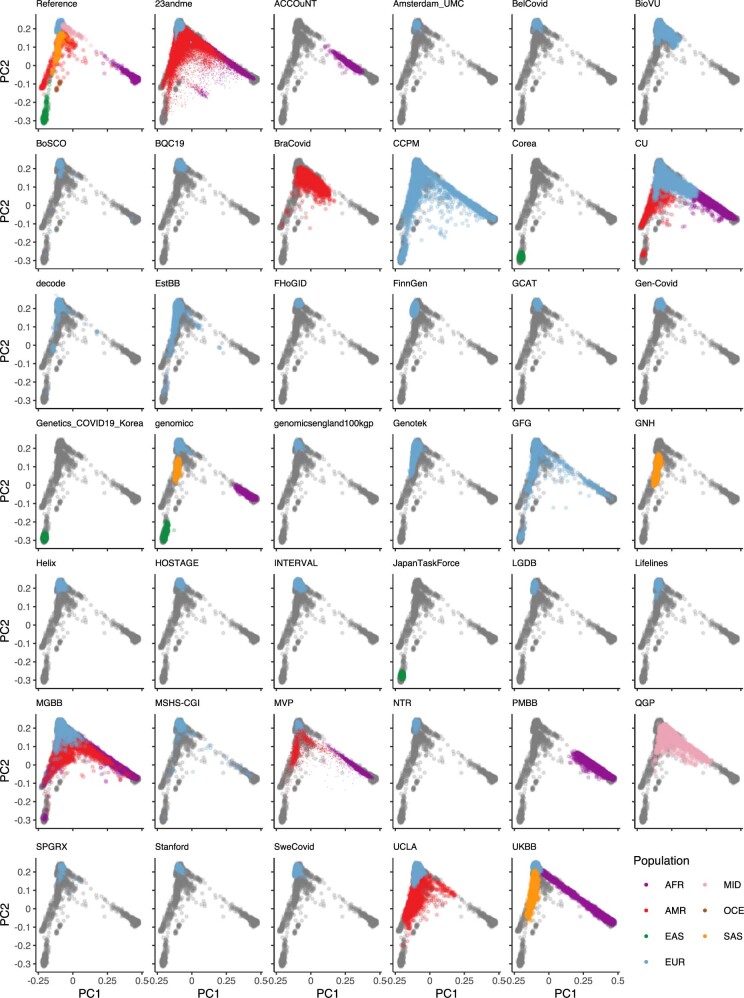
Projection of contributing studies samples into the same PC space. We asked participating studies to perform a PC projection using the 1000 Genomes Project and Human Genome Diversity Project as a reference, with a common set of variants. For each panel (except for the reference), coloured points correspond to contributed samples from each cohort, whereas grey points correspond to the reference samples from the 1000 Genomes Project. Colour represents a genetic population that each cohort specified. As 23andMe, Genomics England 100,000 Genomes Project (GenomicsEngland100kgp), and Million Veterans Program (MVP) only submitted PCA images, we overlaid their submitted transparent images using the same coordinates, instead of directly plotting them. Populations are defined as African (AFR), admixed American (AMR), East Asian (EAS), European (EUR), Middle Eastern (MID) and South Asian (SAS), Oceanian (OCE).

Overall, the COVID-19 HGI combined genetic data from 49,562 cases and 2 million controls across 46 distinct studies (Fig. 1). The data included studies from populations of different genetic ancestries, including European, admixed American, African, Middle Eastern, South Asian and East Asian individuals (Supplementary Table 1). An overview of the study design is provided in Extended Data Fig. 1. We performed case–control meta-analyses in three main categories of COVID-19 disease according to predefined and partially overlapping phenotypic criteria. These included (1) critically ill cases of COVID-19 defined as those individuals who required respiratory support in hospital or who died due to the disease; (2) cases of moderate or severe COVID-19 defined as those participants who were hospitalized due to symptoms associated with the infection; and (3) all cases with reported SARS-CoV-2 infection regardless of symptoms (Methods). Controls for all three analyses were selected as genetically ancestry-matched samples without known SARS-CoV-2 infection, if that information was available (Methods). The average age of the participants with COVID-19 across studies was 55 years (Supplementary Table 1). We report quantile–quantile plots in Supplementary Fig. 1 and ancestry principal component plots for contributing studies in Extended Data Fig. 2.

**Fig. 2:**
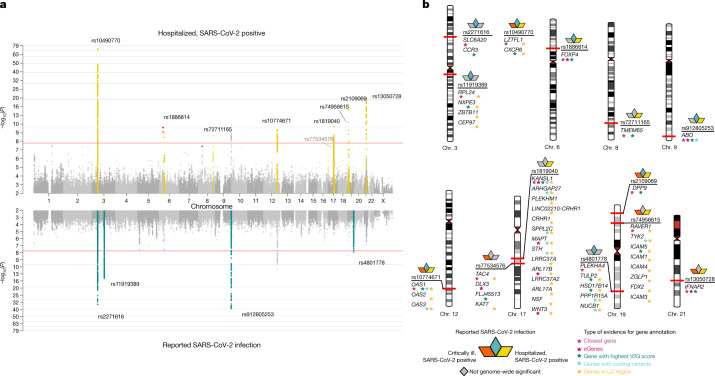
Genome-wide association results for COVID-19. **a**, Top, results of a genome-wide association study of hospitalized cases of COVID-19 (*n* = 13,641 cases and *n* = 2,070,709 controls). Bottom, the results of reported SARS-CoV-2 infections (*n* = 49,562 cases and *n* = 1,770,206 controls). Loci highlighted in yellow (top) represent regions associated with the severity of the COVID-19 manifestation—that is, increased odds of more severe COVID-19 phenotypes. Loci highlighted in green (bottom) are regions associated with susceptibility to a SARS-CoV-2 infection—that is, the effect is the same across mild and severe COVID-19 phenotypes. We highlight in red genome-wide significant variants that had high heterogeneity across contributing studies and that were therefore excluded from the list of loci found. **b**, Results of gene prioritization using different evidence measures of gene annotation. Genes in the LD region, genes with coding variants and eGenes (fine-mapped *cis*-eQTL variant PIP > 0.1 in GTEx Lung) are annotated if in LD with a COVID-19 lead variant (*r*^2^ > 0.6). V2G, highest gene prioritized by the V2G score of Open Target Genetics.

**Extended Data Fig. 3:**
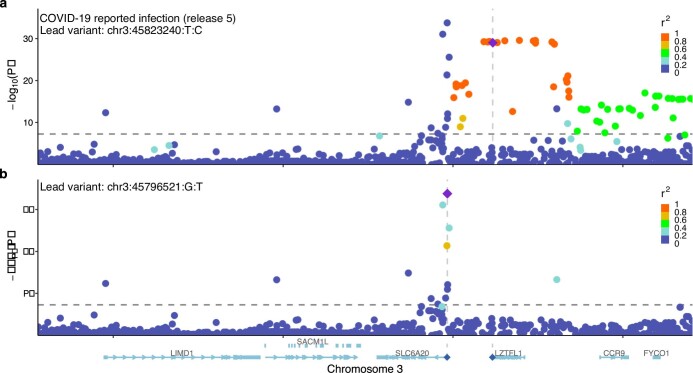
Locus-zoom plots of the 3p21.31 region for reported SARS-CoV-2 infection. **a**, A standard plot without exclusion. Here, the severity lead variant rs10490770 (chr. 3: 45823240T:C) is shown as a lead variant. **b**, Additional independent susceptibility signal(s) after excluding variants with *r*^*2*^ > 0.05 with rs10490770. The susceptibility lead variant rs2271616 (chr. 3: 45796521G:T) is highlighted.

**Extended Data Fig. 4:**
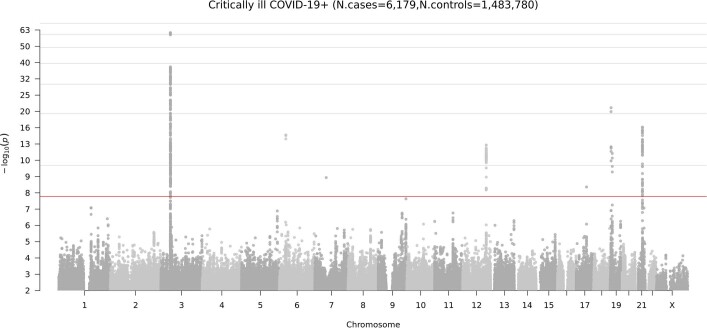
Genome-wide meta-analysis association results for critical illness due to COVID-19. The locus on chromosome 6 is the HLA locus, which was removed from the list of reported loci in Supplementary Table 2 due to the high heterogeneity in effect size estimated between studies included in the analysis. The locus on chromosome 7 was also not reported in Supplementary Table 2 due to missingness across studies—that is, the high number of studies in the meta-analysis that did not report summary statistics for this region. There are two association peaks on chromosome 19.

Across our three analyses, we reported a total of 13 independent genome-wide significant loci associated with COVID-19 (the threshold of *P* < 1.67 × 10^−8^ is adjusted for multiple trait testing) (Supplementary Table 2), most of which were shared between two or more COVID-19 phenotypes. Two of these loci are in very close proximity within the 3p21.31 region, which was previously reported as a single locus associated with COVID-19 severity^12–16^ (Extended Data Fig. 3). Overall, we find six genome-wide significant associations for critical illness due to COVID-19, using data from 6,179 cases and 1,483,780 controls from 16 studies (Extended Data Fig. 4). Nine genome-wide significant loci were detected for moderate to severe hospitalized COVID-19 (including five of the six critical illness loci) from an analysis of 13,641 cases of COVID-19 and 2,070,709 controls across 29 studies (Fig. 2a, top). Finally, seven loci reached genome-wide significance in the analysis using data for all available 49,562 reported cases of SARS-CoV-2 infection and 1,770,206 controls, using data from a total of 44 studies (Fig. 2a, bottom). The proportion of cases with non-European genetic ancestry for each of the three analyses was 23%, 29% and 22%. We report the results for the lead variants at the 13 loci in different ancestry-group meta-analyses in Supplementary Table 3. We note that two loci, tagged by lead variants rs1886814 and rs72711165, had higher allele frequencies in southeast Asian (rs1886814; 15%) and East Asian genetic ancestry (rs72711165; 8%) whereas the minor allele frequencies in European populations were less than 3%. This highlights the value of including data from diverse populations for genetic discovery. We discuss the replication of previous findings and the new discoveries from these three analyses in the Supplementary Note.

## Variant effects on severity and susceptibility

**Extended Data Fig. 5:**
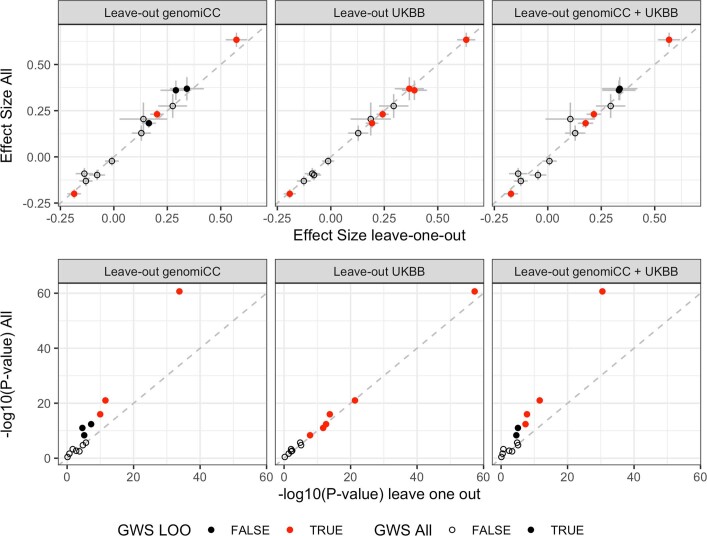
Sensitivity analyses for overlapping controls in genomiCC and UK Biobank. Comparison of the beta effect sizes (top) and unadjusted *P* values (bottom) of the 13 lead variants, using data from the COVID-19 critical illness meta-analysis in all the cohorts to leaving out genomiCC (cases, *n* = 4,354; controls, *n* = 1,474,655; total, *n* = 1,479,009), leaving out the UK Biobank (UKBB; cases, *n* = 5,870; controls, *n* = 1,155,203; total, *n* = 1,161,073) and leaving out both genomiCC and UK Biobank (cases, *n* = 4,045; controls, *n* = 1,146,078; total, *n* = 1,150,123) (from left to right, respectively). Top, dots and grey bars represent the beta effect size estimates ± standard error from the corresponding GWAS meta-analysis. Bottom, dots represent two-sided *P* values from the corresponding GWAS meta-analysis. Filled dots indicate variants that showed genome-wide significance in the full meta-analysis of critical illness due to COVID-19, and empty dots represent variants that were not significant for critical illness but were significant for either hospitalization due to COVID-19 or reported SARS-CoV-2 infection. Red dots represent variants that showed genome-wide significance in the leave-one-out analysis for genomiCC, UK Biobank or genomiCC and UK Biobank.

We found no genome-wide significant sex-specific effects at the 13 loci. However, we did identify significant heterogeneous effects (*P* < 0.004) across studies for 3 out of the 13 loci (Methods), which probably reflects the differential ascertainment of cases (Supplementary Table 2). There was a small number of overlapping samples (*n* = 8,380 European ancestry; *n* = 745 East Asian ancestry) between controls from the genOMICC and the UK Biobank studies, but leave-one-out sensitivity analyses did not reveal any bias in the corresponding effect sizes or *P* values (Extended Data Fig. 5 and Supplementary Information).

We next wanted to better understand whether the 13 significant loci were acting through mechanisms that increased the susceptibility to infection or that affected the progression of symptoms towards more severe disease. For all 13 loci, we compared the lead variant (strongest association *P* value) odds ratios (ORs) for the risk-increasing allele across our different COVID-19 phenotype definitions.

**Extended Data Fig. 6:**
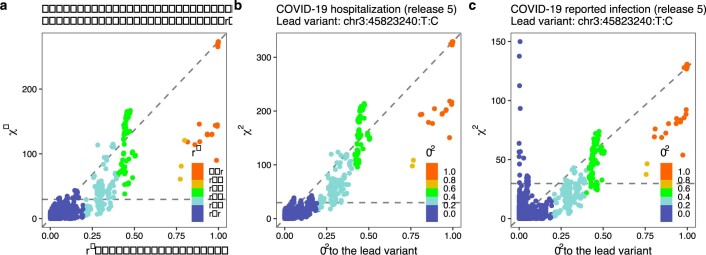
Comparison of *χ*^2^ statistics and *r*^*2*^ values to the lead variant in the 3p21.31 region. **a**–**c**, Data are shown for critical illness (**a**), hospitalization (**b**) and reported SARS-CoV-2 infection (**c**). The left blue peak in **c**, which is uncorrelated with the lead variants in the region, indicates that there are independent signals.

Focusing on the two better powered analyses: all cases with a reported SARS-CoV-2 infection and all cases hospitalized due to COVID-19, we find that four of the loci have similar odds ratios between these two analyses (Methods and Supplementary Table 2). Such consistency suggests a stronger link to susceptibility to SARS-CoV-2 infection rather than to the development of severe COVID-19. The strongest susceptibility signal was the previously reported *ABO* locus (rs912805253)^12,13,15,16^. Notably, and in agreement with a previously reported study^15^, we also report a locus within the 3p21.31 region that was more strongly associated with susceptibility to SARS-CoV-2 than progression to more severe COVID-19 phenotypes. rs2271616 showed a stronger association with a reported SARS-CoV-2 infection (*P* = 1.79 × 10^−34^; OR (95% confidence interval (CI)) = 1.15 (1.13–1.18)) than hospitalization (*P* = 1.05 × 10^−5^; OR (95% CI) = 1.12 (1.06–1.19)). For this locus—which contains additional independent signals—the linkage-disequilibrium (LD) pattern is discordant with the *P*-value expectation (Extended Data Fig. 6 and Supplementary Note), pointing to a key missing causal variant or to a potentially undiscovered multi-allelic or structural variant in this locus.

**Extended Data Fig. 7:**
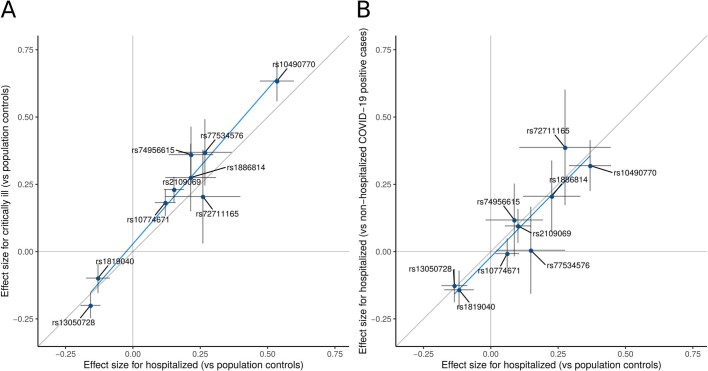
Comparison of the effect sizes of lead variants between pairs of COVID-19 meta-analyses. Comparison of effect sizes for the nine variants associated with severity of COVID-19 disease. **a**, Comparing hospitalized cases of COVID-19 versus population controls (*n* = 10,428 cases and *n* = 1,483,270 controls) and critically ill cases of COVID-19 versus population controls (*n* = 6,179 cases and *n* = 1,483,780 controls). **b**, Hospitalized cases of COVID-19 versus population controls (*n* = 5,806 cases and *n* = 1,144,263 controls) and hospitalized cases of COVID-19 versus non-hospitalized cases of COVID-19 (*n* = 5,773 cases and *n* = 15,497 controls). Sample sizes for hospitalized cases of COVID-19 versus population controls differ between **a** and **b** due to differences in the sampling of studies selected for the analysis. This selection included all studies that were able to contribute data to the respective analyses that the data were compared to (shown on the *y* axis) in each panel. Dots represent the effect size beta estimates, bars represent the 95% confidence interval of the estimates. Effect size estimates and *P* values for heterogeneity tests (Cochran’s *Q*, two-tailed test) are reported in Supplementary Table 3.

By contrast, 9 out of the 13 loci were associated with increased risk of severe symptoms with significantly larger odds ratios for hospitalized COVID-19 compared with the mildest phenotype of reported SARS-CoV-2 infection (eight loci were below the threshold of *P* < 0.004 (test for effect size difference) and, in addition, the lead variant rs10774671 had a clear increase in odds ratios despite not passing this threshold) (Supplementary Table 2). We further compared the odds ratios for these nine loci for critical illness due to COVID-19 versus hospitalized due to COVID-19, and found that these loci exhibited a general increase in effect risk for critical illness (Methods, Extended Data Fig. 7a and Supplementary Table 4), but the lower power for association analysis of critically ill COVID-19 means that these results should be considered as suggestive. Overall, these results indicated that these nine loci were more likely to be associated with progression of the disease and worse outcome from SARS-CoV-2 infection compared to being associated with susceptibility to SARS-CoV-2 infection.

For some of these analyses, the controls were simply existing population controls without knowledge of SARS-CoV-2 infection or COVID-19 status, which may bias effect size estimates as some of these individuals may have either become infected with SARS-CoV-2 or developed COVID-19. We perform several sensitivity analyses (Extended Data Fig. 7b, Supplementary Note and Supplementary Table 4) in which we show that using population controls can be a valid and powerful strategy for host genetic discovery of infectious disease, and particularly those that are widespread and with rare severe outcomes.

## Gene prioritization and association with other traits

**Extended Data Fig. 8:**
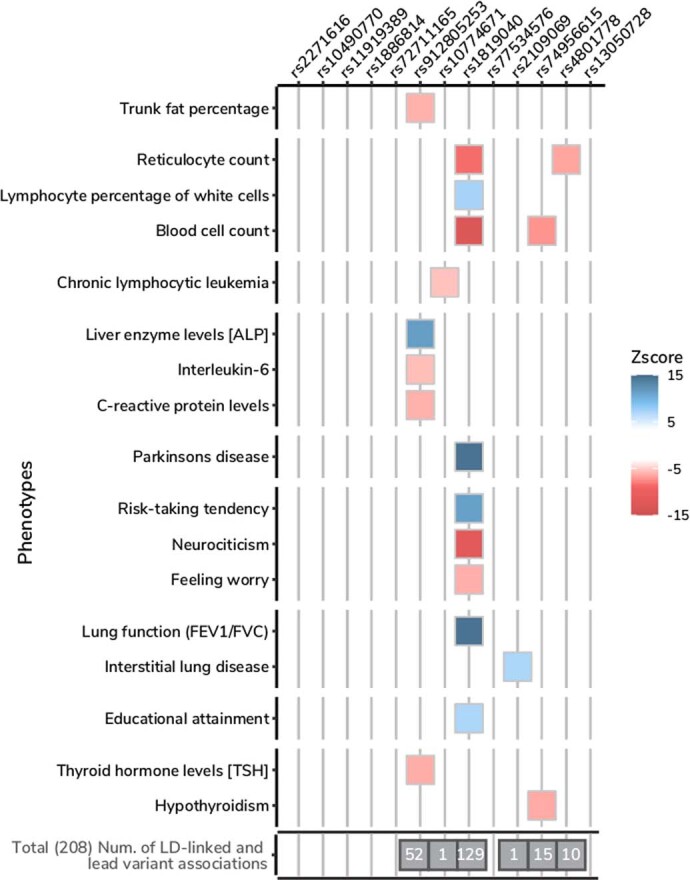
PheWAS for genome-wide significant lead variants. Selected phenotypes associated with genome-wide significant COVID-19 variants (see Supplementary Table 6 for a complete list). We report those associations for which a lead variant from a previous GWAS result was in high LD (*r*^2^ > 0.8) with the index COVID-19 variants. The colour represents the *z*-scores of correlated risk increasing alleles for the trait. The total number of associations for each COVID-19 variant is highlighted in the grey box.

To better understand the potential biological mechanism of each locus, we applied several approaches to prioritize candidate causal genes and explore additional associations with other diseases and traits. Of the 13 genome-wide significant loci, we found that nine loci implicated biologically plausible genes (Supplementary Tables 2, 5). Protein-altering variants in LD with lead variants implicated genes at six loci, including *TYK2* (chromosome and cytogenetic band (chr.) 19p13.2) and *PPP1R15A* (chr. 19q13.33). The COVID-19 lead variant rs74956615T>A in *TYK2*, which confers risk for critical illness (OR (95% CI) = 1.43 (1.29–1.59), *P* = 9.71 × 10^−12^) and hospitalization due to COVID-19 (OR (95% CI) = 1.27 (1.18–1.36), *P* = 5.05 × 10^−10^) is correlated with the missense variant rs34536443:G>C (p.Pro1104Ala; *r*^2^ = 0.82). This is consistent with the primary immunodeficiency described with complete *TYK2* loss of function^3^ as this variant is known to reduce function^18,19^. By contrast, this missense variant was previously reported to be protective against autoimmune diseases (Extended Data Fig. 8 and Supplementary Table 6), including rheumatoid arthritis (OR = 0.74, *P* = 3.0 × 10^−8^; UK Biobank SAIGE) and hypothyroidism (OR = 0.84, *P* = 1.8 × 10^−10^; UK Biobank). At the 19q13.33 locus, the lead variant rs4801778, which was significantly associated with a reported SARS-CoV-2 infection (OR (95% CI) = 0.95 (0.93–0.96), *P* = 2.1 × 10^−8^), is in LD (*r*^2^ = 0.93) with a missense variant rs11541192:G>A (p.Gly312Ser) in *PPP1R15A*.

A lung-specific *cis*-expression quantitative trait loci (*cis*-eQTLs) from GTEx v.8^20^ (*n* = 515) and the Lung eQTL Consortium^21^ (*n* = 1,103) provided further support for a subset of loci (Supplementary Table 7), including *FOXP4* (chr. 6p21.1) and *ABO* (chr. 9q34.2), *OAS1*/*OAS3*/*OAS2* (chr. 12q24.13) and *IFNAR2/IL10RB* (21q22.11), where the COVID-19-associated variants modify gene expression in lung. Furthermore, our phenome-wide association study (PheWAS) analysis (Supplementary Table 6) implicated three additional loci related to lung function, with modest lung eQTL evidence—that is, the lead variant was not fine-mapped but significantly associated. An intronic variant rs2109069:G>A in *DPP9* (chr. 19p13.3), which is positively associated with critical illness, was previously reported to be risk-increasing for interstitial lung disease (tag lead variant rs12610495:A>G (p.Leu8Pro); OR = 1.29, *P* = 2.0 × 10^−12^)^5^. The COVID-19 lead variant rs1886814:A>C in the *FOXP4* locus is correlated (*r*^2^ = 0.64) with a lead variant of lung adenocarcinoma (tag variant is rs7741164; OR = 1.2, *P* = 6.0 × 10^−13^)^6,22^ and similarly with a lead variant reported for subclinical interstitial lung disease^23^. In severe COVID-19, lung cancer and interstitial lung disease, the minor, expression-increasing allele is associated with increased risk. We also found that intronic variants (chr. 1q22) and rs1819040:T>A in *KANSL1* (chr. 17q21.31), associated with protection against hospitalization due to COVID-19, were previously reported for reduced lung function (for example, tag lead variant rs141942982:G>T; OR (95% CI) = 0.96 (0.95–0.97), *P* = 1.00 × 10^−20^)^7^. Notably, the 17q21.31 locus is a well-known locus for structural variants containing a megabase inversion polymorphism (H1 and inverted H2 forms) and complex copy-number variations, in which the inverted H2 forms were shown to be positively selected in European individuals^24,25^.

Lastly, there are two loci in the 3p21.31 region with varying genes prioritized by different methods for different independent signals. For the severity lead variant rs10490770:T>C, we prioritized *CXCR6* with the Variant2Gene (V2G) algorithm^26^, although *LZTFL1* is the closest gene. The *CXCR6* has a role in chemokine signalling^27^ and *LZTFL1* has been implicated in lung cancer^28^. rs2271616:G>T, which is associated with susceptibility, tags a complex region including several independent signals (Supplementary Note) that are all located within the gene body of *SLC6A20*, which encodes a protein that is known to functionally interact with the SARS-CoV-2 receptor ACE2^29^. However, none of the lead variants in the 3p21.31 region has been previously associated with other traits or diseases in our PheWAS analysis. Although these results provide supporting in silico evidence for candidate causal gene prioritization, further functional characterization is needed. Detailed locus descriptions and LocusZoom plots are provided in Supplementary Fig. 2.

## Polygenic architecture of COVID-19

To further investigate the genetic architecture of COVID-19, we used results from meta-analyses including samples from European ancestries (sample sizes are described in the Methods and Supplementary Table 1) to estimate the heritability explained by common single-nucleotide polymorphisms—that is, the proportion of variation in the two phenotypes that was attributable to common genetic variants—and to determine whether heritability of COVID-19 phenotypes was enriched in genes that were specifically expressed in certain tissues^30^ from the GTEx dataset^31^. We detected low, but significant, heritability across all three analyses (<1% on observed scale, all *P* values were *P* < 0.0001) (Supplementary Table 8). The values are low compared to previously published studies^14^, but may be explained by differences in the reported estimate scale (observed versus liability), the specific method used, disease-prevalence estimates, phenotypic differences between patient cohorts or ascertainment of controls. Despite the low reported values, we found that heritability of a reported SARS-CoV-2 infection was significantly enriched in genes that were specifically expressed in the lung (*P* = 5.0 × 10^−4^) (Supplementary Table 9). These findings, together with the genome-wide significant loci identified in the meta-analyses, suggest that there is a significant polygenic architecture that can be better leveraged with future, larger, sample sizes.

## Genetic correlation and Mendelian randomization

Genetic correlations (*r*_g_) between the three COVID-19 phenotypes was high, although lower correlations were observed between hospitalized COVID-19 and reported SARS-CoV-2 infection (critical illness versus hospitalized: *r*_g_ (95% CI) = 1.37 (1.08–1.65), *P* = 2.9 × 10^−21^; critical illness versus reported SARS-CoV-2 infection, *r*_g_ (95% CI) = 0.96 (0.71–1.20), *P* = 1.1 × 10^−14^; hospitalized versus reported SARS-CoV-2 infection: *r*_g_ (95% CI) = 0.85 (0.68–1.02), *P* = 1.1 × 10^−22^). To better understand which traits are genetically correlated and/or potentially causally associated with COVID-19 hospitalization, critical illness and reported SARS-CoV-2 infection, we chose a set of 38 disease, health and neuropsychiatric phenotypes as potential COVID-19 risk factors based on their clinical correlation with disease susceptibility, severity or mortality (Supplementary Table 10).

**Fig. 3:**
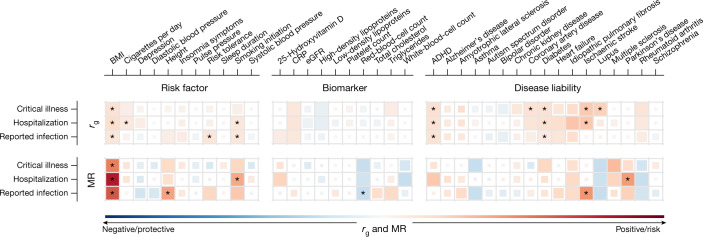
Genetic correlations and Mendelian randomization causal estimates between 38 traits and COVID-19 critical illness, hospitalization and reported SARS-CoV-2 infection. Larger squares correspond to *P* values with higher significance, with genetic correlations (*r*_g_) or Mendelian randomization (MR) causal estimates significantly different from zero. The size of each coloured square indicates the magnitude of the *P* value, with *P* < 0.05 shown as a full-sized square, *P* = 0.05–0.1 as a large square, *P* = 0.1–0.5 as a medium square and *P* > 0.5 as a small square. Genetic correlations or causal estimates that are significantly different from zero at an FDR of 5% are marked with an asterisk. Two-sided *P* values were calculated using LDSC for genetic correlations and inverse-variance-weighted analysis for Mendelian randomization. ADHD, attention-deficit hyperactivity disorder; BMI, body mass index; CRP, C-reactive protein; eGFR, estimated glomerular filtration rate.

**Extended Data Fig. 9:**
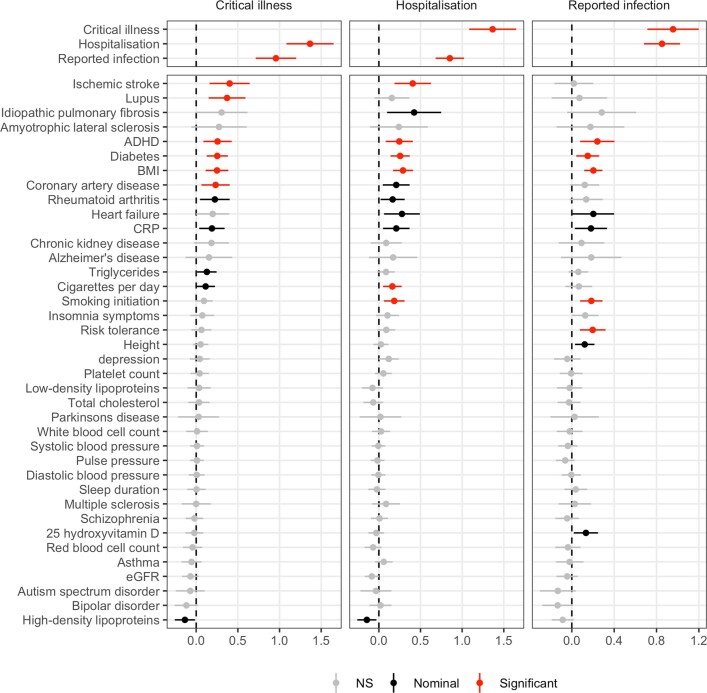
Genetic correlation with COVID-19 phenotypes. Each column shows the genetic correlation results for the three COVID-19 phenotypes (European-ancestry analyses only): critical illness, hospitalization and reported SARS-CoV-2 infection. The traits that the genetic correlation is run against are listed on the left. Significant correlations (FDR < 0.05) are shown with their 95% confidence intervals in red, nominally significant correlations (*P* < 0.05) are in black and non-significant correlations are in grey. Two-sided *P* values were calculated using LDSC for genetic correlations and exact estimates, unadjusted standard errors and two-sided *P* values are available in Supplementary Table 11.

We found evidence (false-discovery rate (FDR) < 0.05) of significant genetic correlations between nine traits and hospitalized COVID-19 and reported SARS-CoV-2 infection (Fig. 3, Extended Data Fig. 9 and Supplementary Table 11). Notably, genetic liability to ischaemic stroke was only significantly positively correlated with critical illness or hospitalization due to COVID-19, but not with a higher likelihood of reported SARS-CoV-2 infection (infection *r*_g_ = 0.019 versus hospitalization *r*_g_ = 0.41, *z* = 2.7, *P* = 0.006; infection *r*_g_ = 0.019 versus critical illness *r*_g_ = 0.40, *z* = 2.49, *P* = 0.013).

**Extended Data Fig. 10:**
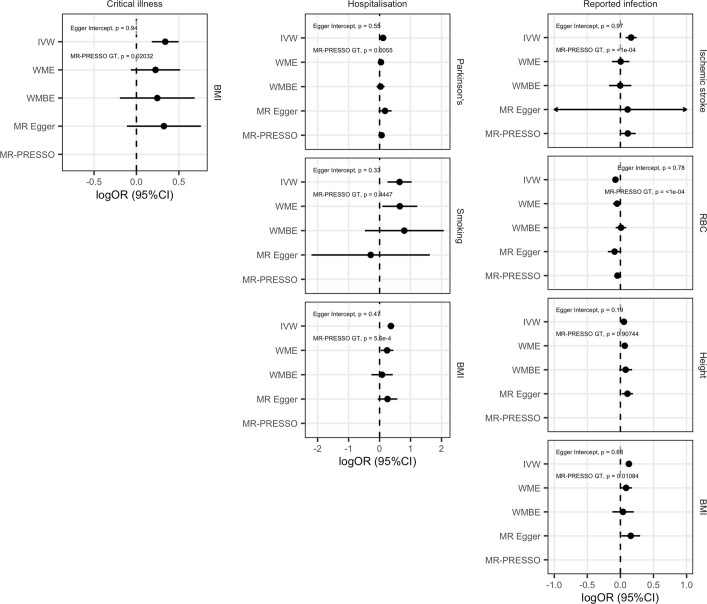
Mendelian randomization sensitivity analyses. Genetic correlations and Forest plots displaying the causal estimates for each of the sensitivity analyses used in the Mendelian randomization analysis for trait pairs that were significant at an FDR of 5%. Two-sided *P* values were estimated using IVW, WME, WMBE and MR-PRESSO analyses. RBC, red blood cell count.

We next used two-sample Mendelian randomization to infer potentially causal relationships between these traits. After correcting for multiple testing (FDR < 0.05), eight exposure–COVID-19 trait pairs showed suggestive evidence of a causal association (Fig. 3, Extended Data Fig. 10, Supplementary Table 12 and Supplementary Fig. 3). Five of these associations were robust to potential violations of the underlying assumptions of Mendelian randomization. Corroborating our genetic correlation results and evidence from epidemiological studies, genetically predicted higher body-mass index (OR (95% CI) = 1.4 (1.3–1.6), *P* = 8.5 × 10^−11^) and smoking (OR (95% CI) = 1.9 (1.3–2.8), *P* = 0.0012) were associated with increased risk of COVID-19 hospitalization, with body-mass index also being associated with increased risk of SARS-CoV-2 infection (OR (95% CI) = 1.1 (1.1–1.2), *P* = 4.8 × 10^−7^). Genetically predicted increased height (OR (95% CI) = 1.1 (1–1.1)), *P* = 8.9 × 10^−4^) was associated with an increased risk of reported SARS-CoV-2 infection, whereas a genetically predicted higher red-blood-cell count (OR (95% CI) = 0.93 (0.89–0.96), *P* = 5.7 × 10^−5^) was associated with a reduced risk of reported SARS-CoV-2 infection. Despite evidence of a genetic correlation between type II diabetes and COVID-19 outcomes, there was no evidence of a causal association in the Mendelian randomization analyses, which suggests that the observed genetic correlations are due to pleiotropic effects between body-mass index and type 2 diabetes. Further sensitivity analyses relating to sample overlap are discussed in the Supplementary Information.

## Discussion

The COVID-19 HGI has brought together investigators from across the world to advance genetic discovery for SARS-CoV-2 infection and severe COVID-19 disease. We report 13 genome-wide significant loci associated with some aspect of SARS-CoV-2 infection or COVID-19. Many of these loci overlap with previously reported associations with lung-related phenotypes or autoimmune or inflammatory diseases, but some loci have no obvious candidate gene.

Four out of the thirteen genome-wide significant loci showed similar effects in the reported SARS-CoV-2 infection analysis (a proxy for disease susceptibility) and all-hospitalized COVID-19 (a proxy for disease severity). Of these, one locus was in close proximity to, yet independent of, the major genetic signal for COVID-19 severity at the 3p21.31 locus. Notably, this locus was associated with COVID-19 susceptibility rather than severity. The locus overlaps *SLC6A20*, which encodes an amino acid transporter that interacts with ACE2. Nonetheless, we caution that more data are needed to resolve the nature of the relationship between genetic variation and COVID-19 at this locus, particularly as the physical proximity, LD structure and patterns of association suggest that untagged genetic variation could drive the association signal in the region. Our findings support the notion that some genetic variants, most notably at the *ABO* and *PPP1R15A* loci, in addition to *SLC6A20*, can indeed affect susceptibility to infection rather than progression to severe COVID-19 once infected.

Several of the loci reported here—as noted in previous publications^12,14^—intersect with well-known genetic variants that have established genetic associations. Examples of these include variants at *DPP9* and *FOXP4*, which show previous evidence of increasing risk for interstitial lung disease^5^, and missense variants within *TYK2* that show a protective effect on several autoimmune-related diseases^32–35^. Together with the heritability enrichment observed in genes expressed in lung tissues, these results highlight the involvement of lung-related biological pathways in the development of severe COVID-19. Several other loci show no previously documented genome-wide significant associations, despite the high significance and attractive candidate genes for COVID-19 (for example, *CXCR6*, *LZTFL1*, *IFNAR2* and *OAS1/OAS2/OAS3* loci). The previously reported associations for the strongest association for COVID-19 severity at the 3p21.31 locus and monocytes count are likely to be due to proximity and not a true co-localization.

Increasing the global representation in genetic studies enhances the ability to detect novel associations. Two of the loci that affect disease severity were only discovered by including the four studies of individuals with East Asian ancestry. One of these loci—close to *FOXP4*—is common particularly in East Asian participants (32%) as well as admixed American participants in the Americas (20%) and Middle Eastern participants (7%), but has a low frequency in most European ancestries (2–3%) in our data. Although we cannot be certain of the mechanism of action, the *FOXP4* association is an attractive biological target, as it is expressed in the proximal and distal airway epithelium^36^ and has been shown to have a role in controlling epithelial cell fate during lung development^37^. The COVID-19 HGI continues to pursue expansion of the datasets included in the analyses of the consortium to populations from underrepresented populations in upcoming data releases. We plan to release ancestry-specific results in full once the sample sizes allow for a well-powered meta-analysis.

Care should be taken when interpreting the results from a meta-analysis because of challenges with case and control ascertainment and collider bias (see Supplementary Note for a more detailed discussion on study limitations). Drawing a comprehensive and reproducible map of the host genetics factors associated with COVID-19 severity and SARS-CoV-2 requires a sustained international effort to include diverse ancestries and study designs. To accelerate downstream research and therapeutic discovery, the COVID-19 HGI regularly publishes meta-analysis results from periodic data freezes on the website https://www.covid19hg.org/ and provides an interactive explorer through which researchers can browse the results and the genomic loci in more detail. Future work will be required to better understand the biological and clinical value of these findings. Continued efforts to collect more samples and detailed phenotypic data should be endorsed globally, allowing for more thorough investigation of variable, heritable symptoms, particularly in light of the newly emerging strains of SARS-CoV-2, which may provoke different host responses that lead to disease.

## Methods

### Contributing studies

All of the participants were recruited following protocols approved by local Institutional Review Boards; this information is collected in Supplementary Table 1 for all 46 studies. All protocols followed local ethics recommendations and informed consent was obtained when required. Information about sample numbers, sex and age from for each contributing study is given in Supplementary Table 1. In total, 16 studies contributed data to the analysis of critical illness due to COVID-19, 29 studies contributed data to hospitalized COVID-19 analysis and 44 studies contributed to the analysis of all cases of COVID-19. Each individual study that contributed data to a particular analysis met a minimum threshold of 50 cases, as defined by the phenotypic criteria, for statistical robustness. The effective sample sizes for each ancestry group shown in Fig. 1 were calculated for display using the formula: ((4 × *N*_case_ × *N*_control_)/(*N*_case_ + *N*_control_)). Details of contributing research groups are provided in Supplementary Table 1.

### Phenotype definitions

COVID-19 disease status (critical illness and hospitalization status) was assessed following the Diagnosis and Treatment Protocol for Novel Coronavirus Pneumonia^38^. The critically ill COVID-19 group included patients who were hospitalized owing to symptoms associated with laboratory-confirmed SARS-CoV-2 infection and who required respiratory support or whose cause of death was associated with COVID-19. The hospitalized COVID-19 group included patients who were hospitalized owing to symptoms associated with laboratory-confirmed SARS-CoV-2 infection.

The reported SARS-CoV-2 infection group included individuals with laboratory-confirmed SARS-CoV-2 infection or electronic health record, ICD coding or clinically confirmed COVID-19, or self-reported COVID-19 (for example, by questionnaire), with or without symptoms of any severity. Genetic-ancestry-matched control individuals for the three case definitions were sourced from population-based cohorts, including individuals whose exposure status to SARS-CoV-2 was either unknown or infection-negative for questionnaire/electronic-health-record-based cohorts. Additional information regarding individual studies contributing to the consortium are described in Supplementary Table 1.

### Genome-wide association studies and meta-analyses

Each contributing study genotyped the samples and performed quality controls, data imputation and analysis independently, but following the consortium recommendations (information is available at https://www.covid19hg.org/). We recommended that genome-wide association study (GWAS) analyses were run using Scalable and Accurate Implementation of GEneralized mixed model (SAIGE)^39^ on chromosomes 1–22 and X. The recommended analysis tool was SAIGE, but studies also used other software such as PLINK^40^. The suggested covariates were age, age^2^, sex, age × sex and the 20 first principal components. Any other study-specific covariates to account for known technical artefacts could be added. SAIGE automatically accounts for sample relatedness and case–control imbalances. Quality-control and analysis approaches for individual studies are reported in Supplementary Table 1.

Study-specific summary statistics were then processed for meta-analysis. Potential false positives, inflation and deflation were examined for each submitted GWAS. Allele frequency plots against gnomAD 3.0 genomes were manually inspected for each study. Standard error values as a function of the effective sample size were used to find studies that deviated from the expected trend. Summary statistics passing this manual quality control were included in the meta-analysis. Variants with an allele frequency of >0.1% and an imputation INFO score of >0.6 were carried forward from each study. Variants and alleles were lifted over to genome build GRCh38, if needed, and harmonized to gnomAD 3.0 genomes^41^ by finding matching variants by strand flipping or switching the ordering of alleles. If multiple matching variants were included, the best match was chosen according to the minimum fold change in absolute allele frequency. Meta-analysis was performed using the inverse-variance-weighted (IVW) method on variants that were present in at least two-thirds of the studies contributing to the phenotype analysis. The method summarizes effect sizes across the multiple studies by computing the mean of the effect sizes weighted by the inverse variance in each individual study.

We report 13 meta-analysis variants that pass the genome-wide significance threshold after adjusting the threshold for multiple traits tested (*P* < 5 × 10^−8^/3). We report the unadjusted *P* values for each variant. We tested for heterogeneity between estimates from contributing studies using Cochran’s *Q*-test^42,43^. This is calculated for each variant as the weighted sum of squared differences between the effects sizes and their meta-analysis effect, the weights being the inverse variance of the effect size. *Q* is distributed as a *χ*^2^ statistic with *k* (number of studies) minus one degrees of freedom. Two loci reached genome-wide significance but were excluded from the significant results in Supplementary Table 2 due to heterogeneity between estimates from contributing studies and missingness between studies at chr. 6: 31057940–31380334 and chr. 7: 54671568–54759789; however, these regions are not excluded from the corresponding summary statistics in data release 5 (COVID-19 HGI (https://www.covid19hg.org/results/r5/) and GWAS Catalog (study code GCST011074)). For each of the lead variants reported in Supplementary Table 2, we aimed to find loci specific to susceptibility or severity by testing whether there was heterogeneity between the effect sizes associated with hospitalized COVID-19 (progression to severe disease) and reported SARS-CoV-2 infection. We used the Cochran’s *Q* measure^42,43^, calculated for each variant as the weighted sum of squared differences between the two analysis effect sizes and their meta-analysis effect with the weights being the inverse variance of the effect size. A significant *P* value of *P* < 0.004 ((0.05/13 loci) for multiple tests) indicates that the effect sizes for a particular variant are significantly different in the two analyses (Supplementary Table 2). For the nine loci, in which the lead variant effect size was significantly higher for hospitalized COVID-19, we carried out the same test again but comparing effect sizes from hospitalized COVID-19 with critically ill COVID-19 (Supplementary Table 4). Furthermore, we carried out the same test comparing meta-analysed hospitalized COVID-19 (population as controls) and hospitalized COVID-19 (SARS-CoV-2-positive but non-hospitalized as controls) (Supplementary Table 4). For these pairs of phenotype comparisons, we generated new meta-analysis summary statistics to use; including only those studies that could contribute data to both phenotypes that were under comparison.

### Principal component projection

To project every GWAS participant into the same principal component (PC) space, we used pre-computed PC loadings and reference allele frequencies. For reference, we used unrelated samples from the 1000 Genomes Project and the Human Genome Diversity Project and computed PC loadings and allele frequencies for the 117,221 single-nucleotide polymorphisms (SNPs) that (1) are available in every cohort; (2) have a minor allele frequency of >0.1% in the reference; and (3) are LD-pruned (*r*^2^ < 0.8; 500-kb window). We then asked each cohort to project their samples using our automated script provided at https://github.com/covid19-hg/. It internally uses the PLINK2^44^ --score function with the variance-standardize option and reference allele frequencies (--read-freq); so that each cohort-specific genotype/dosage matrix is mean-centred and variance-standardized with respect to reference allele frequencies, but not cohort-specific allele frequencies. We further normalized the projected PC scores by dividing the values by a square root of the number of variants used for projection to account for a subtle difference due to missing variants.

### Gene prioritization

To prioritize candidate causal genes reported in full in Supplementary Table 2, we used various gene prioritization approaches using both locus-based and similarity-based methods. Because we only describe the in silico gene prioritization results without characterizing the actual functional activity in vitro or in vivo, we aimed to provide a systematic approach to nominate potential causal genes in a locus using the following criteria.

(1) The closest gene: a gene that is closest to a lead variant by distance to the gene body.

(2) Genes in the LD region: genes that overlap with a genomic range containing any variants in LD (*r*^*2*^ > 0.6) with a lead variant. For LD computation, we retrieved LD matrices provided by gnomAD v.2.1.1^41^ for each population analysed in this study (except for admixed American, Middle Eastern and South Asian genetic ancestry populations, for whom data are not available). We then constructed a weighted-average LD matrix by per-population sample sizes in each meta-analysis, which we used as a LD reference.

(3) Genes with coding variants: genes with at least one loss-of-function or missense variant (annotated by VEP^45^ v.95 with GENCODE v.29) that is in LD with a lead variant (*r*^2^ > 0.6).

(4) eGenes: genes with at least one fine-mapped *cis*-eQTL variant (PIP > 0.1) that is in LD with a lead variant (*r*^2^ > 0.6) (Supplementary Table 5). We retrieved fine-mapped variants from the GTEx v.8^20^ (https://www.finucanelab.org/) and eQTL catalogue^46^. In addition, we looked up significant associations in the Lung eQTL Consortium^21^ (*n* = 1,103) to further support our findings in lung with a larger sample size (Supplementary Table 7). We note that, in contrast to the GTEx or eQTL catalogue, we only looked at associations and did not fine-map our data to the Lung eQTL Consortium data.

(5) V2G: a gene with the highest overall V2G score based on Open Targets Genetics (OTG)^26^. For each variant, the overall V2G score aggregates differentially weighted evidence of variant–gene associations from several data sources, including molecular *cis*-QTL data (for example, *cis*-protein QTLs from ref. ^47^, *cis*-eQTLs from GTEx v.7 and so on), interaction-based datasets (for example, promoter capture Hi-C), genomic distance and variant effect predictions (VEP) from Ensembl. A detailed description of the evidence sources and weights used is provided in the OTG documentation (https://genetics-docs.opentargets.org/our-approach/data-pipeline)^26^.

### Phenome-wide association study

To investigate the evidence of shared effects of 15 index variants for COVID-19 and previously reported phenotypes, we performed a phenome-wide association study. We considered phenotypes in OTG obtained from the GWAS catalogue (this included studies with and without full summary statistics, *n* = 300 and 14,013, respectively)^48^ and from the UK Biobank. Summary statistics for UK Biobank traits were extracted from SAIGE^39^ for binary outcomes (*n* = 1,283 traits) and Neale v.2 (*n* = 2,139 traits) for both binary and quantitative traits (http://www.nealelab.is/uk-biobank/) and FinnGen Freeze 4 cohort (https://www.finngen.fi/en/access_results). We report PheWAS results for phenotypes for which the lead variants were in high LD (*r*^2^ > 0.8) with the 13 genome-wide significant lead variants from our main COVID-19 meta-analysis (Supplementary Table 6). This conservative approach allowed spurious signals primarily driven by proximity rather than actual colocalization to be removed (see Methods).

To remove plausible spurious associations, we retrieved phenotypes for GWAS lead variants that were in LD (*r*^2^ > 0.8) with COVID-19 index variants.

### Heritability

LD score regression v.1.0.1^49^ was used to estimate the SNP heritability of the phenotypes from the meta-analysis summary statistic files. As this method depends on matching the LD structure of the analysis sample to a reference panel, the summary statistics of European ancestry only were used. Sample sizes were *n* = 5,101 critically ill cases of COVID-19 and *n* = 1,383,241 control participants, *n* = 9,986 hospitalized cases of COVID-19 and *n* = 1,877,672 control participants, and *n* = 38,984 cases and *n* = 1,644,784 control participants for the analysis of all cases—all including the 23andMe cohort. Pre-calculated LD scores from the 1000 Genomes European reference population were obtained online (https://data.broadinstitute.org/alkesgroup/LDSCORE/). Analyses were conducted using the standard program settings for variant filtering (removal of non-HapMap3 SNPs, the HLA region on chromosome 6, non-autosomal, *χ*^2^ > 30, minor allele frequency of <1%, or allele mismatch with reference). We additionally report SNP heritability estimates for the all-ancestries meta-analyses, calculated using European panel LD scores, in Supplementary Table 8.

### Partitioned heritability

We used partitioned LD score regression^50^ to partition COVID-19 SNP heritability in cell types in our summary statistics for European ancestry only. We ran the analysis using the baseline model LD scores calculated for European populations and regression weights that are available online (https://github.com/bulik/ldsc). We used the COVID-19 summary statistics for European ancestry only for the analysis.

### Genome-wide association summary statistics

We obtained genome-wide association summary statistics for 43 complex-disease, neuropsychiatric, behavioural or biomarker phenotypes (Supplementary Table 10). These phenotypes were selected based on their putative relevance to COVID-19 susceptibility, severity or mortality, with 19 selected based on the Centers for Disease Control list of underlying medical conditions associated with COVID-19 severity^51^ or traits reported to be associated with increased risk of COVID-19 mortality by OpenSafely^52^. Summary statistics generated from GWAS using individuals of European ancestry were preferentially selected if available. These summary statistics were used in subsequent genetic correlation and Mendelian randomization analyses.

### Genetic correlation

LD score regression^50^ was also used to estimate the genetic correlations between our COVID-19 meta-analysis phenotypes reported using samples of only European ancestry, and between these and the curated set of 38 summary statistics. Genetic correlations were estimated using the same LD score regression settings as for heritability calculations. Differences between the observed genetic correlations of SARS-CoV-2 infection and COVID-19 severity were compared using a *z*-score method^53^.

### Mendelian randomization

Two-sample Mendelian randomization was used to evaluate the potential for causal association of the 38 traits on COVID-19 hospitalization, on COVID-19 severity and reported SARS-CoV-2 infection using samples of only European ancestry. Independent genome-wide significant SNPs robustly associated with the exposures of interest (*P* < 5 × 10^−8^) were selected as genetic instruments by performing LD clumping using PLINK^40^. We used a strict *r*^*2*^ threshold of 0.001, a 10-Mb clumping window, and the European reference panel from the 1000 Genomes Project^54^ to discard SNPs in LD with another variant with a smaller *P*-value association. For genetic variants that were not present in the hospitalized COVID-19 analysis, PLINK was used to identify proxy variants that were in LD (*r*^*2*^ > 0.8). Next, the exposure and outcome datasets were harmonized using the R package TwoSampleMR^55^. Namely, we ensured that the effect of a variant on the exposure and outcome corresponded to the same allele, we inferred positive-strand alleles and dropped palindromes with ambiguous allele frequencies, as well as incompatible alleles. Supplementary Table 10 includes the harmonized datasets used in the analyses.

The global test from Mendelian randomization pleiotropy residual sum and outlier (MR-PRESSO)^56^ software was used to investigate overall horizontal pleiotropy. In brief, the standard IVW meta-analytic framework was used to calculate the average causal effect by excluding each genetic variant used to instrument the analysis. A global statistic was calculated by summing the observed residual sum of squares, that is, the difference between the effect predicted by the IVW slope excluding the SNP, and the observed effect of the SNP on the outcome. Overall horizontal pleiotropy was subsequently analysed by comparing the observed residual sum of squares, with the residual sum of squares expected under the null hypothesis of no pleiotropy. The MR-PRESSO global test was shown to perform well when the outcome and exposure GWASs are not disjoint (although the power to detect horizontal pleiotropy is slightly reduced by complete sample overlap). We also used the regression intercept in MR-Egger^57^ to evaluate potential bias due to directional pleiotropic effects. This additional check was used in Mendelian randomization analyses with an ${I}_{{\rm{GX}}}^{2}$ index surpassing the recommended threshold (${I}_{{\rm{GX}}}^{2} > 90 \%$)^58^. Contingent on the MR-PRESSO global test results we analysed the causal effect of each exposure on COVID-19 hospitalization by using a fixed-effect IVW meta-analysis as the primary analysis, or, if pleiotropy was present, the MR-PRESSO outlier-corrected test. The IVW approach estimates the causal effect by aggregating the single-SNP causal effects (obtained using the ratio of coefficients method—that is, the ratio of the effect of the SNP on the outcome over the effect of the SNP on the exposure) in a fixed-effects meta-analysis. The SNPs were assigned weights based on their inverse variance. The IVW method confers the greatest statistical power for estimating causal associations^59^, but assumes that all variants are valid instruments and can produce biased estimates if the average pleiotropic effect differs from zero. Alternatively, when horizontal pleiotropy was present, we used the MR-PRESSO outlier-corrected method to correct the IVW test by removing outlier SNPs. We conducted further sensitivity analyses using alternative Mendelian randomization methods that provide consistent estimates of the causal effect even when some instrumental variables are invalid, at the cost of reduced statistical power including: (1) Weighted median estimator (WME); (2) weighted mode-based estimator (WMBE); and (3) MR-Egger regression. Robust causal estimates were defined as those that were significant at an FDR of 5% and either (1) showed no evidence of heterogeneity (MR-PRESSO global test *P* > 0.05) or horizontal pleiotropy (Egger intercept *P* > 0.05); or (2) in the presence of heterogeneity or horizontal pleiotropy, the WME-, WMBE-, MR-Egger- or MR-PRESSO-corrected estimates were significant (*P* < 0.05). All statistical analyses were conducted using R v.4.0.3. Mendelian randomization analysis was performed using the ‘TwoSampleMR’ v.0.5.5 package^55^.

### Website and data distribution

In anticipation of the need to coordinate many international partners around a single meta-analysis effort, we created the COVID-19 HGI website (https://covid19hg.org). We were able to centralize information, recruit partner studies, rapidly distribute summary statistics and present preliminary interpretations of the results to the public. Open meetings are held on a monthly basis to discuss future plans and new results; video recordings and supporting documents are shared (https://covid19hg.org/meeting-archive). This centralized resource provides a conceptual and technological framework for organizing global academic and industry groups around a shared goal. The website source code and additional technical details are available at https://github.com/covid19-hg/covid19hg.

To recruit new international partner studies, we developed a workflow in which new studies are registered and verified by a curation team (https://covid19hg.org/register). Users can explore the registered studies using a customized interface to find and contact studies with similar goals or approaches (https://covid19hg.org/partners). This helps to promote organic assembly around focused projects that are adjacent to the centralized effort (https://covid19hg.org/projects). Visitors can query study information, including study design and research questions. Registered studies are visualized on a world map and are searchable by institutional affiliation, city and country.

To encourage data sharing and other forms of participation, we created a rolling acknowledgements page (https://covid19hg.org/acknowledgements) and directions on how to contribute data to the central meta-analysis effort (https://covid19hg.org/data-sharing). Upon the completion of each data freeze, we post summary statistics, plots and sample size breakdowns for each phenotype and contributing cohort (https://covid19hg.org/results). The results can be explored using an interactive web browser (https://app.covid19hg.org). Several computational research groups carry out follow-up analyses, which are made available for download (https://covid19hg.org/in-silico). To enhance scientific communication to the public, preliminary results are described in blog posts by the scientific communications team and shared on Twitter. The first post was translated to 30 languages with the help of 85 volunteer translators. We compile publications and preprints submitted by participating groups and summarize genome-wide significant findings from these publications (https://covid19hg.org/publications).

### Reporting summary

Further information on research design is available in the Nature Research Reporting Summary linked to this paper.

## Online content

Any methods, additional references, Nature Research reporting summaries, source data, extended data, supplementary information, acknowledgements, peer review information; details of author contributions and competing interests; and statements of data and code availability are available at 10.1038/s41586-021-03767-x.

### Supplementary information

Supplementary Information This Supplementary Information file contains the following sections: New and replicated loci from COVID-19 HGI meta-analyses; Additional independent susceptibility signals at the 3p21.31 locus ; Sensitivity analysis for use of population controls; Sensitivity analysis for overlapping samples between cohorts in Mendelian randomization analyses; Supplementary discussion on study limitations; Supplementary References; and titles and summaries for Supplementary Tables 1-13 (see Excel file for Supplementary Tables).

Reporting Summary

Supplementary Figure 1 Quantile-quantile plots for GWAS from all individual studies that contributed data. QQ-plots showing the expected -log_10_(*P*-values) on the x-axis and the observed unadjusted *P*-values values from two-tailed inverse variance weighted meta-analysis on the y-axis (red line showing no deviation from the expected) for each study contributing data to the analyses. Sample size of cases and controls is listed for each study in the plot title, as well as the median lambda value.

Supplementary Figure 2 LozusZoom plots to visualise the meta-analysis results at the loci passing genome-wide significance. For each genome-wide significant locus in three meta-analyses: meta-analysis of critical illness, hospitalization, and reported infection, we showed 1) a manhattan plot of each locus where a color represents a weighted-average *r*^2^ value (see Methods) to a lead variant (unadjusted *P*-values from the two-tailed inverse variance weighted meta-analysis); 2) *r*^*2*^ values to a lead variant across gnomAD v2 populations, i.e., African/African-American (AFR), Latino/Admixed American (AMR), Ashkenazi Jewish (ASJ), East Asian (EAS), Estonian (EST), Finnish (FIN), Non-Finish Europeans (NFE), North-Western Europeans (NWE), and Southern Europeans (SEU); 3) genes at a locus; and 4) genes prioritized by each gene prioritization metric where a size of circles represents a rank in each metric. Note that the COVID-19 lead variants were chosen across all the meta-analyses (Supplementary Table 2; see Methods) and were not necessarily a variant with the most significant *P*-value from each inverse variance weighted meta-analysis.

Supplementary Figure 3 Scatter and funnel plots for each for exposure - COVID-19 outcome pair. Scatter plots show the exposure variant effect size against the COVID-19 outcome variant effect size and corresponding standard errors. Funnel plots show the Mendelian randomization (MR) causal estimates for each variant against their precision, with asymmetry in the plot indicating potential violations of the assumptions of MR. Regression lines show the corresponding causal estimates fixed effect inverse-weighted (IVW, red-solid line) meta-analysis; MR-Egger regression (blue-dashed); Weighted median estimator (WME, green-dashed); weighted mode based estimator (WMBE, purple-dashed); and Mendelian Randomization Pleiotropy RESidual Sum and Outlier corrected (MR-PRESSO, orange-dashed). Variants highlighted in red were flagged as outliers by MR-PRESSO.

Supplementary Tables This file contains Supplementary Tables 1-13; see main Supplementary Information PDF for table titles and summaries.

Supplementary Information This file contains the full authorship for the Covid-19 Host Genetics Initiative.

Peer Review File

## Data Availability

Summary statistics generated by the COVID-19 HGI are available at https://www.covid19hg.org/results/r5/ and are available in the GWAS Catalog (study code GCST011074). The analyses described here include the freeze-5 data. COVID-19 HGI continues to regularly release new data freezes. Summary statistics for non-European ancestry samples are not currently available due to the small individual sample sizes of these groups, but results for lead variants of 13 loci are reported in Supplementary Table 3. Individual level data can be requested directly from contributing studies, listed in Supplementary Table 1. We used publicly available data from GTEx (https://gtexportal.org/home/), the Neale lab (http://www.nealelab.is/uk-biobank/), Finucane lab (https://www.finucanelab.org), the FinnGen Freeze 4 cohort (https://www.finngen.fi/en/access_results) and the eQTL catalogue release 3 (http://www.ebi.ac.uk/eqtl/).

## Code availability

The code for summary statistics lift-over, the projection PCA pipeline including precomputed loadings and meta-analyses are available on GitHub (https://github.com/covid19-hg/) and the code for the Mendelian randomization and genetic correlation pipeline is available on GitHub at https://github.com/marcoralab/MRcovid.
