# Supplementary material for: Mapping the human genetic architecture of COVID-19
Source: Nature. 2021 Jul 8;600(7889):472–7. doi: 10.1038/s41586-021-03767-x (PMC8674144; doi:10.1038/s41586-021-03767-x)
Supplement: Supplementary file 1 — This Supplementary Information file contains the following sections: New and replicated loci from COVID-19 HGI meta-analyses; Additional independent susceptibility signals at the 3p21.31 locus ; Sensitivity analysis for use of population controls; Sensitivity analysis for overlapping samples between cohorts in Mendelian randomization analyses; Supplementary discussion on study limitations; Supplementary References; and titles and summaries for Supplementary Tables 1-13 (see Excel file for Supplementary Tables). [file 41586_2021_3767_MOESM1_ESM.pdf]

nature portfolio

https://doi.org/10.1038/s41586-021-03767-x

Supplementary information

Mapping the human genetic architecture of COVID-19

In the format provided by the authors and unedited

Supplementary Note

New and replicated loci from COVID-19 HGI meta-analyses 1

Additional independent susceptibility signals at the 3p21.31 locus 2

Sensitivity analysis for use of population controls 3

Sensitivity analysis for overlapping samples between cohorts in Mendelian randomization analyses 4

Supplementary discussion on study limitations 4

References 5

New and replicated loci from COVID-19 HGI meta-analyses

The current manuscript reports 13 genome-wide significant loci, that include many previously described loci but also new loci that have not previously been described. The COVID-19 Host Genetics Initiative (HGI) currently has the most comprehensive number of samples and studies included, and has also previously contributed to published work by collaborators. The data released used in manuscript (version 5) includes studies that have previously published their results and used earlier versions of the HGI data as replication. The studies included in the HGI, despite our best efforts to pre-define the phenotypes, also have various types of study designs (hospital collection, biobank, existing disease-cohorts) and include patients with different degrees of COVID-19 severity. Additionally, due to the inclusion of studies with variable sample sizes, individual loci found in our analyses can be biased towards being driven by a smaller number of more well-powered studies. As such, comparing the findings from previously reported studies to our meta-analysis of 46 studies has its limitations, but we attempt to describe how these findings reported in our manuscript have contributed to the replication and new knowledge of COVID-19 host genetics.

The first genome-wide association study reporting significant hits was published on a preprint site in June 2020 by the Severe Covid-19 GWAS Group [1], who had identified the 3p21.31 and *ABO* loci. The COVID-19 HGI contributed data for replication with the release 2 data freeze. The HGI release 2 results on their own were not genome-wide significant; likely due to the still relatively small numbers of cases accumulated in the studies involved (total *n*=1,678 all reported cases). However, the meta-analysis of HGI data with Ellinghaus et al. did provide supporting evidence for 3p21.31 locus. In July 2020, the COVID-19 HGI released its third data freeze which included data *n*=3,199 hospitalized cases and *n*=6,696 cases of reported infection; including the data from Severe Covid-19 GWAS Group. The findings in this publicly available dataset were stronger evidence for the 3p21.31 locus, but the ABO locus did not reach

significance in the severe case nor in the reported infection analysis. The next well powered analysis was published on a preprint site by the genOMICC study in September 2020 [2]. This study included cases enriched for severe phenotypes, and reported evidence for eight genome-wide significant loci. These included the chr3 region, *OAS* gene cluster, *DPP9*, *TYK2* and *IFNAR2* loci. The COVID-19 HGI provided replication for these from release 4 data, which was made available online to the public in October 2020. The *ABO* locus was not significant in the genOMICC study, but was significant in the release 4 of COVID-19 HGI for all three now established phenotypes: critical illness due to COVID-19, hospitalized due to COVID-19 and reported infection. In this manuscript we discuss how, by using HGI release 5 ([ADD release month, 2020) data, we show that the *ABO* is in fact a susceptibility locus. We note that the HLA-locus on chr6, reported also by genOMICC, was significant in the HGI release 4 and 5, but due to the high heterogeneity between estimates from studies contributing data to the HGI meta-analysis, we have excluded this locus from our main reported genome-wide significant hits; but the locus is still included in the release summary statistics. Interestingly, in October 2020 [3] reported that the 3p21.31 locus was more associated with susceptibility phenotypes rather than severe COVID-19, as had previously been reported by the aforementioned studies. In this manuscript, we report that the locus contains potentially more than one independent associations that affect either severity or susceptibility (more below). The three other associations reported in the paper are not replicated in the HGI data.

In the current HGI release 5 data, which we use for this manuscript, we also report loci that to our knowledge have not been previously described. These are loci in the regions of the *FOXP4*, *TMEM65*, *KANSL1*, *TAC4* genes and a locus on chr19 that currently has no genes prioritized by our pipeline. From these, particularly the *FOXP4* locus suggests appealing biological mechanisms discussed in the main manuscript text. Further work is needed to investigate the mechanism of any associations found through genome-wide studies, and the HGI is committed to providing the data and analysis to support these undertakings.

Additional independent susceptibility signals at the 3p21.31 locus

To identify loci that potentially have multiple causal variants, we compared marginal chi-squared association statistics and r^2 values to the lead variant for each locus. In theory, marginal chi-squared statistics should correlate with r^2 values to a lead variant (that is tagged by a single causal variant), and any deviation might be a sign of additional causal variants in a locus, or existence of missing causal variant(s) that are not properly tagged by a lead variant. We found the 3p21.31 locus has multiple independent signals only for reported infection (**Extended Data Figure 3**), and confirmed an additional susceptibility lead variant rs2271616:G>T is independent from the severity lead variant rs10490770:T>C ($r^2 = 0.0$).

Unfortunately, we found that a proper statistical fine-mapping or conditional analysis is extremely challenging for our current meta-analysis due to the concerns over inter-cohort heterogeneity (difference

in phenotyping, genotyping, and imputation, etc) and a lack of appropriate in-sample LD reference. Instead, we took a closer look at rs2271616:G>T and its proximal variants rs2271615:G>C (r^2 = 0.79 to rs2271616), rs73062389:G>A (r^2 = 0.34), and rs73062394:A>T (r^2 = 0.30); We observed a clear deviation from an expected relationship between their chi-squared statistics and r^2 values to rs2271616, suggesting that there might be missing causal variants or complex structural variants in this region. We note that this deviation holds true even when restricting to the studies that have all the four variants available.

Given the potential sign of missing causal variants in the region, we caution further disentangling the observed susceptibility association with the present result. Indeed, by using the latest release of UK Biobank (number of cases = 13,256 in European samples for reported infection; released Apr 9, 2021), we observed rs73062389:G>A showed more significant association (P =2.2 × 10^{-13}) than the current lead variant rs2271616:G>T (P =2.4 × 10^{-12}) (unpublished data), which re-emphasizes the crucial needs to collect more data to dissect this signal.

Sensitivity analysis for use of population controls

Using population controls for COVID-19 phenotypes has multiple challenges because the status of exposure to the SARS-CoV-2 virus and COVID-19 status are typically unknown for these samples. Additionally, the ascertainment of population controls may have been done for other purposes, and therefore the demographic of the population may not be a full match to the cases population, for e.g. age or sex distribution, or exact geographical location. The potential benefits of population control cohorts is that the data is often already available and for a large number of samples, increasing the power for association testing. We therefore perform sensitivity analyses (**Extended Data Figure 7B, Supplementary Table 4**) to better understand the effect of using such controls. We conducted a meta-analysis, where we restricted to studies that compared hospitalized COVID-19 cases with controls with laboratory-confirmed SARS-CoV-2 infection but who had mild symptoms or were asymptomatic (n = 5,773 cases and n = 15,497 controls) (**Methods**). We used two-tailed Cochran's Q-test (**Methods**) to compare variant effect sizes between this new analysis and our main analysis using population controls. We found that across the nine loci that had reached genome-wide significance in our main hospitalized COVID-19 analysis, the ORs were not significantly different in the analysis with better refined controls (**Extended Data Figure 7B, Supplementary Table 4**). This shows that the use of population controls for infectious disease host genetic studies is a valid approach.

Sensitivity analysis for overlapping samples between cohorts in meta-analysis of hospitalized COVID-19

We noted that there was sample overlap between some datasets contributing to our meta-analysis of hospitalized COVID-19, as a result of inclusion of samples from the UK Biobank. Specifically, this affected the genOMICC dataset: there was sample overlap of n = 8,380 EUR and n = 745 EAS between controls from the genOMICC and the UK Biobank studies**.** We therefore conducted a sensitivity analysis

comparing the effect size and *P*-values of the lead SNPs from summary statistics in which 1) genOMICC was excluded, 2) the UK Biobank was excluded and 3) genOMICC and the UK Biobank was excluded, to the full meta-analysis summary statistics (**Extended Data Figure 5)**. Effect sizes across all three sensitivity analyses were not significantly different, however the *P*-values were significantly attenuated when excluding genOMICC from the meta-analysis, but not when the UK Biobank was excluded. As such, we do not believe the minimal sample overlap in controls between the UK Biobank and genOMICC is biasing the observed results.

Sensitivity analysis for overlapping samples between cohorts in Mendelian randomization analyses

We noted that there was sample overlap between some datasets used to generate the exposures used in the primary MR previous analysis and COVID-19 hospitalization, on COVID-19 severity and SARS-CoV-2 reported infection, in particular due to the inclusion of samples from the UK Biobank. We therefore conducted an additional sensitivity analysis by conducting the MR analyses using COVID-19 hospitalization, on COVID-19 severity and SARS-CoV-2 reported infection summary statistics in which the UK Biobank study had been removed, (**Supplementary Table 12**). In this analysis, genetically predicted higher BMI was associated with an increased risk COVID-19 critical illness (OR[95%CI], 1.4 [1.2, 1.6], *P* = 0.0001), COVID-19 hospitalization (OR[95%CI], 1.3 [1.2, 1.5], *P* = 4.10E-05) and SARS-CoV-2 reported infection (OR[95%CI], 1.1 [1, 1.1], *P* = 0.018). Higher genetically predicted height was associated with increased risk of SARS-CoV-2 reported infection (OR[95%CI], 1.1 (1, 1.1), *P* = 2.60E-05). Genetically predicted higher red blood cell counts was associated with a reduced risk of SARS-CoV-2 reported infection (OR[95%CI], 0.94 [0.9, 0.98], *P* = 0.0016).

Supplementary discussion on study limitations

A central challenge for the COVID-19 HGI was to define the phenotypes and analytic pipelines from the outset of the Initiative so that these would be applicable to cohorts with extremely heterogeneous designs, sample ascertainment and control populations. Large-scale biobanks with existing genotype resources and connections to medical systems, newly enrolled hospital-based studies (particularly well-powered to study the extremes of severity by through the recruitment of individuals from intensive care units), and direct-to-consumer genetics studies with customer surveys each contributed different aspects to understanding the genetic basis of susceptibility and severity traits. Indeed, working together through aligning phenotype definitions and sharing results accelerated progress and has enhanced the robustness of the reported findings.

Nevertheless, the differences in study sample size, ascertainment and phenotyping of COVID-19 cases are unavoidable and care should be taken when interpreting the results from a meta-analysis because of

challenges with cases and controls ascertainment and collider bias. First, studies enriched with severe cases or studies with antibody-tested controls may disproportionately contribute to genetic discovery despite potentially smaller sample sizes. Second, differences in genomic profiling technology, imputation, and sample size across the constituent studies can have dramatic impacts on replication and downstream analyses (particularly fine-mapping where differential missing patterns in the reported results can muddy the signal). Third, the use of population controls with no complete information about SARS-CoV-2 exposure might result in cases of misclassification or reflect ascertainment biases in testing and reporting rather than true susceptibility to infection. Genotyping large numbers of control samples who have been exposed to the virus but remained asymptomatic or experienced only mild symptoms is challenging. Therefore many studies prefer to use pre-existing datasets of genetically ancestry-matched samples as their controls, protecting against population stratification, but potentially introducing some of these biases. Our analysis comparing the discovery meta-analysis effects to one where controls were phenotypically refined, indicated that, for genome-wide significant variants, such bias was limited. Finally, sociodemographic factors may influence an individual's susceptibility to SARS-CoV-2 infection and COVID-19 severity. In particular, lower socioeconomic level is associated with a higher risk of infection and hospitalization [4]. This can result in collider bias which distorts the relationship between genetic variation and the phenotypes being examined [5]. Additionally, other factors such as time of infection, which affects mortality and critical care admissions [6]), or differences in vaccination schemes may change the sociodemographic characteristics of COVID-19 positive participants.

References

1. Genomewide Association Study of Severe Covid-19 with Respiratory Failure. *N. Engl. J. Med.* **383**, 1522–1534 (2020).
2. Pairo-Castineira, E. *et al.* Genetic mechanisms of critical illness in COVID-19. *Nature* **591**, 92–98 (2021).
3. Roberts, G. H. L. *et al.* AncestryDNA COVID-19 Host Genetic Study Identifies Three Novel Loci. *medRxiv* (2020).
4. Niedzwiedz, C. L. *et al.* Ethnic and socioeconomic differences in SARS-CoV-2 infection: prospective cohort study using UK Biobank. *BMC Med.* **18**, 160 (2020).
5. Griffith, G. J. *et al.* Collider bias undermines our understanding of COVID-19 disease risk and severity. *Nat. Commun.* **11**, 5749 (2020).
6. Dennis, J. M., McGovern, A. P., Vollmer, S. J. & Mateen, B. A. Improving Survival of Critical Care

Patients With Coronavirus Disease 2019 in England: A National Cohort Study, March to June 2020. *Crit. Care Med.* **49**, 209–214 (2021).

Supplementary Table titles and summaries

Supplementary Table 1. Information regarding studies contributing to the consortium. Summary information from all independent studies that contributed data to at least one of the three main all- ancestry meta-analyses, including summary information about sex and age, cohort ethics and data analysis.

Supplementary Table 2. Genome-wide significant results from three COVID-19 phenotype meta-analyses. Detailed summary statistics for each locus that reached genome-wide significance in the three main meta-analyses, including gene prioritization results.

Supplementary Table 3. COVID-19 meta-analysis lead variant results stratified by ancestry. We report lead variant summary information about each locus that reached genome-wide significance in the three main meta-analyses, stratified by genetic ancestry.

Supplementary Table 4. Comparison of lead variant effect sizes between pairs of meta-analyses. Comparison of effect sizes for lead variants in genome-wide significant loci.

Supplementary Table 5. eGenes in GTEx v8 or eQTL catalogue. Genes with at least one fine-mapped cis-eQTL variant (PIP > 0.1) that is in LD with a lead variant (r2 > 0.6) in any tissue of GTEx v8 or eQTL catalogue. The r2 column represents the r2 value between the COVID-19 lead variant and eQTL variant. In Table 1 we report only eGenes for Lung tissue.

Supplementary Table 6. PheWAS associations for GWAS lead variants in LD (r2>0.8) with COVID-19 index variants. The associations were retrieved from OpenTargetsGenetics, UK Biobank and FinnGen.

Supplementary Table 7. Significant cis-eQTL associations in the Lung eQTL Consortium that are in LD with the COVID-19 lead variants.

Supplementary Table 8. SNP heritability estimated for three meta-analysis phenotypes. We used LD score regression (LDSC) to estimate SNP heritability from the European-only summary statistics and All ancestries summary statistics, for our three meta-analysis phenotypes.

Supplementary Table 9. Heritability enrichment in genes specifically expressed in tissue types. We used partitioned LD score regression to calculate heritability enrichment, using

European-only samples and All-ancestries meta-analyses, for our three main COVID-19 phenotypes.

Supplementary Table 10. Traits investigated in the genetic correlation and Mendelian randomization analyses. Information about publicly available summary statistics used in the genetic correlation and Mendelian randomization analyses.

Supplementary Table 11: Genetic correlation results between complex traits and COVID phenotypes (EUR only).

Supplementary Table 12: Causal association of complex traits on COVID-19 phenotypes (EUR only and EUR only w/o UKBB). Mendelian randomization results for covid phenotypes.

Supplementary Table 13. Study specific acknowledgements and competing interests.
