# Supplementary material for: Mapping the human genetic architecture of COVID-19
Source: Nature. 2021 Jul 8;600(7889):472–7. doi: 10.1038/s41586-021-03767-x (PMC8674144; doi:10.1038/s41586-021-03767-x)
Supplement: Supplementary file 3 — Quantile-quantile plots for GWAS from all individual studies that contributed data. QQ-plots showing the expected -log10(P-values) on the x-axis and the observed unadjusted P-values values from two-tailed inverse variance weighted meta-analysis on the y-axis (red line showing no deviation from the expected) for each study contributing data to the analyses. Sample size of cases and controls is listed for each study in the plot title, as well as the median lambda value. [file 41586_2021_3767_MOESM3_ESM.pdf]

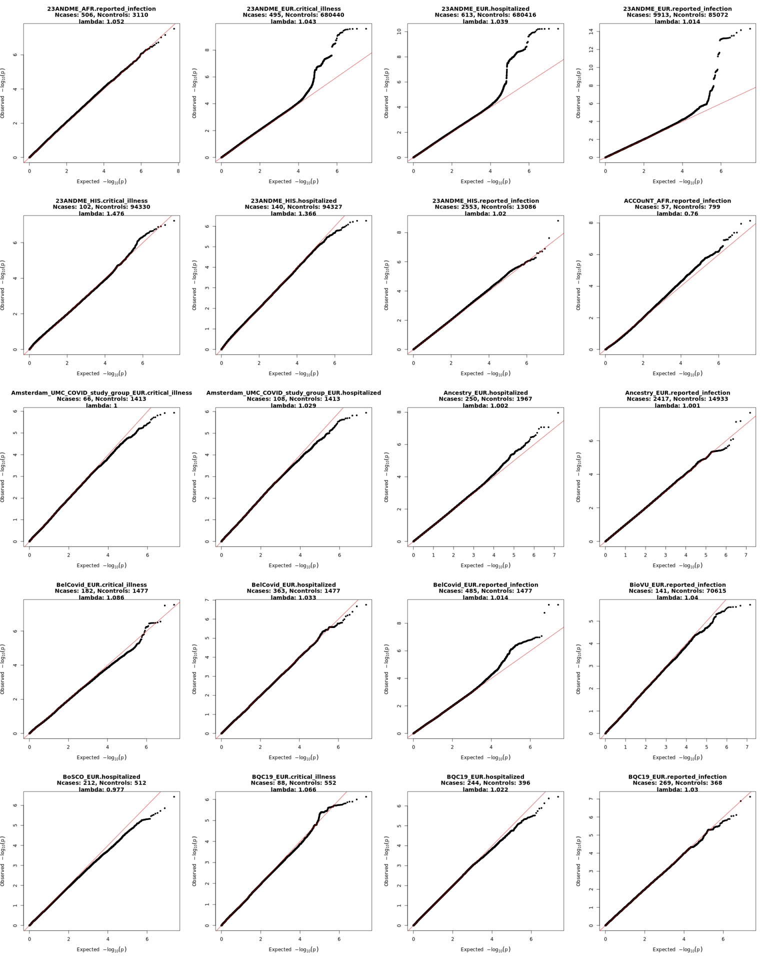
23ANDME_AFR.reported_infection
Ncases: 506, Ncontrols: 3110
lambda: 1.052
23ANDME_EUR.critical_illness
Ncases: 495, Ncontrols: 680440
lambda: 1.043
23ANDME_EUR.hospitalized
Ncases: 613, Ncontrols: 680416
lambda: 1.039
23ANDME_EUR.reported_infection
Ncases: 9913, Ncontrols: 85072
lambda: 1.014
23ANDME_HIS.critical_illness
Ncases: 102, Ncontrols: 94330
lambda: 1.476
23ANDME_HIS.hospitalized
Ncases: 140, Ncontrols: 94327
lambda: 1.366
23ANDME_HIS.reported_infection
Ncases: 2553, Ncontrols: 13086
lambda: 1.02
ACCOuNT_AFR.reported_infection
Ncases: 57, Ncontrols: 799
lambda: 0.76
Amsterdam_UMC_COVID_study_group_EUR.critical_illness
Ncases: 66, Ncontrols: 1413
lambda: 1
Amsterdam_UMC_COVID_study_group_EUR.hospitalized
Ncases: 108, Ncontrols: 1413
lambda: 1.029
Ancestry_EUR.hospitalized
Ncases: 250, Ncontrols: 1967
lambda: 1.002
Ancestry_EUR.reported_infection
Ncases: 2417, Ncontrols: 14933
lambda: 1.001
BelCovid_EUR.critical_illness
Ncases: 182, Ncontrols: 1477
lambda: 1.086
BelCovid_EUR.hospitalized
Ncases: 363, Ncontrols: 1477
lambda: 1.033
BelCovid_EUR.reported_infection
Ncases: 485, Ncontrols: 1477
lambda: 1.014
BioVU_EUR.reported_infection
Ncases: 141, Ncontrols: 70615
lambda: 1.04
BoSCO_EUR.hospitalized
Ncases: 212, Ncontrols: 512
lambda: 0.977
BQC19_EUR.critical_illness
Ncases: 88, Ncontrols: 552
lambda: 1.066
BQC19_EUR.hospitalized
Ncases: 244, Ncontrols: 396
lambda: 1.022
BQC19_EUR.reported_infection
Ncases: 269, Ncontrols: 368
lambda: 1.03
Expected −log10(p)
Observed −log10(p)

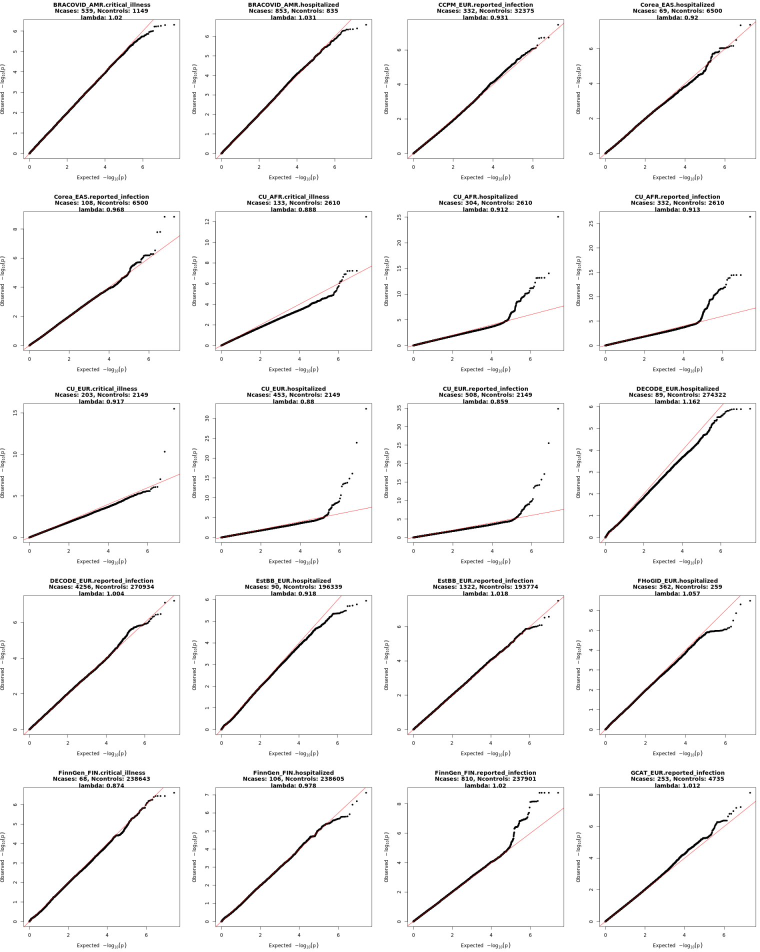
BRACOVID_AMR.critical_illness
Ncases: 539, Ncontrols: 1149
lambda: 1.02
BRACOVID_AMR.hospitalized
Ncases: 853, Ncontrols: 835
lambda: 1.031
CCPM_EUR.reported_infection
Ncases: 332, Ncontrols: 32375
lambda: 0.931
Corea_EAS.hospitalized
Ncases: 69, Ncontrols: 6500
lambda: 0.92
Corea_EAS.reported_infection
Ncases: 108, Ncontrols: 6500
lambda: 0.968
CU_AFR.critical_illness
Ncases: 133, Ncontrols: 2610
lambda: 0.888
CU_AFR.hospitalized
Ncases: 304, Ncontrols: 2610
lambda: 0.912
CU_AFR.reported_infection
Ncases: 332, Ncontrols: 2610
lambda: 0.913
CU_EUR.critical_illness
Ncases: 203, Ncontrols: 2149
lambda: 0.917
CU_EUR.hospitalized
Ncases: 453, Ncontrols: 2149
lambda: 0.88
CU_EUR.reported_infection
Ncases: 508, Ncontrols: 2149
lambda: 0.859
DECODE_EUR.hospitalized
Ncases: 89, Ncontrols: 274322
lambda: 1.162
DECODE_EUR.reported_infection
Ncases: 4256, Ncontrols: 270934
lambda: 1.004
EstBB_EUR.hospitalized
Ncases: 90, Ncontrols: 196339
lambda: 0.918
EstBB_EUR.reported_infection
Ncases: 1322, Ncontrols: 193774
lambda: 1.018
FHoGID_EUR.hospitalized
Ncases: 362, Ncontrols: 259
lambda: 1.057
FinnGen_FIN.critical_illness
Ncases: 68, Ncontrols: 238643
lambda: 0.874
FinnGen_FIN.hospitalized
Ncases: 106, Ncontrols: 238605
lambda: 0.978
FinnGen_FIN.reported_infection
Ncases: 810, Ncontrols: 237901
lambda: 1.02
GCAT_EUR.reported_infection
Ncases: 253, Ncontrols: 4735
lambda: 1.012
Expected −log10(p)
Observed −log10(p)

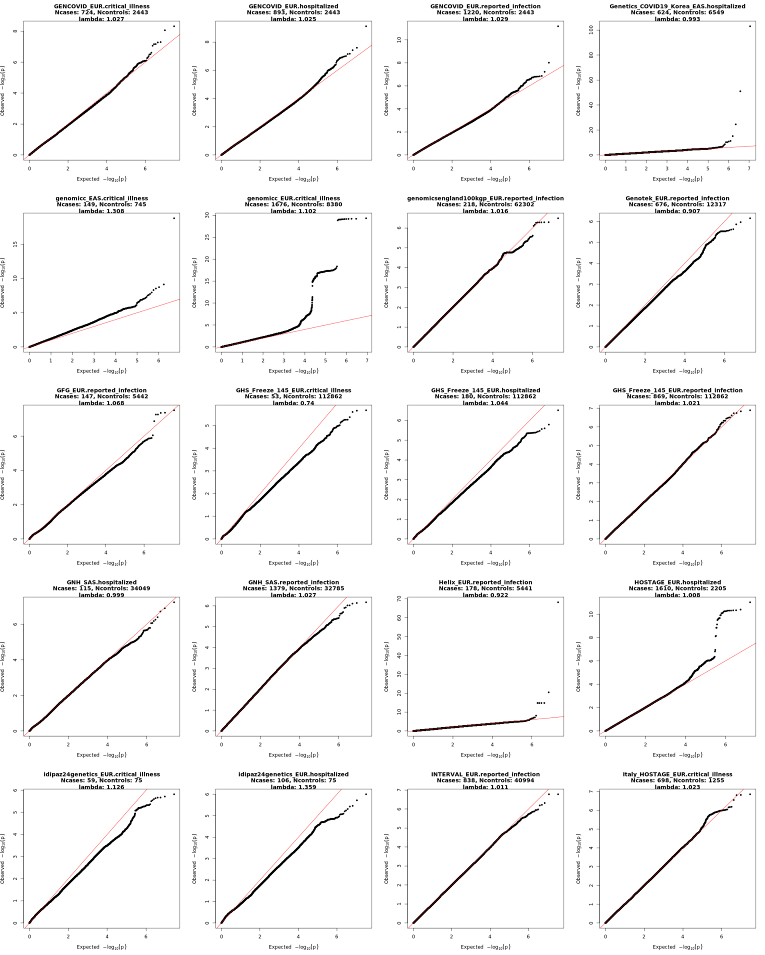
GENCOVID_EUR.critical_illness
Ncases: 724, Ncontrols: 2443
lambda: 1.027
GENCOVID_EUR.hospitalized
Ncases: 893, Ncontrols: 2443
lambda: 1.025
GENCOVID_EUR.reported_infection
Ncases: 1220, Ncontrols: 2443
lambda: 1.029
Genetics_COVID19_Korea_EAS.hospitalized
Ncases: 624, Ncontrols: 6549
lambda: 0.993
genomicc_EAS.critical_illness
Ncases: 149, Ncontrols: 745
lambda: 1.308
genomicc_EUR.critical_illness
Ncases: 1676, Ncontrols: 8380
lambda: 1.102
genomicsengland100kgp_EUR.reported_infection
Ncases: 218, Ncontrols: 62302
lambda: 1.016
Genotek_EUR.reported_infection
Ncases: 676, Ncontrols: 12317
lambda: 0.907
GFG_EUR.reported_infection
Ncases: 147, Ncontrols: 5442
lambda: 1.068
GHS_Freeze_145_EUR.critical_illness
Ncases: 53, Ncontrols: 112862
lambda: 0.74
GHS_Freeze_145_EUR.hospitalized
Ncases: 180, Ncontrols: 112862
lambda: 1.044
GHS_Freeze_145_EUR.reported_infection
Ncases: 869, Ncontrols: 112862
lambda: 1.021
GNH_SAS.hospitalized
Ncases: 115, Ncontrols: 34049
lambda: 0.999
GNH_SAS.reported_infection
Ncases: 1379, Ncontrols: 32785
lambda: 1.027
Helix_EUR.reported_infection
Ncases: 178, Ncontrols: 5441
lambda: 0.922
HOSTAGE_EUR.hospitalized
Ncases: 1610, Ncontrols: 2205
lambda: 1.008
idipaz24genetics_EUR.critical_illness
Ncases: 59, Ncontrols: 75
lambda: 1.126
idipaz24genetics_EUR.hospitalized
Ncases: 106, Ncontrols: 75
lambda: 1.359
INTERVAL_EUR.reported_infection
Ncases: 838, Ncontrols: 40994
lambda: 1.011
Italy_HOSTAGE_EUR.critical_illness
Ncases: 698, Ncontrols: 1255
lambda: 1.023
Expected −log10(p)
Observed −log10(p)

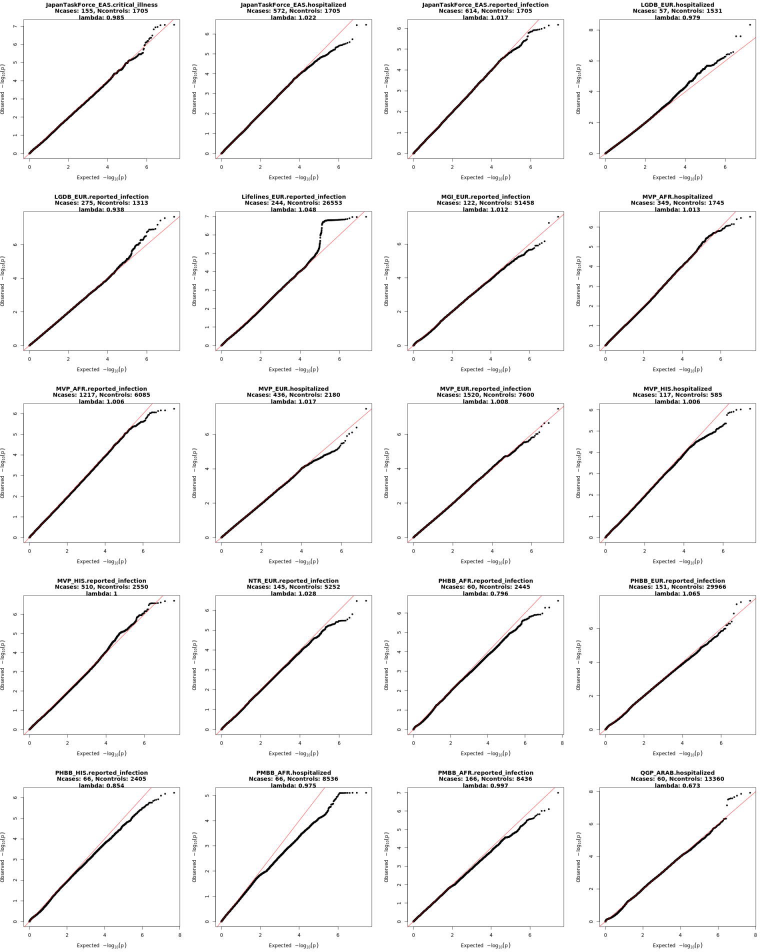
JapanTaskForce_EAS.critical_illness
Ncases: 155, Ncontrols: 1705
lambda: 0.985
JapanTaskForce_EAS.hospitalized
Ncases: 572, Ncontrols: 1705
lambda: 1.022
JapanTaskForce_EAS.reported_infection
Ncases: 614, Ncontrols: 1705
lambda: 1.017
LGDB_EUR.hospitalized
Ncases: 57, Ncontrols: 1531
lambda: 0.979
LGDB_EUR.reported_infection
Ncases: 275, Ncontrols: 1313
lambda: 0.938
Lifelines_EUR.reported_infection
Ncases: 244, Ncontrols: 26553
lambda: 1.048
MGI_EUR.reported_infection
Ncases: 122, Ncontrols: 51458
lambda: 1.012
MVP_AFR.hospitalized
Ncases: 349, Ncontrols: 1745
lambda: 1.013
MVP_AFR.reported_infection
Ncases: 1217, Ncontrols: 6085
lambda: 1.006
MVP_EUR.hospitalized
Ncases: 436, Ncontrols: 2180
lambda: 1.017
MVP_EUR.reported_infection
Ncases: 1520, Ncontrols: 7600
lambda: 1.008
MVP_HIS.hospitalized
Ncases: 117, Ncontrols: 585
lambda: 1.006
MVP_HIS.reported_infection
Ncases: 510, Ncontrols: 2550
lambda: 1
NTR_EUR.reported_infection
Ncases: 145, Ncontrols: 5252
lambda: 1.028
PHBB_AFR.reported_infection
Ncases: 60, Ncontrols: 2445
lambda: 0.796
PHBB_EUR.reported_infection
Ncases: 151, Ncontrols: 29966
lambda: 1.065
PHBB_HIS.reported_infection
Ncases: 66, Ncontrols: 2405
lambda: 0.854
PMBB_AFR.hospitalized
Ncases: 66, Ncontrols: 8536
lambda: 0.975
PMBB_AFR.reported_infection
Ncases: 166, Ncontrols: 8436
lambda: 0.997
QGP_ARAB.hospitalized
Ncases: 60, Ncontrols: 13360
lambda: 0.673
Observed −log10(p)
Expected −log10(p)

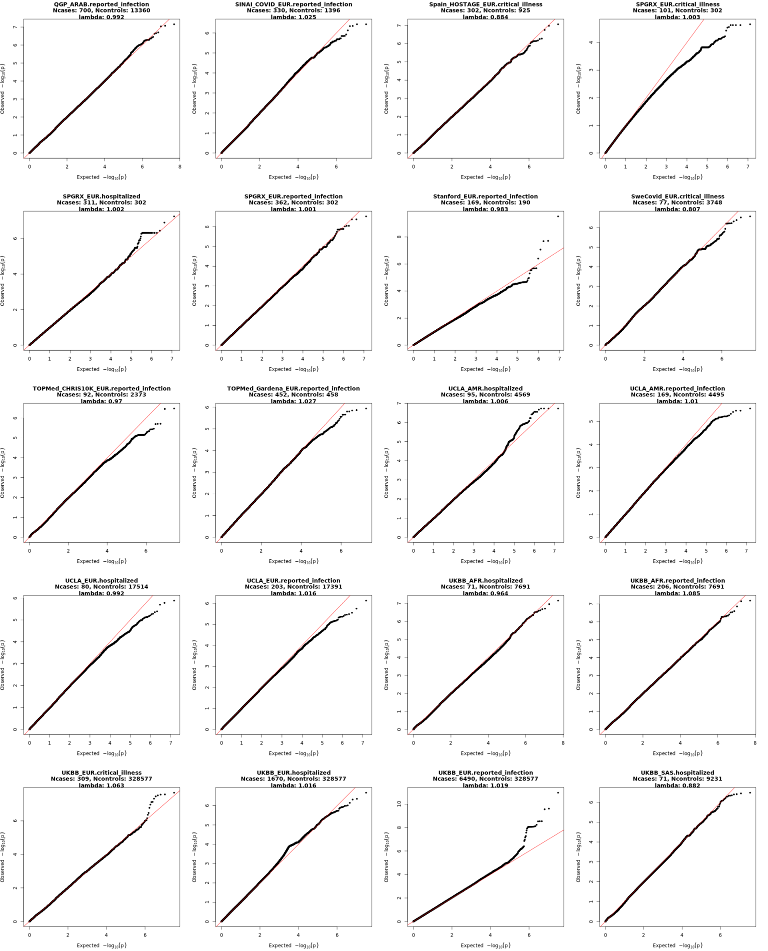
QGP_ARAB.reported_infection
Ncases: 700, Ncontrols: 13360
lambda: 0.992
SINAI_COVID_EUR.reported_infection
Ncases: 330, Ncontrols: 1396
lambda: 1.025
Spain_HOSTAGE_EUR.critical_illness
Ncases: 302, Ncontrols: 925
lambda: 0.884
SPGRX_EUR.critical_illness
Ncases: 101, Ncontrols: 302
lambda: 1.003
SPGRX_EUR.hospitalized
Ncases: 311, Ncontrols: 302
lambda: 1.002
SPGRX_EUR.reported_infection
Ncases: 362, Ncontrols: 302
lambda: 1.001
Stanford_EUR.reported_infection
Ncases: 169, Ncontrols: 190
lambda: 0.983
SweCovid_EUR.critical_illness
Ncases: 77, Ncontrols: 3748
lambda: 0.807
TOPMed_CHRIS10K_EUR.reported_infection
Ncases: 92, Ncontrols: 2373
lambda: 0.97
TOPMed_Gardena_EUR.reported_infection
Ncases: 452, Ncontrols: 458
lambda: 1.027
UCLA_AMR.hospitalized
Ncases: 95, Ncontrols: 4569
lambda: 1.006
UCLA_AMR.reported_infection
Ncases: 169, Ncontrols: 4495
lambda: 1.01
UCLA_EUR.hospitalized
Ncases: 80, Ncontrols: 17514
lambda: 0.992
UCLA_EUR.reported_infection
Ncases: 203, Ncontrols: 17391
lambda: 1.016
UKBB_AFR.hospitalized
Ncases: 71, Ncontrols: 7691
lambda: 0.964
UKBB_AFR.reported_infection
Ncases: 206, Ncontrols: 7691
lambda: 1.085
UKBB_EUR.critical_illness
Ncases: 309, Ncontrols: 328577
lambda: 1.063
UKBB_EUR.hospitalized
Ncases: 1670, Ncontrols: 328577
lambda: 1.016
UKBB_EUR.reported_infection
Ncases: 6490, Ncontrols: 328577
lambda: 1.019
UKBB_SAS.hospitalized
Ncases: 71, Ncontrols: 9231
lambda: 0.882
Expected −log10(p)
Observed −log10(p)

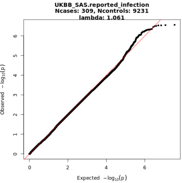
UKBB_SAS.reported_infection
Ncases: 309, Ncontrols: 9231
lambda: 1.061
Observed −log10(p)
Expected −log10(p)
0
1
2
3
4
5
6
0
2
4
6
