# Supplementary material for: Mapping the human genetic architecture of COVID-19
Source: Nature. 2021 Jul 8;600(7889):472–7. doi: 10.1038/s41586-021-03767-x (PMC8674144; doi:10.1038/s41586-021-03767-x)
Supplement: Supplementary file 5 — Scatter and funnel plots for each for exposure - COVID-19 outcome pair. Scatter plots show the exposure variant effect size against the COVID-19 outcome variant effect size and corresponding standard errors. Funnel plots show the Mendelian randomization (MR) causal estimates for each variant against their precision, with asymmetry in the plot indicating potential violations of the assumptions of MR. Regression lines show the corresponding causal estimates fixed effect inverse-weighted (IVW, red-solid line) meta-analysis; MR-Egger regression (blue-dashed); Weighted median estimator (WME, green-dashed); weighted mode based estimator (WMBE, purple-dashed); and Mendelian Randomization Pleiotropy RESidual Sum and Outlier corrected (MR-PRESSO, orange-dashed). Variants highlighted in red were flagged as outliers by MR-PRESSO. [file 41586_2021_3767_MOESM5_ESM.pdf]

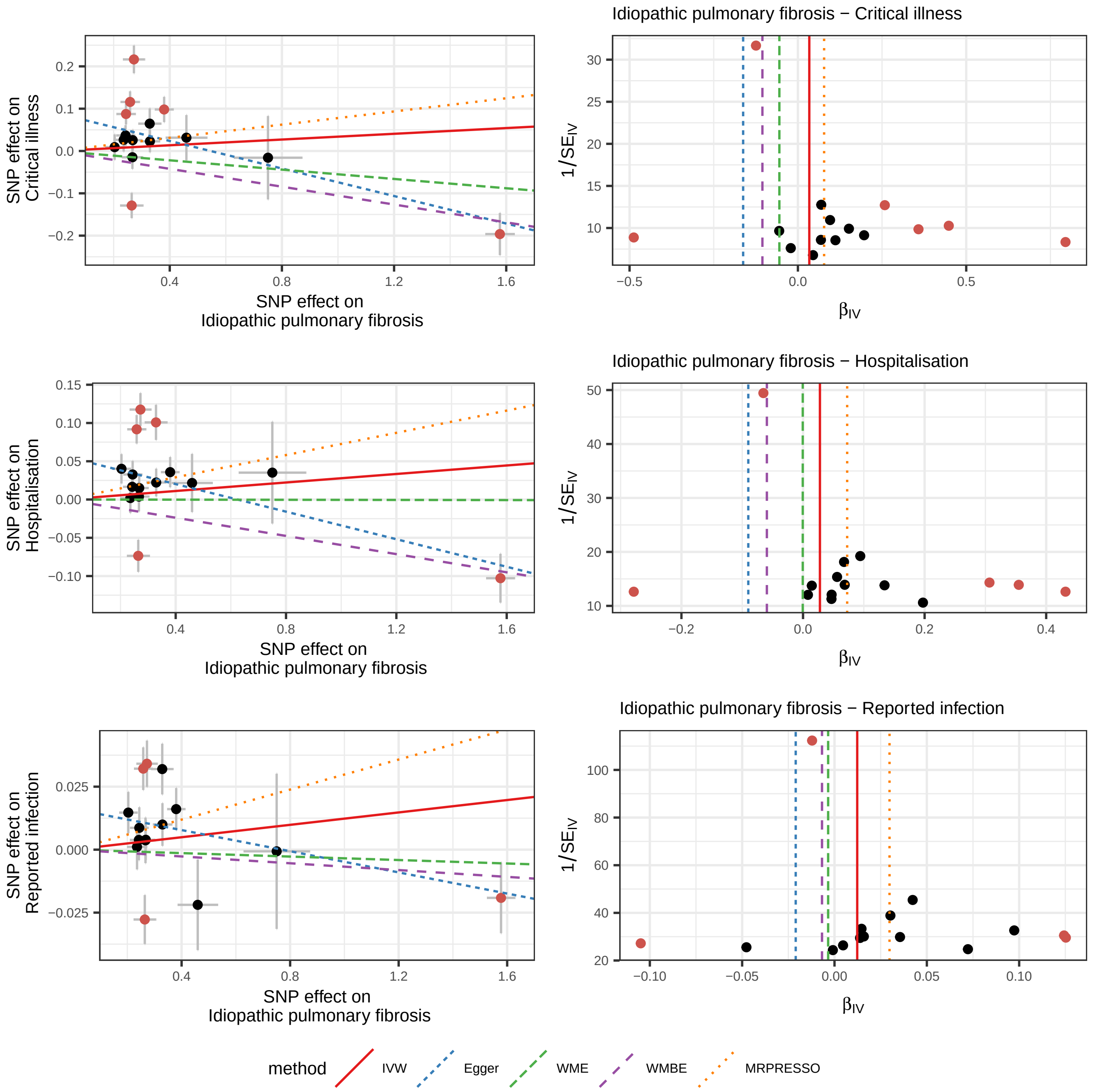

Idiopathic pulmonary fibrosis − Critical illness
Idiopathic pulmonary fibrosis − Hospitalisation
Idiopathic pulmonary fibrosis − Reported infection
SNP effect on Critical illness
SNP effect on Hospitalisation
SNP effect on Reported infection
SNP effect on Idiopathic pulmonary fibrosis
$1/SE_{IV}$
β_{IV}
method
IVW
Egger
WME
WMBE
MRPRESSO

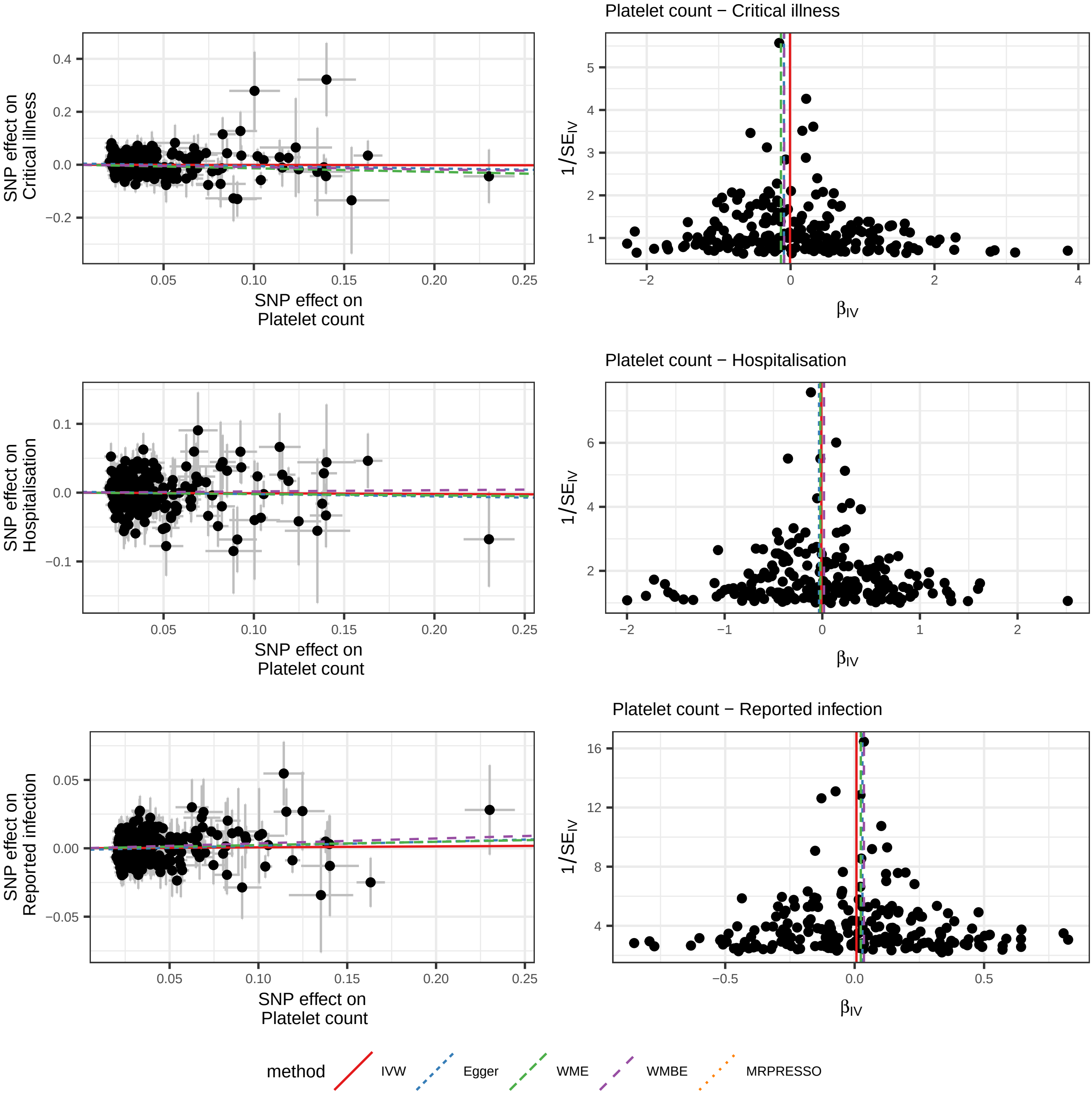

Platelet count − Critical illness
SNP effect on Critical illness
SNP effect on Platelet count
1/SE_IV
β_IV
Platelet count − Hospitalisation
SNP effect on Hospitalisation
SNP effect on Platelet count
1/SE_IV
β_IV
Platelet count − Reported infection
SNP effect on Reported infection
SNP effect on Platelet count
1/SE_IV
β_IV
method
IVW
Egger
WME
WMBE
MRPRESSO

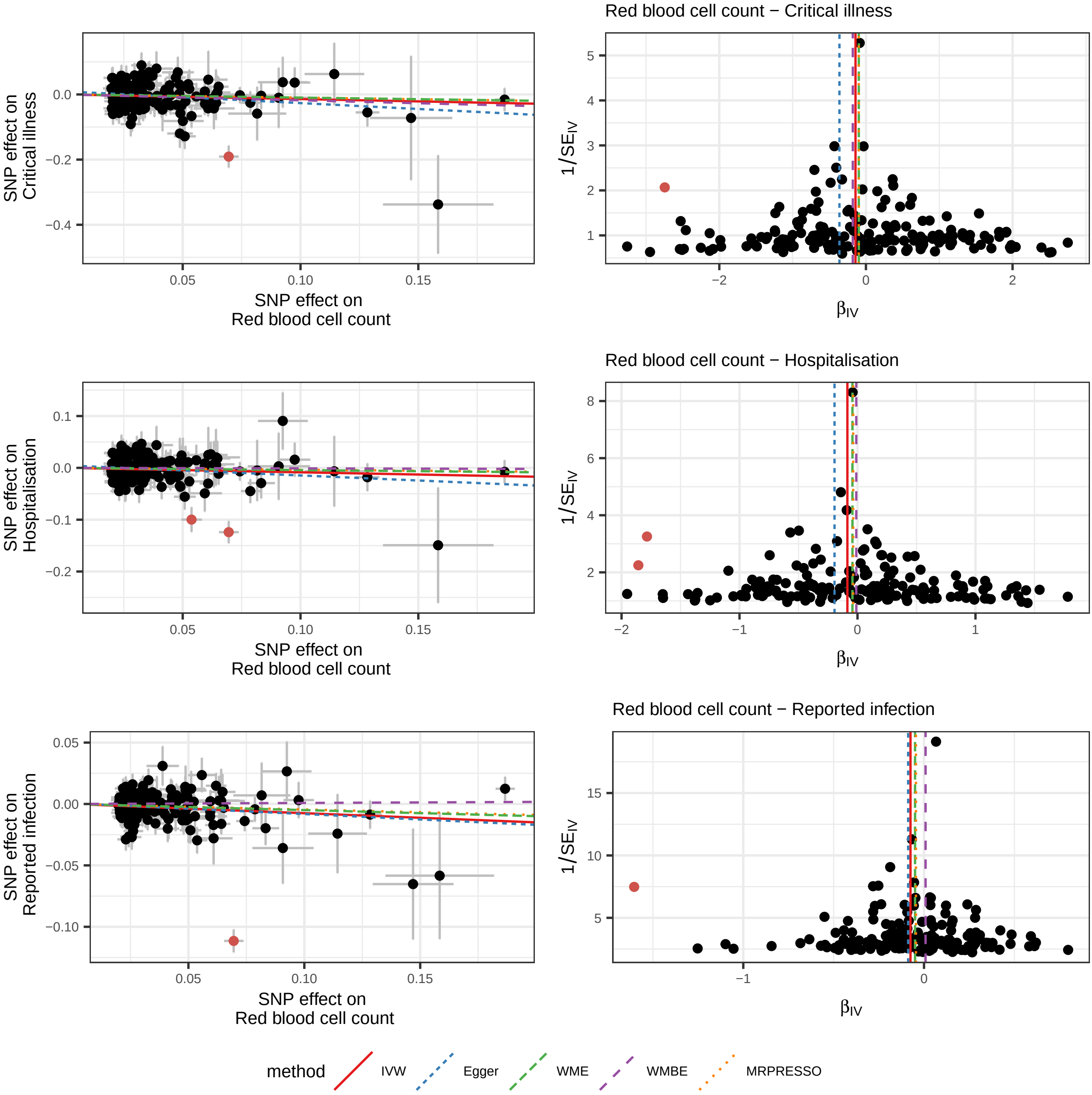

Red blood cell count − Critical illness
Red blood cell count − Hospitalisation
Red blood cell count − Reported infection
SNP effect on Critical illness
SNP effect on Hospitalisation
SNP effect on Reported infection
SNP effect on Red blood cell count
1/SE$_{IV}$
β$_{IV}$
method
IVW
Egger
WME
WMBE
MRPRESSO

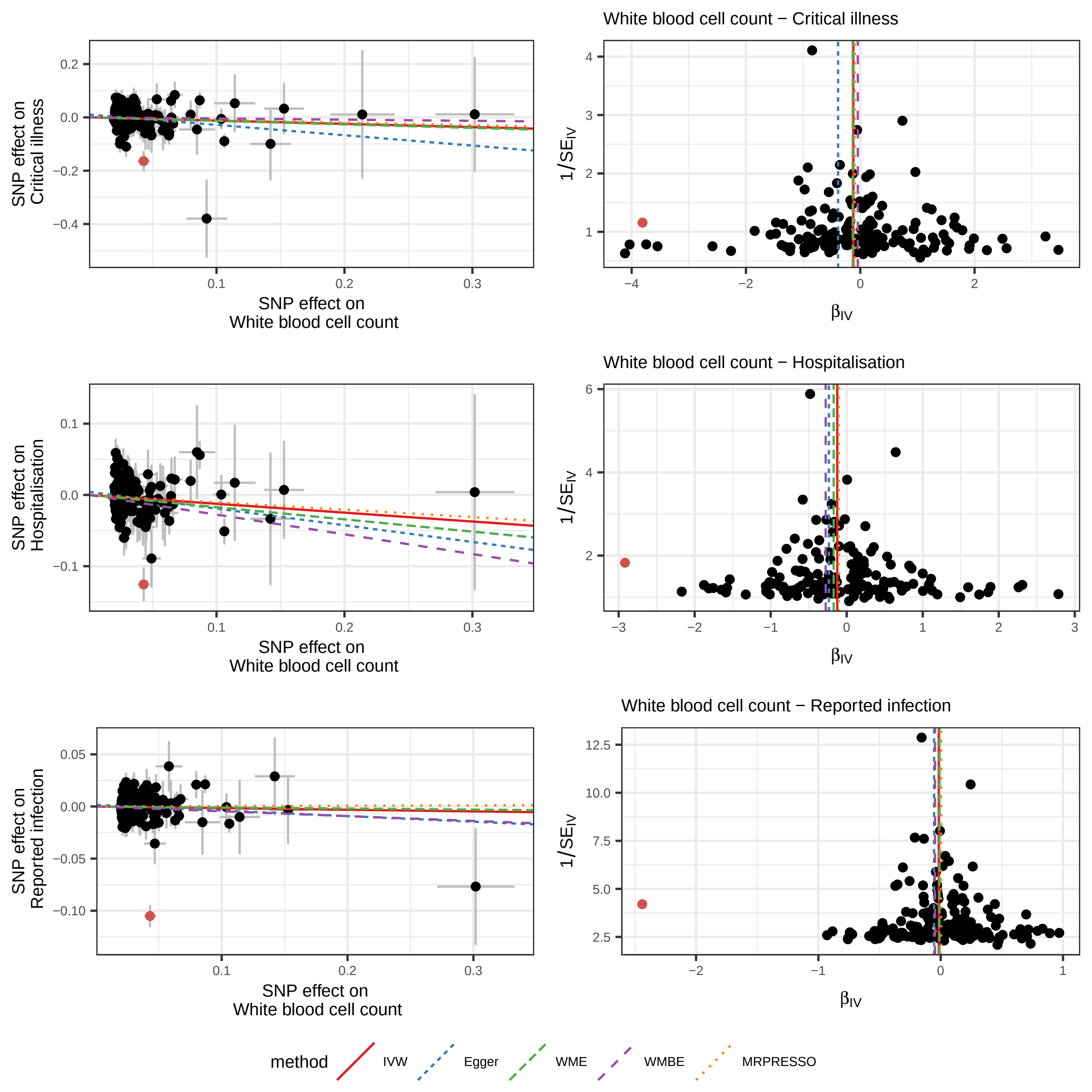

White blood cell count − Critical illness
White blood cell count − Hospitalisation
White blood cell count − Reported infection
SNP effect on Critical illness
SNP effect on Hospitalisation
SNP effect on Reported infection
SNP effect on White blood cell count
$1/SE_{IV}$
β_{IV}
method
IVW
Egger
WME
WMBE
MRPRESSO

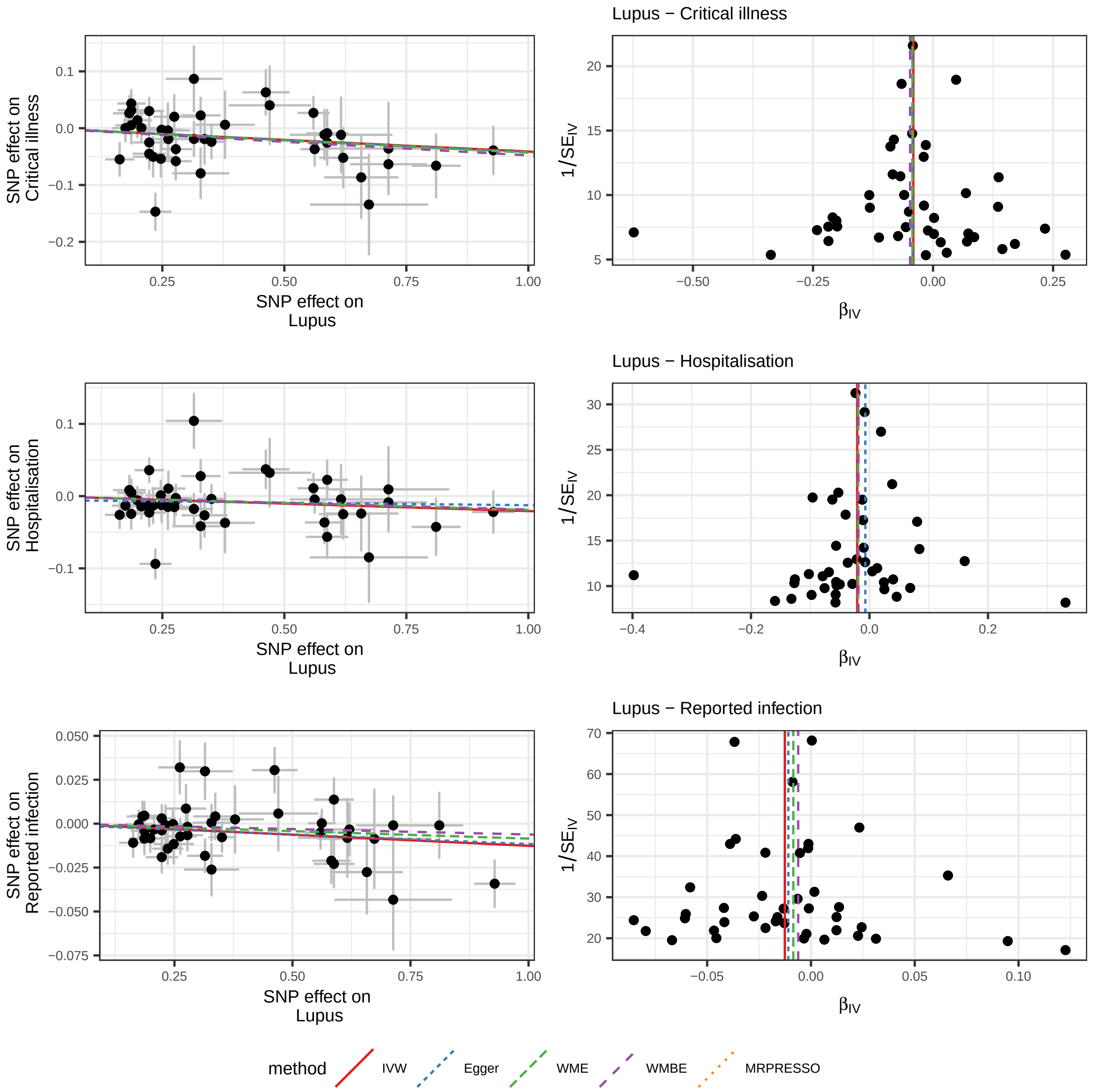

Lupus − Critical illness
Lupus − Hospitalisation
Lupus − Reported infection
SNP effect on Critical illness
SNP effect on Hospitalisation
SNP effect on Reported infection
SNP effect on Lupus
1/SE$_{IV}$
β$_{IV}$
method
IVW
Egger
WME
WMBE
MRPRESSO

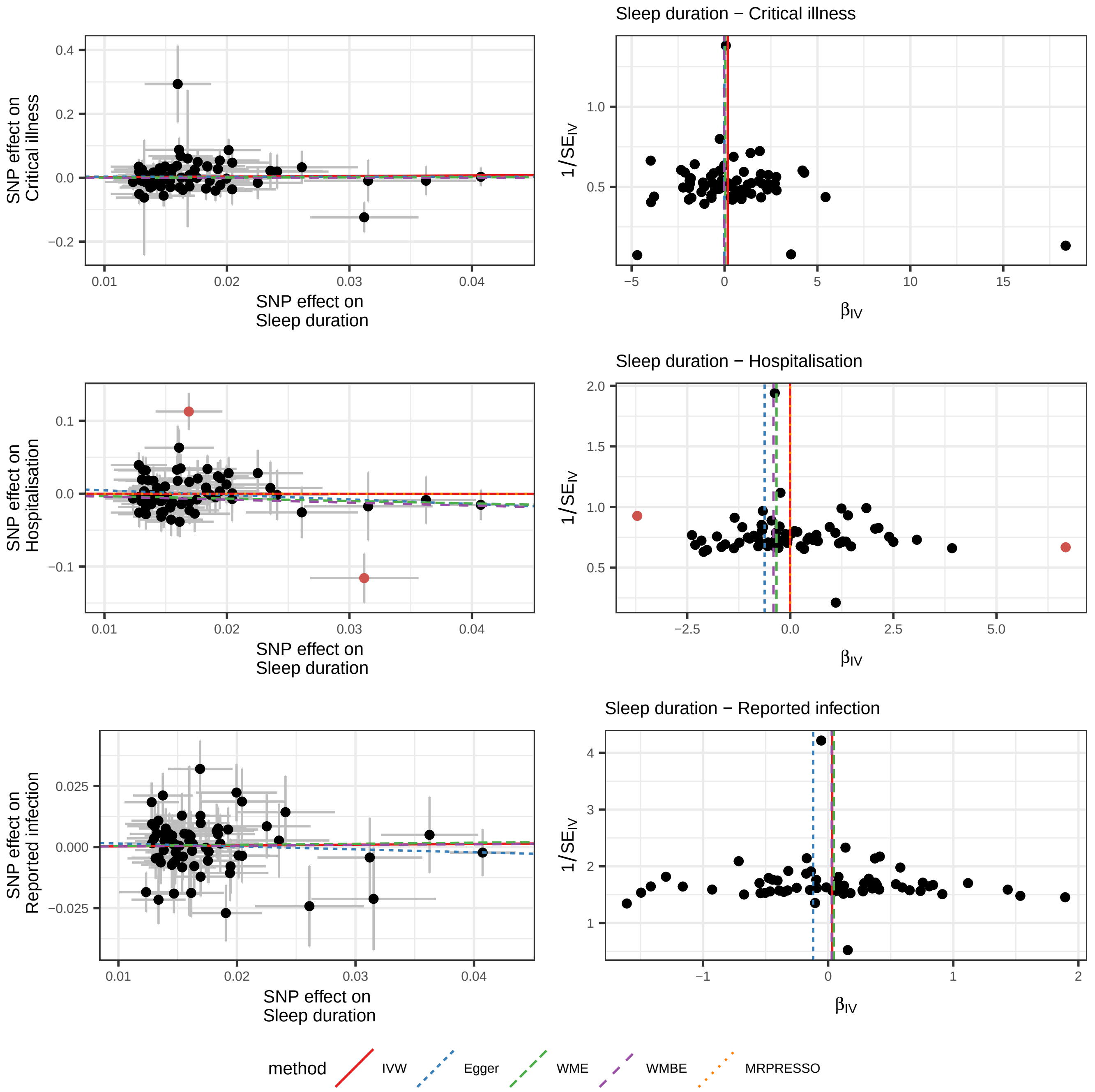
Sleep duration − Critical illness
Sleep duration − Hospitalisation
Sleep duration − Reported infection
SNP effect on Critical illness
SNP effect on Hospitalisation
SNP effect on Reported infection
SNP effect on Sleep duration
$1/SE_{IV}$
β_{IV}
method
IVW
Egger
WME
WMBE
MRPRESSO

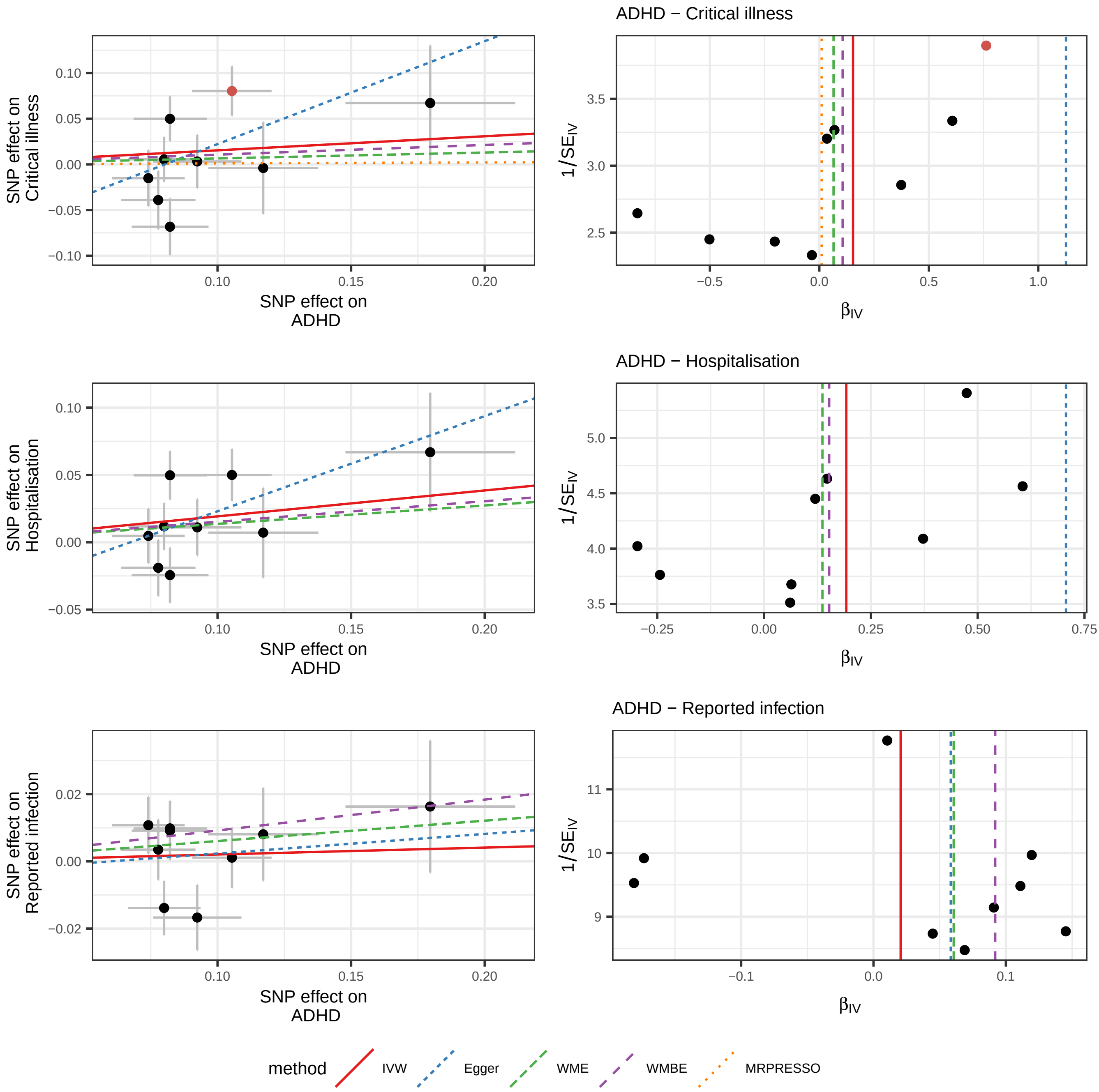

ADHD − Critical illness
ADHD − Hospitalisation
ADHD − Reported infection
SNP effect on Critical illness
SNP effect on Hospitalisation
SNP effect on Reported infection
SNP effect on ADHD
1/SE_IV
β_IV
method
IVW
Egger
WME
WMBE
MRPRESSO

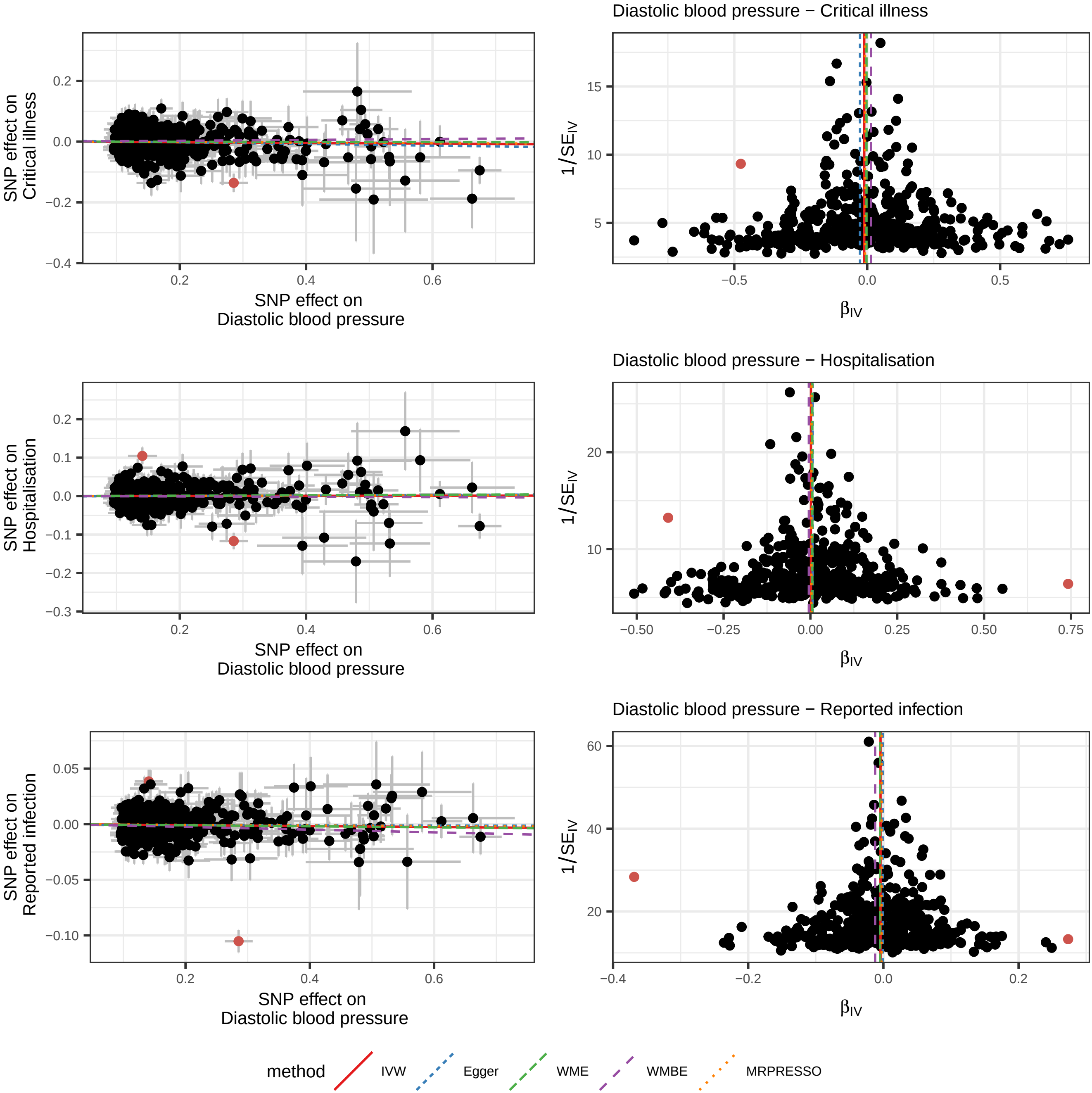

Diastolic blood pressure − Critical illness
Diastolic blood pressure − Hospitalisation
Diastolic blood pressure − Reported infection
SNP effect on Critical illness
SNP effect on Hospitalisation
SNP effect on Reported infection
SNP effect on Diastolic blood pressure
1/SE_IV
β_IV
method
IVW
Egger
WME
WMBE
MRPRESSO

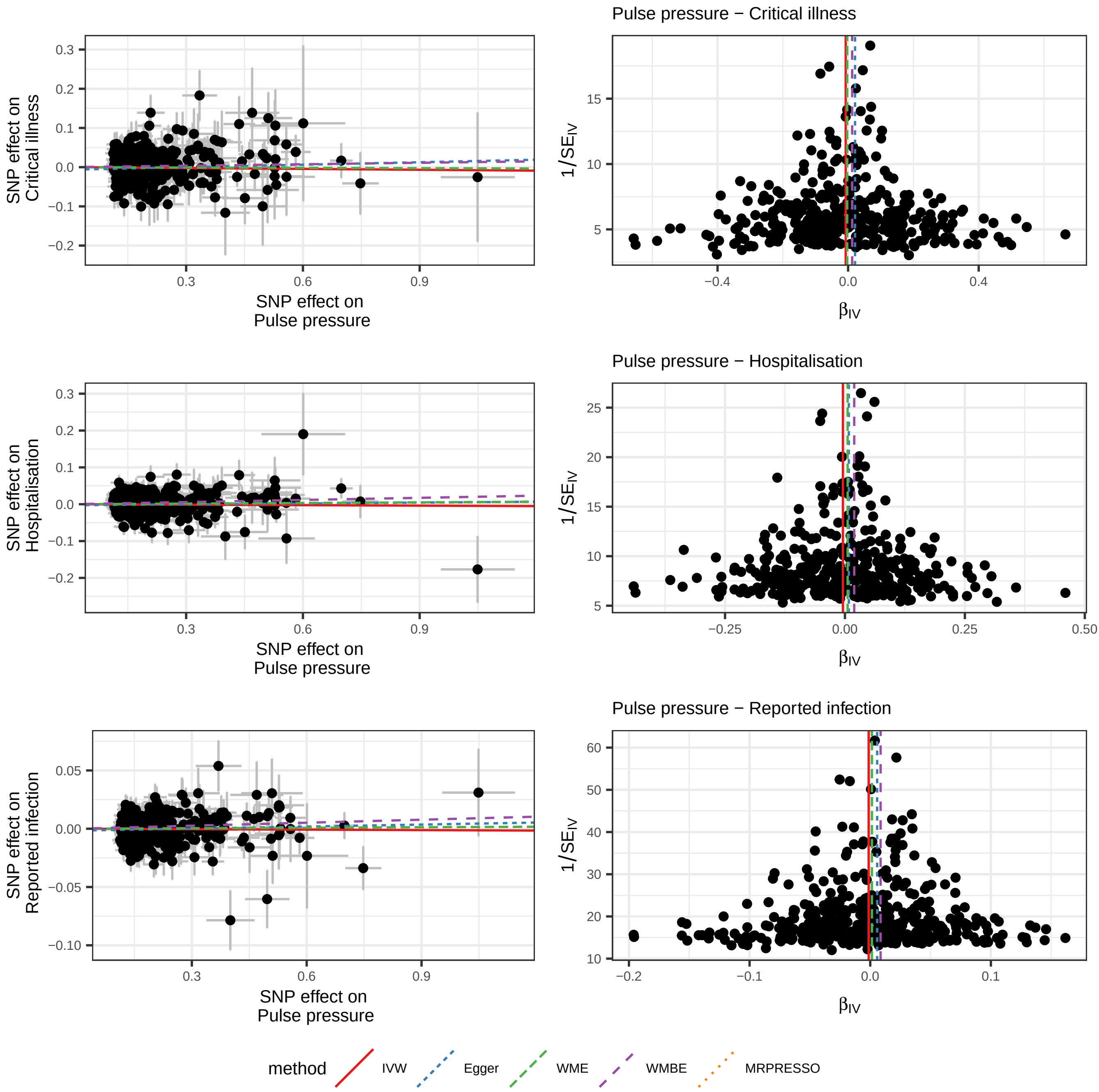

Pulse pressure − Critical illness
Pulse pressure − Hospitalisation
Pulse pressure − Reported infection
SNP effect on Critical illness
SNP effect on Hospitalisation
SNP effect on Reported infection
SNP effect on Pulse pressure
$1/SE_{IV}$
β_{IV}
method
IVW
Egger
WME
WMBE
MRPRESSO

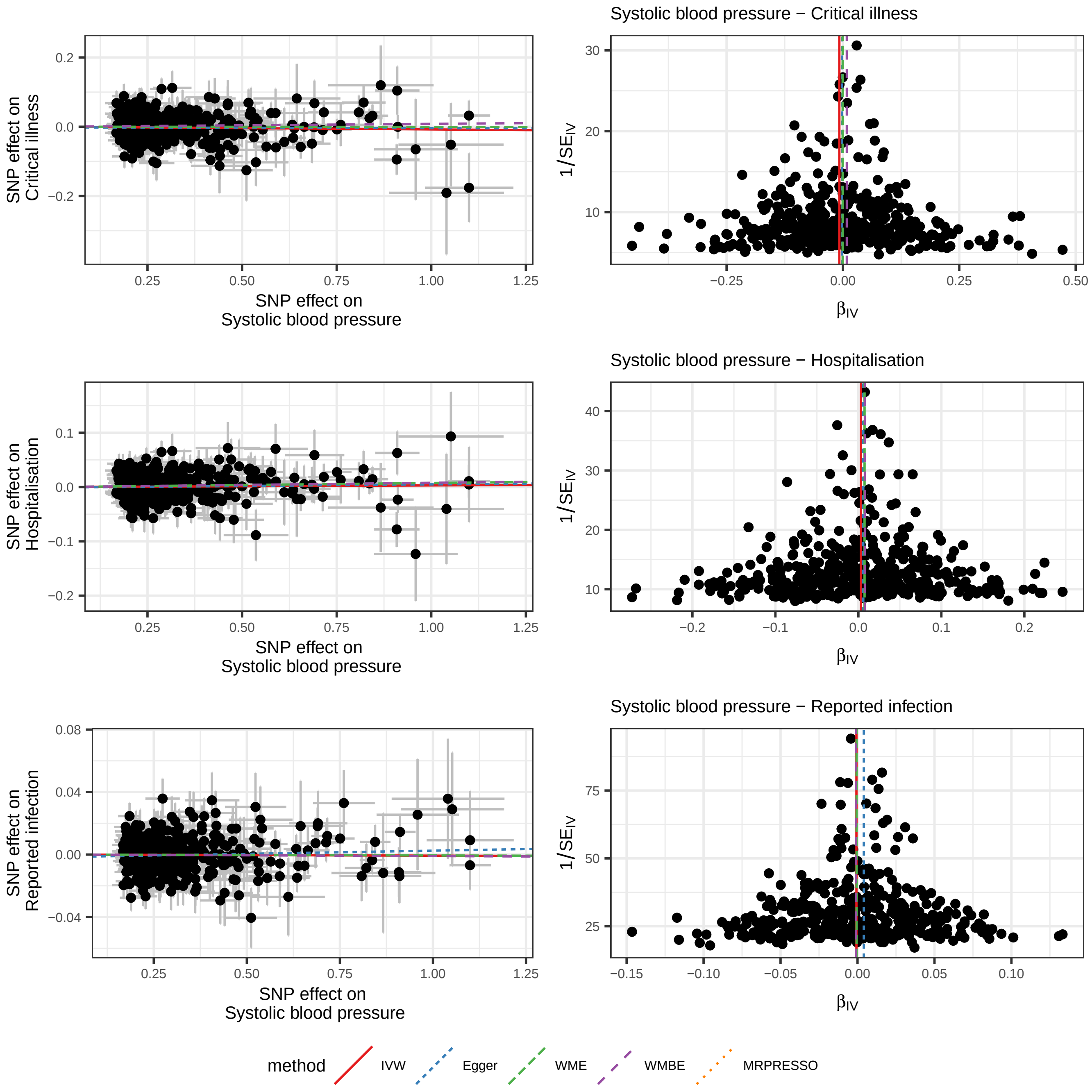

Systolic blood pressure − Critical illness
SNP effect on Critical illness
SNP effect on Systolic blood pressure
1/SE_IV
β_IV
Systolic blood pressure − Hospitalisation
SNP effect on Hospitalisation
SNP effect on Systolic blood pressure
1/SE_IV
β_IV
Systolic blood pressure − Reported infection
SNP effect on Reported infection
SNP effect on Systolic blood pressure
1/SE_IV
β_IV
method
IVW
Egger
WME
WMBE
MRPRESSO

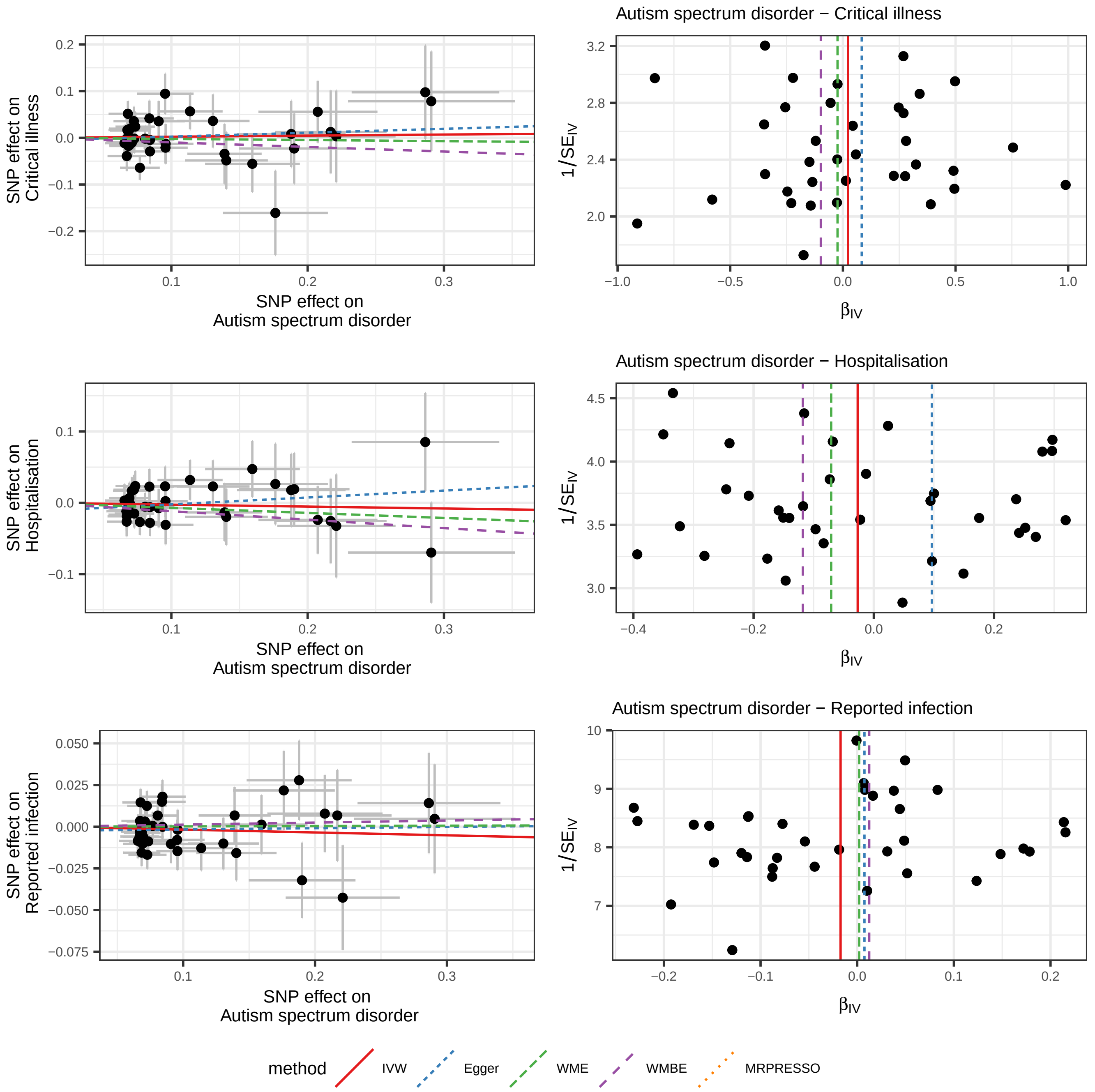

Autism spectrum disorder − Critical illness
SNP effect on Critical illness
SNP effect on Autism spectrum disorder
$1/SE_{IV}$
β_{IV}
Autism spectrum disorder − Hospitalisation
SNP effect on Hospitalisation
SNP effect on Autism spectrum disorder
$1/SE_{IV}$
β_{IV}
Autism spectrum disorder − Reported infection
SNP effect on Reported infection
SNP effect on Autism spectrum disorder
$1/SE_{IV}$
β_{IV}
method
IVW
Egger
WME
WMBE
MRPRESSO

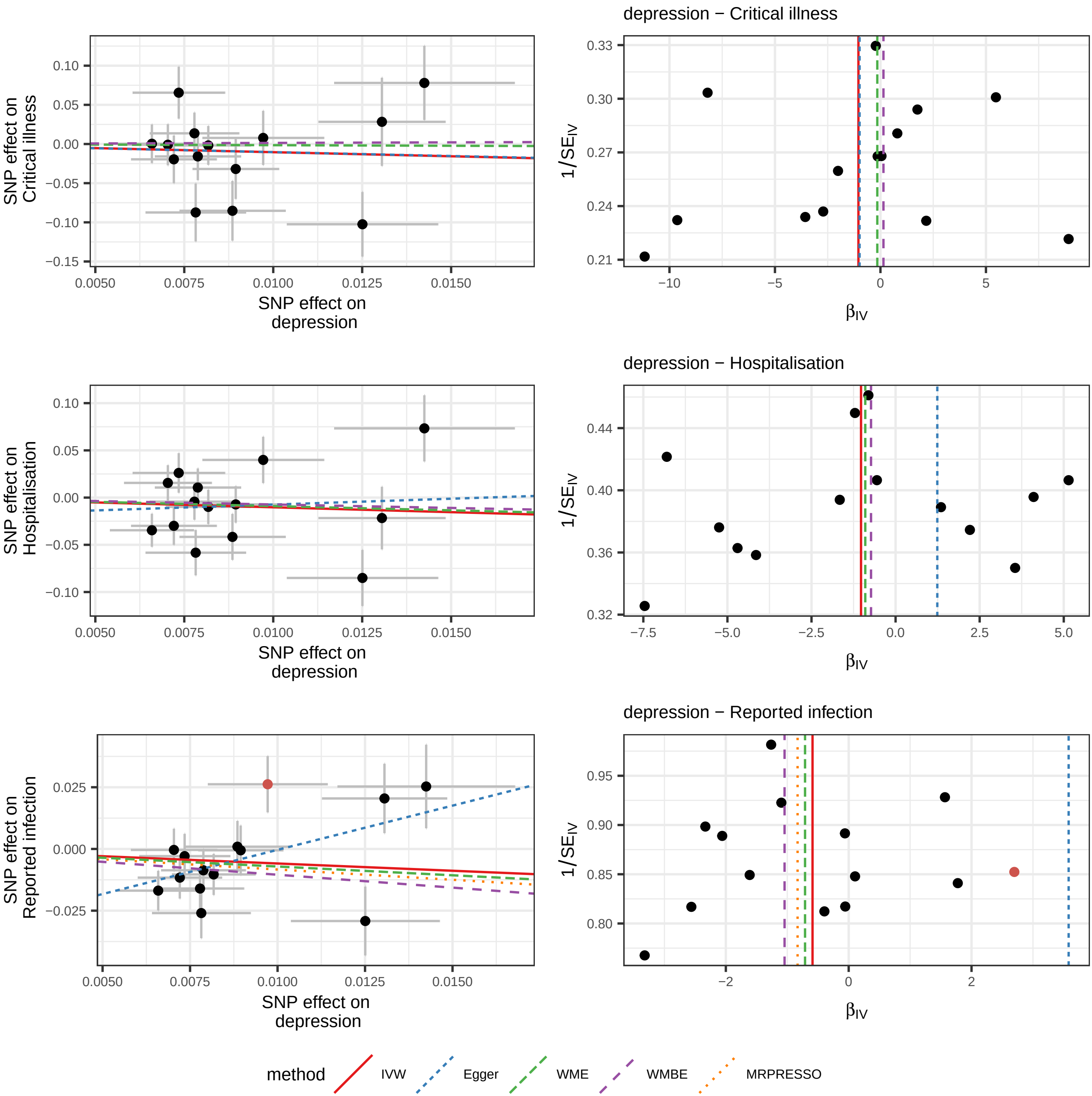
depression − Critical illness
depression − Hospitalisation
depression − Reported infection
SNP effect on Critical illness
SNP effect on Hospitalisation
SNP effect on Reported infection
SNP effect on depression
1/SE$_{IV}$
β$_{IV}$
0.0050
0.0075
0.0100
0.0125
0.0150
0.10
0.05
0.00
−0.05
−0.10
−0.15
0.025
0.000
−0.025
0.33
0.30
0.27
0.24
0.21
0.44
0.40
0.36
0.32
0.95
0.90
0.85
0.80
−10
−5
0
5
−7.5
−5.0
−2.5
0.0
2.5
5.0
−2
2
method
IVW
Egger
WME
WMBE
MRPRESSO

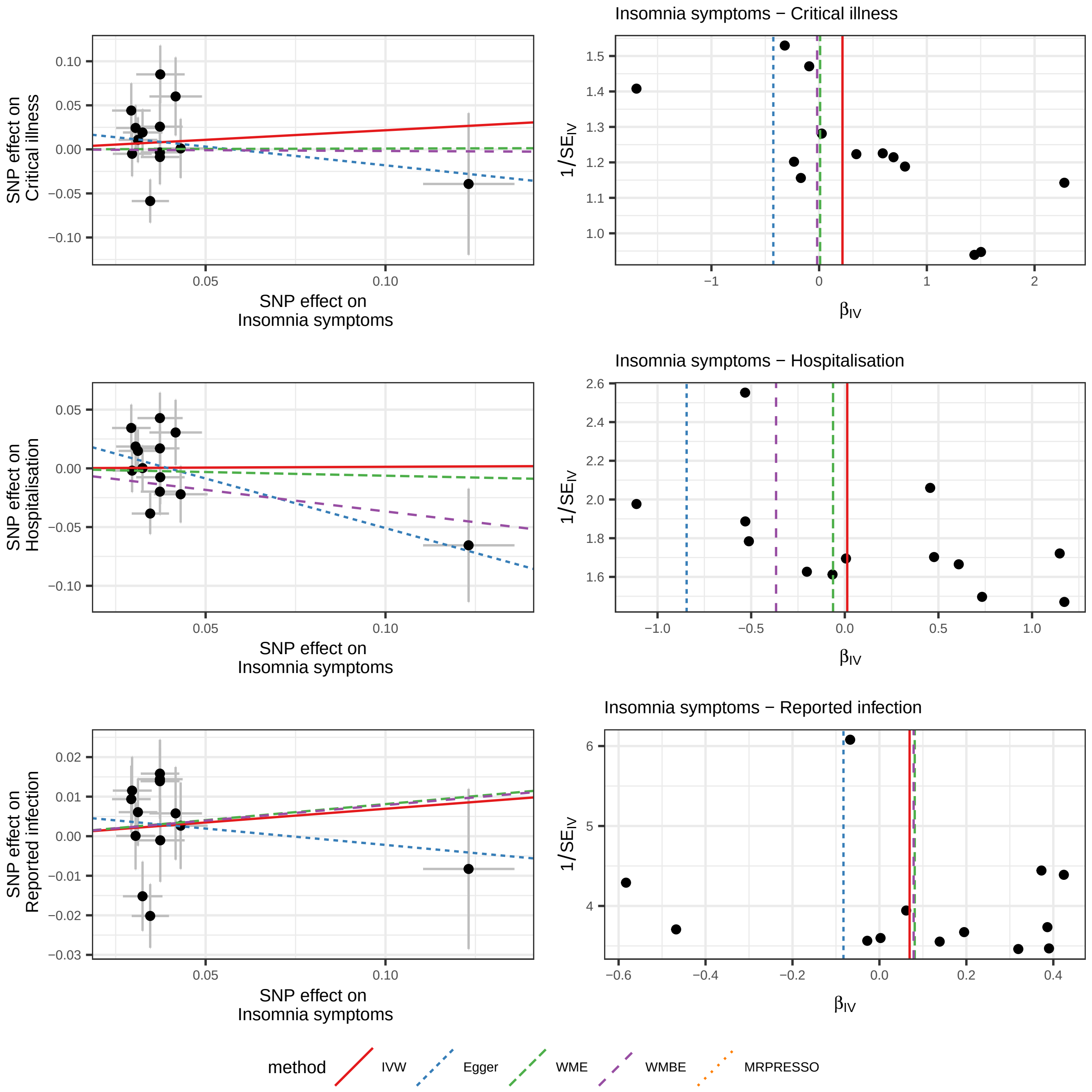
Insomnia symptoms − Critical illness
SNP effect on Critical illness
SNP effect on Insomnia symptoms
1/SE$_{IV}$
β$_{IV}$
Insomnia symptoms − Hospitalisation
SNP effect on Hospitalisation
SNP effect on Insomnia symptoms
1/SE$_{IV}$
β$_{IV}$
Insomnia symptoms − Reported infection
SNP effect on Reported infection
SNP effect on Insomnia symptoms
1/SE$_{IV}$
β$_{IV}$
method
IVW
Egger
WME
WMBE
MRPRESSO

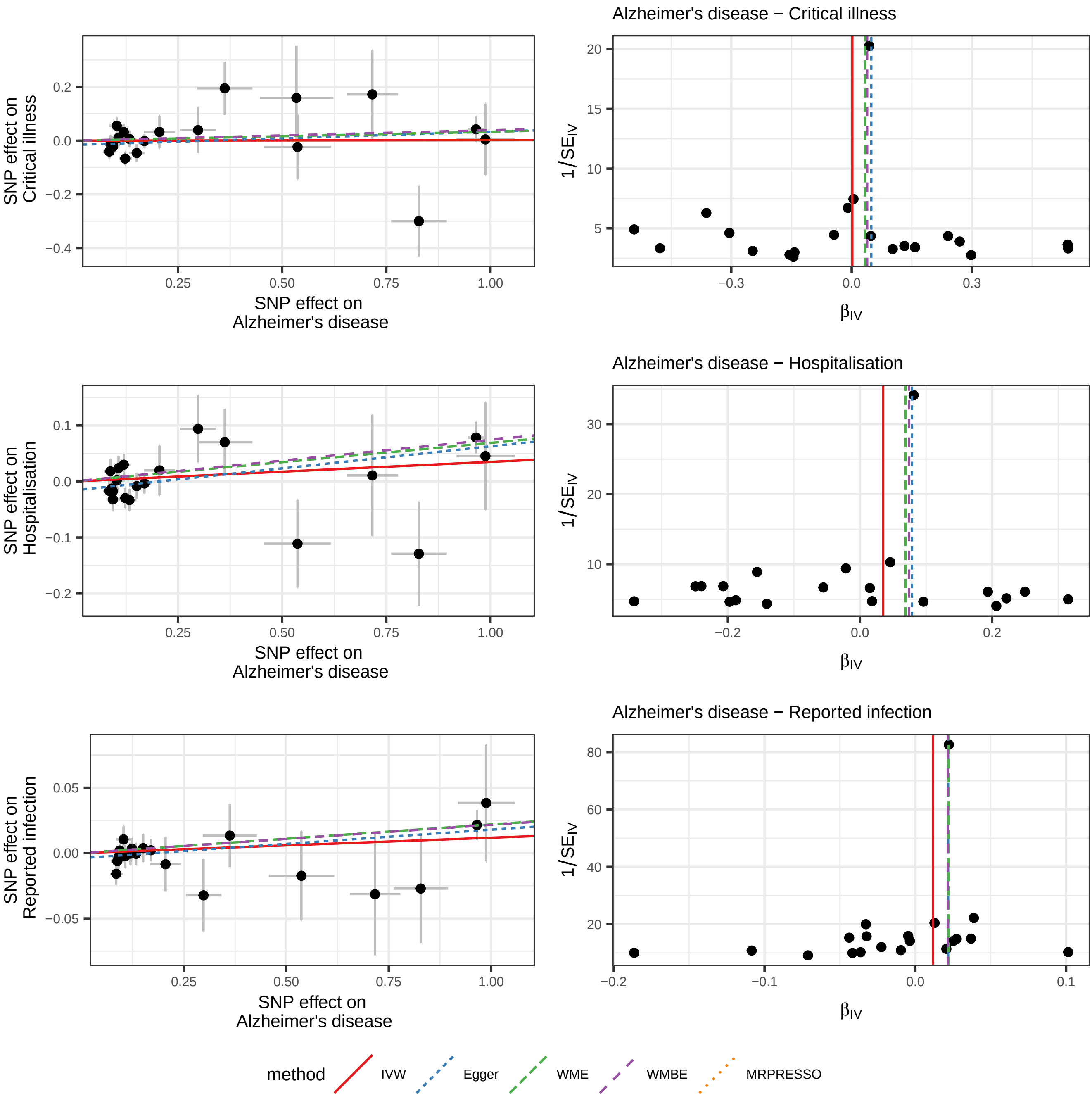

Alzheimer's disease − Critical illness
Alzheimer's disease − Hospitalisation
Alzheimer's disease − Reported infection
SNP effect on Critical illness
SNP effect on Hospitalisation
SNP effect on Reported infection
SNP effect on Alzheimer's disease
$1/SE_{IV}$
β_{IV}
method
IVW
Egger
WME
WMBE
MRPRESSO

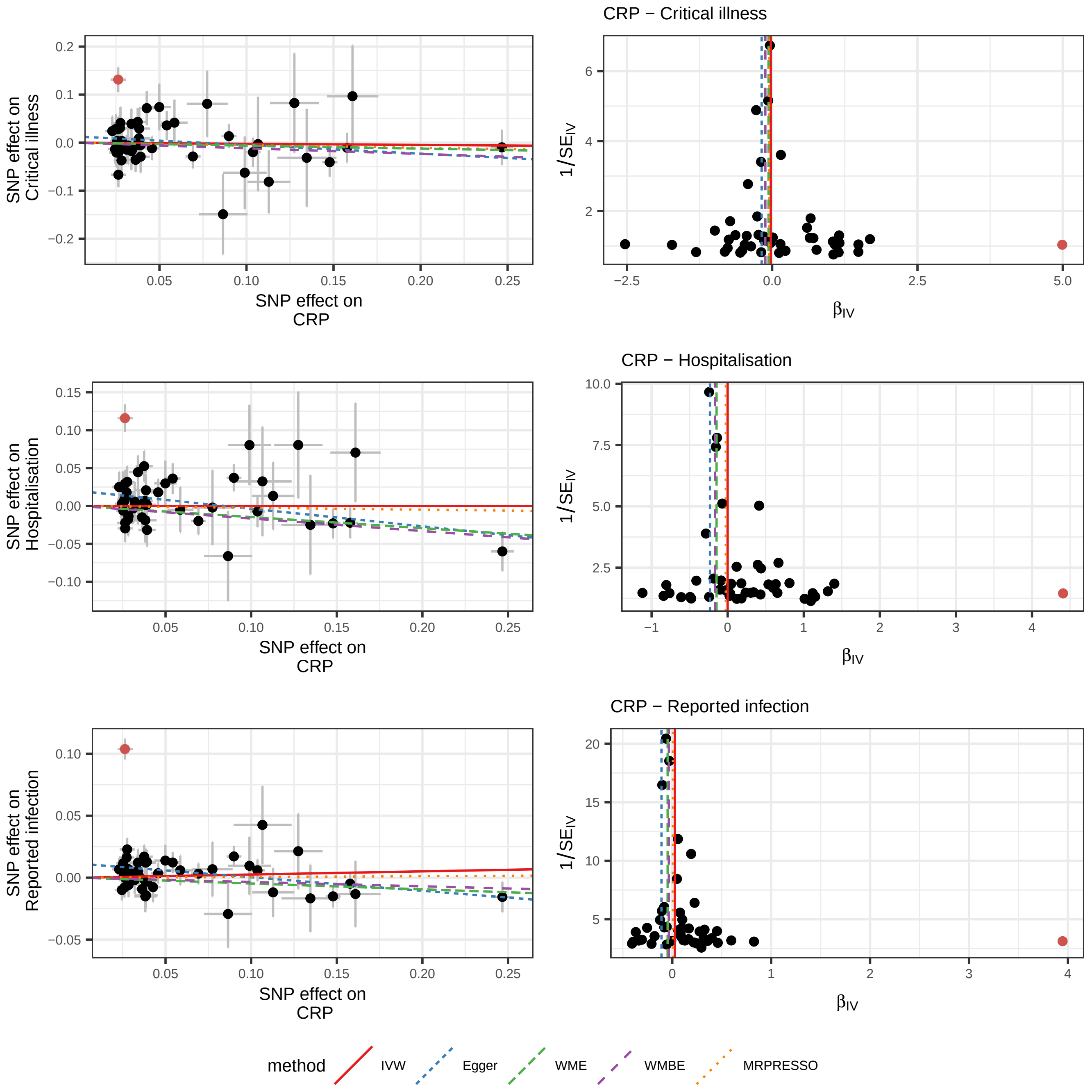

CRP − Critical illness
SNP effect on Critical illness
SNP effect on CRP
1/SE_IV
β_IV
CRP − Hospitalisation
SNP effect on Hospitalisation
SNP effect on CRP
1/SE_IV
β_IV
CRP − Reported infection
SNP effect on Reported infection
SNP effect on CRP
1/SE_IV
β_IV
method
IVW
Egger
WME
WMBE
MRPRESSO

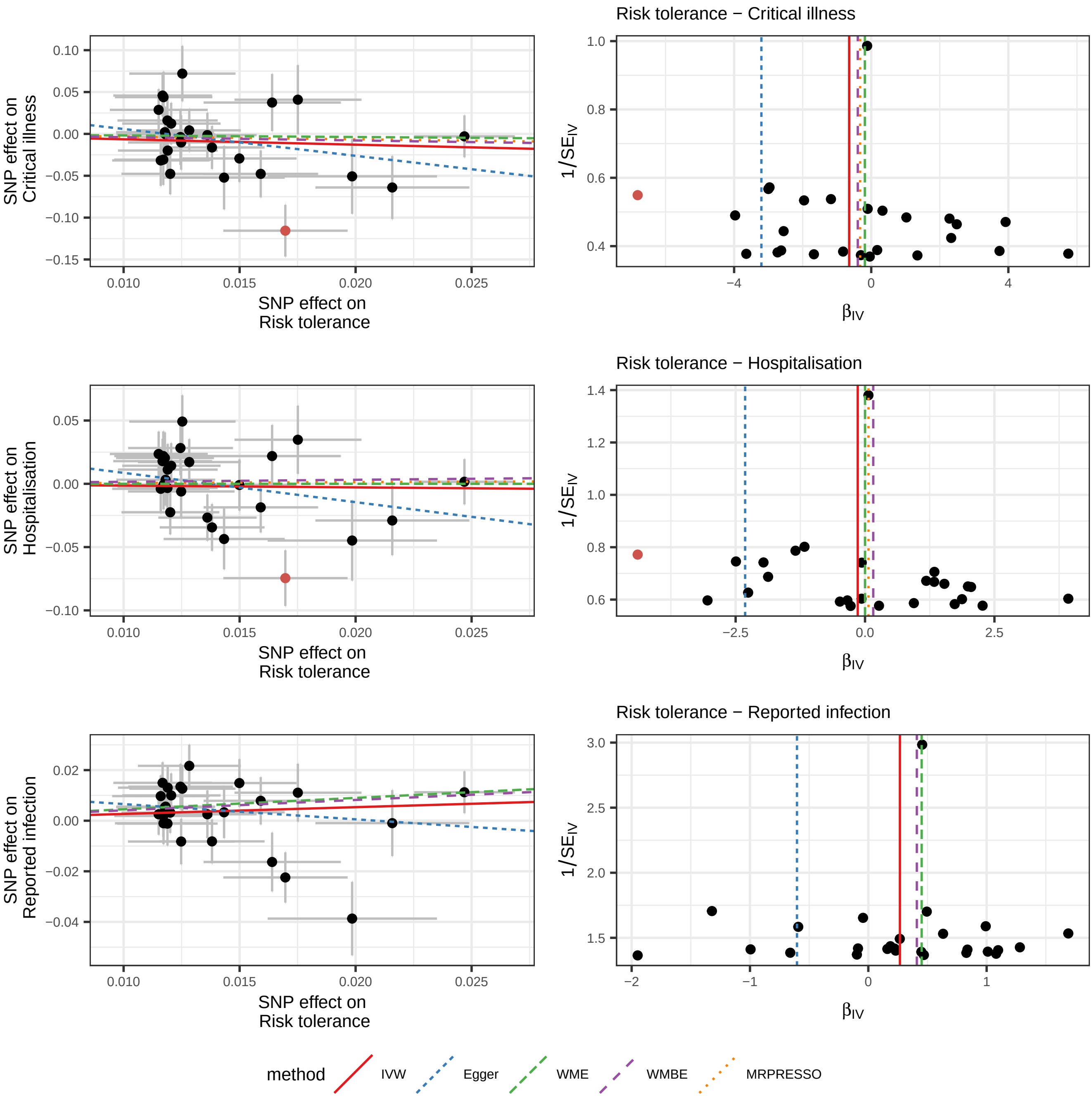

Risk tolerance − Critical illness
Risk tolerance − Hospitalisation
Risk tolerance − Reported infection
SNP effect on Critical illness
SNP effect on Hospitalisation
SNP effect on Reported infection
SNP effect on Risk tolerance
1/SE_IV
β_IV
method
IVW
Egger
WME
WMBE
MRPRESSO

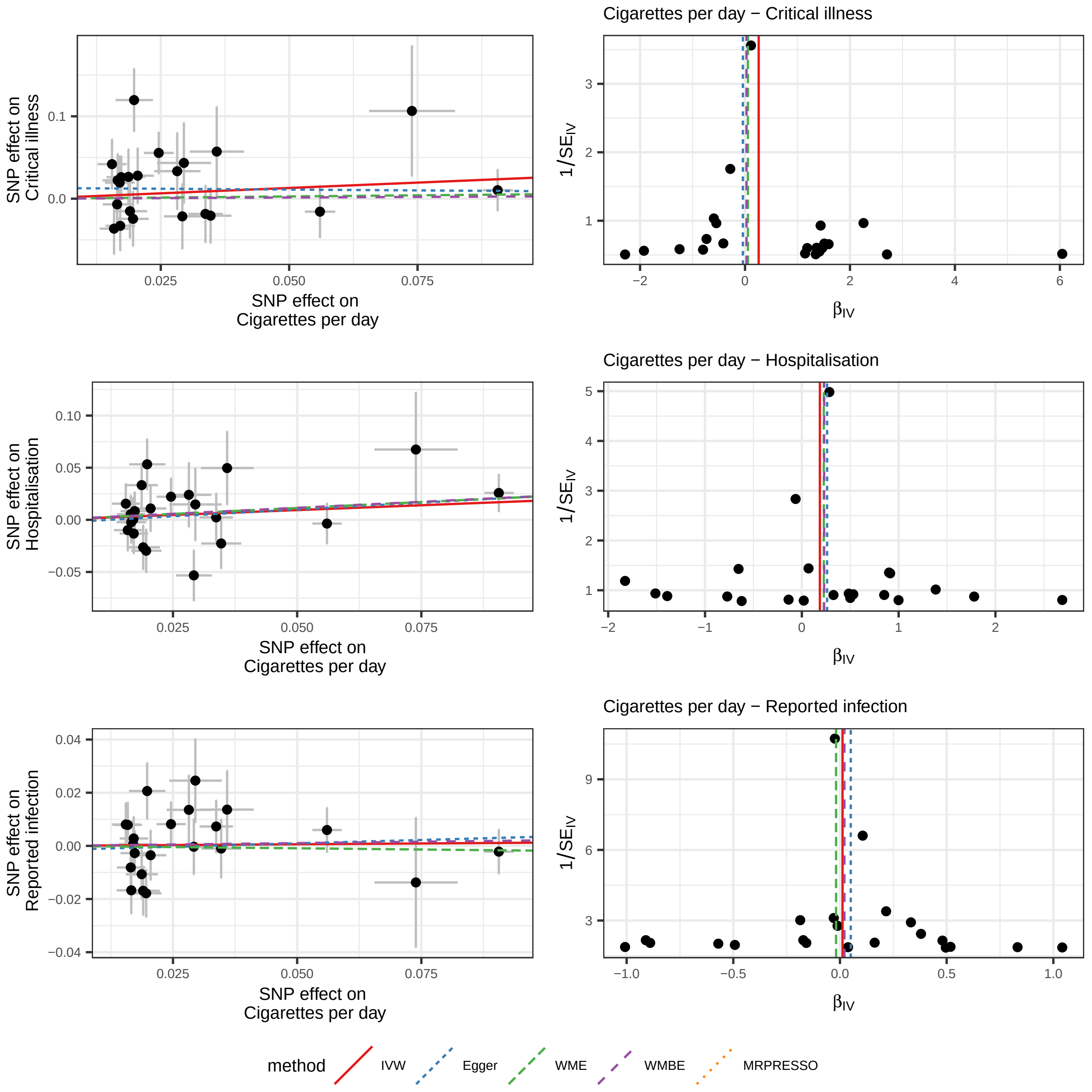

Cigarettes per day − Critical illness
Cigarettes per day − Hospitalisation
Cigarettes per day − Reported infection
SNP effect on Critical illness
SNP effect on Hospitalisation
SNP effect on Reported infection
SNP effect on Cigarettes per day
$1/SE_{IV}$
β_{IV}
method
IVW
Egger
WME
WMBE
MRPRESSO

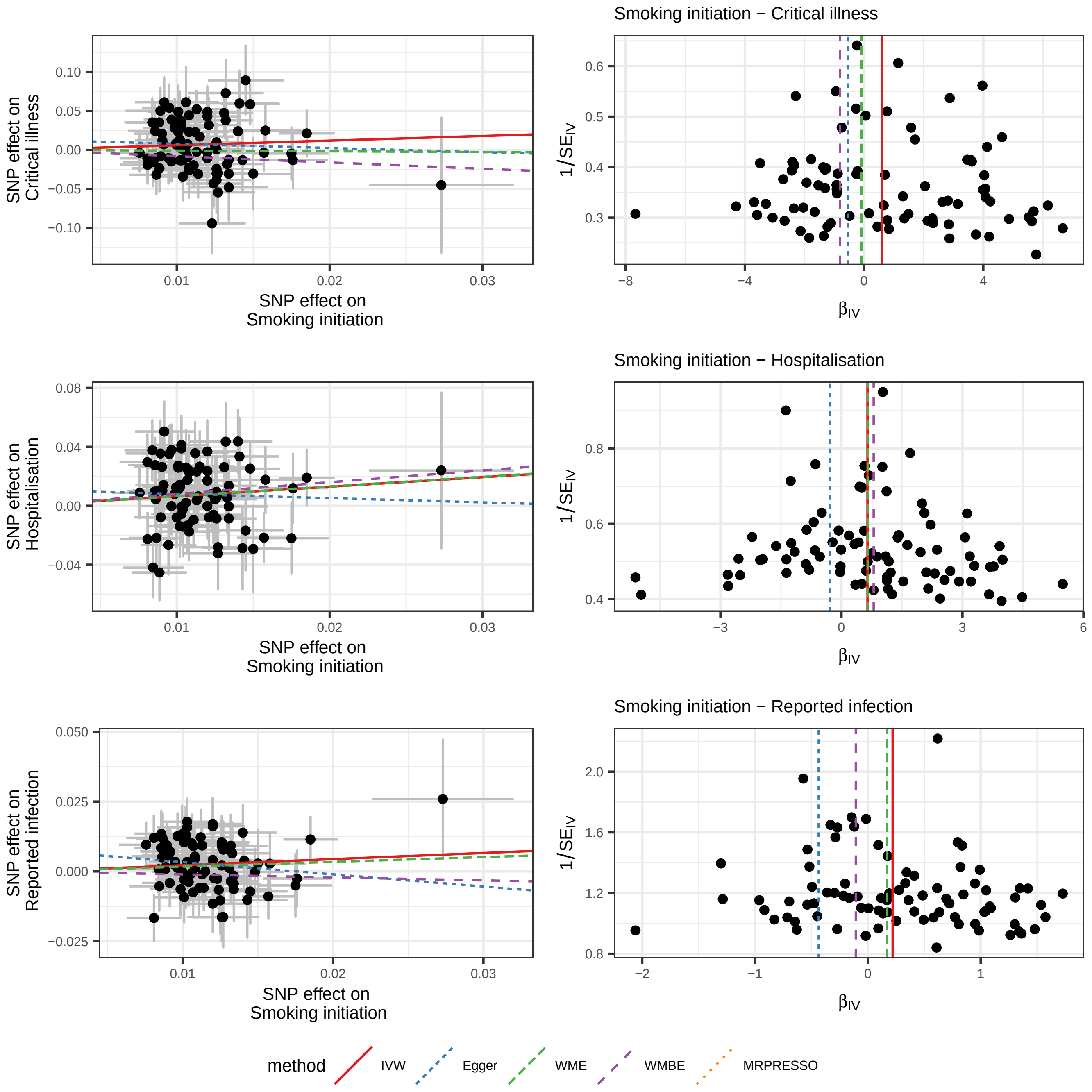

Smoking initiation − Critical illness
Smoking initiation − Hospitalisation
Smoking initiation − Reported infection
SNP effect on Critical illness
SNP effect on Hospitalisation
SNP effect on Reported infection
SNP effect on Smoking initiation
1/SE$_{IV}$
β$_{IV}$
method
IVW
Egger
WME
WMBE
MRPRESSO

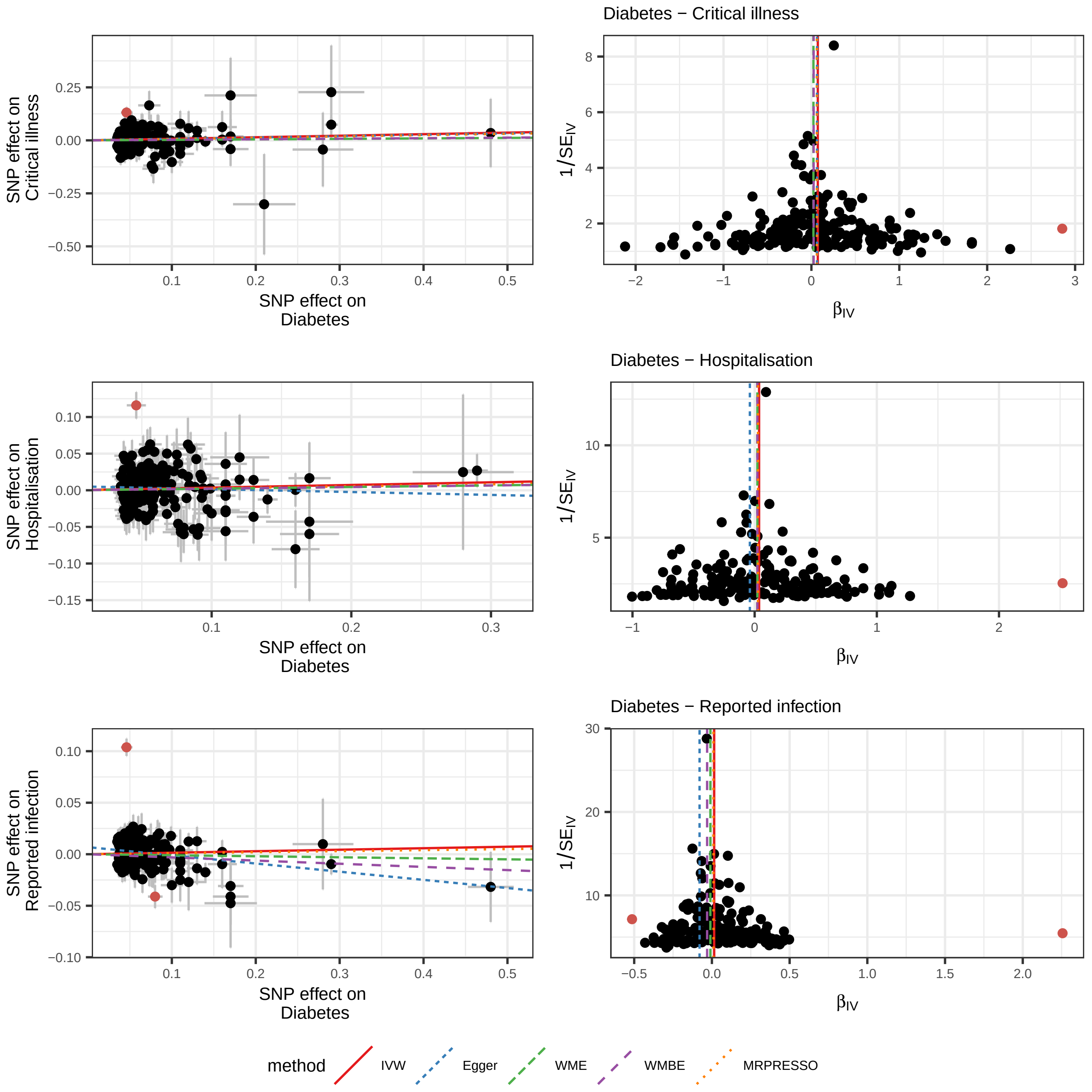

Diabetes − Critical illness
Diabetes − Hospitalisation
Diabetes − Reported infection
SNP effect on Critical illness
SNP effect on Hospitalisation
SNP effect on Reported infection
SNP effect on Diabetes
$1/SE_{IV}$
β_{IV}
method
IVW
Egger
WME
WMBE
MRPRESSO

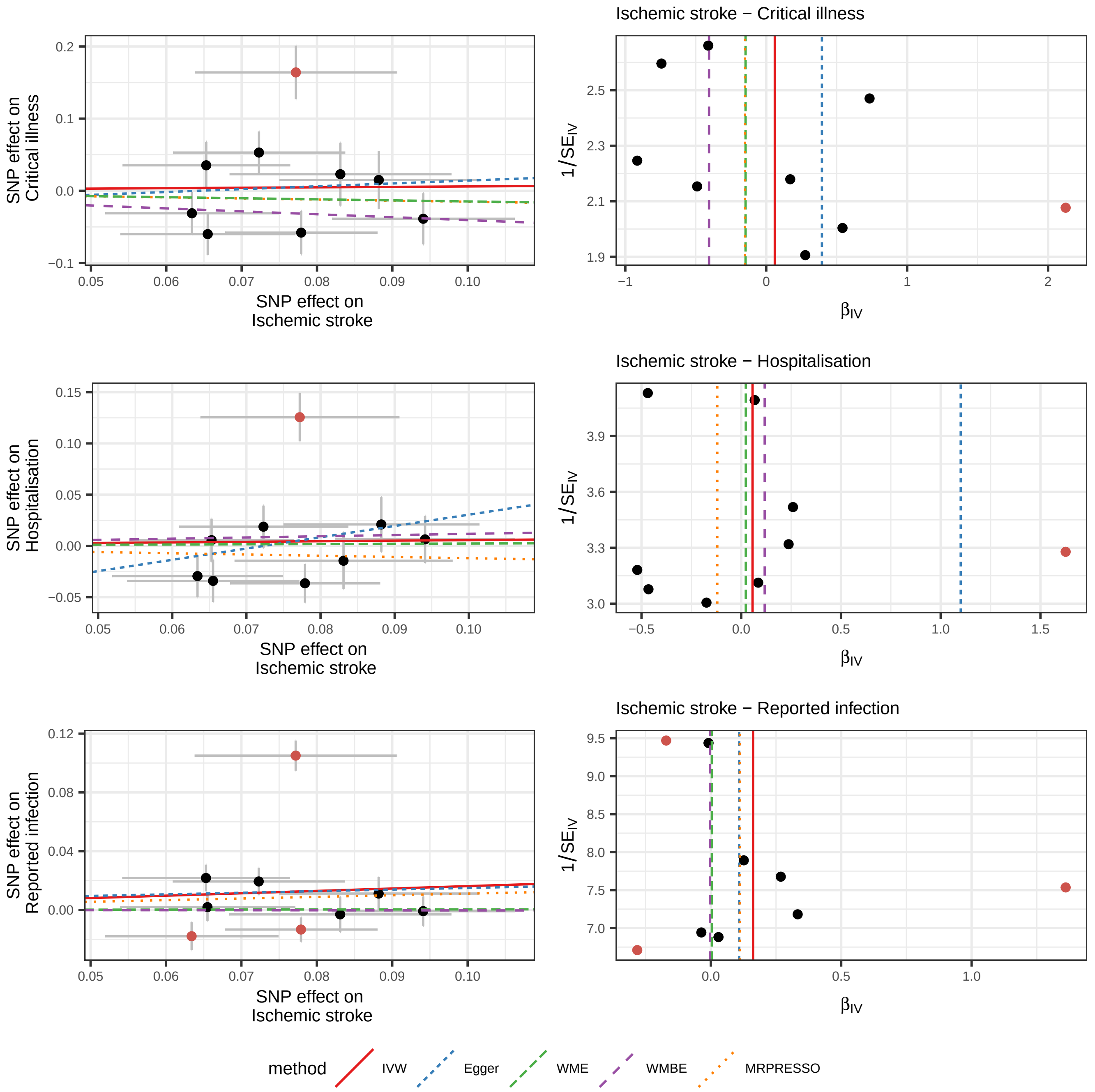

Ischemic stroke − Critical illness
SNP effect on Critical illness
SNP effect on Ischemic stroke
1/SE_IV
β_IV
Ischemic stroke − Hospitalisation
SNP effect on Hospitalisation
SNP effect on Ischemic stroke
1/SE_IV
β_IV
Ischemic stroke − Reported infection
SNP effect on Reported infection
SNP effect on Ischemic stroke
1/SE_IV
β_IV
method
IVW
Egger
WME
WMBE
MRPRESSO

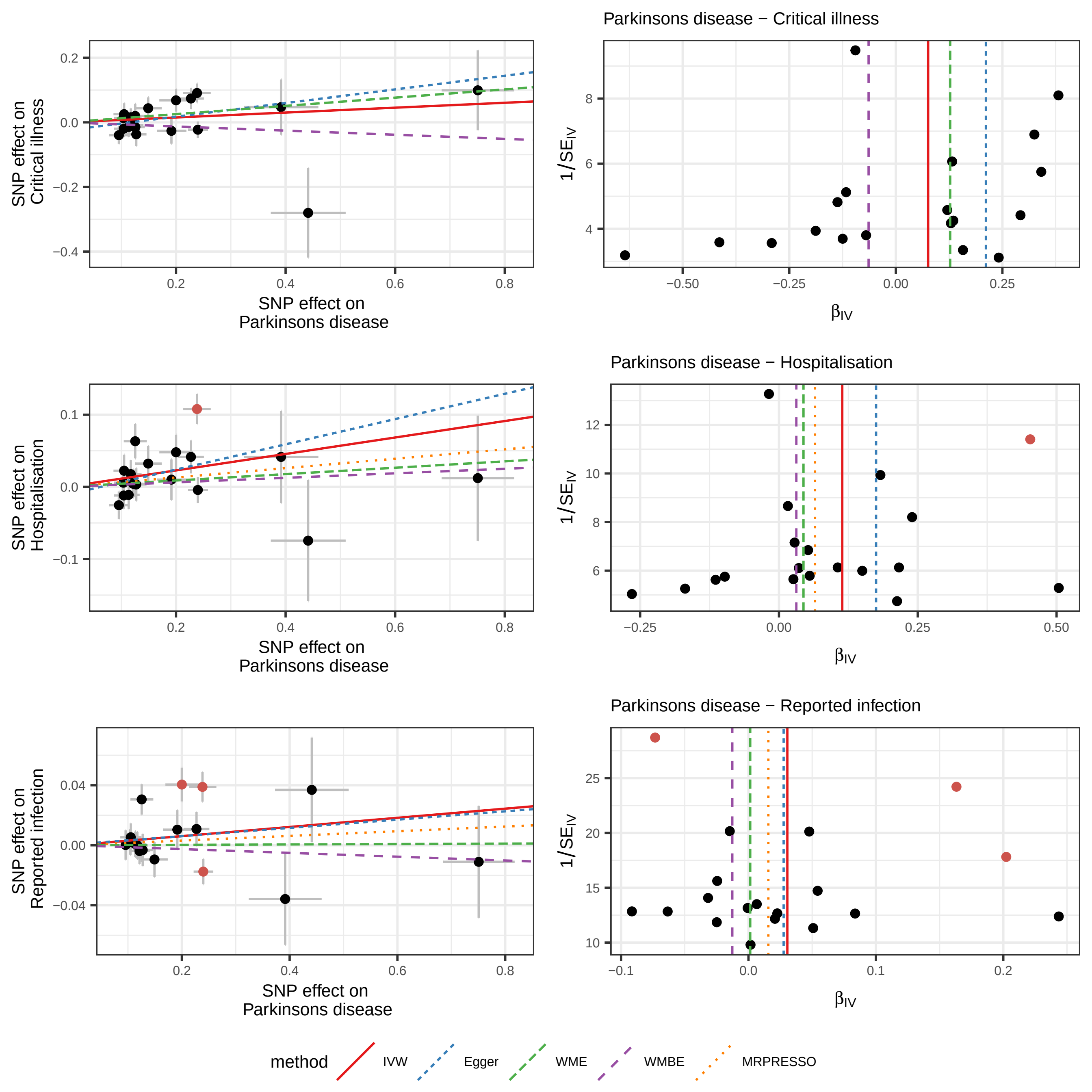

Parkinsons disease − Critical illness
SNP effect on Critical illness
SNP effect on Parkinsons disease
1/SE_IV
β_IV
Parkinsons disease − Hospitalisation
SNP effect on Hospitalisation
SNP effect on Parkinsons disease
1/SE_IV
β_IV
Parkinsons disease − Reported infection
SNP effect on Reported infection
SNP effect on Parkinsons disease
1/SE_IV
β_IV
method
IVW
Egger
WME
WMBE
MRPRESSO

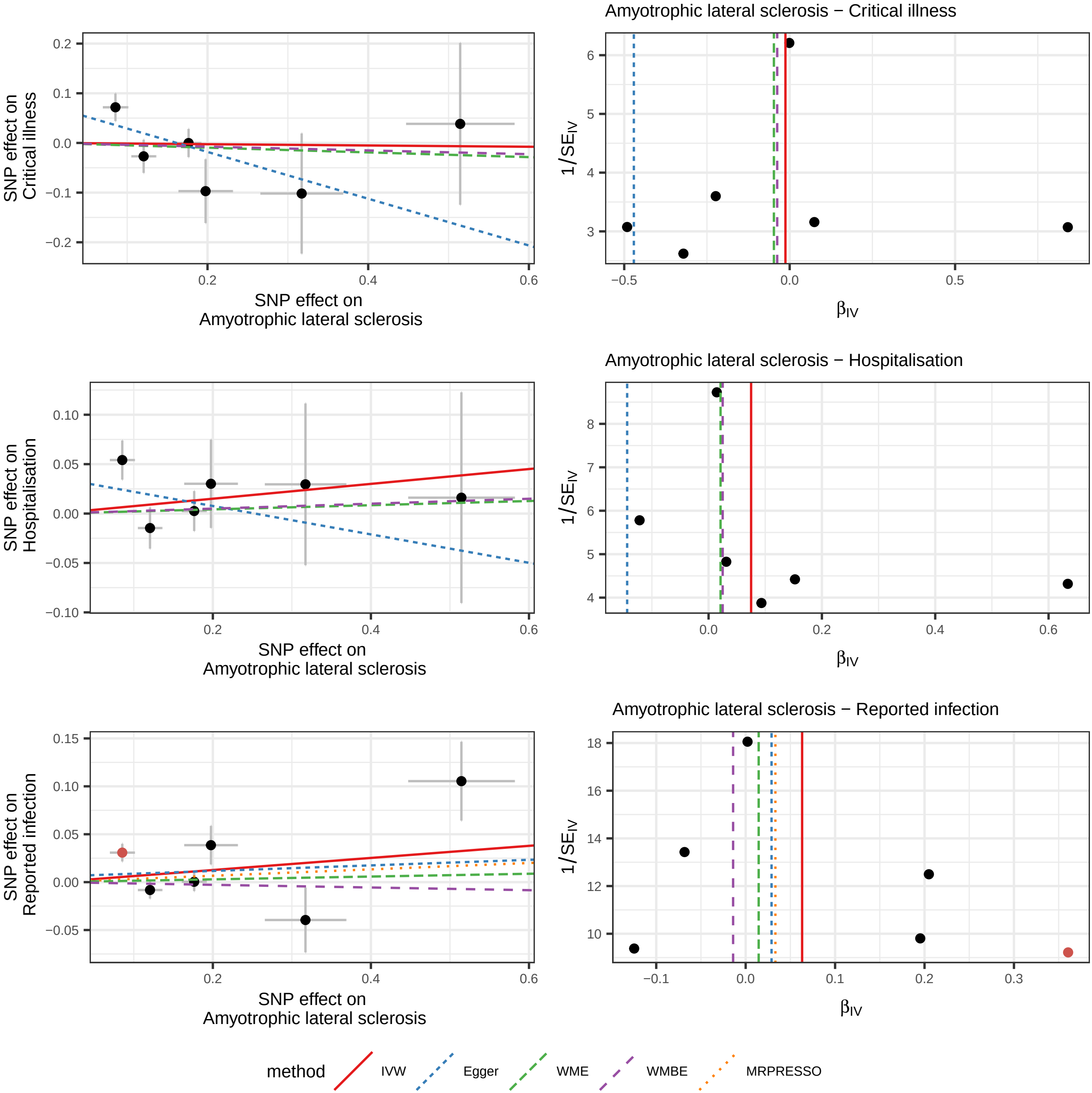

Amyotrophic lateral sclerosis − Critical illness
SNP effect on Critical illness
SNP effect on Amyotrophic lateral sclerosis
1/SE_IV
β_IV
Amyotrophic lateral sclerosis − Hospitalisation
SNP effect on Hospitalisation
SNP effect on Amyotrophic lateral sclerosis
1/SE_IV
β_IV
Amyotrophic lateral sclerosis − Reported infection
SNP effect on Reported infection
SNP effect on Amyotrophic lateral sclerosis
1/SE_IV
β_IV
method
IVW
Egger
WME
WMBE
MRPRESSO

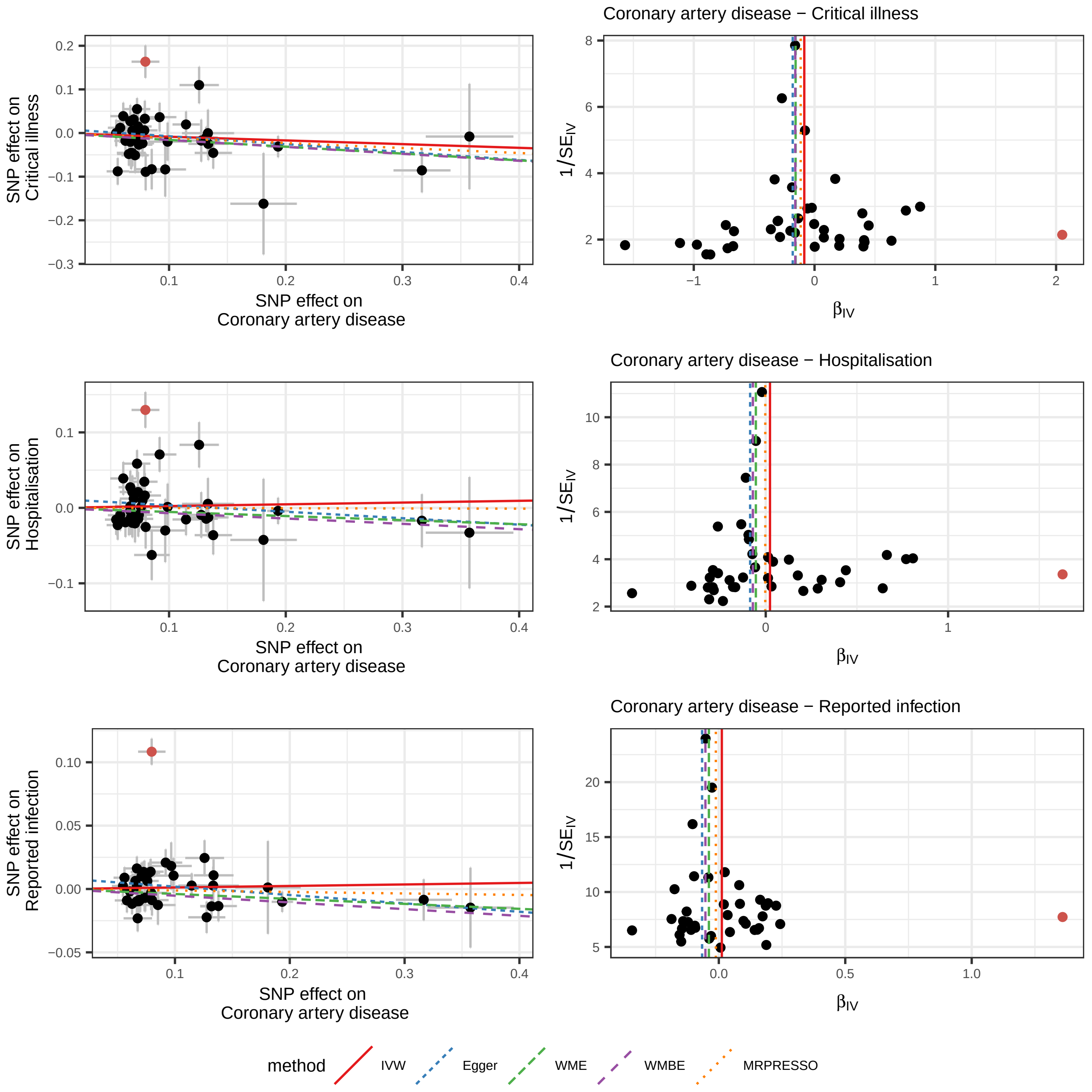

Coronary artery disease − Critical illness
Coronary artery disease − Hospitalisation
Coronary artery disease − Reported infection
SNP effect on Critical illness
SNP effect on Hospitalisation
SNP effect on Reported infection
SNP effect on Coronary artery disease
$1/SE_{IV}$
β_{IV}
method
IVW
Egger
WME
WMBE
MRPRESSO

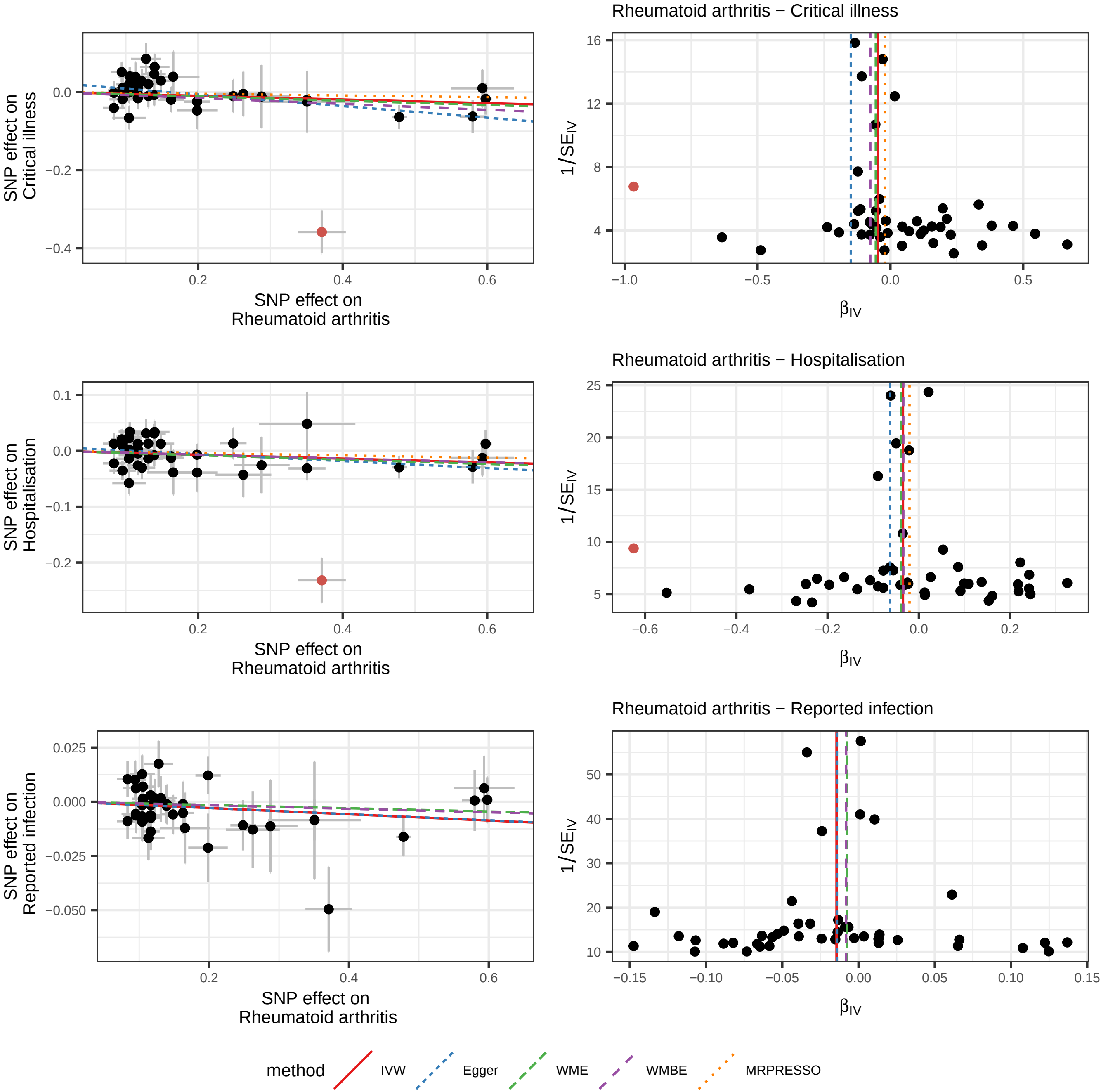
Rheumatoid arthritis − Critical illness
Rheumatoid arthritis − Hospitalisation
Rheumatoid arthritis − Reported infection
SNP effect on Critical illness
SNP effect on Hospitalisation
SNP effect on Reported infection
SNP effect on Rheumatoid arthritis
$1/SE_{IV}$
β_{IV}
method
IVW
Egger
WME
WMBE
MRPRESSO

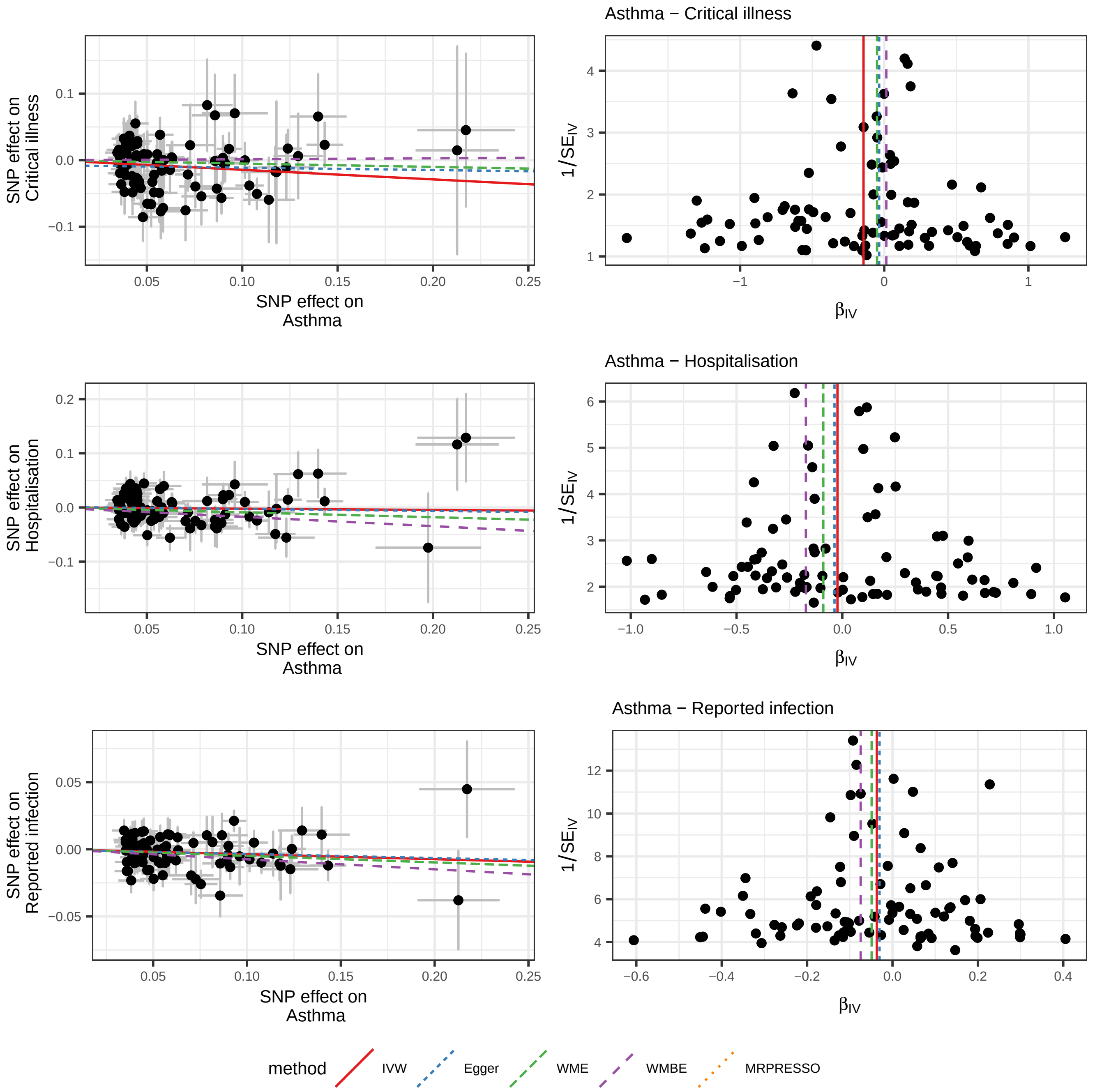

Asthma − Critical illness
SNP effect on Critical illness
SNP effect on Asthma
1/SE_IV
β_IV
Asthma − Hospitalisation
SNP effect on Hospitalisation
SNP effect on Asthma
1/SE_IV
β_IV
Asthma − Reported infection
SNP effect on Reported infection
SNP effect on Asthma
1/SE_IV
β_IV
method
IVW
Egger
WME
WMBE
MRPRESSO

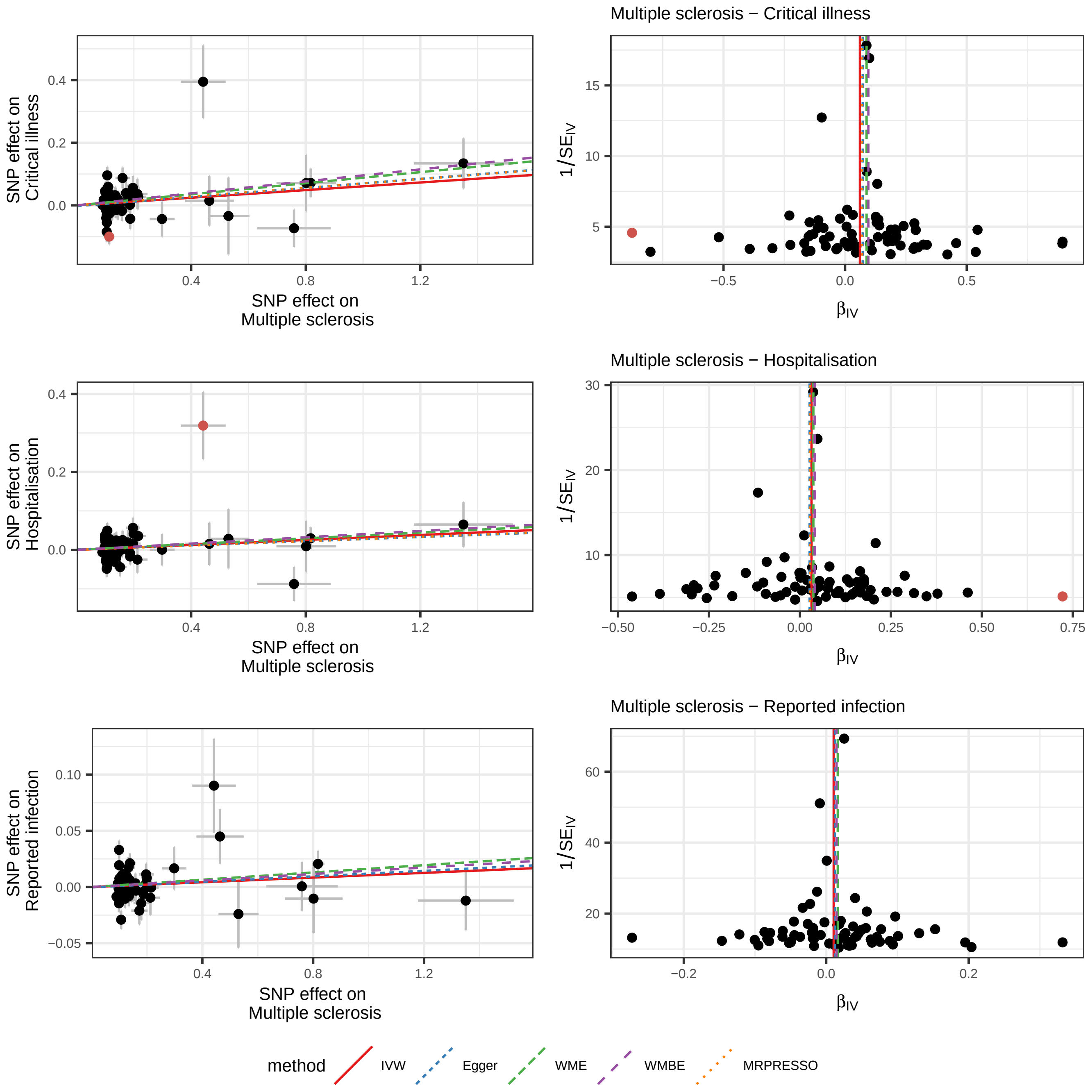
Multiple sclerosis − Critical illness
Multiple sclerosis − Hospitalisation
Multiple sclerosis − Reported infection
SNP effect on Critical illness
SNP effect on Hospitalisation
SNP effect on Reported infection
SNP effect on Multiple sclerosis
$1/SE_{IV}$
β_{IV}
method
IVW
Egger
WME
WMBE
MRPRESSO

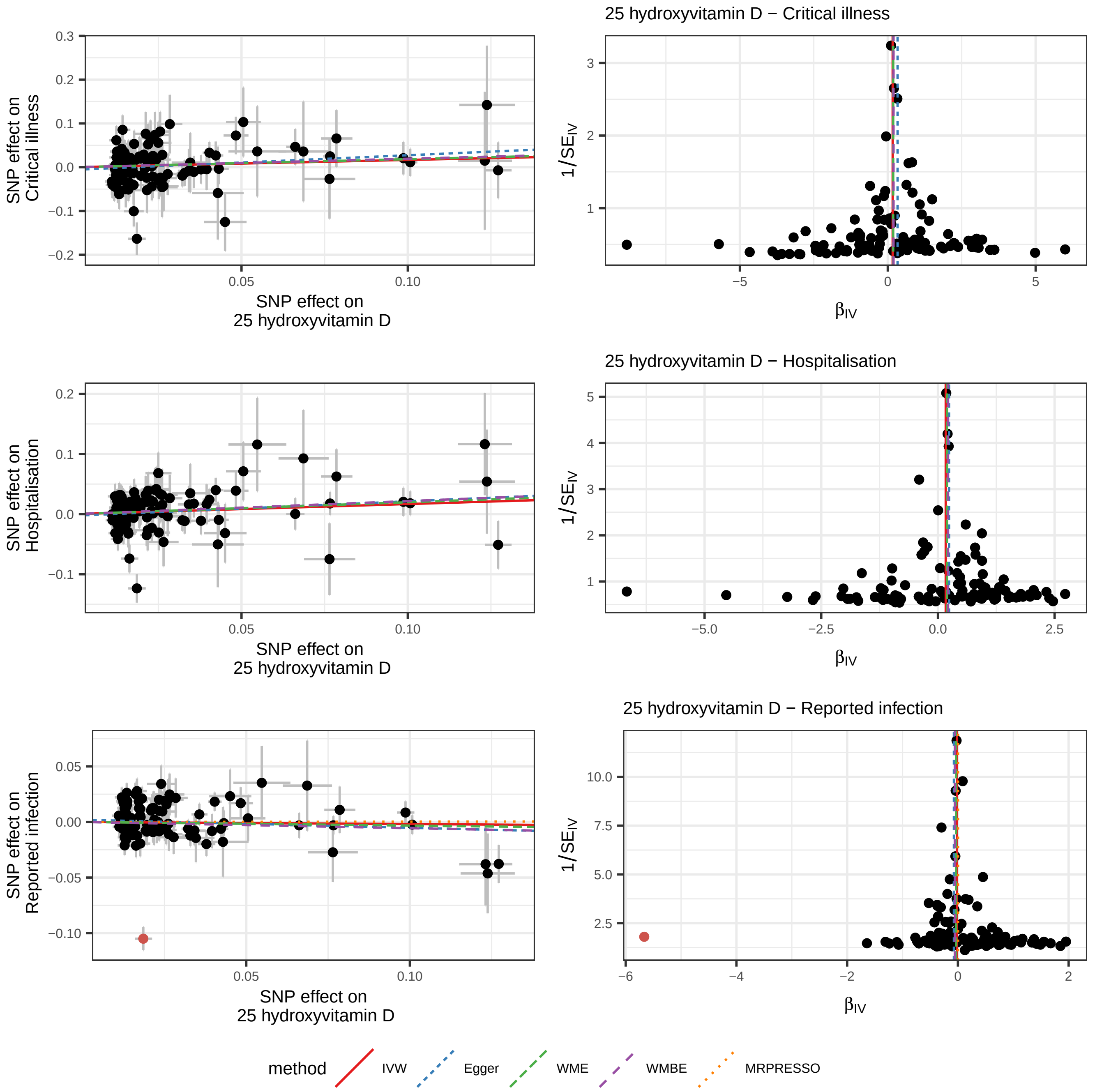
25 hydroxyvitamin D − Critical illness
25 hydroxyvitamin D − Hospitalisation
25 hydroxyvitamin D − Reported infection
SNP effect on Critical illness
SNP effect on Hospitalisation
SNP effect on Reported infection
SNP effect on 25 hydroxyvitamin D
1/SE_IV
β_IV
method
IVW
Egger
WME
WMBE
MRPRESSO

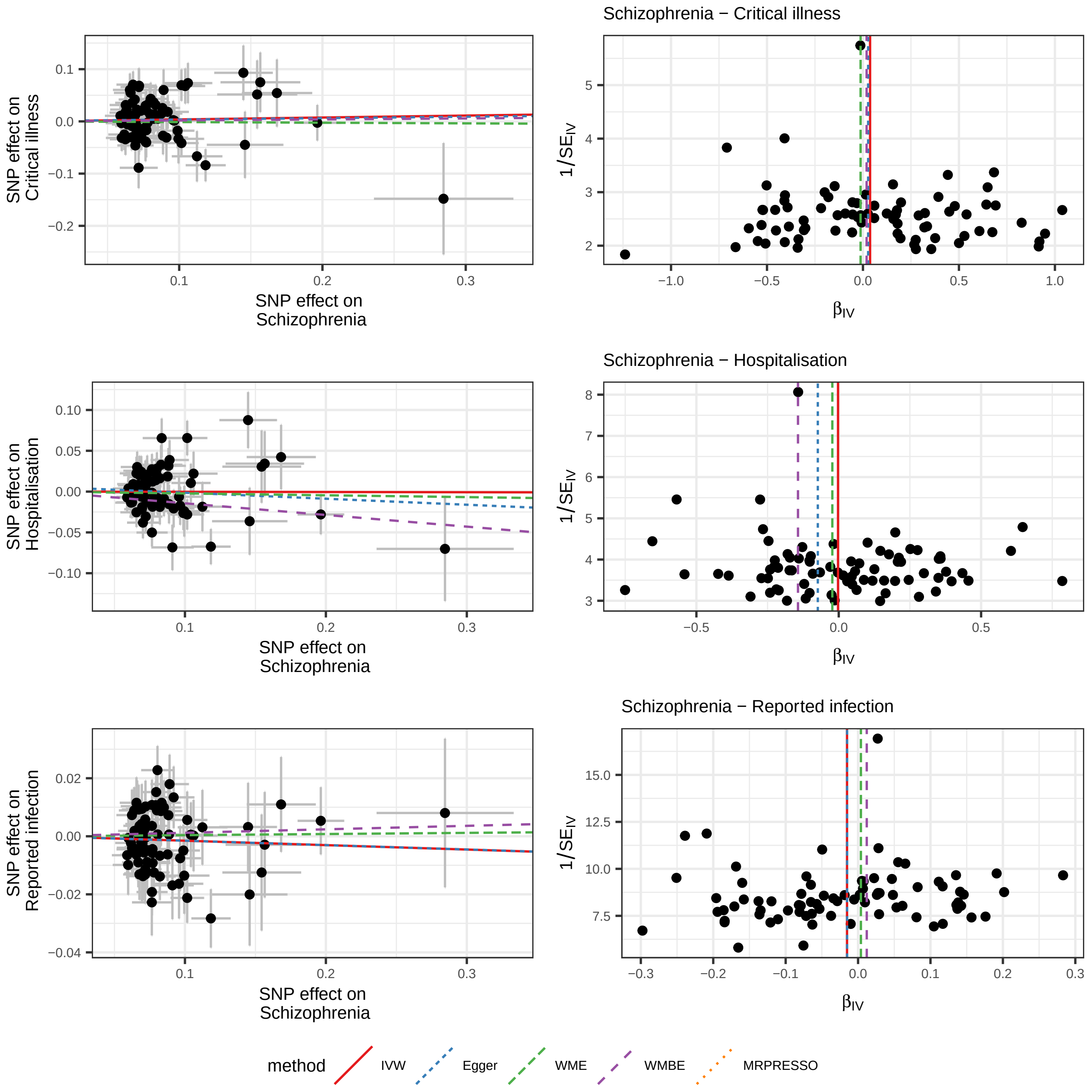

Schizophrenia − Critical illness
Schizophrenia − Hospitalisation
Schizophrenia − Reported infection
SNP effect on Critical illness
SNP effect on Hospitalisation
SNP effect on Reported infection
SNP effect on Schizophrenia
$1/SE_{IV}$
β_{IV}
method
IVW
Egger
WME
WMBE
MRPRESSO

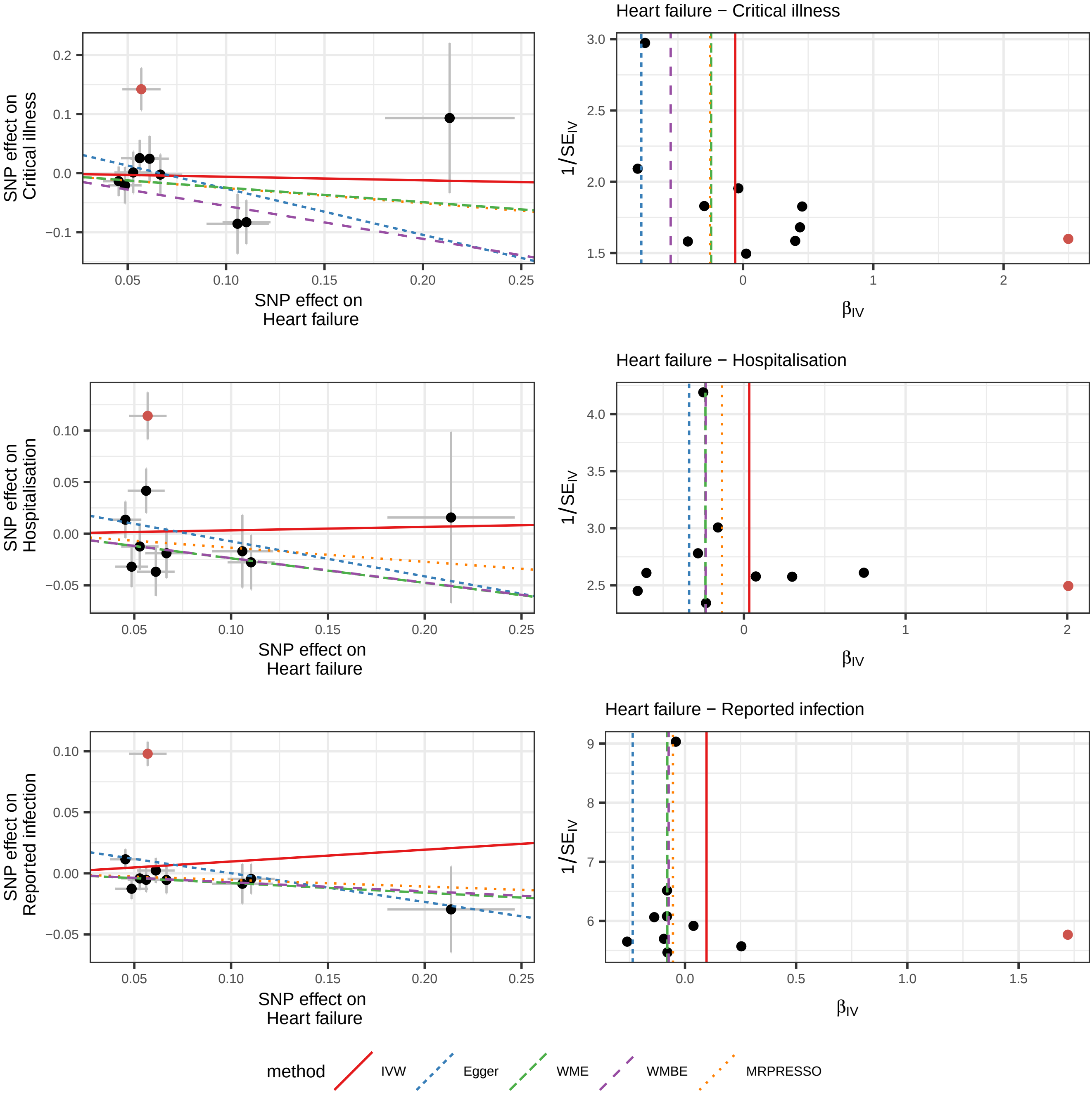
Heart failure − Critical illness
Heart failure − Hospitalisation
Heart failure − Reported infection
SNP effect on Critical illness
SNP effect on Hospitalisation
SNP effect on Reported infection
SNP effect on Heart failure
1/SE_IV
β_IV
method
IVW
Egger
WME
WMBE
MRPRESSO

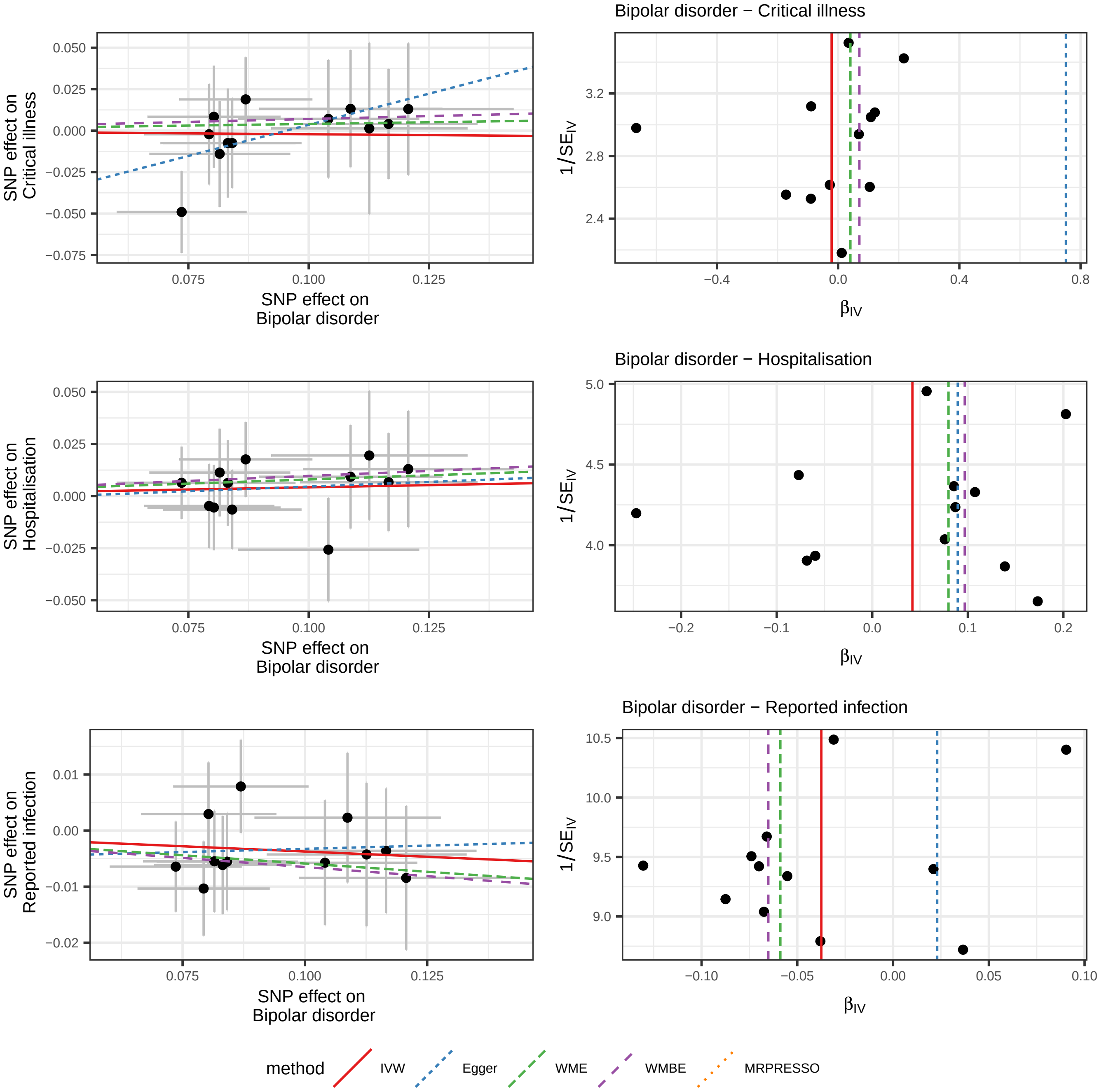
Bipolar disorder − Critical illness
SNP effect on Critical illness
SNP effect on Bipolar disorder
1/SE_IV
β_IV
Bipolar disorder − Hospitalisation
SNP effect on Hospitalisation
SNP effect on Bipolar disorder
1/SE_IV
β_IV
Bipolar disorder − Reported infection
SNP effect on Reported infection
SNP effect on Bipolar disorder
1/SE_IV
β_IV
method
IVW
Egger
WME
WMBE
MRPRESSO

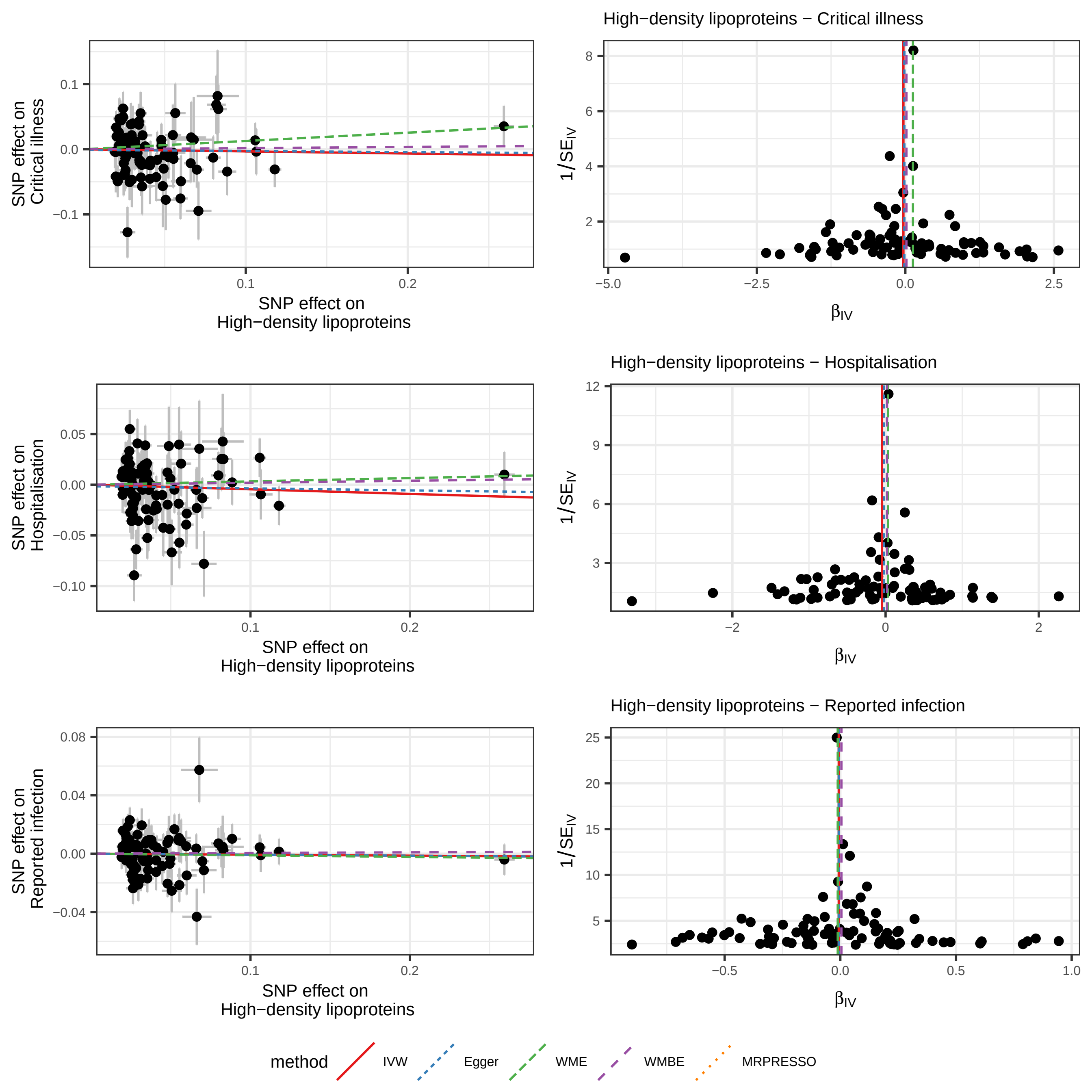
High−density lipoproteins − Critical illness
SNP effect on Critical illness
SNP effect on High−density lipoproteins
$1/SE_{IV}$
β_{IV}
High−density lipoproteins − Hospitalisation
SNP effect on Hospitalisation
High−density lipoproteins − Reported infection
SNP effect on Reported infection
method
IVW
Egger
WME
WMBE
MRPRESSO

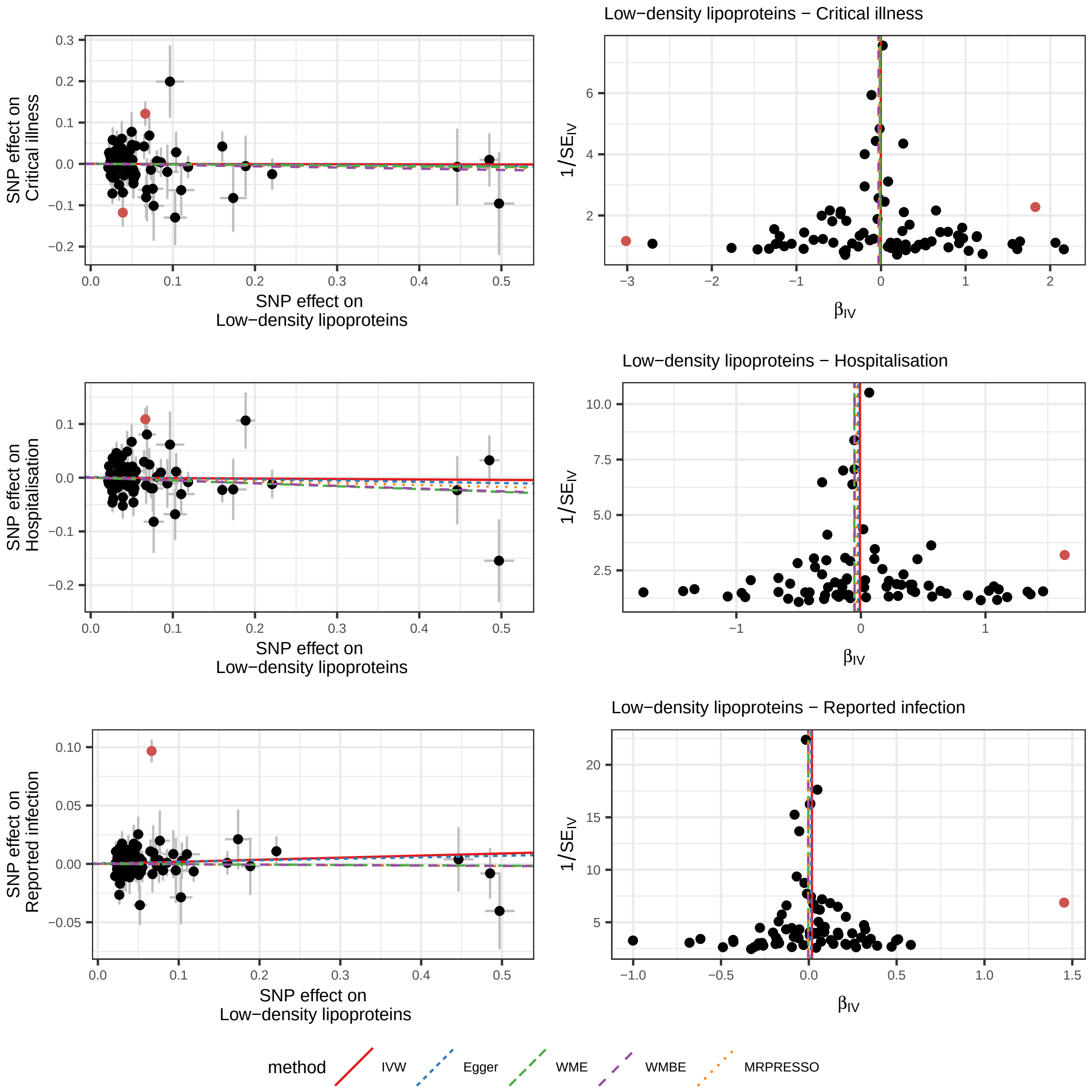

Low−density lipoproteins − Critical illness
Low−density lipoproteins − Hospitalisation
Low−density lipoproteins − Reported infection
SNP effect on Critical illness
SNP effect on Hospitalisation
SNP effect on Reported infection
SNP effect on Low−density lipoproteins
1/SE_IV
β_IV
method
IVW
Egger
WME
WMBE
MRPRESSO

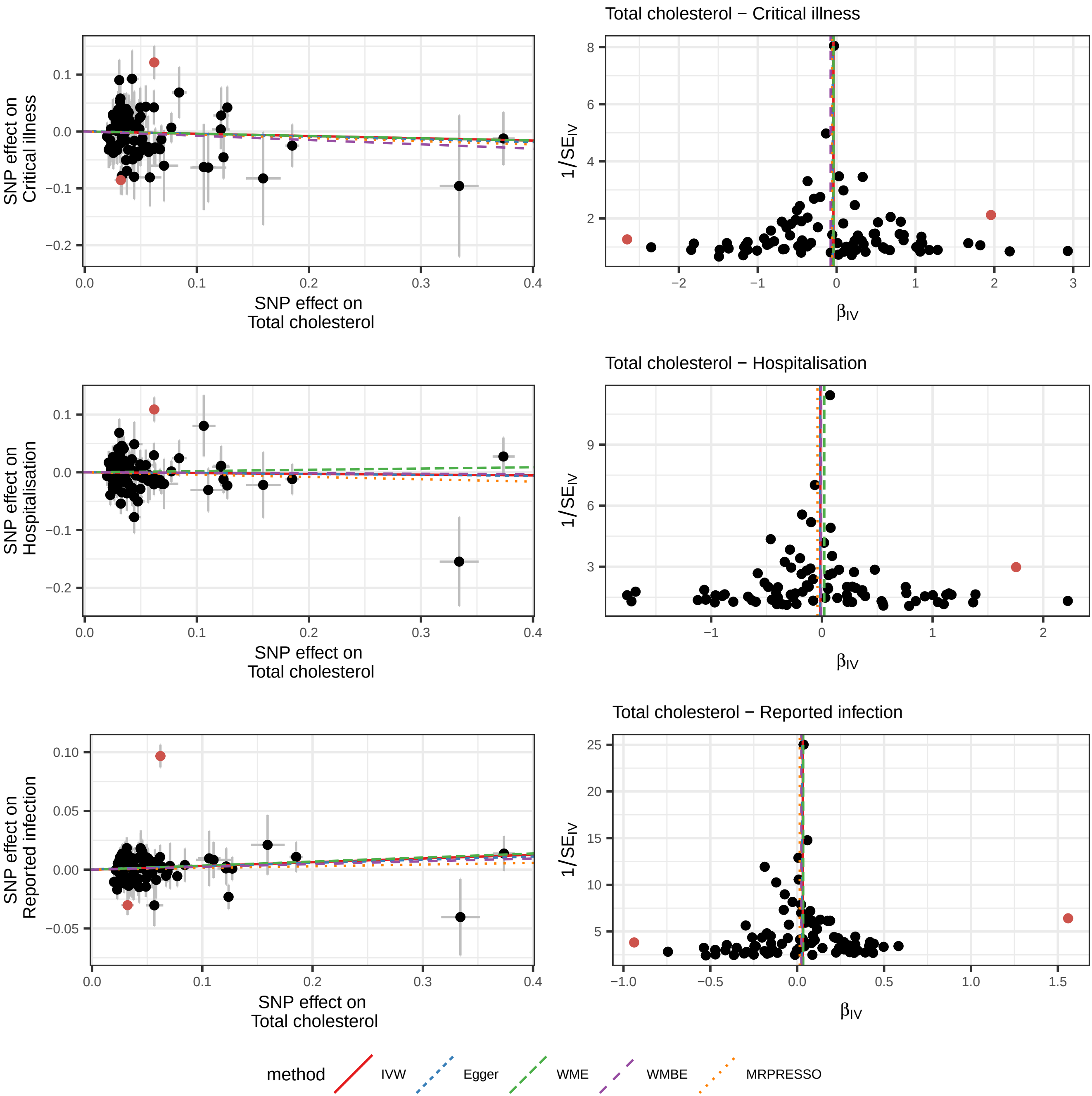
Total cholesterol − Critical illness
Total cholesterol − Hospitalisation
Total cholesterol − Reported infection
SNP effect on Critical illness
SNP effect on Hospitalisation
SNP effect on Reported infection
SNP effect on Total cholesterol
1/SE_IV
β_IV
method
IVW
Egger
WME
WMBE
MRPRESSO

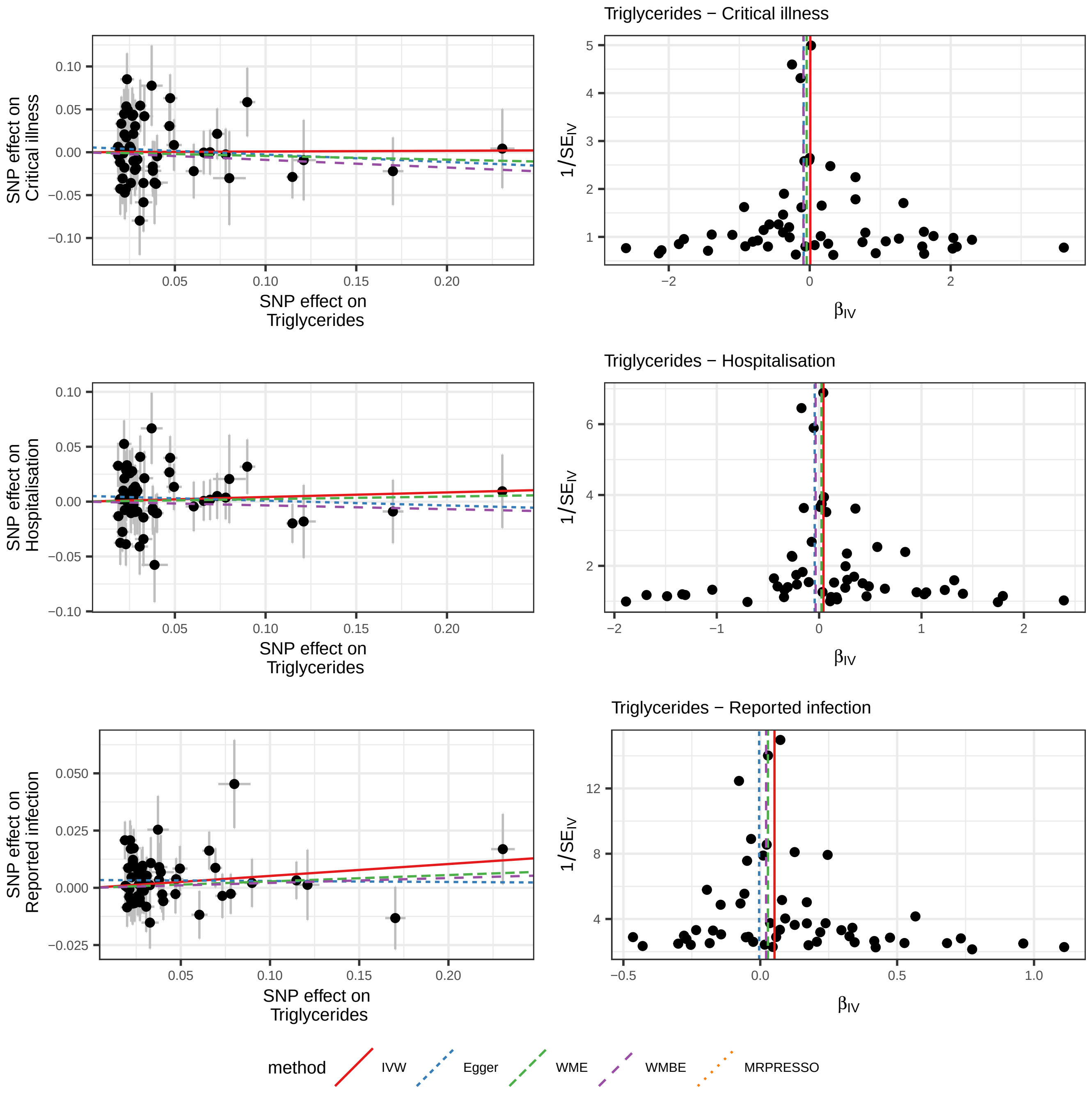

Triglycerides − Critical illness
SNP effect on Critical illness
SNP effect on Triglycerides
$1/SE_{IV}$
β_{IV}
Triglycerides − Hospitalisation
SNP effect on Hospitalisation
SNP effect on Triglycerides
$1/SE_{IV}$
β_{IV}
Triglycerides − Reported infection
SNP effect on Reported infection
SNP effect on Triglycerides
$1/SE_{IV}$
β_{IV}
method
IVW
Egger
WME
WMBE
MRPRESSO

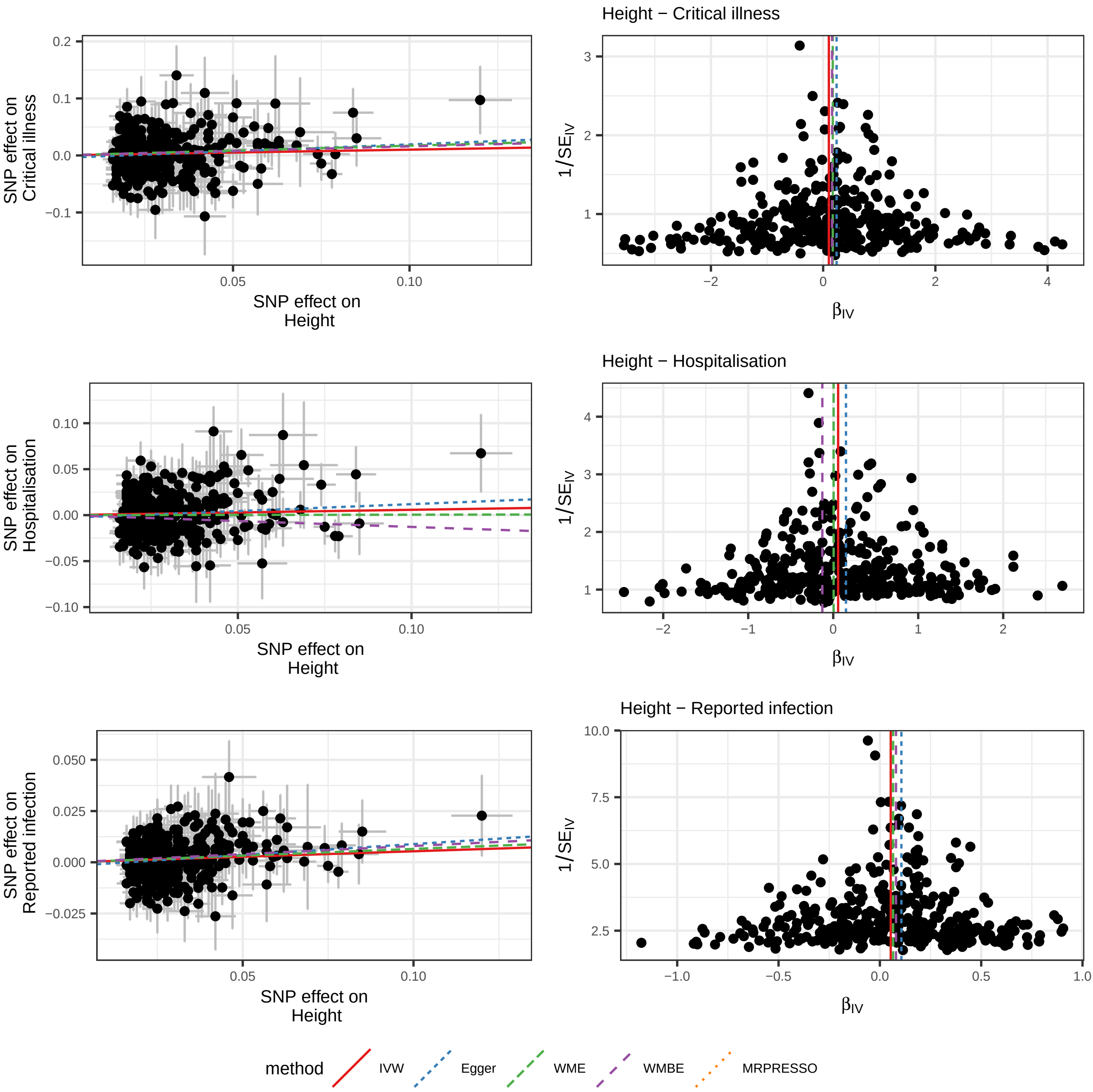
Height − Critical illness
Height − Hospitalisation
Height − Reported infection
SNP effect on Critical illness
SNP effect on Hospitalisation
SNP effect on Reported infection
SNP effect on Height
1/SE_IV
β_IV
method
IVW
Egger
WME
WMBE
MRPRESSO

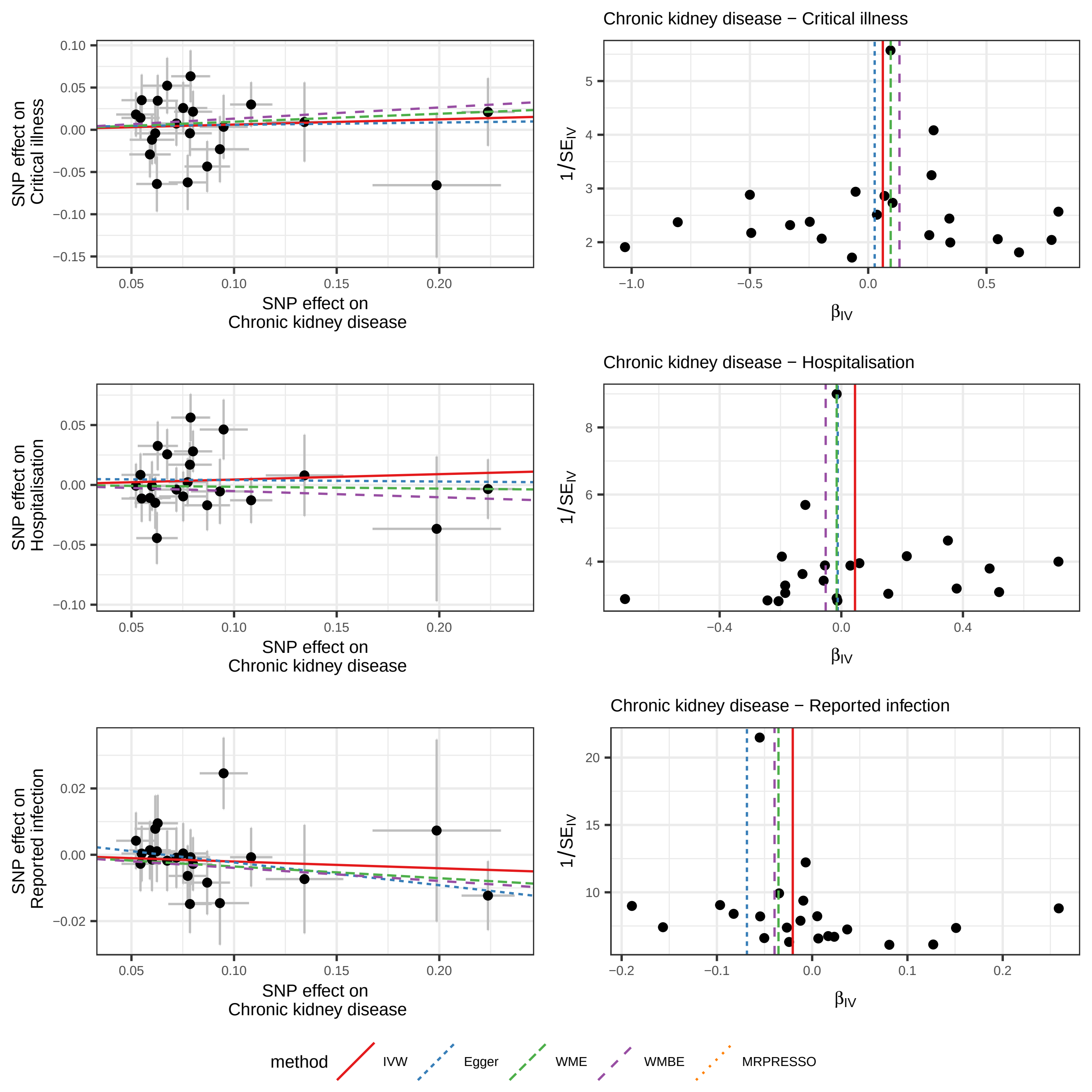

Chronic kidney disease − Critical illness
SNP effect on Critical illness
SNP effect on Chronic kidney disease
$1/SE_{IV}$
β_{IV}
Chronic kidney disease − Hospitalisation
SNP effect on Hospitalisation
SNP effect on Chronic kidney disease
Chronic kidney disease − Reported infection
SNP effect on Reported infection
SNP effect on Chronic kidney disease
method
IVW
Egger
WME
WMBE
MRPRESSO

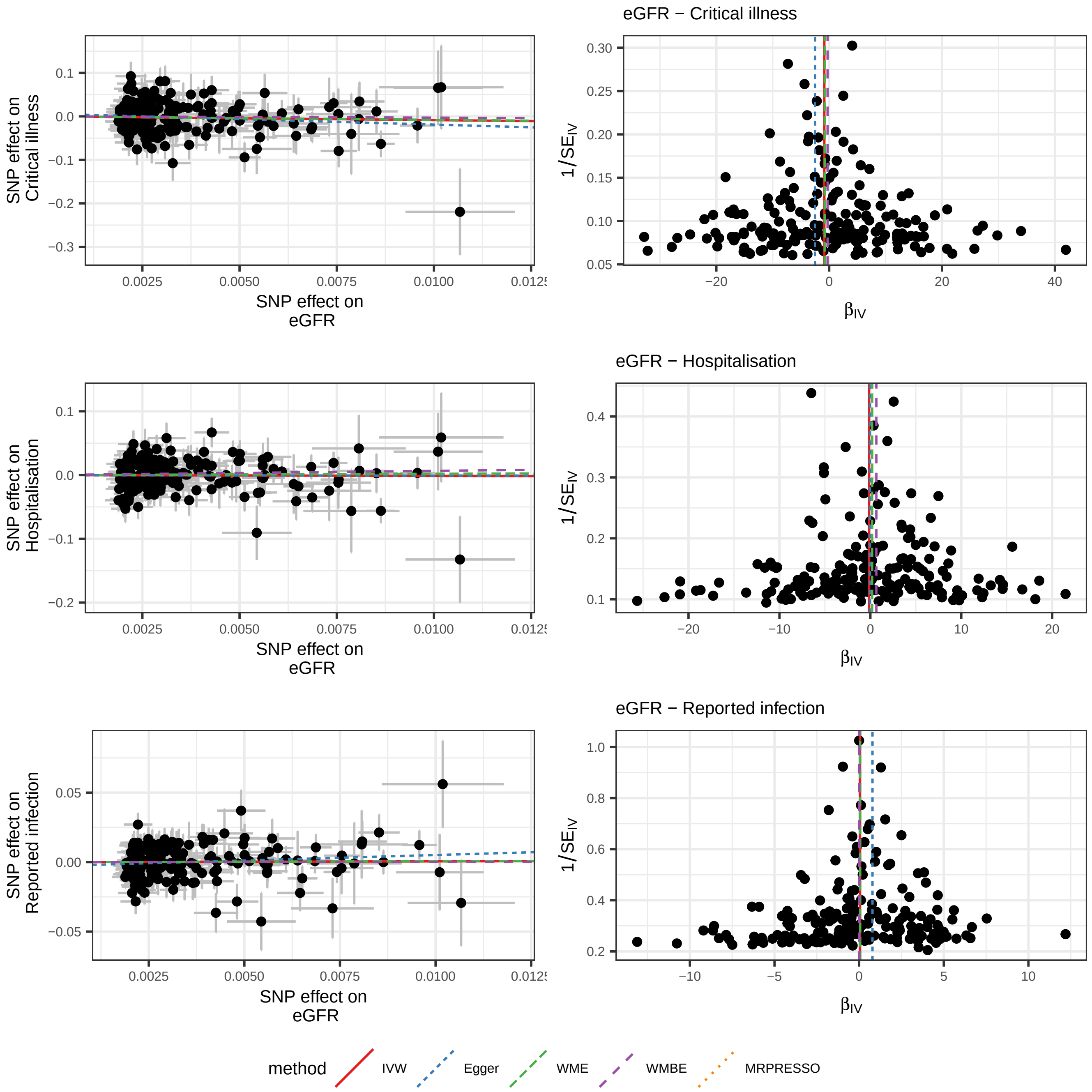

eGFR − Critical illness
eGFR − Hospitalisation
eGFR − Reported infection
SNP effect on Critical illness
SNP effect on Hospitalisation
SNP effect on Reported infection
SNP effect on eGFR
$1/SE_{IV}$
β_{IV}
method
IVW
Egger
WME
WMBE
MRPRESSO

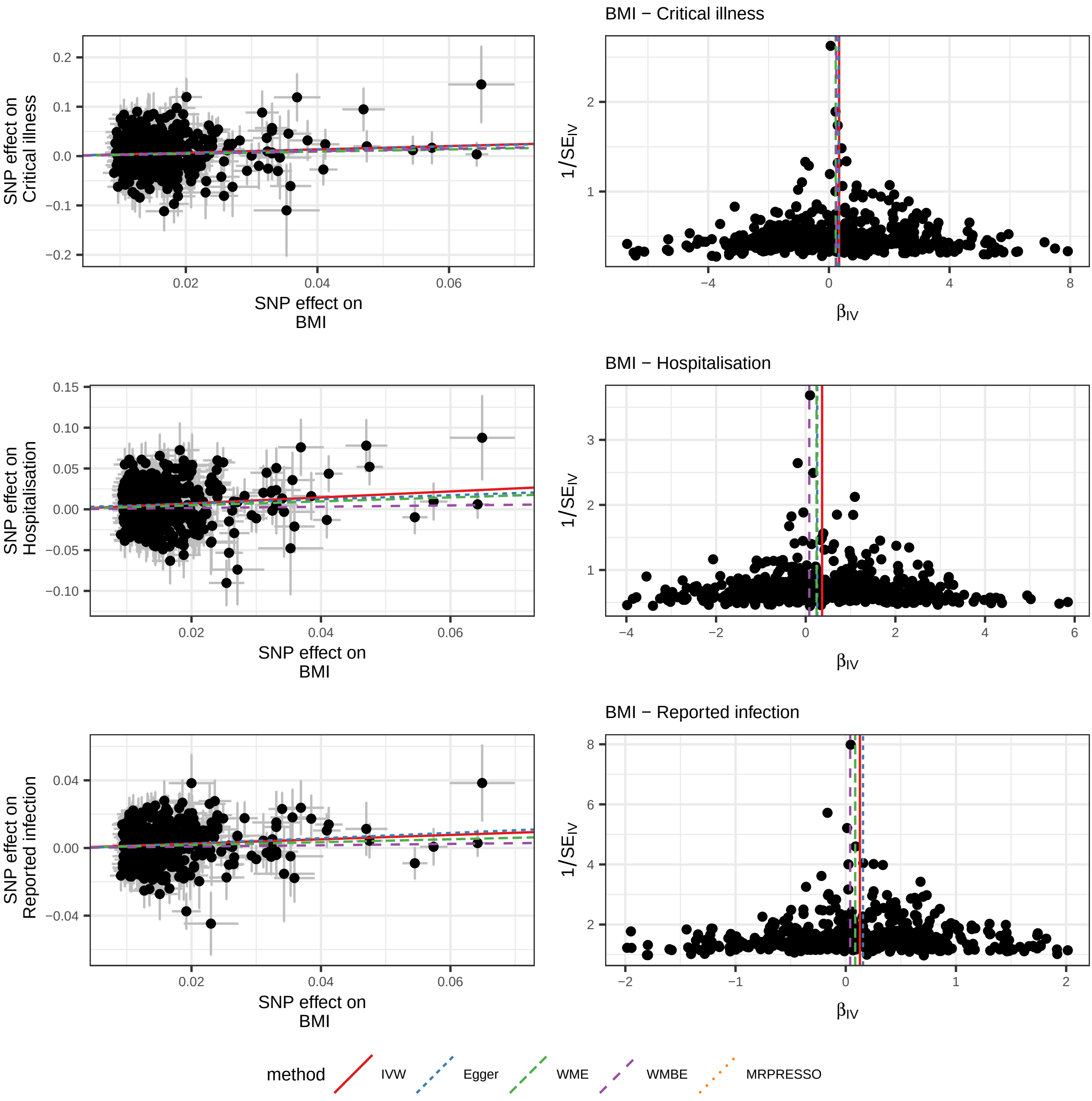
BMI − Critical illness
BMI − Hospitalisation
BMI − Reported infection
SNP effect on Critical illness
SNP effect on Hospitalisation
SNP effect on Reported infection
SNP effect on BMI
1/SE_IV
β_IV
method
IVW
Egger
WME
WMBE
MRPRESSO
