# Supplementary material for: Mapping the human genetic architecture of COVID-19
Source: Nature. 2021 Jul 8;600(7889):472–7. doi: 10.1038/s41586-021-03767-x (PMC8674144; doi:10.1038/s41586-021-03767-x)
Supplement: Supplementary file 7 — This file contains the full authorship for the Covid-19 Host Genetics Initiative. [file 41586_2021_3767_MOESM7_ESM.pdf]

Covid-19 Host Genetics Initiative

Full authorship list

* These authors contributed equally to this work

+ These authors are corresponding authors

Leadership

Leadership

Mari E.K. Niemi[1*], Juha Karjalainen[1*], Rachel G. Liao[2], Benjamin M Neale[4 +] (bneale@broadinstitute.org), Mark Daly[1,2,3 +] (mark.daly@helsinki.fi), Andrea Ganna[1,2,3 +] (andrea.ganna@helsinki.fi)

Writing group

Writing group lead

Mari E.K. Niemi[1 *], Gita A. Pathak[6], Shea J. Andrews[7], Masahiro Kanai[2],

Writing group member

Kumar Veerapen[2], Israel Fernandez-Cadenas[8], Eva C. Schulte[9,10,11], Pasquale Striano[12], Minttu Marttila[13], Camelia Minica[2], Eirini Marouli[14], Mohd Anisul Karim[15], Frank R. Wendt[6], Jeanne Savage[16], Laura Sloofman[7], Guillaume Butler-Laporte[17], Han-Na Kim[18], Stavroula Kanoni[14], Yukinori Okada[19], Jinyoung Byun[20], Younghun Han[20], Mohammed Jashim Uddin[21], George Davey Smith[22], Cristen J. Willer[23,24,25], Joseph D. Buxbaum[26,27,28],

Analysis group

Manuscript analyses team lead

Juha Karjalainen[1 *],

Manuscript analyses team members: meta-analysis

Juha Mehtonen[1],

Manuscript analyses team members: heritability, methods and supplements

Mari E.K. Niemi[1] ,

Manuscript analyses team members: phewas

Gita A. Pathak[6],

Manuscript analyses team members: mendelian randomization

Shea J. Andrews[7],

Manuscript analyses team members: PC projection, gene prioritization

Masahiro Kanai[2],

Manuscript analyses team members: gene prioritization

Hilary Finucane[3,29],

Manuscript analyses team members: sensitivity analysis

Mattia Cordioli[30],

Manuscript analyses team members: PC projection

Alicia R. Martin[3,29], Wei Zhou[3,29],

In-silico analysis team member

Mattia Cordioli[31], Bogdan Pasaniuc[32], Hanna Julienne[33], Hugues Aschard[33], Huwenbo Shi[34], Loic Yengo[35,36,37], Renato Polimanti[6], Maya Ghoussaini[38,39], Jeremy Schwartzentruber[38,39], Ian Dunham[39,40], Joseph D. Buxbaum[41],

Project management group

Project management lead

Rachel G. Liao[2],

Project managment support

Karolina Chwialkowska[42], Margherita Francescatto[43], Amy Trankiem[2], Mary K. Balaconis[2],

Phenotype steering group

Lea Davis[44], Andrea Ganna[1,2,3] , Sulggi Lee[45], James Priest[46], Alessandra Renieri[47,48], Vijay G. Sankaran[49], David van Heel[50], Patrick Deelen[51,52], J. Brent Richards[53,54,55], Tomoko Nakanishi[54,56,57,58], Les Biesecker[59], V. Eric Kerchberger[44], J. Kenneth Baillie[60,61,62],

Data dictionary

Alessandra Renieri[47,48], Francesca Mari[63,64,65], Anna Bernasconi[66], Stefano Ceri[67], Arif Canakoglu[67],

Scientific communication group

Scientific communication lead

Kumar Veerapen[2], Brooke Wolford[68],

Scientific communication member

Amy Trankiem[2], Annika Faucon[69], Atanu Kumar Dutta[70], Claudia Schurmann[71], Emi Harry[72], Ewan Birney[73], Gita A. Pathak[6], Huy Nguyen[2], Jamal Nasir[74], James Priest[46], Mari Kaunisto[75], Minttu Marttila[13], Matthew Solomonson[2], Nicole Dueker[76], Nirmal Vadgama[77], Shea J Andrews[7], Sophie Limou[78], Rachel G. Liao[2], Andrea Ganna[1,2,3],

Translator

Souad Rahmouni[79], Hamdi Mbarek[80], Dima Darwish[81], Atanu Kumar Dutta[70], Md Mesbah Uddin[82,83], Israel Fernandez-Cadenas[8], Raquel Albertos[84], Jordi Pérez-Tur[85,86,87], Ruolin Li[88], Lasse Folkersen[89], Ida Moltke[90], Nils Koelling[91], Eva C. Schulte[92], Claudia Schurmann[71], Alexander Teumer[93,94], Athanasios Kousathanas[95], Eirini Marouli[14], Alicia Utrilla[96], Ricardo A. Verdugo[97], Ruth Zárate[98], Carolina Medina-Gómez[99], David Gómez-Cabrero[100,101], Elena Carnero-Montoro[102], Jordi Pérez-Tur[85,86,87], Israel Fernandez-Cadenas[8], Carmen L. Cadilla[103], Andrés Moreno-Estrada[104], Adriana Garmendia[104], Leire Moya[105], Bahar Sedaghati-khayat[99], Minttu Marttila[13], Palwendé Romuald Boua[106], Guillaume Butler-Laporte[17], Marie-Julie Favé[107], Laurent Francioli[2], Audrey Lemaçon[108], Sophie Limou[78], Isabelle Migeotte[109], Atanu Kumar Dutta[110], Sanjay Patel[111], Reka Varnai[112], Jozsef L. Szentpeteri[112], Csilla Sipeky[113], Francesca Colombo[114], Margherita Francescatto[43], Kathrin von Hohenstaufen[115], Pietro Lio[116], Costanza Vallerga[99], Pasquale Striano[12], Qingbo Wang[2], Masahiro Kanai[2], Yosuke Tanigawa[46], Tomoko Nakanishi[54,56,57,58], Hogune Im[117], Chulho Han[117], Han Song[117], Jiwoo Lim[117], Younhe Lee[117], Sugyeong Kim[117], Sangyoon Im[118], Biljana Atanasovska[119], Hajar Fauzan Ahmad[120], Kumar Veerapen[121], Cindy Boer[99], Philip Jansen[122], Lude Franke[123], Karolina Chwialkowska[42], Elżbieta Kaja[124], Dorota Pasko[95], Ingrid Kennis-Szilagyi[99], Sergey A.

Kornilov[125], Vid Prijatelj[99], Ivana Prokić[99], Minttu Marttila[13], Ilangkumaran Sivanadhan[126], Sarala Perumal[127], Sahar Esmaeeli[128], Nathaniel M. Pearson[129], Ruth Zárate[98], Mohd Anisul Karim[38,39],

Website Development

Website Development Lead

Huy Nguyen[2], Matthew Solomonson[2],

23andMe

Analysis Team Lead

Adam Auton[130],

Data Collection Lead

23andMe COVID-19 Team[130], Janie F. Shelton[130], Anjali J. Shastri[130],

Analysis Team Member

Teresa Filshtein-Sonmez[130], Daniella Coker[130], Antony Symons[130], Jorge Esparza-Gordillo[131], Stella Aslibekyan[130], Jared O'Connell[130],

Data Collection Member

Chelsea Ye[130], Catherine H. Weldon[130],

ACCOuNT

Analysis Team Lead

Minoli Perera[132],

Data Collection Lead

Kevin O'Leary[133], Matthew Tuck[134], Travis O'Brien[135], David Meltzer[136], Peter O'Donnell[137], Edith Nutescu[138],

Analysis Team Member

Guang Yang[139],

Data Collection Member

Cristina Alarcon[140], Stefanie Herrmann[140], Sophia Mazurek[141], Jeff Banagan[142], Zacharia Hamidi[143], April Barbour[144], Noora Raffat[145], Diana Moreno[146],

Admin Team Member

Paula Friedman[147],

Amsterdam UMC COVID Study Group

Analysis Team Lead

Bart Ferwerda[148],

Data Collection Lead

Diederik van de Beek[148], Matthijs C. Brouwer[148], Alexander P.J. Vlaar[149], W. Joost Wiersinga[150],

Analysis Team Member

Danielle Posthuma[16], Elleke Tissink[16], A. H. (Koos) Zwinderman[151], Emil Uffelmann[16],

Data Collection Member

Michiel van Agtmael[150], Anne Geke Algera[149], Frank van Baarle[149], Diane Bax[152], Martijn Beudel[148], Harm Jan Bogaard[153], Marije Bomers[150], Peter I. Bonta[153], Lieuwe Bos[149], Michela Botta[149], Justin de Brabander[150], Godelieve de Bree[150], Sanne de Bruin[149], Marianna Bugiani[154], Esther Bulle[149], Osoul

Chouchane[150], Alex Cloherty[152], Dave Dongelmans[149], Paul Elbers[149], Lucas Fleuren[149], Suzanne Geerlings[150], Bart Geerts[155], Theo Geijtenbeek[152], Armand Girbes[149], Bram Goorhuis[150], Martin P. Grobusch[150], Florianne Hafkamp[152], Laura Hagens[149], Jorg Hamann[156], Vanessa Harris[150], Robert Hemke[157], Sabine M. Hermans[150], Leo Heunks[149], Markus Hollmann[155], Janneke Horn[149], Joppe W. Hovius[150], Menno D. de Jong[158], Rutger Koning[148], Niels van Mourik[149], Jeannine Nellen[150], Esther J. Nossent[153], Frederique Paulus[149], Edgar Peters[150], Tom van der Poll[150], Bennedikt Preckel[152], Jan M. Prins[150], Jorinde Raasveld[149], Tom Reijnders[150], Michiel Schinkel[150], Marcus J. Schultz[149], Alex Schuurman[150], Kim Sigaloff[150], Marry Smit[149], Cornelis S. Stijnis[150], Willemke Stilma[159], Charlotte Teunissen[149], Patrick Thoral[149], Anissa Tsonas[150], Marc van der Valk[150], Denise Veelo[155], Heder de Vries[149], Michèle van Vugt[150], Dorien Wouters[159], René P. Minnaar[160], Adrie Kromhout[160], Kees W.J. van Uffelen[160], Ruud A. Wolterman[161],

AncestryDNA COVID-19 Research Study

Analysis Team Lead

Genevieve Roberts[162],

Data Collection Lead

Danny Park[162],

Admin Team Lead

Catherine A. Ball[162],

Analysis Team Member

Marie Coignet[162], Shannon McCurdy[162], Spencer Knight[162], Raghavendran Partha[162], Brooke Rhead[162],

Data Collection Member

Miao Zhang[162], Nathan Berkowitz[162], Michael Gaddis[162], Keith Noto[162], Luong Ruiz[162], Milos Pavlovic[162],

Admin Team Member

Eurie L. Hong[162], Kristin Rand[162], Ahna Girshick[162], Harendra Guturu[162], Asher Haug Baltzell[162],

BelCovid

Analysis Team Lead

Mari E.K. Niemi[1] ,

Data Collection Lead

Isabelle Migeotte[109], Souad Rahmouni[163], Julien Guntz[164],

Admin Team Lead

Yves Beguin[165],

Analysis Team Member

Mattia Cordioli[31], Sara Pigazzini[5], Lindokuhle Nkambule[166,167],

Data Collection Member

Youssef Bouysran[168], Adeline Busson[168], Xavier Peyrassol[168], Françoise Wilkin[168], Bruno Pichon[168], Guillaume Smits[168], Isabelle Vandernoot[168], Jean-Christophe Goffard[169], Michel Georges[163], Michel Moutschen[170], Benoit Misset[170], Gilles Darcis[170], Julien Guiot[170], Laurent Jadot[164], Samira Azarzar[170], Patricia Dellot[171], Stéphanie Gofflot[165], Sabine Claassen[164], Axelle Bertrand[172], Gilles Parzibut[171], Mathilde Clarinval[171], Catherine Moermans[171], Olivier Malaise[171], Kamilia El Kandoussi[165], Raphaël Thonon[165],

Pascale Huynen[171], Alyssia Mesdagh[171], Sofia Melo[163], Nicolas Jacques[163], Emmanuel Di Valentin[163], François Giroule[163], Alice Collignon[163], Coraline Radermecker[163], Marielle Lebrun[163], Alice Collignon[163], Hélène Perée[163], Samuel Latour[163], Olivia Barada[163], Judit Sanchez[163], Claire Josse[173], Bouchra Boujemla[173], Margot Meunier[163], Emeline Mariavelle[163], Sandy Anania[163], Hélène Gazon[163], Danusia JUSZCZAK[173], Marjorie FADEUR[173], Séverine CAMBY[173], Christelle Meuris[173], Marie Thys[173], Jessica Jacques[173], Monique Henket[173], Philippe Léonard[173], Frederic Frippiat[173], Jean-Baptiste Giot[173], Anne-Sophie Sauvage[173], Christian Von Frenckell[173], Myriam Mni[163], Marie Wéry[163], Alicia Staderoli[173], Yasmine Belhaj[163], Bernard Lambermont[173],

Biobanque Quebec COVID19

Analysis Team Lead

Tomoko Nakanishi[54,56,57,58],

Data Collection Lead

David R. Morrison[54],

Admin Team Lead

Vincent Mooser[174], J. Brent Richards[53,54,175],

Analysis Team Member

Guillaume Butler-Laporte[54,176], Vincenzo Forgetta[54], Rui Li[174],

Data Collection Member

Biswarup Ghosh[54], Laetitia Laurent[54], Alexandre Belisle[174], Danielle Henry[54], Tala Abdullah[54], Olumide Adeleye[54], Noor Mamlouk[54], Nofar Kimchi[54], Zaman Afrasiabi[54], Nardin Rezk[54], Branka Vulesevic[54], Meriem Bouab[54], Charlotte Guzman[54], Louis Petitjean[54], Chris Tselios[54], Xiaoqing Xue[54], Jonathan Afilalo[54], Marc Afilalo[177,178], Maureen Oliveira[179], Bluma Brenner[180], Nathalie Brassard[181], Madeleine Durand[182,183],

Admin Team Member

Erwin Schurr[184], Pierre Lepage[174], Jiannis Ragoussis[174], Daniel Auld[174], Michaël Chassé[183,185], Daniel E. Kaufmann[183,186], G. Mark Lathrop[174], Darin Adra[54],

BioVU

Analysis Team Lead

Lea K. Davis[187,188], Nancy J. Cox[187,188], Jennifer E. Below[187,188],

Analysis Team Member

Julia M. Sealock[187,188], Annika B. Faucon[187,188], Megan M. Shuey[187,188], Hannah G. Polikowsky[187,188], Lauren E. Petty[187,188], Douglas M. Shaw[187,188], Hung-Hsin Chen[187,188], Wanying Zhu[187,188],

Bonn Study of COVID19 genetics

Data Collection Lead

Kerstin U. Ludwig[189],

Analysis Team Member

Julia Schröder[189], Carlo Maj[190],

Data Collection Member

Selina Rolker[189], Markus M. Nöthen[189], Julia Fazaal[189], Verena Keitel[191], Björn-Erik Ole Jensen[191], Torsten Feldt[191], Ingo Kurth[192], Nikolaus Marx[193], Michael Dreher[194], Isabell Pink[195], Markus Cornberg[196], Thomas Illig[197], Clara Lehmann[198,199,200], Philipp Schommers[198,199,200], Max Augustin[201], Jan Rybniker[201], Lisa Knopp[191], Thomas Eggermann[192], Sonja Volland[197], Janine Altmüller[202], Marc M. Berger[203], Thorsten Brenner[203], Anke Hinney[204], Oliver Witzke[205], Robert Bals[206], Christian Herr[206], Nicole Ludwig[207], Jörn Walter[208],

CHRIS

Analysis Team Lead
Christian Fuchsberger[209],
Data Collection Lead
Cristian Pattaro[209], Alessandro De Grandi[209],
Admin Team Lead
Peter Pramstaller[209],
Analysis Team Member
David Emmert[209], Roberto Melotti[209], Luisa Foco[209],
Admin Team Member
Deborah Mascalzoni[209], Martin Gögele[209], Francisco Domingues[209], Andrew Hicks[209],

Colorado Center for Personalized Medicine (CCPM)

Analysis Team Lead
Christopher R Gignoux[210],
Data Collection Lead
Stephen J Wicks[210], Kristy Crooks[210],
Admin Team Lead
Kathleen C Barnes[210],
Analysis Team Member
Michelle Daya[210], Jonathan Shortt[210], Nicholas Rafaels[210], Sameer Chavan[210],

Columbia University COVID19 Biobank

Analysis Team Lead
David B. Goldstein[211], Krzysztof Kiryluk[212],
Data Collection Lead
Soumitra Sengupta[213], Wendy Chung[214], Muredach P. Reilly[215],
Analysis Team Member
Atlas Khan[215], Chen Wang[215], Gundula Povysil[216], Nitin Bhardwaj[216], Ali G. Gharavi[215], Iuliana Ionita-Laza[217],
Data Collection Member
Ning Shang[215], Sheila M. O'Byrne[215], Renu Nandakumar[215], Amritha Menon[213], Yat S. So[213], Eldad Hod[218],
Admin Team Member
Danielle Pendrick[218],

Corea (Genetics of COVID-related Manifestation)

Analysis Team Lead

Han-Na Kim[219,220],

Data Collection Lead

Soo-kyung Park[221], Hyung-Lae Kim[222], Chang Kyung Kang[223], Hyo-Jung Lee[224], Kyoung-Ho Song[225],

Admin Team Lead

Kyung Jae Yoon[226,227,228], Nam-Jong Paik[229,230],

Analysis Team Member

Woojin Seok[231], Heejun Yoon[232],

Data Collection Member

Eun-Jeong Joo[233], Yoosoo Chang[234,235], Seungho Ryu[234,235], Wan Beom Park[223], Jeong Su Park[236], Kyoung Un Park[236], Sin Young Ham[225], Jongtak Jung[225], Eu Suk Kim[225], Hong Bin Kim[225],

COVID19-Hostage

Analysis Team Lead

David Ellinghaus[237,238], Frauke Degenhardt[237], Mario Cáceres[239,240], Simonas Juzenas[237], Tobias L Lenz[241,242],

Data Collection Lead

Agustín Albillos[243,244], Antonio Julià[245], Bettina Heidecker[246], Eva C Schulte[9,247,248], Federico Garcia[249,250], Florian Kurth[246], Florian Tran[237], Frank Hanses[251,252], Heinz Zoller[253], Jan C Holter[254,255], Javier Fernández[256,257], Leif Erik Sander[246], Philip Rosenstiel[237], Philipp Koehler[258,259,260], Rafael de Cid[261], Rosanna Asselta[262,263], Siegfried Goerg[264], Stefan Schreiber[237,265], Ute Hehr[266],

Admin Team Lead

Andre Franke[237,267], Johannes R Hov[254,268,269,270], Sandra May[237], Tom H Karlsen[254,269,270,271], Trine Folseraas[254,268,269,270],

Analysis Team Member

Ana Teles[241,242], Clinton Azuure[241], Eike Matthias Wacker[237], Florian Uellendahl-Werth[237], Hesham ElAbd[237], Jatin Arora[29,272,273,274,275], Jon Lerga-Jaso[239], Lars Wienbrandt[237], Malte Christoph Rühlemann[237], Mareike Wendorff[237], May Sissel Vadla[276,277], Ole Bernt Lenning[276], Onur Özer[241,242], Ronny Myhre[278], Soumya Raychaudhuri[29,272,273,274,275,279],

Data Collection Member

Aaron Blandino Ortiz[280], Adolfo Garrido Chercoles[281], Agustín Ruiz[282,283], Alberto Mantovani[262,263], Aleksander Rygh Holten[254,284], Alena Mayer[246], Alessandro Cherubini[285], Alessandro Protti[262,263], Alessio Aghemo[262,263], Alessio Gerussi[286,287], Alfredo Ramirez[288,289,290,291], Alice Braun[246], Ana Barreira[292], Ana Lleo[262,263], Anders Benjamin Kildal[293], Andrea Ganna[1,2,3], Andreas Glück[294], Anna Carreras Nolla[261], Anna Latiano[295], Anne Ma Dyrhol-Riise[254,296], Antonio Muscatello[285], Antonio Voza[262], Ariadna Rando-Segura[297,298], Aurora Solier[299], Banasik Karina[238], Beatriz Cortes[261], Beatriz Mateos[243,244], Beatriz Nafria Jimenez[281], Beatriz Nafria-Jimenez[281], Benedikt Schaefer[253], Carla Bellinghausen[300], Carlos Ferrando[256], Carmen Quereda[301], Carsten Skurk[246], Charlotte Thibeault[246], Christoph D Spinner[302], Christoph Lange[303,304,305], Cinzia Hu[285], Claudio Cappadona[263], Cristiana Bianco[285], Cristina Sancho[306], Dag Arne Lihaug Hoff[307,308], Daniela Galimberti[285], David Jiménez[299], David Pestaña[309], David Toapanta[310], Elena Azzolini[262,263], Elio Scarpini[285], Elisa T Helbig[246], Eloisa Urrechaga[311], ELOISA URRECHAGA[311], Elvezia

Maria Paraboschi[262,263], Emanuele Pontali[312], Enric Reverter[256,313], Enrique Navas[301], Eunate Arana[314], Félix García Sánchez[315], Ferruccio Ceriotti[285], Francesco Malvestiti[316], Francisco Mesonero[243,244], Gianni Pezzoli[317], Giuseppe Lamorte[285], Holger Neb[318], Ilaria My[262], Isabel Hernández[282,283], Itziar de Rojas[282,283], Iván Galván-Femenia[261], Jan Heyckendorf[303,304,305], Jan Rybniker[258,260,319], Jesus Banales[320], Jesus M Banales[244,321,322], Joan Ramon Badia[256], Jochen Schneider[302], Josune Goikoetxea[323], Julia Kraft[246], Karl Erik Müller[324], Karoline I Gaede[325,326,327], Koldo Garcia-Etxebarria[244,328,329], Kristian Tonby[254,330], Lars Heggelund[324,331], Laura Izquierdo[332], Laura Izquierdo-Sanchez[244,328], Lauro Sumoy[333], Lena J Lippert[246], Leonardo Terranova[285], Lindokuhle Nkambule[334], Lucia Garbarino[312], Luis Bujanda[244,328,329], Luis Téllez[243,244], Luisa Roade[298], Mahnoosh Ostadreza[285], Maider Intxausti[306], Manolis Kogevinas[335,336,337,338], Mari EK Niemi[31] , María A Gutiérrez-Stampa[339], Maria JGT Vehreschild[340], Marta Marquié[282,283], Massimo Castoldi[341], Mattia Cordioli[31], Maurizio Cecconi[262,263], Mauro D'Amato[321,342,343,344], Mercè Boada[282,283], Michael J Seilmaier[345], Michela Mazzocco[312], Miguel Rodríguez-Gandía[243,244], Natale Imaz Ayo[314], Natalia Blay[261], Nilda Martínez[346], Norwegian SARS-CoV-2 Study group[347], Oliver A Cornely[258,348,349,350], Orazio Palmieri[295], Paolo Tentorio[262], Pedro M Rodrigues[244,322,332], Pedro P España[311], Pedro Pablo España[311], Per Hoffmann[351], Petra Bacher[352,353,354], Phillip Suwalski[246], Raúl de Pablo[280], Rosa Nieto[299], Salvatore Badalamenti[262], Sandra Ciesek[355,356], Sara Bombace[262], Sara Pigazzi[31], Sibylle Wilfling[252,266,357], Søren Brunak[238], Stefanie Heilmann-Heimbach[358], Stefano Duga[262,263], Stephan Ripke[246], The Humanitas COVID-19 Task Force[262], The Humanitas Gavazzeni COVID-19 Task Force[341], Thomas Bahmer[294], Ulf Landmesser[359], Ulrike Protzer[9,360], Valeria Rimoldi[263], Vegard Skogen[361,362], Victor Andrade[289,291], Victor Moreno[336,363,364,365], Wolfgang Poller[246], Xavier Farre[261], Xiaomin Wang[246], Yascha Khodamoradi[340], Zehra Karadeniz[246],

Admin Team Member

Marit M Grimsrud[254,269,270],

deCODE

Analysis Team Lead

Daniel F. gudbjartsson[366],

Data Collection Lead

Kari Stefansson[366],

Analysis Team Member

Patrick Sulem[366], Gardar Sveinbjornsson[366], Pall Melsted[366], Gudmundur Norddahl[366], Kristjan Helgi Swerford Moore[366],

Data Collection Member

Unnur Thorsteinsdottir[366], Hilma Holm[366],

Determining the Molecular Pathways and Genetic Predisposition of the Acute Inflammatory Process Caused by SARS-CoV-2

Analysis Team Lead

Marta E. Alarcón-Riquelme[367],

Data Collection Lead

David Bernardo[368,369],

Analysis Team Member

Manuel Martínez-Bueno[367],

Data Collection Member

Silvia Rojo Rello[370],

Estonian Biobank

Analysis Team Lead

Reedik Mägi[371],

Data Collection Lead

Lili Milani[371],

Admin Team Lead

Andres Metspalu[371],

Analysis Team Member

Triin Laisk[371], Kristi Läll[371], Maarja Lepamets[371],

Data Collection Member

Tõnu Esko[371], Ene Reimann[371], Paul Naaber[372], Edward Laane[373], Jaana Pesukova[374], Pärt Peterson[375], Kai Kisand[376], Jekaterina Tabri[377], Raili Allos[377], Kati Hensen[377], Joel Starkopf[378], Inge Ringmets[379], Anu Tamm[380], Anne Kallaste[380],

Admin Team Member

Helene Alavere[371], Kristjan Metsalu[371], Mairo Puusepp[371],

FinnGen

Admin Team Lead

FinnGen[381],

Data Collection Member

Kati Kristiansson[382], Sami Koskelainen[382], Markus Perola[382,383], Kati Donner[30], Katja Kivinen[30], Aarno Palotie[30],

Admin Team Member

Mari Kaunisto[30],

Functional Host Genomics in Infectious Diseases (FHoGID)

Analysis Team Lead

Carlo Rivolta[384,385],

Data Collection Lead

Pierre-Yves Bochud[386], Stéphanie Bibert[387], Noémie Boillat[386], Semira Gonseth Nussle[388], Werner Albrich[389],

Analysis Team Member

Mathieu Quinodoz[384,385], Dhryata Kamdar[384,385],

Data Collection Member

Noémie Suh[390], Dionysios Neofytos[391], Véronique Erard[392], Cathy Voide[393], FHoGID[394], RegCOVID[395], P-PredictUs[396], SeroCOVID[397], CRiPSI[398],

GCAT. Genomes For Life.

Analysis Team Lead

Rafael de Cid[399],

Data Collection Lead

Anna Carreras[399], Victor Moreno[400], Manolis Kogevinas[335,336,337,338],

Analysis Team Member

Iván Galván-Femenía[399], Natalia Blay[399], Xavier Farré[399], Lauro Sumoy[399],

Data Collection Member

Beatriz Cortés[399], Josep Maria Mercader[401], Marta Guindo-Martinez[402], David Torrents[403], Judith Garcia-Aymerich[335,336,337], Gemma Castaño-Vinyals[335,336,337,338], Carlota Dobaño[335,336],

GEN-COVID Multicenter Study

Analysis Team Lead

Marco Gori[404,405], Mari E.K. Niemi[1] ,

Data Collection Lead

Alessandra Renieri[64,406,407], Francesca Mari[64,406,407], Mario Umberto Mondelli[408,409], Francesco Castelli[410], Massimo Vaghi[411], Stefano Rusconi[412,413], Francesca Montagnani[65,414], Elena Bargagli[415], Federico Franchi[416], Maria Antonietta Mazzei[417], Luca Cantarini[418], Danilo Tacconi[419], Marco Feri[420], Raffaele Scala[421], Genni Spargi[422], Cesira Nencioni[423], Maria Bandini[424], Gian Piero Caldarelli[425], Maurizio Spagnesi[424], Anna Canaccini[426], Agostino Ognibene[427], Antonella D'Arminio Monforte[428], Massimo Girardis[429], Andrea Antinori[430], Daniela Francisci[431,432], Elisabetta Schiaroli[431,432], Pier Giorgio Scotton[433], Sandro Panese[434], Renzo Scaggiante[435], Matteo Della Monica[436], Mario Capasso[437,438,439], Giuseppe Fiorentino[440], Marco Castori[441], Filippo Aucella[442], Antonio Di Biagio[443], Luca Masucci[444,445], Serafina Valente[446], Marco Mandalà[447], Patrizia Zucchi[448], Ferdinando Giannattasio[449], Domenico A. Coviello[450], Cristina Mussini[451], Giancarlo Bosio[452], Luisa Tavecchia[453], Lia Crotti[454,455,456,457], Marco Rizzi[458], Maria Teresa La Rovere[459], Simona Sarzi-Braga[460], Maurizio Bussotti[461], Sabrina Ravaglia[462], Rosangela Artuso[463], Antonio Perrella[464], Davide Romani[465], Paola Bergomi[466], Emanuele Catena[466],

Admin Team Lead

Simone Furini[407], Simona Dei[467],

Analysis Team Member

Elisa Benetti[407], Nicola Picchiotti[404,468], Maurizio Sanarico[469], Stefano Ceri[66], Pietro Pinoli[66], Francesco Raimondi[470], Filippo Biscarini[471], Alessandra Stella[472], Mattia Bergomi[473], Kristina Zguro[474], Katia Capitani[65,475], Mattia Cordioli[31], Sara Pigazzini[5], Mattia Cordioli[31], Sara Pigazzini[5], Lindokuhle Nkambule,

Data Collection Member

Chiara Fallerini[406,407], Sergio Daga[406,407], Margherita Baldassarri[406,407], Francesca Fava[64,406,407], Elisa Frullanti[406,407], Floriana Valentino[406,407], Gabriella Doddato[406,407], Annarita Giliberti[406,407], Rossella Tita[477], Sara Amitrano[477], Mirella Bruttini[65,406,477], Susanna Croci[406,407], Ilaria Meloni[406,407], Maria Antonietta Mencarelli[477], Caterina Lo Rizzo[477], Anna Maria Pinto[477], Giada Beligni[406,407], Andrea Tommasi[65,406,477], Laura Di Sarno[406,407], Maria Palmieri[406,407], Miriam Lucia Carriero[406,407], Diana Alaverdian[406,407], Nicola Iuso[406,407], Gabriele Inchingolo[406,407], Stefano Busani[429], Raffaele Bruno[408,409], Marco Vecchia[478], Mary Ann Belli[453], Stefania Mantovani[478], Serena Ludovisi[408,409], Eugenia Quiros-Roldan[410], Melania Degli Antoni[410], Isabella Zanella[479,480], Matteo Siano[413], Arianna Emiliozzi[430], Massimiliano Fabbiani[414], Barbara Rossetti[414],

Giacomo Zanelli[65,414], Laura Bergantini[415], Miriana D'Alessandro[415], Paolo Cameli[415], David Bennet[415], Federico Anedda[416], Simona Marcantonio[416], Sabino Scolletta[416], Susanna Guerrini[417], Edoardo Conticini[418], Bruno Frediani[418], Chiara Spertilli[419], Alice Donati[420], Luca Guidelli[421], Marta Corridi[422], Leonardo Croci[423], Paolo Piacentini[424], Elena Desanctis[424], Silvia Cappelli[424], Agnese Verzuri[426], Valentina Anemoli[426], Alessandro Pancrazi[427], Maria Lorubbio[427], Esther Merlini[428], Federica Gaia Miraglia[428], Sophie Venturelli[429], Andrea Cossarizza[481], Alessandra Vergori[430], Arianna Gabrieli[413], Agostino Riva[412,413], Francesco Paciosi[432], Francesca Andretta[433], Francesca Gatti[435], Saverio Giuseppe Parisi[482], Stefano Baratti[482], Carmelo Piscopo[436], Roberta Russo[437,438], Immacolata Andolfo[437,438], Achille Iolascon[437,438], Massimo Carella[441], Giuseppe Merla[437,483], Gabriella Maria Squeo[483], Pamela Raggi[484], Carmen Marciano[484], Rita Perna[484], Matteo Bassetti[443,485], Maurizio Sanguinetti[444,445], Alessia Giorli[447], Lorenzo Salerni[447], Pierpaolo Parravicini[448], Elisabetta Menatti[486], Tullio Trotta[449], Gabriella Coiro[449], Fabio Lena[487], Enrico Martinelli[452], Sandro Mancarella[453], Chiara Gabbi[488], Franco Maggiolo[458], Diego Ripamonti[458], Tiziana Bachetti[489], Claudia Suardi[490], Gianfranco Parati[454,455], Giordano Bottà[491], Paolo Di Domenico[491], Ilaria Rancan[492], Francesco Bianchi[65,464], Riccardo Colombo[466],

Genes & Health

Analysis Team Lead

David A van Heel[50],

Data Collection Lead

Karen A Hunt[50],

Admin Team Lead

Richard C Trembath[493],

Analysis Team Member

Qin Qin Huang[494], Hilary C Martin[494],

Data Collection Member

Dan Mason[495], Bhavi Trivedi[496], John Wright[495],

Admin Team Member

Sarah Finer[497], Genes & Health Research Team[498], Christopher J Griffiths[499],

Genes for Good

Analysis Team Lead

Albert V Smith[500],

Data Collection Member

Andrew P Boughton[500], Kevin W. Li[500], Jonathon LeFaive[500], Aubrey Annis[500],

Genetic determinants of COVID-19 complications in the Brazilian population

Analysis Team Lead

Mari E.K. Niemi[1] ,

Data Collection Lead

Cinthia E Jannes[501]

Admin Team Lead

Jose E Krieger[501], Alexandre C Pereira[501],
Analysis Team Member
Mariliza Velho[501], Emanuelle Marques[501], Mattia Cordioli[31], Sara Pigazzini[5], Lindokuhle Nkambule[166,167],
Data Collection Member
Isabella Ramos Lima[501], Mauricio Teruo Tada[501], Karina Valino[501],

Genetic influences on severity of COVID-19 illness in Korea

Analysis Team Lead
Mark McCarthy[502], Carrie Rosenberger[502],
Data Collection Lead
Jong Eun Lee[503],
Analysis Team Member
Diana Chang[502], Christian Hammer[502], Julie Hunkapiller[502], Anubha Mahajan[502], Sarah Pendergrass[502], Lara Sucheston-Campbell[502], Brian Yaspan[502],
Data Collection Member
Hyun Soo Lee[503], Eunsoon Shin[503], Hye Yoon Jang[503], Sunmie Kim[504], Sungmin Kym[505], Yeon-Sook Kim[505], Hyeongseok Jeong[506], Ki Tae Kwon[507], Shin-Woo Kim[507], Jin Yong Kim[508], Young Rock Jang[508], Hyun ah Kim[509], Ji yeon Lee[509], Jeong Eun Lee[510], Shinwon Lee[510], Kang-Won Choe[511], Yu Min Kang[511], Sun Ha Jee[512], Keum Ji Jung[512],

Genomic epidemiology of SARS-Cov-2 and host genetics in Coronavirus Disease 2019 (COVID-19)

Data Collection Lead
Victoria Parikh[513], Euan Ashley[514], Matthew Wheeler[515], Manuel Rivas[516], Carlos Bustamante[517], Benjamin Pinksy[518], Phillip Febbo[519], Kyle Farh[519], Gary P. Schroth[519], Francis deSouza[519],
Admin Team Lead
Karen Dalton[513], Jeff Christle[513],
Analysis Team Member
Christopher Deboever[520], Sándor Szalma[520], Yosuke Tanigawa[516], Simone Rubinacci[521], Olivier Delaneau[521],
Data Collection Member
John Gorzynski[513], Hannah de Jong[513], Shirley Sutton[513], Nathan Youlton[513], Ruchi Joshi[513], David Jimenez-Morales[513], Christopher Hughes[513], David Amar[513], Alex Ioannidis[516], Steve Hershman[513], Anna Kirillova[513], Kinya Seo[513], Yong Huang[513], Massa Shoura[522], Nathan Hammond[522], Nathaniel Watson[522], Archana Raja[513], ChunHong Huang[522], Malaya Sahoo[522], Hannah Wang[522],
Admin Team Member
Jimmy Zhen[513],

Genotek COVID-19 study

Analysis Team Lead
Alexander Rakitko[974],

Admin Team Lead
Valery Ilinsky[974],
Analysis Team Member
Danat Yermakovich[974], Iaroslav Popov[974], Alexander Chernitsov[974], Elena Kovalenko[974], Anna Krasnenko[974], Nikolay Plotnikov[974], Ivan Stetsenko[974], Anna Kim[974],

Helix and Healthy Nevada Project Exome+ COVID-19 Phenotypes

Analysis Team Lead
Elizabeth T. Cirulli[975],
Analysis Team Member
Kelly M. Schiabor Barrett[975], Alexandre Bolze[975], Simon White[975], Nicole L. Washington[975], James T. Lu[975],
Data Collection Member
Stephen Riffle[975], Francisco Tanudjaja[975], Xueqing Wang[975], Jimmy M. Ramirez III[975], Nicole Leonetti[975], Efren Sandoval[975], Iva Neveux[976], Shaun Dabe[977], Joseph J. Grzymski[976],

Host genomic variants associated to COVID-19 infection outcome

Analysis Team Lead
Juan Ignacio Esteban Miñano[978],
Data Collection Lead
Luis A. Aguirre[979],
Admin Team Lead
Eduardo López-Collazo[979],
Analysis Team Member
Manuel de la Mata Pazos[978], Luciano Cerrato[978], Lasse Folkersen[978],
Data Collection Member
Roberto Lozano-Rodríguez[979], José Avendaño-Ortiz[979], Verónica Terrón Arcos[979], Karla Marina Montalbán-Hernández[979], Jaime Valentín Quiroga[979], Alejandro Pascual-Iglesias[979],
Admin Team Member
Charbel Maroun-Eid[979], Alejandro Martín-Quirós[979],

Japan Coronavirus Taskforce

Analysis Team Lead
Ho Namkoong[980], Yukinori Okada[981,982,983], Seiya Imoto[984],
Data Collection Lead
Kazuhiko Katayama[985], Koichi Fukunaga[980], Yuko Kitagawa[986], Toshiro Sato[987], Naoki Hasegawa[988], Atsushi Kumanogoh[983,989,990], Akinori Kimura[991], Masumi Ai[992], Katsushi Tokunaga[993],
Admin Team Lead
Takanori Kanai[994], Satoru Miyano[995], Seishi Ogawa[996,997],
Analysis Team Member
Ryuya Edahiro[981,989], Kyuto Sonehara[981], Yuya Shirai[998], Masahiro Kanai[274],
Data Collection Member

Makoto Ishii[980], Hiroki Kabata[980], Katsunori Masaki[980], Hirofumi Kamata[980], Shinnosuke Ikemura[980], Shotaro Chubachi[980], Satoshi Okamori[980], Hideki Terai[980], Hiromu Tanaka[980], Atsuho Morita[980], Ho Lee[980], Takanori Asakura[980], Junichi Sasaki[999], Hiroshi Morisaki[1000], Yoshifumi Uwamino[1001], Kosaku Nanki[994], Yohei Mikami[994], Kazunori Tomono[1002], Kazuto Kato[1003], Fumihiko Matsuda[1004], Meiko Takahashi[1004], Nobuyuki Hizawa[1005], Yoshito Takeda[989], Haruhiko Hirata[989], Takayuki Shiroyama[989], Satoru Miyawaki[1006], Ken Suzuki[981], Yuichi Maeda[989,1007], Takuro Nii[1008], Yoshimi Noda[989], Takayuki Niitsu[989], Yuichi Adachi[989], Takatoshi Enomoto[989], Saori Amiya[989], Reina Hara[989], Kunihiko Takahashi[995], Tatsuhiko Anzai[995], Takanori Hasegawa[995], Satoshi Ito[995], Ryuji Koike[1009], Akifumi Endo[1010], Yuji Uchimura[1011], Yasunari Miyazaki[1012], Takayuki Honda[1012], Tomoya Tateishi[1012], Shuji Tohda[1013], Naoya Ichimura[1013], Kazunari Sonobe[1013], Chihiro Sassa[1013], Jun Nakajima[1013], Yasuhito Nannya[1014], Yosuke Omae[993], Kazuhisa Takahashi[1015], Norihiro Harada[1015], Makoto Hiki[1016,1017], Haruhi Takagi[1015], Ai Nakamura[1015], Etsuko Tagaya[1018], Masatoshi Kawana[1019], Ken Arimura[1018], Takashi Ishiguro[1020], Noboru Takayanagi[1020], Taisuke Isono[1020], Yotaro Takaku[1020], Kenji Takano[1020], Ryusuke Anan[1021], Yukiko Nakajima[1021], Yasushi Nakano[1021], Kazumi Nishio[1021], Soichiro Ueda[1022], Reina Hayashi[1022], Hiroki Tateno[1023], Isano Hase[1023], Shuichi Yoshida[1023], Shoji Suzuki[1023], Keiko Mitamura[1024], Fumitake Saito[1025], Tetsuya Ueda[1026], Masanori Azuma[1026], Tadao Nagasaki[1027], Yoshinori Yasui[1028], Yoshinori Hasegawa[1027], Yoshikazu Mutoh[1029], Takashi Yoshiyama[1030], Tomohisa Shoko[1031], Mitsuaki Kojima[1031], Tomohiro Adachi[1031], Motonao Ishikawa[1032], Kenichiro Takahashi[1033], Kazuyoshi Watanabe[1034], Tadashi Manabe[1035], Fumimaro Ito[1035], Takahiro Fukui[1035], Yohei Funatsu[1035], Hidefumi Koh[1035], Yoshihiro Hirai[1036], Hidetoshi Kawashima[1036], Atsuya Narita[1036], Kazuki Niwa[1037], Yoshiyuki Sekikawa[1037], Fukuki Saito[1038], Kazuhisa Yoshiya[1038], Tomoyuki Yoshihara[1038], Yusuke Suzuki[1039], Sohei Nakayama[1039], Keita Masuzawa[1039], Koichi Nishi[1040], Masaru Nishitsuji[1040], Maiko Tani[1040], Takashi Inoue[1041], Toshiyuki Hirano[1041], Keigo Kobayashi[1041], Naoki Miyazawa[1042], Yasuhiro Kimura[1042], Reiko Sado[1042], Takashi Ogura[1043], Hideya Kitamura[1043], Kota Murohashi[1043], Ichiro Nakachi[1044], Rie Baba[1044], Daisuke Arai[1044], Satoshi Fuke[1045], Hiroshi Saito [1045], Naota Kuwahara[1046], Akiko Fujiwara[1046], Takenori Okada[1046], Tomoya Baba[1047], Junya Noda[1047], Shuko Mashimo[1047], Kazuma Yagi[1048], Tetsuya Shiomi[1048], Mizuha Hashiguchi[1048], Toshio Odani[1049], Takao Mochimaru[1050,1051], Yoshitaka Oyamada[1050,1051], Nobuaki Mori[1052], Namiki Izumi[1053], Kaoru Nagata[1053], Reiko Taki[1053], Koji Murakami[1054], Mitsuhiro Yamada[1054], Hisatoshi Sugiura[1054], Kentaro Hayashi[1055], Tetsuo Shimizu[1055], Yasuhiro Gon[1055], Shigeki Fujitani[1056], Tomoya Tsuchida[1057], Toru Yoshida[1056], Takashi Kagaya[1058], Toshiyuki Kita[1058], Satoru Sakagami[1058], Yoshifumi Kimizuka[1059], Akihiko Kawana[1059], Yoshihiko Nakamura[1060], Hiroyasu Ishikura[1060], Tohru Takata[1061], Takahide Kikuchi[1062], Daisuke Taniyama[1062], Morio Nakamura[1062], Nobuhiro Kodama[1063], Yasunari Kaneyama[1063], Shunsuke Maeda[1063], Yoji Nagasaki[1064], Masaki Okamoto[1065], Sayoko Ishihara[1066], Akihiro Ito[1067], Yusuke Chihara[1068], MayumiTakeuchi[1068], Keisuke Onoi[1068], Naozumi Hashimoto[1069], Keiko Wakahara[1069], Akira Ando[1069], Makoto Masuda[1070], Aya Wakabayashi[1070], Hiroki Watanabe[1070], Hisako Sageshima[1071], Taka-Aki Nakada[1072], Ryuzo Abe[1072], Tadanaga Shimada[1072], Kodai Kawamura[1073], Kazuya Ichikado[1073], Kenta Nishiyama[1073], Masaki Yamasaki[1074], Satoru Hashimoto[1074], Yu Kusaka[1075], Takehiko Ohba[1075], Susumu Isogai[1075], Minoru Takada[1076], Hidenori Kanda[1076], Yuko Komase[1077], Fumiaki Sano[1078], Koichiro Asano[1079], Tsuyoshi Oguma[1080], Masahiro Harada[1081], Takeshi Takahashi[1081], Takayuki Shibusawa[1081], Shinji Abe[1082], Yuta Kono[1082], Yuki Togashi[1082], Takehiro Izumo[1083], Minoru Inomata[1083],

Nobuyasu Awano[1083], Shinichi Ogawa[1084], Tomouki Ogata[1084], Shoichiro Ishihara[1084], Arihiko Kanehiro[1085], Shinji Ozaki[1085], Yasuko Fuchimoto[1085], Yuichiro Kitagawa[1086], Shozo Yoshida[1086], Shinji Ogura[1086], Kei Nishiyama[1087], Kousuke Yoshida[1088], Satoru Beppu[1088], Satoru Fukuyama[1089], Yoshihiro Eriguchi[1090], Akiko Yonekawa[1090], Yoshiaki Inoue[1091], Kunihiro Yamagata[1092], Shigeru Chiba[1093], Osamu Narumoto[1094], Hideaki Nagai[1094], Nobuharu Ooshima[1094], Mitsuru Motegi[1095], Hironori Sagara[1096], Akihiko Tanaka[1096], Shin Ohta[1096], Yoko Shibata[1097], Yoshinori Tanino[1097], Yuki Sato[1097], Yuichiro Yamada[1098], Takuya Hashino[1098], Masato Shinoki[1098], Hajime Iwagoe[1099], Tomonori Imamura[1100], Akira Umeda[1101], Hisato Shimada[1101], Mayu Endo[1102], Shinichi Hayashi[1103], Mai Takahashi[1103], Shigefumi Nakano[1103], Masakiyo Yatomi[1104], Toshitaka Maeno[1104], Tomoo Ishii[1105], Mitsuyoshi Utsugi[1106], Akihiro Ono[1106], Kensuke Kanaoka[1107], Shoichi Ihara[1107], Kiyoshi Komuta[1107],

Lifelines

Analysis Team Lead

Lude Franke[1108],

Data Collection Lead

Marike Boezen[1109],

Analysis Team Member

Patrick Deelen[1110], Annique Claringbould[1108], Esteban Lopera[1108], Robert Warmerdam[1108], Judith. M. Vonk[1111],

Data Collection Member

Pauline Lanting[1112], Anil P. S. Ori[1113],

Lung eQTL Consortium

Data Collection Member

Ma'en Obeidat[1114], Ana I. Hernández Cordero[1114], Don D. Sin[1114,1115], Yohan Bossé[1116], Philippe Joubert[1116], Ke Hao[1117], David Nickle[1118,1119], Wim Timens[1120,1121], Maarten van den Berge[1121,1122],

Mass General Brigham - Host Vulnerability to COVID-19

Analysis Team Lead

Yen-Chen Anne Feng[1123], Josep Mercader[1124,1125],

Data Collection Lead

Scott T Weiss[1126], Elizabeth W. Karlson[1127], Jordan W. Smoller[1128], Shawn N Murphy[1129], James B. Meigs[1130], Ann E. Woolley[1127],

Admin Team Lead

Robert C. Green[1131],

Data Collection Member

Emma F Perez[273],

Michigan Genomics Initiative (MGI)

Analysis Team Lead

Brooke Wolford[1132],

Admin Team Lead

Sebastian Zöllner[500],

Analysis Team Member

Jiongming Wang[500], Andrew Beck[500],

Mount Sinai Health System COVID-19 Genomics Initiative

Analysis Team Lead

Laura G. Sloofman[26,27,28],

Data Collection Lead

Steven Ascolillo[1133], Robert P. Sebra[1134,1135], Brett L. Collins[1136], Tess Levy[1136],

Admin Team Lead

Joseph D. Buxbaum[1136], Stuart C. Sealfon[28],

Analysis Team Member

Shea J. Andrews[7], Daniel M. Jordan[1137,1138], Ryan C. Thompson[1139,1140,1141], Kyle Gettler[1142], Kumardeep Chaudhary[1138,1143], Gillian M. Belbin[1144], Michael Preuss[1145,1146], Clive Hoggart[28,1147], Sam Choi[1148], Slayton J. Underwood[28,1149],

Data Collection Member

Irene Salib[1134], Bari Britvan[1136], Katherine Keller[1136], Lara Tang[1136], Michael Peruggia[1136], Liam L. Hiester[1136], Kristi Niblo[1136], Alexandra Aksentijevich[1136], Alexander Labkowsky[1136], Avromie Karp[1136], Menachem Zlatopolsky[1136], Marissa Zyndorf[1134],

Admin Team Member

Alexander W. Charney[1150], Noam D. Beckmann[1133], Eric E. Schadt[1134,1135], Noura S. Abul-Husn[1144], Judy H. Cho[1138,1143], Yuval Itan[1138,1143], Eimear E. Kenny[1144], Ruth J.F. Loos[1145,1146,1151], Girish N. Nadkarni[1145,1152,1153,1154,1155], Ron Do[1138,1143], Paul O'Reilly[1148], Laura M. Huckins[1156],

MyCode Health Initiative

Analysis Team Lead

Manuel A.R. Ferreira[1157], Goncalo R. Abecasis[1157],

Data Collection Lead

Joseph B. Leader[1158], Michael N. Cantor[1157],

Admin Team Lead

Anne E Justice[1159], Dave J. Carey[1160],

Analysis Team Member

Geetha Chittoor[1159], Navya Shilpa Josyula[1159], Jack A. Kosmicki[1157], Julie E Horowitz[1157], Aris Baras[1157],

Data Collection Member

Matthew C. Gass[1158], Ashish Yadav[1157],

Admin Team Member

Tooraj Mirshahi[1160],

Netherlands Twin Register

Analysis Team Lead

Jouke Jan Hottenga[1161],

Data Collection Lead

Meike Bartels[1161],

Admin Team Lead

Eco (E.J.C.) de geus[1161],

Analysis Team Member

Michel (M.G.) Nivard[1161],

Penn Medicine Biobank

Analysis Team Lead

Anurag Verma[1162], Marylyn D. Ritchie[1162],

Admin Team Lead

Daniel Rader[1162],

Analysis Team Member

Binglan Li[1163], Shefali S Verma[1162], Anastasia Lucas[1162], Yuki Bradford[1162],

Population controls

Data Collection Member

Federico Zara[1164],

Population Controls

Analysis Team Lead

Federico Zara[1165],

Data Collection Lead

Pasquale Striano[1165], Edouad Louis[171], Michel Georges[163], Souad Rahmouni[163], Cynthia M. Bulik[709,1166,1167], Mikael Landén[709,1168], Alfredo Brusco[1169], Giovanni Battista Ferrero[1170],

Admin Team Lead

Francesca Madia[1171], Bengt Fundín[709],

Analysis Team Member

Vincenzo Salpietro[1165], Marcello Scala[1172], Michele Iacomino[1171], Paolo Scudieri[1165], Renata Bocciardi[1165],

Data Collection Member

Carlo Minetti[1165], Antonella Riva[1172], Maria Stella Vari[1171], Myriam Mni[163], Jean-François Rahier[1173], Elisa Giorgio[1174], Diana Carli[1175],

Qatar Genome Program

Analysis Team Lead

Hamdi Mbarek[80],

Data Collection Lead

Said I. Ismail[80],

Analysis Team Member

Chadi Saad[80], Yaser Al-Sarraj[80],

Data Collection Member

Radja Messai Badji[80], Wadha Al-Muftah[80], Asma Al Thani[80], Nahla Afifi[1176],

Study of the COVID-19 host genetics in population of Latvia

Analysis Team Lead

Janis Klovins[1177],

Data Collection Lead

Vita Rovite[1177],

Analysis Team Member

Raimonds Rescenko[1177], Raitis Peculis[1177],

Data Collection Member

Monta Ustinova[1177],

The genetic predisposition to severe COVID-19

Analysis Team Lead

Mari E.K. Niemi[1] ,

Data Collection Lead

Hugo Zeberg[1178,1179],

Analysis Team Member

Mattia Cordioli[31], Sara Pigazzini[5], Lindokuhle Nkambule[166,167],

Data Collection Member

Robert Frithiof[1180], Michael Hultström[1180,1181], Miklos Lipcsey[1180,1182],

UCLA Precision Health COVID-19 Host Genomics Biobank

Analysis Team Lead

Ruth Johnson[1183],

Data Collection Lead

UCLA Health ATLAS and Data Mart Working Group[1184],

Admin Team Lead

Nelson Freimer[1185], Manish J. Butte[1186,1187], Daniel H Geschwind[1188], Bogdan Pasaniuc[1189,1190],

Analysis Team Member

Yi Ding[1191], Alec Chiu[1191], Timothy S Chang[1192], Paul Boutros[1193],

UK 100,000 Genomes Project (Genomics England)

Analysis Team Lead

Loukas Moutsianas[1194,1195],

Data Collection Lead

Mark J Caulfield[1194,1195], Richard H Scott[1194,1196],

Analysis Team Member

Athanasios Kousathanas[95], Dorota Pasko[95], Susan Walker[95], Alex Stuckey[95], Christopher A Odhams[95], Daniel Rhodes[95],

Data Collection Member

Tom Fowler[95], Augusto Rendon[1194,1197], Georgia Chan[95], Prabhu Arumugam[95],

UK Biobank

Analysis Team Lead
Tomoko Nakanishi[1198], Konrad J. Karczewski[3,29], Alicia R. Martin[3,29], Daniel J Wilson[1199], Chris C A Spencer[1200],
Data Collection Lead
Derrick W Crook[1201], David H Wyllie[1201,1202], Anne Marie O'Connell[1203],
Admin Team Lead
J. Brent Richards[53,54,175],
Analysis Team Member
Guillaume Butler-Laporte[54,176], Vincenzo Forgetta[54], Elizabeth G. Atkinson[3,29], Masahiro Kanai[3,29,1204], Kristin Tsuo[3,29,1205], Nikolas Baya[3,29], Patrick Turley[3,29], Rahul Gupta[3,29], Raymond K. Walters[3,29], Duncan S. Palmer[3,29], Gopal Sarma[3,29], Matthew Solomonson[3,29], Nathan Cheng[3,29], Wenhan Lu[3,29], Claire Churchhouse[3,29], Jacqueline I. Goldstein[3,29], Daniel King[3,29], Wei Zhou[3,29], Cotton Seed[3,29], Mark J. Daly[1,2,3], Benjamin M. Neale[3,29], Hilary Finucane[3,29], Sam Bryant[2], F. Kyle Satterstrom[3,29], Gavin Band[1206], Sarah G Earle[1199], Shang-Kuan Lin[1199], Nicolas Arning[1199], Nils Koelling[1200],
Data Collection Member
Jacob Armstrong[1199], Justine K Rudkin[1199],
Admin Team Member
Shawneequa Callier[1207], Sam Bryant[3,29], Caroline Cusick[29],

UK Blood Donors Cohort

Analysis Team Lead
Nicole Soranzo[1208,1209,1210], Jing Hua Zhao[1211],
Data Collection Lead
John Danesh[1211,1212,1213,1214,1215], Emanuele Di Angelantonio[1211,1212,1213,1214],
Analysis Team Member
Adam S. Butterworth[1211,1212,1213,1214],

VA Million Veteran Program (MVP)

Analysis Team Lead
Yan V Sun[1216,1217], Jennifer E Huffman[1218],
Data Collection Lead
Kelly Cho[1219],
Admin Team Lead
Christopher J O'Donnell[1218], Phil Tsao[1220,1221], J. Michael Gaziano[1219],
Analysis Team Member
Gina Peloso[1218,1222],
Data Collection Member
Yuk-Lam Ho[1219],

Val Gardena

Analysis Team Lead
Christian Fuchsberger[209],

Data Collection Lead
Michael Mian[1223],
Data Collection Member
Federica Scaggiante[1224],
Admin Team Member
Cristian Pattaro[209], Peter Pramstaller[209]

GenOMICC/ISARIC4C

Data Collection Lead
J. Kenneth Baillie[60,61,62], Peter J McGuigan[523], Luke Stephen Prockter Moore[524], Marcela Paola Vizcaychipi[524], Kathryn Hall[525], Andy Campbell[526], Ailstair Nichol[527], Geraldine Ward[528], Valerie Joan Page[529], J. Kenneth Baillie[60,61,62], Malcolm G Semple[530], Kayode Adeniji[531], Daniel Agranoff[532], Ken Agwuh[533], Dhiraj Ail[534], Erin L. Aldera[535], Ana Alegria[536], Brian Angus[537], Abdul Ashish[538], Dougal Atkinson[539], Shahedal Bari[540], Gavin Barlow[541], Stella Barnass[542], Nicholas Barrett[543], Christopher Bassford[544], Sneha Basude[545], David Baxter[546], Michael Beadsworth[547], Jolanta Bernatoniene[548], John Berridge[549], Nicola Best[550], Pieter Bothma[551], David Chadwick[552], Robin Brittain-Long[553], Naomi Bulteel[554], Tom Burden[555], Andrew Burtenshaw[556], Vikki Caruth[557], David Chadwick[552], Duncan Chambler[558], Nigel Chee[559], Jenny Child[560], Srikanth Chukkambotla[561], Tom Clark[562], Paul Collini[563], Catherine Cosgrove[564], Jason Cupitt[565], Maria-Teresa Cutino-Moguel[566], Paul Dark[567], Chris Dawson[568], Samir Dervisevic[569], Phil Donnison[570], Sam Douthwaite[571], Andrew Drummond[572], Ingrid DuRand[573], Ahilanadan Dushianthan[574], Tristan Dyer[575], Cariad Evans[563], Chi Eziefula[532], Chrisopher Fegan[576], Adam Finn[577], Duncan Fullerton[578], Sanjeev Garg[579], Atul Garg[580], Effrossyni Gkrania-Klotsas[581], Jo Godden[582], Arthur Goldsmith[583], Clive Graham[584], Elaine Hardy[585], Stuart Hartshorn[586], Daniel Harvey[587], Peter Havalda[588], Daniel B Hawcutt[589], Maria Hobrok[590], Luke Hodgson[591], Anil Hormis[592], Michael Jacobs[593], Susan Jain[594], Paul Jennings[595], Agilan Kaliappan[596], Vidya Kasipandian[597], Stephen Kegg[598], Michael Kelsey[599], Jason Kendall[347], Caroline Kerrison[600], Ian Kerslake[601], Oliver Koch[602], Gouri Koduri[603], George Koshy[604], Shondipon Laha[605], Steven Laird[606], Susan Larkin[607], Tamas Leiner[604], Patrick Lillie[608], James Limb[609], Vanessa Linnett[610], Jeff Little[611], Mark Lyttle[612], Michael MacMahon[347], Emily MacNaughton[613], Ravish Mankregod[614], Huw Masson[615], Elijah Matovu[578], Katherine McCullough[616], Ruth McEwen[617], Manjula Meda[618], Gary Mills[619], Jane Minton[620], Karl Ward[620], Mariyam Mirfenderesky[621], Kavya Mohandas[622], Quen Mok[623], James Moon[624], Elinoor Moore[581], Patrick Morgan[625], Craig Morris[626], Katherine Mortimore[604], Samuel Moses[627], Mbiye Mpenge[628], Rohinton Mulla[629], Michael Murphy[630], Megan Nagel[631], Thapas Nagarajan[632], Mark Nelson[633], Matthew K. O'Shea[634], Igor Otahal[635], Marlies Ostermann[571], Mark Pais[636], Selva Panchatsharam[637], Danai Papakonstantinou[638], Hassan Paraiso[639], Brij Patel[640], Natalie Pattison[641], Justin Pepperell[642], Mark Peters[347], Mandeep Phull[643], Stefania Pintus[644], Jagtur Singh Pooni[645], Frank Post[646], David Price[647], Rachel Prout[648], Nikolas Rae[649], Henrik Reschreiter[650], Tim Reynolds[651], Neil Richardson[652], Mark Roberts[653], Devender Roberts[654], Alistair Rose[655], Guy Rousseau[656], Brendan Ryan[657], Taranprit Saluja[658], Aarti Shah[659], Prad Shanmuga[660], Anil Sharma[661], Anna Shawcross[662], Jeremy Sizer[663], Manu Shankar-Hari[571], Richard Smith[664], Catherine Snelson[665], Nick Spittle[666], Nikki Staines[667], Tom Stambach[668], Richard Stewart[669], Pradeep Subudhi[670], Tamas Szakmany[671], Kate Tatham[672], Jo Thomas[673], Chris Thompson[674], Robert Thompson[347], Ascanio

Tridente[675], Darell Tupper-Carey[551], Mary Twagira[676], Andrew Ustianowski[572], Nick Vallotton[677], Lisa Vincent-Smith[678], Shico Visuvanathan[667], Alan Vuylsteke[679], Sam Waddy[680], Rachel Wake[681], Andrew Walden[682], Ingeborg Welters[547], Tony Whitehouse[665], Paul Whittaker[683], Ashley Whittington[684], Padmasayee Papineni[685], Meme Wijesinghe[686], Martin Williams[347], Lawrence Wilson[617], Sarah Cole[347], Stephen Winchester[687], Martin Wiselka[688], Adam Wolverson[689], Daniel G Wooton[690], Andrew Workman[588], Bryan Yates[691], Peter Young[692],

Analysis Team Member

J. Kenneth Baillie[60,61,62], Rupert Beale[693], Andrew D. Bretherick[62], Mark Caulfield[694,695], Sara Clohisey[60], Max Head Fourman[60], James Furniss[60], Elvina Gountouna[696], Graeme Grimes[62], Chris Haley[60], David Harrison[697], Caroline Hayward[62,696], Sean Keating[698], Lucija Klaric[699], Paul Klenerman[700], Athanasios Kousathanas[701], Andy Law[702], Alison Meynert[699], Jonathan Millar[702], Loukas Moutsianas[701], Erola Pairo-Castineira[60,699], Nick Parkinson[702], Dorota Pasko[701], Chris P Ponting[699], David J. Porteous[703], Konrad Rawlik[702], Anne Richmond[699], Kathy Rowan[704], Clark D Russell[60,705], Richard Scott[694,706], Xia Shen[707,708,709], Barbara Shih[60], Albert Tenesa[60,62,708], Veronique Vitart[62], Susan Walker[701], Bo Wang[702], James F. Wilson[62,708], Yang Wu[710], Jian Yang[711,712], Zhijian Yang[713], Marie Zechner[702], Ranran Zhai[713], Chenqing Zheng[713], J. Kenneth Baillie[60,61,62], Rupert Beale[693], Andrew D. Bretherick[699], Mark Caulfield[694,695], Sara Clohisey[702], Max Head Fourman[702], James Furniss[702], Elvina Gountouna[703], Graeme Grimes[699], Chris Haley[702], David Harrison[704], Caroline Hayward[696,699], Sean Keating[698], Lucija Klaric[699], Paul Klenerman[700], Athanasios Kousathanas[701], Andy Law[702], Alison Meynert[699], Jonathan Millar[702], Loukas Moutsianas[701], Erola Pairo-Castineira[60,699], Nick Parkinson[702], Dorota Pasko[701], Chris P Ponting[699], David J. Porteous[703], Konrad Rawlik[702], Anne Richmond[699], Kathy Rowan[704], Clark D Russell[702,705], Richard Scott[694,706], Xia Shen[707,708,709], Barbara Shih[702], Albert Tenesa[60,62,708], Veronique Vitart[699], Susan Walker[701], Bo Wang[702], James F. Wilson[699,708], Yang Wu[710], Jian Yang[711,712], Zhijian Yang[713], Marie Zechner[702], Ranran Zhai[713], Chenqing Zheng[713], Lisa Norman[714], Riinu Pius[714], Thomas M Drake[714], Cameron J Fairfield[714], Stephen R Knight[714], Kenneth A Mclean[714], Derek Murphy[714], Catherine A Shaw[714], Jo Dalton[715], Michelle Girvan[715], Egle Saviciute[715], Stephanie Roberts[715], Janet Harrison[715], Laura Marsh[715], Marie Connor[715], Sophie Halpin[715], Clare Jackson[715], Carrol Gamble[715], Gary Leeming[716], Andrew Law[60], Murray Wham[717], Sara Clohisey[60], Ross Hendry[60], James Scott-Brown[718],

Data Collection Member

Colin Begg[719], Sara Clohisey[60], Charles Hinds[720], Antonia Ho[721], Peter Horby[722], Julian Knight[723], Lowell Ling[724], David Maslove[725], Danny McAuley[726,727], Johnny Millar[60], Hugh Montgomery[728], Alistair Nichol[729], Peter J.M. Openshaw[730,731], Chris P Ponting[62], Kathy Rowan[697], Malcolm G. Semple[732,733], Manu Shankar-Hari[734], Charlotte Summers[735], Timothy Walsh[61], Lisa Armstrong[736], Hayley Bates[736], Emma Dooks[736], Fiona Farquhar[736], Brigid Hairsine[736], C McParland[736], Sophie Packham[736], Zoe Alldis[737], Raine Astin-Chamberlain[737], Fatima Bibi[737], Jack Biddle[737], Sarah Blow[737], Matthew Bolton[737], Catherine Borra[737], Ruth Bowles[737], Maudrian Burton[737], Yasmin Choudhury[737], David Collier[737], Amber Cox[737], Amy Easthope[737], Patrizia Ebano[737], Stavros Fotiadis[737], Jana Gurasashvili[737], Rosslyn Halls[737], Pippa Hartridge[737], Delordson Kallon[737], Jamila Kassam[737], Ivone Lancoma-Malcolm[737], Maninderpal Matharu[737], Peter May[737], Oliver Mitchelmore[737], Tabitha Newman[737], Mital Patel[737], Jane Pheby[737], Irene Pinzuti[737], Zoe Prime[737], Oleksandra Prysyazhna[737], Julian Shiel[737], Melanie Taylor[737], Carey Tierney[737], Suzanne Wood[737], Anne

Zak[737], Olivier Zongo[737], Miranda Forsey[738], Agilan Kaliappan[738], Anne Nicholson[738], Joanne Riches[738], Mark Vertue[738], Christopher Wasson[523], Stephanie Finn[523], Jackie Green[523], Erin Collins[523], Bernadette King[523], Lina Grauslyte[739], Musarat Hussain[739], Mandeep Phull[739], Tatiana Pogreban[739], Lace Rosaroso[739], Erika Salciute[739], George Franke[739], Joanna Wong[739], Aparna George[739], Louise Akeroyd[740], Shereen Bano[740], Matt Bromley[740], Lucy Gurr[740], Tom Lawton[740], James Morgan[740], Kirsten Sellick[740], Deborah Warren[740], Brian Wilkinson[740], Janet McGowan[740], Camilla Ledgard[740], Amelia Stacey[740], Kate Pye[740], Ruth Bellwood[740], Michael Bentley[740], Maria Hobrok[741], Ronda Loosley[741], Heather McGuinness[741], Helen Tench[741], Rebecca Wolf-Roberts[741], Sian Gibson[742], Amanda Lyle[742], Fiona McNeela[742], Jayachandran Radhakrishnan[742], Alistair Hughes[742], Asifa Ali[743], Megan Brady[743], Sam Dale[743], Annalisa Dance[743], Lisa Gledhill[743], Jill Greig[743], Kathryn Hanson[743], Kelly Holdroyd[743], Marie Home[743], Diane Kelly[743], Ross Kitson[743], Lear Matapure[743], Deborah Melia[743], Samantha Mellor[743], Tonicha Nortcliffe[743], Jez Pinnell[743], Matthew Robinson[743], Lisa Shaw[743], Ryan Shaw[743], Lesley Thomis[743], Alison Wilson[743], Tracy Wood[743], Lee-Ann Bayo[743], Ekta Merwaha[743], Tahira Ishaq[743], Sarah Hanley[743], David Antcliffe[744], Dorota Banach[744], Stephen Brett[744], Phoebe Coghlan[744], Ziortza Fernandez[744], Anthony Gordon[744], Roceld Rojo[744], Sonia Sousa Arias[744], Maie Templeton[744], Rajeev Jha[745], Vinodh Krishnamurthy[745], Lai Lim[745], Rehana Bi[746], Barney Scholefield[746], Lydia Ashton[746], Alison Williams[747], Claire Cheyne[747], Anne Saunderson[747], Angela Allan[748], Felicity Anderson[748], Callum Kaye[748], Jade Liew[748], Jasmine Medhora[748], Teresa Scott[748], Erin Trumper[748], Adriana Botello[748], Petra Polgarova[749], Katerina Stroud[749], Charlotte Summers[749], Eoghan Meaney[749], Megan Jones[749], Anthony Ng[749], Shruti Agrawal[749], Nazima Pathan[749], Deborah White[749], Esther Daubney[749], Kay Elston[749], Robert Parker[750], Amie Reddy[750], Ian Turner-Bone[750], Laura Wilding[750], Peter Harding[751], Reni Jacob[752], Cathy Jones[752], Craig Denmade[752], Maria Croft[753], Ian White[753], Rajeev Jha[745], Vinodh Krishnamurthy[745], Li Lim[745], Denise Griffin[754], Nycola Muchenje[754], Mcdonald Mupudzi[754], Richard Partridge[754], Jo-Anna Conyngham[754], Rachel Thomas[754], Mary Wright[754], Maria Alvarez Corral[754], Victoria Bastion[663], Daphene Clarke[663], Beena David[663], Harriet Kent[663], Rachel Lorusso[663], Gamu Lubimbi[663], Sophie Murdoch[663], Melchizedek Penacerrada[663], Alastair Thomas[663], Jennifer Valentine[663], Ana Vochin[663], Retno Wulandari[663], Brice Djeugam[663], Joy Dawson[755], Sweyn Garrioch[755], Melanie Tolson[755], Jonathan Aldridge[755], Laura Gomes de Almeida Martins[524], Jaime Carungcong[524], Sarah Beavis[756], Katie Dale[756], Rachel Gascoyne[756], Joanne Hawes[756], Kelly Pritchard[756], Lesley Stevenson[756], Amanda Whileman[756], Anne Cowley[757], Judith Highgate[757], Rikki Crawley[758], Abigail Crew[758], Mishell Cunningham[758], Allison Daniels[758], Laura Harrison[758], Susan Hope[758], Ken Inweregbu[758], Sian Jones[758], Nicola Lancaster[758], Jamie Matthews[758], Alice Nicholson[758], Gemma Wray[758], Leonie Benham[759], Zena Bradshaw[759], Joanna Brown[759], Melanie Caswell[759], Jason Cupitt[759], Sarah Melling[759], Stephen Preston[759], Nicola Slawson[759], Emma Stoddard[759], Scott Warden[759], Edward Combes[760], Teishel Joefield[760], Sonja Monnery[760], Valerie Beech[760], Sallyanne Trotman[760], Bridget Hopkins[761], James Scriven[761], Laura Thrasyvoulou[761], Heather Willis[761], Susan Anderson[762], Janine Birch[762], Emma Collins[762], Kate Hammerton[762], Ryan O'Leary[762], Caroline Abernathy[763], Louise Foster[763], Andrew Gratrix[763], Vicky Martinson[763], Priyai Parkinson[763], Elizabeth Stones[763], Llucia Carbral-Ortega[764], Ritoo Kapoor[765], David Loader[765], Karen Castle[765], Craig Brandwood[766], Lara Smith[766], Richard Clark[766], Katie Birchall[766], Laurel Kolakaluri[766], Deborah Baines[766], Anila Sukumaran[766], Isheunesu Mapfunde[525], Megan Meredith[767], Lucy Morris[767], Lucy Ryan[767], Amy Clark[767], Julia Sampson[767], Cecilia Peters[767], Martin Dent[767], Margaret Langley[767], Saima Ashraf[767], Shuying Wei[767],

Angela Andrew[767], Manish Chablani[768], Amy Kirkby[768], Kimberley Netherton[768], Michelle Bates[769], Jo Dasgin[769], Jaspret Gill[769], Annette Nilsson[769], James Scriven[769], Elena Apetri[770], Cathrine Basikolo[770], Bethan Blackledge[770], Laura Catlow[770], Bethan Charles[770], Paul Dark[770], Reece Doonan[770], Jade Harris[770], Alice Harvey[770], Daniel Horner[770], Karen Knowles[770], Stephanie Lee[770], Diane Lomas[770], Chloe Lyons[770], Tracy Marsden[770], Danielle McLaughlan[770], Liam McMorrow[770], Jessica Pendlebury[770], Jane Perez[770], Maria Poulaka[770], Nicola Proudfoot[770], Melanie Slaughter[770], Kathryn Slevin[770], Melanie Taylor[770], Vicky Thomas[770], Danielle Walker[770], Angiy Michael[770], Matthew Collis[770], Martyn Clark[771], Martina Coulding[771], Edward Jude[771], Jacqueline McCormick[771], Oliver Mercer[771], Darsh Potla[771], Hafiz Rehman[771], Heather Savill[771], Victoria Turner[771], Miriam Davey[772], David Golden[772], Rebecca Seaman[772], Jodie Hunt[773], Joy Dearden[773], Emma Dobson[773], Andy Drummond[773], Michelle Mulcahy[773], Sheila Munt[773], Grainne O'Connor[773], Jennifer Philbin[773], chloe Rishton[773], Redmond Tully[773], Sarah Winnard[773], Lenka Cagova[774], Adama Fofano[774], Lucie Garner[774], Helen Holcombe[774], Sue Mepham[774], Alice Michael Mitchell[774], Lucy Mwaura[774], K Praman[774], Alain Vuylsteke[774], Julie Zamikula[774], Miriam Davey[772], David Golden[772], Rebecca Seaman[772], Georgia Bercades[775], David Brealey[775], Ingrid Hass[775], Niall MacCallum[775], Gladys Martir[775], Eamon Raith[775], Anna Reyes[775], Deborah Smyth[775], Abigail Taylor[776], Rachel Anne Hughes[776], Helen Thomas[776], Alun Rees[776], Michaela Duskova[776], Janet Phipps[776], Suzanne Brooks[776], Michelle Edwards[776], Peter Alexander[777], Schvearn Allen[777], Joanne Bradley-Potts[777], Craig Brantwood[777], Jasmine Egan[777], Timothy Felton[777], Grace Padden[777], Luke Ward[777], Stuart Moss[777], Susannah Glasgow[777], Kate Beesley[778], Sarah Board[778], Agnieszka Kubisz-Pudelko[778], Alison Lewis[778], Jess Perry[778], Lucy Pippard[778], Di Wood[778], Clare Buckley[778], Alison Brown[779], Jane Gregory[779], Susan O'Connell[779], Tim Smith[779], Zakaula Belagodu[780], Bridget Fuller[780], Anca Gherman[780], Olumide Olufuwa[780], Remi Paramsothy[780], Carmel Stuart[780], Naomi Oakley[780], Charlotte Kamundi[780], David Tyl[780], Katy Collins[780], Pedro Silva[780], June Taylor[780], Laura King[780], Charlotte Coates[780], Maria Crowley[780], Phillipa Wakefield[780], Jane Beadle[780], Laura Johnson[780], Janet Sargeant[780], Madeleine Anderson[780], Catherine Jardine[781], Dewi Williams[781], Victoria Parris[782], Sheena Quaid[782], Ekaterina Watson[782], Julie Melville[783], Jay Naisbitt[783], Rosane Joseph[783], Maria Lazo[783], Olivia Walton[783], Alan Neal[783], Michaela Hill[784], Thogulava Kannan[784], Wild Laura[784], Elizabeth Allan[785], Kate Darlington[785], Ffyon Davies[785], Jack Easton[785], Sumit Kumar[785], Richard Lean[785], Daniel Menzies[785], Richard Pugh[785], Xinyi Qiu[785], Llinos Davies[785], Hannah Williams Williams[785], Jeremy Scanlon[785], Gwyneth Davies[785], Callum Mackay[785], Joannne Lewis[785], Stephanie Rees[785], Samantha Coetzee[786], Alistair Gales[786], Igor Otahal[786], Meena Raj[786], Craig Sell[786], Helen Langton[787], Rachel Prout[787], Malcolm Watters[787], Catherine Novis[787], Gill Arbane[788], Aneta Bociek[788], Sara Campos[788], Neus Grau[788], Tim Owen Jones[788], Rosario Lim[788], Martina Marotti[788], Marlies Ostermann[788], Manu Shankar-Hari[788], Christopher Whitton[788], Anthony Barron[789], Ciara Collins[789], Sundeep Kaul[789], Heather Passmore[789], Claire Prendergast[789], Anna Reed[789], Paula Rogers[789], Rajvinder Shokkar[789], Meriel Woodruff[789], Hayley Middleton[789], Oliver Polgar[789], Claire Nolan[789], Vicky Thwaites[789], Kanta Mahay[789], Chunda Sri-Chandana[790], Joslan Scherewode[790], Lorraine Stephenson[790], Sarah Marsh[790], Hollie Bancroft[791], Mary Bellamy[791], Margaret Carmody[791], Jacqueline Daglish[791], Faye Moore[791], Joanne Rhodes[791], Mirriam Sangombe[791], Salma Kadiri[791], James Scriven[791], Amanda Ayers[792], Wendy Harrison[792], Julie North[792], Anna Cavazza[793], Maeve Cockrell[793], Eleanor Corcoran[793], Maria Depante[793], Clare Finney[793], Ellen Jerome[793], Mark McPhail[793], Monalisa Nayak[793], Harriet Noble[793], Kevin O'Reilly[793], Evita Pappa[793], Rohit Saha[793], Sian Saha[793], John Smith[793],

Abigail Knighton[793], Mandy Gill[794], Paul Paul[794], Valli Ratnam[794], Sarah Shelton[794], Inez Wynter[794], David Baptista[795], Rebecca Crowe[795], Rita Fernandes[795], Rosaleen Herdman-Grant[795], Anna Joseph[795], Adam Loveridge[795], India McKenley[795], Eriko Morino[795], Andres Naranjo[795], Richard Simms[795], Kathryn Sollesta[795], Andrew Swain[795], Harish Venkatesh[795], Jacyntha Khera[795], Jonathan Fox[795], Russell Barber[796], Claire Hewitt[796], Annette Hilldrith[796], Karen Jackson-Lawrence[796], Sarah Shepardson[796], Maryanne Wills[796], Susan Butler[796], Silvia Tavares[796], Amy Cunningham[796], Julia Hindale[796], Sarwat Arif[796], Linsha George[797], Sophie Twiss[797], David Wright[797], Maureen Holland[798], Natalie Keenan[798], Marc Lyons[798], Helen Wassall[798], Chris Marsh[798], Mervin Mahenthran[798], Emma Carter[798], Thomas Kong[798], Oluronke Adanini[799], Nikhil Bhatia[799], Maines Msiska[799], Miranda Forsey[738], Agilan Kaliappan[738], Anne Nicholson[738], Joanne Riches[738], Mark Vertue[738], Louise Mew[800], Esther Mwaura[800], Richard Stewart[800], Felicity Williams[800], Lynn Wren[800], Sara-Beth Sutherland[800], Ceri Battle[801], Elaine Brinkworth[801], Rachel Harford[801], Carl Murphy[801], Luke Newey[801], Tabitha Rees[801], Marie Williams[801], Sophie Arnold[801], David Brealey[802], John Hardy[802], Henry Houlden[802], Eleanor Moncur[802], Eamon Raith[802], Ambreen Tariq[802], Arianna Tucci[802], Karen Convery[803], Deirdre Fottrell-Gould[803], Lisa Hudig[803], Jocelyn Keshet-price[803], Georgina Randell[803], Katie Stammers[803], Marwa Abdelrazik[804], Dhanalakshmi Bakthavatsalam[804], Munzir Elhassan[804], Arunkumar Ganesan[804], Anne Haldeos[804], Jeronimo Moreno-Cuesta[804], Dharam Purohit[804], Rachel Vincent[804], Kugan Xavier[804], kumar Rohit[805], Frater Alasdair[804], Malik Saleem[804], Carter David[804], Jenkins Samuel[804], Zoe Lamond[804], Wall Alanna[804], Bryan Yates[806], Jessica Reynolds[806], Helen Campbell[806], Maria Thompsom[806], Steve Dodds[806], Stacey Duffy[806], Deborah Butcher[807], Susie O'Sullivan[807], Nicola Butterworth-Cowin[807], Bethan Deacon[808], Meg Hibbert[808], Carla Pothecary[808], Dariusz Tetla[808], Chrsitopher Woodford[808], Latha Durga[808], Gareth Kennard-Holden[808], Laura Ortiz-Ruiz de Gordoa[809], Emily Peasgood[809], Claire Phillips[809], Denise Skinner[810], Jane Gaylard[810], Dee Mullan[810], Julie Newman[810], Ellie Davies[811], Lisa Roche[811], Sonia Sathe[811], Lutece Brimfield[812], Zoe Daly[812], David Pogson[812], Steve Rose[812], Amy Collins[813], Waqas Khaliq[813], Estefania Treus Gude[813], Louise Allen[814], Eva Beranova[814], Nikki Crisp[814], Joanne Deery[814], Tracy Hazelton[814], Alicia Knight[814], Carly Price[814], Sorrell Tilbey[814], Salah Turki[814], Sharon Turney[814], Julian Giles[815], Simon Booth[815], Gillian Bell[816], Katy English[816], Amro Katary[816], Louise Wilcox[816], Rachael Campbell[817], Noreen Clarke[817], Jonathan Whiteside[817], Mairi Mascarenhas[817], Avril Donaldson[817], Joanna Matheson[817], Fiona Barrett[817], Marianne O'Hara[817], Laura Okeefe[817], Clare Bradley[817], Dawn Collier[818], Anil Hormis[818], Rachel Walker[818], Victoria Maynard[818], Tahera Patel[819], Matthew Smith[819], Srikanth Chukkambotla[819], Aayesha Kazi[819], Janice Hartley[819], Joseph Dykes[819], Muhammad Hijazi[819], Sarah Keith[819], Meherunnisa Khan[819], Janet Ryan-Smith[819], Philippa Springle[819], Jacqueline Thomas[819], Nick Truman[819], Samuel Saad[819], Dabheoc Coleman[819], Christopher Fine[819], Roseanna Matt[819], Bethan Gay[819], Jack Dalziel[819], Syamlan Ali[819], Drew Goodchild[819], Rhiannan Harling[819], Ravi Bhatterjee[819], Wendy Goddard[819], Chloe Davison[819], Stephen Duberly[819], Jeanette Hargreaves[819], Rachel Bolton[819], Shondipon Laha[820], Mark Verlander[820], Alexandra Williams[820], Helen Blackman[821], Ben Creagh-Brown[821], Sinead Donlon[821], Natalia Michalak-Glinska[821], Sheila Mtuwa[821], Veronika Pristopan[821], Armorel Salberg[821], Eleanor Smith[821], Sarah Stone[821], Charles Piercy[821], Jerik Verula[821], Dorota Burda[821], Rugia Montaser[821], Lesley Harden[821], Irving Mayangao[821], Cheryl Marriott[821], Paul Bradley[821], Celia Harris[821], Joshua Cooper[822], Cheryl Finch[822], Sarah Liderth[822], Alison Quinn[822], Natalia Waddington[822], Katy Fidler[823], Emma Tagliavini[823], Kevin Donnelly[823], Lynn Abel[824], Michael Brett[824], Brian Digby[824], Lisa Gemmell[824], James Hornsby[824], Patrick MacGoey[824], Pauline O'Neil[824],

Richard Price[824], Natalie Rodden[824], Kevin Rooney[824], Radha Sundaram[824], Nicola Thomson[824], Rebecca Flanagan[825], Gareth Hughes[825], scott Latham[825], Emma McKenna[825], Jennifer Anderson[825], Robert Hull[825], Kat Rhead[825], Debbie Branney[826], Jordan Frankham[826], Sally Pitts[826], Nigel White[826], Daniele Cristiano[827], Natalie Dormand[827], Zohreh Farzad[827], Mahitha Gummadi[827], Kamal Liyanage[827], Brijesh V Patel[828], Sara Salmi[827], Geraldine Sloane[827], Vicky Thwaites[827], Mathew Varghese[827], Anelise C Zborowski[827], Sarah Bean[829], Karen Burt[829], Michael Spivey[829], Christine Eastgate-Jackson[830], Helder Filipe[830], Daniel Martin[830], Amitaa Maharajh[830], Sara Mingo Garcia[830], Mark De Neef[830], Bethan Deacon[831], Ceri Lynch[831], Carla Pothecary[831], Lisa Roche[831], Gwenllian Sera Howe[831], Jayaprakash Singh[831], Keri Turner[831], Hannah Ellis[831], Natalie Stroud[831], Shiney Cherian[832], Sean Cutler[832], Anne Emma Heron[832], Anna Roynon-Reed[832], Tamas Szakmany[832], Gemma Williams[832], Owen Richards[832], Yusuf Cheema[832], Norfaizan Ahmad[833], Joann Barker[833], Kris Bauchmuller[833], Sarah Bird[833], Kay Cawthron[833], Kate Harrington[833], Yvonne Jackson[833], Faith Kibutu[833], Becky Lenagh[833], Shamiso Masuko[833], Gary H Mills[833], Ajay Raithatha[833], Matthew Wiles[833], Jayne Willson[833], Helen Newell[833], Alison Lye[833], Lorenza Nwafor[833], Claire Jarman[833], Sarah Rowland-Jones[833], David Foote[833], Joby Cole[833], Roger Thompson[833], James Watson[833], Lisa Hesseldon[833], Irene Macharia[833], Luke Chetam[833], Jacqui Smith[833], Amber Ford[833], Samantha Anderson[833], Kathryn Birchall[833], Kay Housley[833], Sara Walker[833], Leanne Milner[833], Helena Hanratty[833], Helen Trower[833], Patrick Phillips[833], Simon Oxspring[833], Ben Donne[833], Emily Bevan[834], Jane Martin[834], Dawn Trodd[834], Geoff Watson[834], Caroline Wrey Brown[834], Lara Bunni[835], Claire Jennings[835], Monica Latif[835], Rebecca Marshall[835], Gayathri Subramanian[835], Nageswar Bandla[836], Minnie Gellamucho[836], Michelle Davies[836], Christopher Thompson[836], Laura Ortiz-Ruiz de Gordoa[809], Emily Peasgood[809], Claire Phillips[809], Denise Skinner[810], Jane Gaylard[810], Dee Mullan[810], Julie Newman[810], Phil Donnison[837], Fiona Trim[837], Beena Eapen[837], Cecilia Ahmed[838], Balvinder Baines[838], Sarah Clamp[838], Julie Colley[838], Risna Haq[838], Anne Hayes[838], Jonathan Hulme[838], Samia Hussain[838], Sibet Joseph[838], Rita Kumar[838], Zahira Maqsood[838], Manjit Purewal[838], Dr Ben Chandler[839], Kerry Elliott[839], Janine Mallinson[839], Alison Turnbull[839], Kathy Dent[840], Elizabeth Horsley[840], Muhmmad Nauman Akhtar[840], Sandra Pearson[840], Dorota Potoczna[840], Sue Spencer[840], Hayley Blakemore[841], Borislava Borislavova[841], Beverley Faulkner[841], Emma Gendall[841], Elizabeth Goff[841], Kati Hayes[841], Matt Thomas[841], Ruth Worner[841], Kerry Smith[841], Deanna Stephens[841], Carlos Castro Delgado[842], Deborah Dawson[842], Lijun Ding[842], Georgia Durrant[842], Obiageri Ezeobu[842], Sarah Farnell-Ward[842], Abiola Harrison[842], Rebecca Kanu[842], Susannah Leaver[842], elena Maccacari[842], Soumendu Manna[842], Romina Pepermans Saluzzio[842], Joana Queiroz[842], Tinashe Samakomva[842], Christine Sicat[842], Joana Texeira[842], Edna Fernandes Da Gloria[842], Ana Lisboa[842], John Rawlins[842], Jisha Mathew[842], Ashley Kinch[842], William James Hurt[842], Nirav Shah[842], Victoria Clark[842], Maria Thanasi[842], Nikki Yun[842], Kamal Patel[842], Alison Brown[843], Vikki Crickmore[843], Gabor Debreceni[843], Joy Wilkins[843], Liz Nicol[843], Iona Burn[844], Geraldine Hambrook[844], Katarina Manso[844], Ruth Penn[844], Pradeep Shanmugasundaram[844], Julie Tebbutt[844], Danielle Thornton[844], Anthony Rostron[845], Alistair Roy[845], Lindsey Woods[845], Sarah Cornell[845], Fiona Wakinshaw[845], Kimberley Rogerson[845], Jordan Jarmain[845], Peter Anderson[846], Katie Archer[846], Karen Austin[846], caroline Davis[846], Alison Durie[846], Olivia Kelsall[846], Jessica Thrush[846], Charlie Vigurs[846], Laura Wild[846], Hannah-Louise Wood[846], Helen Tranter[846], Alison Harrison[846], Nicholas Cowley[846], Michael McAlindon[846], Andrew Burtenshaw[846], Stephen Digby[846], Emma Low[846], Aled Morgan[846], Naiara Cother[846], Tobias Rankin[846], Sarah Clayton[846], Alex McCurdy[846], Suzanne Allibone[847], Roman Mary-Genetu[847], Vidya Kasipandian[847], Amit

Patel[847], Ainhi Mac[847], Anthony Murphy[847], Parisa Mahjoob[847], Roonak Nazari[847], Lucy Worsley[847], Andrew Fagan[847], Inthakab Ali Mohamed Ali[848], Karen Beaumont[848], Mark Blunt[848], Zoe Coton[848], Hollie Curgenven[848], Mohamed Elsaadany[848], Kay Fernandes[848], Sameena Mohamed Ally[848], Harini Rangarajan[848], Varun Sarathy[848], Sivarupan Selvanayagam[848], Dave Vedage[848], Matthew White[848], Jaime Fernandez-Roman[849], David O. Hamilton[849], Emily Johnson[849], Brian Johnston[849], Maria Lopez Martinez[849], Suleman Mulla[849], David Shaw[849], Alicia A.C. Waite[849], Victoria Waugh[849], Ingeborg D. Welters[849], Karen Williams[849], Thomas Bemand[850], Ethel Black[850], Arnold Dela Rosa[850], Ryan Howle[850], Shaman Jhanji[850], Ravishankar Rao Baikady[850], Kate Colette Tatham[850], Benjamin Thomas[850], Matthew Halkes[851], Pauline Mercer[851], Lorraine Thornton[851], West Joe[852], Baird Tracy[852], Ruddy Jim[852], Waqas Khaliq[853], Rosie Reece-Anthony[853], Mark Birt[854], Amanda Cowton[854], Andrea Kay[854], Melanie Kent[854], Kathryn Potts[854], Ami Wilkinson[854], Suzanne Naylor[854], Ellen Brown[854], Michele Clark[855], Sarah Purvis[855], Jade Cole[856], Michelle Davies[856], Rhys Davies[856], Donna Duffin[856], Helen Hill[856], Ben Player[856], Emma Thomas[856], Angharad Williams[856], Claire Marie Beith[857], Karen Black[857], Suzanne Clements[857], Alan Morrison[857], Dominic Strachan[857], Margaret Taylor[857], Michelle Clarkson[857], Stuart D'Sylva[857], Kathryn Norman[857], Tina Coventry[858], Susan Fowler[858], Michael MacMahon[858], Amanda McGregor[858], Ailbhe Brady[859], Rebekah Chan[859], Jeff Little[859], Shane McIvor[859], Helena Prady[859], Helen Whittle[859], Bijoy Mathew[859], Melanie Clapham[860], Rosemary Harper[860], Una Poultney[860], Polly Rice[860], Tim Smith[860], Rachel Mutch[860], Yolanda Baird[861], Aaron Butler[861], Indra Chadbourn[861], Linda Folkes[861], Heather Fox[861], Amy Gardner[861], Raquel Gomez[861], Gillian Hobden[861], Luke Hodgson[861], Kirsten King[861], Michael Margarson[861], Tim Martindale[861], Emma Meadows[861], Dana Raynard[861], Yvette Thirlwall[861], David Helm[861], Jordi Margalef[861], Sandra Greer[862], Karen Shuker[862], Ascanio Tridente[862], Sara Smuts[526], Joseph Duffield[526], Oliver Smith[526], Lewis Mallon[526], Watkins Claire[526], Isobel Birkinshaw[863], Joseph Carter[863], Kate Howard[863], Joanne Ingham[863], Rosie Joy[863], Harriet Pearson[863], Samantha Roche[863], Zoe Scott[863], Ellen Knights[864], Alicia Price[864], Alice Thomas[864], Chris Thorpe[864], Azmerelda Abraheem[865], Peter Bamford[865], Kathryn Cawley[865], Charlie Dunmore[865], Maria Faulkner[865], Rumanah Girach[865], Helen Jeffrey[865], Rhianna Jones[865], Emily London[865], Imrun Nagra[865], Farah Nasir[865], Hannah Sainsbury[865], Clare Smedley[865], Reena Khade[866], Ashok Sundar[866], George Tsinaslanidis[866], Teresa Behan[867], Caroline Burnett[867], Jonathan Hatton[867], Elaine Heeney[867], Atideb Mitra[867], Maria Newton[867], Rachel Pollard[867], Rachael Stead[867], Jenny Birch[868], Laura Bough[868], Josie Goodsell[868], Rebecca Tutton[868], Patricia Williams[868], Sarah Williams[868], Barbara Winter-Goodwin[868], Anne Cowley[757], Judith Highgate[757], Fiona Auld[869], Joanne Donnachie[869], Ian Edmond[869], Lynn Prentice[869], Nikole Runciman[869], Dario Salutous[869], Lesley Symon[869], Anne Todd[869], Patricia Turner[869], Abigail Short[869], Laura Sweeney[869], Euan Murdoch[869], Dhaneesha Senaratne[869], Karen Burns[870], A Higham[870], Taya Anderson[871], Dan Hawcutt[871], Laura O'Malley[871], Laura Rad[871], Naomi Rogers[871], Paula Saunderson[871], Kathryn Sian Allison[871], Deborah Afolabi[871], jennifer whitbread[871], Dawn jones[871], Rachael Dore[871], Liana Lankester[872], Nikitas Nikitas[872], Colin Wells[872], Bethan Stowe[872], Kayleigh Spencer[872], Susanne Cathcart[873], Katharine Duffy[873], Alex Puxty[873], Kathryn Puxty[873], Lynne Turner[873], Jane Ireland[873], Gary Semple[873], Peter Barry[874], Paula Hilltout[875], Jayne Evitts[875], Amanda Tyler[875], Joanne Waldron[875], Val Irvine[876], Benjamin Shelley[876], Olugbenga Akinkugbe[877], Alasdair Bamford[877], Emily Beech[877], Holly Belfield[877], Michael Bell[877], Charlene Davies[877], Gareth A. L. Jones[877], Tara McHugh[877], Hamza Meghari[877], Lauran O'Neill[877], Mark J. Peters[877], Samiran Ray[877], Ana Luisa Tomas[877], Amy Easthope[878], Claire Gorman[878], Abhinav Gupta[878], Elizabeth

Timlick[878], Rebecca Brady[878], Stephen Bonner[879], Keith Hugill[879], Jessica Jones[879], Steven Liggett[879], Archana Bashyal[880], Neil Davidson[880], Paula Hutton[880], Stuart McKechnie[880], Jean Wilson[880], Neil Flint[881], Patel Rekha[881], Dawn Hales[881], Carina Cruz[882], Natalie Pattison[882], Shameer Gopal[883], Nichola Harris[883], Victoria Lake[883], Stella Metherell[883], Elizabeth Radford[883], Ian Clement[884], Bijal Patel[884], A Gulati[884], Carole Hays[884], K Webster[884], Anne Hudson[884], A Webster[884], E Stephenson[884], L McCormack[884], V Slater[884], R Nixon[884], H Hanson[884], M fearby[884], S Kelly[884], V Bridgett[884], P Robinson[884], Christine Almaden-Boyle[885], Pauline Austin[885], Louise Cabrelli[885], Stephen Cole[885], Matt Casey[885], Susan Chapman[885], Stephen Cole[885], Clare Whyte[885], Adam Brayne[886], Emma Fisher[886], Jane Hunt[886], Peter Jackson[886], Duncan Kaye[886], Nicholas Love[886], Juliet Parkin[886], Victoria Tuckey[886], Lynne van Koutrik[886], Sasha Carter[886], Benedict Andrew[886], Louise Findlay[886], Katie Adams[886], Michelle Bruce[887], Karen Connolly[887], Tracy Duncan[887], Helen T-Michael[887], Gabriella Lindergard[887], Samuel Hey[887], Claire Fox[887], Jordan Alfonso[887], Laura Jayne Durrans[887], Jacinta Guerin[887], Bethan Blackledge[887], Jade Harris[887], Martin Hruska[887], Ayaa Eltayeb[887], Thomas Lamb[887], Tracey Hodgkiss[887], Lisa Cooper[887], Joanne Rothwell[887], Catherine Dennis[888], Alastair McGregor[888], Victoria Parris[888], Sinduya Srikaran[888], Anisha Sukha[888], Kim Davies[889], Linda O'Brien[889], Zohra Omar[889], Igor Otahal[889], Emma Perkins[889], Tracy Lewis[889], Isobel Sutherland[889], Hollie Brooke[890], Sarah Buckley[890], Jose Cebrian Suarez[890], Ruth Charlesworth[890], Karen Hansson[890], John Norris[890], Alice Poole[890], Alastair Rose[890], Rajdeep Sandhu[890], Brendan Sloan[890], Elizabeth Smithson[890], Muthu Thirumaran[890], Veronica Wagstaff[890], Alexandra Metcalfe[890], Julie Camsooksai[891], Charlotte Humphrey[891], Sarah Jenkins[891], Henrik Reschreiter[891], Beverley Wadams[891], Yasmin DeAth[891], Colene Adams[892], Anita Agasou[892], Tracie Arden[893], Amy Bowes[892], Pauline Boyle[892], Mandy Beekes[894], Heather Button[895], Nigel Capps[896], Mandy Carnahan[892], Anne Carter[892], Danielle Childs[892], Denise Donaldson[894], Kelly Hard[892], Fran Hurford[897], Yasmin Hussain[892], Ayesha Javaid[895], James Jones[894], Sanal Jose[896], Michael Leigh[892], Terry Martin[894], Helen Millward[898], Nichola Motherwell[894], Rachel Rikunenko[892], Jo Stickley[892], Julie Summers[894], Louise Ting[894], Helen Tivenan[892], Louise Tonks[898], Rebecca Wilcox[892], Maria Bokhari[899], Vanessa Linnett[899], Rachael Lucas[899], Wendy McCormick[899], Jenny Ritzema[899], Amanda Sanderson[899], Helen Wild[899], Nicola Baxter[900], Steven Henderson[900], Sophie Kennedy-Hay[900], Christopher McParland[900], Laura Rooney[900], Malcolm Sim[900], Gordan McCreath[900], Mark Brunton[901], Jess Caterson[901], Holly Coles[901], Matthew Frise[901], Sabi Gurung Rai[901], Nicola Jacques[901], Liza Keating[901], Emma Tilney[901], Shauna Bartley[901], Parminder Bhuie[901], Charlotte Downes[902], Kathleen Holding[902], Katie Riches[902], Mary Hilton[902], Mel Hayman[902], Deepak Subramanian[902], Priya Daniel[902], Letizia Zitter[903], Sarah Benyon[903], Suzie Marriott[903], Linda Park[903], Samantha Keenan[903], Elizabeth Gordon[903], Helen Quinn[903], Kizzy Baines[903], Gillian Andrew[904], J. Kenneth Baillie[904], Lucy Barclay[904], Marie Callaghan[904], Rachael Campbell[904], Sarah Clark[904], Dave Hope[904], Lucy Marshall[904], Corrienne McCulloch[904], Kate Briton[904], Jo Singleton[904], Sohphie Birch[904], Andrew Higham[905], Kerry Simpson[905], Jayne Craig[905], Carrie Demetriou[906], Charlotte Eckbad[906], Sarah Hierons[906], Lucy Howie[906], Sarah Mitchard[906], Lidia Ramos[906], Alfredo Serrano-Ruiz[906], Katie White[906], Fiona Kelly[906], Vishal Amin[907], Elena Anastasescu[907], Vikram Anumakonda[907], Komala Karthik[907], Rizwana Kausar[907], Karen Reid[907], Jacqueline Smith[907], Janet Imeson-Wood[907], Arianna Bellini[908], Jade Bryant[908], Anton Mayer[908], Amy Pickard[908], Nicholas Roe[908], Jason Sowter[908], Alex Howlett[908], Kristine Criste[909], Rebecca Cusack[909], Kim Golder[909], Hannah Golding[909], Oliver Jones[909], Samantha Leggett[909], Michelle Male[909], Martyna Marani[909], Kirsty Prager[909], Toran Williams[909], Belinda Roberts[909], Karen Salmon[909], Prisca Gondo[910], B

Hadebe[910], Abdul Kayani[910], Bridgett Masunda[910], Ashar Ahmed[911], Anna Morris[911], Srinivas Jakkula[911], Kate Long[912], Simon Whiteley[912], Elizabeth Wilby[912], Bethan Ogg[912], Sam Moultrie[747], M Odam[747], Jeremy Bewley[913], Zoe Garland[913], Lisa Grimmer[913], Bethany Gumbrill[913], Rebekah Johnson[913], Katie Sweet[913], Denise Webster[913], Georgia Efford[913], Sara Bennett[914], Emma Goodwin[914], Matthew Jackson[914], Alissa Kent[914], Clare Tibke[914], Wiesia Woodyatt[914], Ahmed Zaki[914], Amelia Daniel[915], Joanne Finn[915], Rajnish Saha[915], Nikki Staines[915], Amy Easthope[915], Pamela Bremmer[528], J Allan[916], T Geary[916], Gordon Houston[916], A Meikle[916], P O'Brien[916], Dina Bell[917], Rosalind Boyle[917], Katie Douglas[917], Lynn Glass[917], Emma Lee[917], Liz Lennon[917], Austin Rattray[917], Rob Charnock[918], Denise McFarland[918], Denise Cosgrove[918], Ben Attwood[919], Penny Parsons[919], Siobhain Carmody[529], Metod Oblak[920], Monica Popescu[920], Mini Thankachen[920], Rosie Baruah[921], Sheila Morris[921], Susie Ferguson[921], Amy Shepherd[921], Abdelhakim Altabaibeh[922], Ana Alvaro[922], Kayleigh Gilbert[922], Louise Ma[922], Loreta Mostoles[922], Chetan Parmar[922], Kathryn Simpson[922], Champa Jetha[922], Lauren Booker[922], Anezka Pratley[922], Tracey Cosier[923], Gemma Millen[923], Neil Richardson[923], Natasha Schumacher[923], Heather Weston[923], James Rand[923], Colin Begg[719], Sara Clohisey[702], Charles Hinds[695], Antonia Ho[924], Peter Horby[925], Julian Knight[700], Lowell Ling[926], David Maslove[927], Danny McAuley[726,727], Johnny Millar[702], Hugh Montgomery[928], Alistair Nichol[929], Peter J.M. Openshaw[730,731], Chris P Ponting[699], Kathy Rowan[704], Malcolm G. Semple[732,733], Manu Shankar-Hari[930], Charlotte Summers[931], Timothy Walsh[698], Peter JM Openshaw[730], Beatrice Alex[718], Benjamin Bach[718], Wendy S Barclay[932], Debby Bogaert[705], Meera Chand[933], Graham S Cooke[934], Annemarie B Docherty[714], Jake Dunning[935], Ana da Silva Filipe[936], Tom Fletcher[937], Christoper A Green[634], Ewen M Harrison[714], Julian A Hiscox[938], Antonia Ying Wai Ho[936], Peter W Horby[939], Samreen Ijaz[940], Saye Khoo[941], Paul Klenerman[942], Andrew Law[943], Wei Shen Lim[944], Alexander J Mentzer[945], Laura Merson[946], Alison M Meynert[717], Mahdad Noursadeghi[947], Shona C Moore[948], Massimo Palmarini[936], William A Paxton[948], Georgios Pollakis[948], Nicholas Price[949], Andrew Rambaut[950], David L Robertson[936], Clark D Russell[705], Vanessa Sancho-Shimizu[951], Janet T Scott[936], Thushan de Silva[952], Louise Sigfrid[946], Tom Solomon[530], Shiranee Sriskandan[934], David Stuart[953], Charlotte Summers[954], Richard S Tedder[955], Emma C Thomson[936], AA Roger Thompson[956], Ryan S Thwaites[730], Lance CW Turtle[530], Rishi K Gupta[957], Carlo Palmieri[958], Olivia V Swann[959], Maria Zambon[935], Marc-Emmanuel Dumas[960], Julian L Griffin[960], Zoltan Takats[960], Kanta Chechi[961], Petros Andrikopoulos[960], Anthonia Osagie[960], Michael Olanipekun[960], Sonia Liggi[960], Matthew R Lewis[962], Gonçalo dos Santos Correia[962], Caroline J Sands[962], Panteleimon Takis[962], Lynn Maslen[962], William Greenhalf[963], Victoria Shaw[964], Sarah E McDonald[936], Seán Keating[965], Katie A. Ahmed[966], Jane A Armstrong[966], Milton Ashworth[966], Innocent G Asiimwe[966], Siddharth Bakshi[966], Samantha L Barlow[966], Laura Booth[966], Benjamin Brennan[967], Katie Bullock[966], Benjamin WA Catterall[966], Jordan J Clark[966], Emily A Clarke[966], Sarah Cole[966], Louise Cooper[966], Helen Cox[966], Christopher Davis[966], Oslem Dincarslan[966], Chris Dunn[966], Philip Dyer[966], Angela Elliott[966], Anthony Evans[966], Lorna Finch[966], Lewis WS Fisher[966], Terry Foster[966], Isabel Garcia-Dorival[966], William Greenhalf[966], Philip Gunning[966], Catherine Hartley[966], Rebecca L Jensen[966], Christopher B Jones[966], Trevor R Jones[966], Shadia Khandaker[966], Katharine King[966], Robyn T. Kiy[966], Chrysa Koukorava[966], Annette Lake[967], Suzannah Lant[966], Diane Latawiec[966], Lara Lavelle-Langham[966], Daniella Lefteri[967], Lauren Lett[966], Lucia A Livoti[966], Maria Mancini[966], Sarah McDonald[966], Laurence McEvoy[966], John McLauchlan[967], Soeren Metelmann[966], Nahida S Miah[966], Joanna Middleton[966], Joyce Mitchell[966], Shona C Moore[966], Ellen G Murphy[966], Rebekah Penrice-Randal[966], Jack Pilgrim[966], Tessa Prince[966], Will Reynolds[966],

P. Matthew Ridley[966], Debby Sales[966], Victoria E Shaw[966], Rebecca K Shears[966], Benjamin Small[966], Krishanthi S Subramaniam[966], Agnieska Szemiel[966], Aislynn Taggart[967], Jolanta Tanianis-Hughes[966], Jordan Thomas[966], Erwan Trochu[966], Libby van Tonder[966], Eve Wilcock[966], J. Eunice Zhang[966], Lisa Flaherty[966], Nicole Maziere[966], Emily Cass[966], Alejandra Doce Carracedo[966], Nicola Carlucci[966], Anthony Holmes[966], Hannah Massey[966], Lee Murphy[554], Nicola Wrobel[554], Sarah McCafferty[554], Kirstie Morrice[554], Alan MacLean[554],

Admin Team Member

Ruth Armstrong[702], J. Kenneth Baillie[60,61,62], Ceilia Boz[702], Adam Brown[702], Richard Clark[968], Sara Clohisey[702], Audrey Coutts[968], Louise Cullum[702], Nicky Day[702], Lorna Donnelly[968], Esther Duncan[702], Angie Fawkes[968], Paul Finernan[702], Max Head Fourman[702], James Furniss[702], Tammy Gilchrist[968], Ailsa Golightly[702], Katarzyna Hafezi[968], Ross Hendry[702], Andy Law[702], Dawn Law[702], Rachel Law[702], Sarah Law[702], Louise Macgillivray[968], Alan Maclean[968], Hanning Mal[702], Sarah McCafferty[968], Ellie Mcmaster[702], Jen Meikle[702], Shona C Moore[969], Kirstie Morrice[968], Lee Murphy[968], Wilna Oosthuyzen[702], Nicholas Parkinson[702], Trevor Paterson[702], Andrew Stenhouse[702], Maaike Swets[60,970], Helen Szoor-McElhinney[702], Filip Taneski[702], Lance Turtle[969], Tony Wackett[702], Mairi Ward[702], Jane Weaver[702], Nicola Wrobel[968], Marie Zechner[702], Judy Coyle[702], Bernadette Gallagher[702], Rebecca Lidstone-Scott[702], Debbie Hamilton[702], Katherine Schon[971], Anita Furlong[971], Heather Biggs[971], Fiona Griffiths[60], Eleanor Andrews[762], Kathy Brickell[527], Michelle Smyth[527], Lorna Murphy[527], Ruth Armstrong[702], J. Kenneth Baillie[972], Ceilia Boz[702], Adam Brown[702], Richard Clark[968], Sara Clohisey[702], Audrey Coutts[968], Louise Cullum[702], Nicky Day[702], Lorna Donnelly[968], Esther Duncan[702], Angie Fawkes[968], Paul Finernan[702], Max Head Fourman[702], James Furniss[702], Tammy Gilchrist[968], Ailsa Golightly[702], Katarzyna Hafezi[968], Ross Hendry[702], Andy Law[702], Dawn Law[702], Rachel Law[702], Sarah Law[702], Louise Macgillivray[968], Alan Maclean[968], Hanning Mal[702], Sarah McCafferty[968], Ellie Mcmaster[702], Jen Meikle[702], Shona C Moore[969], Kirstie Morrice[968], Lee Murphy[968], Wilna Oosthuyzen[702], Nicholas Parkinson[702], Trevor Paterson[702], Andrew Stenhouse[702], Maaike Swets[973], Helen Szoor-McElhinney[702], Filip Taneski[702], Lance Turtle[969], Tony Wackett[702], Mairi Ward[702], Jane Weaver[702], Nicola Wrobel[968], Marie Zechner[702], Judy Coyle[702], Bernadette Gallagher[702], Rebecca Lidstone-Scott[702], Debbie Hamilton[702], Fiona Griffiths[702], Gail Carson[946], Hayley Hardwick[530], Chloe Donohue[715], Fiona Griffiths[60], Wilna Oosthuyzen[60],

# Corresponding authors

Corresponding authors

Benjamin M Neale[4] **+**, Mark Daly[1,2,3] **+**, Andrea Ganna[1,2,3] **+**

Affiliations

1. Institute for Molecular Medicine Finland (FIMM), University of Helsinki, Helsinki, Finland
2. Broad Institute of MIT and Harvard, Cambridge, MA, USA
3. Analytic and Translational Genetics Unit, Massachusetts General Hospital, Boston, MA, USA
4. Massachusetts General Hospital, Broad Institute of MIT and Harvard, Cambridge, MA, USA
5. Institute for Molecular Medicine Finland (FIMM), Helsinki, Finland
6. Yale University, New Haven, CT, USA

7. Icahn School of Medicine at Mount Sinai, New York, NY, USA
8. Stroke Pharmacogenomics and Genetics, Biomedical Research Institute Sant Pau (IIB Sant Pau), Sant Pau Hospital, Inmungen-CoV2, Barcelona, Spain
9. Institute of Virology, Technical University Munich/Helmholtz Zentrum München, Munich, Germany
10. Institute of Psychiatric Phenomics and Genomics, Medical Center of the University of Munich, Munich, Germany
11. Department of Psychiatry, Medical Center of the University of Munich, Munich, Germany
12. IRCCS Istituto Giannina Gaslini, Genova, Italy, Department of Neurosciences, Rehabilitation, Ophthalmology, Genetics, Maternal and Child Health, University of Genova, Genova, Italy
13. Univerisity of Helsinki, Helsinki, Finland
14. Queen Mary University of London, London, United Kingdom
15. Wellcome Sanger Institute, Wellcome Genome Campus, Hinxton, Cambridgeshire CB10 1SA, UK, Open Targets, Wellcome Genome Campus, Hinxton, Cambridgeshire CB10 1SD, UK
16. Department of Complex Trait Genetics, Center for Neurogenomics and Cognitive Research, Amsterdam Neuroscience, Vrije Universiteit Amsterdam, Amsterdam, The Netherlands
17. McGill University. Lady Davis Institute, Jewish General Hospital, Montréal, Canada
18. Medical Research Institute, Kangbuk Samsung Hospital, Sungkyunkwan University School of Medicine, Suwon, Republic of Korea
19. Osaka University Graduate School of Medicine, Osaka, Japan
20. Baylor College of Medicine, Houston, TX, USA
21. Mohammed Bin Rashid University of Medicine and Health Sciences, Dubai, United Arab Emirates
22. MRC Integrative Epidemiology Unit (IEU) at the University of Bristol, Bristol, UK
23. Department of Internal Medicine, Division of Cardiovascular Medicine, Michigan Medicine, Ann Arbor, MI, USA
24. Department of Human Genetics, University of Michigan Medical School, Ann Arbor, MI, USA
25. Department of Computational Medicine and Bioinformatics, University of Michigan Medical School, Ann Arbor, MI, USA
26. Seaver Autism Center for Research and Treatment
27. Department of Psychiatry
28. Icahn School of Medicine at Mount Sinai, New York, NY 10029, USA
29. Program in Medical and Population Genetics, Broad Institute of MIT and Harvard, Cambridge, MA, USA
30. Institute for Molecular Medicine Finland (FIMM), University of Helsinki, Helsinki, Finland
31. Institute for Molecular Medicine Finland, University of Helsinki, Helsinki, Finland
32. David Geffen School of Medicine at UCLA, Los Angeles, CA, USA
33. Institut Pasteur, Paris, France
34. Harvard School of Public Health, Boston, MA, USA
35. Institute for Molecular Bioscience, The University of Queensland, 306 Carmody Road, QLD 4072
36. Brisbane
37. Australia
38. Wellcome Sanger Institute, Wellcome Genome Campus, Hinxton, Cambridgeshire CB10 1SA, UK
39. Open Targets, Wellcome Genome Campus, Hinxton, Cambridgeshire CB10 1SD, UK
40. European Molecular Biology Laboratory, European Bioinformatics Institute (EMBL-EBI), Wellcome Genome Campus, Hinxton, Cambridgeshire CB10 1SD, UK
41. Seaver Autism Center for Research and Treatment, Department of Psychiatry Icahn School of Medicine at Mount Sinai, New York, NY 10029, USA
42. Centre for Bioinformatics and Data Analysis, Medical University of Bialystok, Bialystok, Poland
43. Trieste University, Trieste, Italy
44. Vanderbilt University Medical Center, Nashville, TN, USA
45. University of California San Francisco, San Fransisco, CA, USA

46. Stanford University, Stanford, USA
47. University of Siena, Genetica Medica, Azienda Ospedaliero-Universitaria Senese, Italy
48. Med Biotech Hub and Competence Center, Siena, Italy
49. Boston Children's Hospital, Broad Institute of MIT and Harvard, Cambridge, MA, USA
50. Blizard Institute, Queen Mary University of London, London, United Kingdom
51. Department of Genetics, University Medical Centre Groningen, Groningen, The Netherlands
52. University of Groningen Department of Genetics, University Medical Centre Utrecht, P.O. Box 85500, 3508 GA, Utrecht, The Netherlands
53. Department of Human Genetics, Epidemiology, Biostatistics and Occupational Health, McGill University, Montréal, Québec, Canada
54. Lady Davis Institute, Jewish General Hospital, McGill University, Montréal, Québec, Canada
55. Department of Twin Research, King's College London, London, United Kingdom.
56. Department of Human Genetics, McGill University, Montréal, Québec, Canada
57. Kyoto-McGill International Collaborative School in Genomic Medicine, Graduate School of Medicine, Kyoto University, Kyoto, Japan
58. Research Fellow, Japan Society for the Promotion of Science
59. National Institutes of Health, Bethesda, MD, USA
60. Roslin Institute, University of Edinburgh, Easter Bush, Edinburgh, EH25 9RG, UK
61. Intensive Care Unit, Royal Infirmary of Edinburgh, 54 Little France Drive, Edinburgh, EH16 5SA, UK
62. MRC Human Genetics Unit, Institute of Genetics and Molecular Medicine, University of Edinburgh, Western General Hospital, Crewe Road, Edinburgh, EH4 2XU, UK
63. Medical Genetics, University of Siena, Siena, Italy
64. Genetica Medica, Azienda Ospedaliero-Universitaria Senese, Italy
65. Med Biotech Hub and Competence Center, Department of Medical Biotechnologies, University of Siena, Italy
66. Department of Electronics, Information and Bioengineering (DEIB), Politecnico di Milano, Milano, Italy
67. Politecnico di Milano, Milan, Italy
68. University of Michigan, Ann Arbor, MI, USA
69. Vanderbilt School of Medicine, Nashville, TN, USA
70. All India Institute of Medical Sciences Kalyani, Kalyani, India
71. Hasso Plattner Institute, New York, NY, USA
72. Naina Tech, Hyderabad, India
73. EMBL-European Bioformatics Institute, Hinxton, UK
74. University of Northampton, Northampton, UK
75. University of Helsinki, Helsinki, Finland
76. University of Miami, Miami, FL, USA
77. Stanford UniversityStanford University, Stanford, USA
78. Nantes University, Ecole Centrale de Nantes, Inserm, Centre de Recherche en Transplantation et Immunologie, UMR1064, ITUN, Nantes, France
79. University of Liège, Liège, Belgium
80. Qatar Genome Program, Qatar Foundation Research, Development and Innovation, Qatar Foundation, Doha, Qatar
81. Scientific education head, Qatar Genome Program, Doha, Qatar
82. Medical and Population Genetics and Cardiovascular Disease Initiative, Broad Institute of Harvard and MIT, Cambridge, MA, USA
83. Cardiovascular Research Center, Massachusetts General Hospital, Boston, MA, USA
84. Intensive Care Unit Vall d'Hebron Hospital, Barcelona, Spain
85. Institut de Biomedicina de València – CSIC, València, Spain

86. Centro de Investigación Biomédica en Red en Enfermedades Neurodegenerativas (CIBERNED), València, Spain
87. Unidad Mixta de Neurología y Genética, Instituto de Investigación Sanitaria La Fe, València, Spain
88. Erasmus Medical Center, The Netherlands
89. National Genome Center: Copenhagen, Denmark
90. University of Copenhagen, Copenhagen, Denmark
91. Genomics plc, Oxford, UK
92. Medical Center of the University of Munich, Munich, Germany
93. Institute for Community Medicine, University Medicine Greifswald, Greifswald, Germany
94. Department of Population Medicine and Lifestyle Diseases Prevention, Medical University of Bialystok, Bialystok, Poland
95. Genomics England, London, United Kingdom
96. Junta de Andalucía, Seville, Spain
97. Human Genetics Program of ICBM and Department of Basic-Clinical Oncology, University of Chile, Santiago, Chile
98. Center for the Development of Scientific Research (CEDIC), Paris, France
99. Erasmus MC, Rotterdam, Netherlands
100. Translational Bioinformatics Unit, Navarrabiomed, Complejo Hospitalario de Navarra (CHN), Universidad Pública de Navarra (UPNA), IdiSNA, Pamplona, Spain
101. Mucosal & Salivary Biology Division, King's College London Dental Institute, London, UK
102. GENYO, Center for Genomics and Oncological Research Pfizer/University of Granada/Andalusian Regional Government, 18016 Granada, Spain
103. University of Puerto Rico, San Juan, Peurto Rico
104. National Laboratory of Genomics for Biodiversity (LANGEBIO), Advanced Genomics Unit, CINVESTAV, Irapuato, Guanajuato 36824, Mexico
105. Queensland University of Technology, Brisbane, Australia
106. Clinical Research Unit of nanoro, Institut de Recherche en Sciences de la Santé, CNRST, Ouagadougou, Burkina Faso
107. McGill University, Montréal, Canada
108. Université de Montréal, Montréal, Canada
109. Fonds de la Recherche Scientifique (FNRS) & Centre de Génétique Humaine, Hôpital Erasme, Université Libre de Bruxelles, Brussels, Belgium
110. All India Institute of Medical Sciences Kalyani, West Bengal
111. All India Institute of Medical Sciences, Kalyani, West Bengal
112. University of Pecs Medical School, Pécs, Hungary
113. Institute of Biomedicine, and Cancer Research Laboratories, Western Cancer Centre FICAN West, University of Turku, Turku, Finland
114. Institute of Biomedical Technologies, National Research Council, Segrate (Milan) 20090, Italy
115. Immediate, Milan, Italy
116. University of Cambridge, Cambridge, United Kingdom
117. Genome Opinion, Seoul, Republic of Korea
118. -
119. University of Groningen, Groningen, Netherlands
120. Universiti Malaysia Pahang, Gambang, Malaysia
121. Broad Institute of MIT and Harvard
122. Vrije Universiteit Amsterdam, Amsterdam, Netherlands
123. University Medical Centre Groningen, University of Groningen, Groningen, Netherlands
124. MNM DIAGNOSTICS, Poznań, Poland
125. Institute for Systems Biology, Seattle, WA, USA

126. Sultan Idris Education University, Tanjung Malim, Malaysia
127. Hospital Kulim, Kedah, Malaysia
128. AbbVie Inc, Lake Buff, IL, USA
129. Root Deep Insight, Boston MA, 02134 USA
130. 23andMe, Sunnyvale, CA, USA
131. GSK, Stevenage, United Kingdom
132. Northwestern Univerisity, Feinberg School of Medicine, Department of Pharmacology, Chicago, IL 60611
133. Northwestern Univeristy, Chief of Hospital Medicine, Department of Medicine, Chicago IL 60611
134. Washington, DC Veterans Affairs Medical Center, Hospital Medicine, Washington DC, 20422
135. George Washington University, Department of Medicine, Washington DC 20037
136. Univeristy of Chicago, Section of Hospital Medicine, Department of Medicine, Chicago IL 60637
137. Univeristy of Chicago, Section of Hematology and Oncology, Department of Medicine, Chicago IL 60639
138. University of Illinois at Chicago, College of Pharmacy, Chicago, IL 60612
139. Northwestern Univerisity, Feinberg School of Medicine, Department of Pharmacology, Chicago, IL 60612
140. Northwestern Univerisity, Feinberg School of Medicine, Department of Pharmacology, Chicago, IL 60614
141. Northwestern Univeristy, Department of Medicine, Chicago IL 60611
142. Washington, DC Veterans Affairs Medical Center, Hospital Medicine, Washington DC, 20423
143. Washington, DC Veterans Affairs Medical Center, Hospital Medicine, Washington DC, 20424
144. George Washington University, Department of Pharmacology, Washington DC 20038
145. Univeristy of Chicago, Section of Hospital Medicine, Department of Medicine, Chicago IL 60638
146. University of Illinois at Chicago, College of Pharmacy, Chicago, IL 60613
147. Northwestern Univerisity, Feinberg School of Medicine, Department of Pharmacology, Chicago, IL 60613
148. Department of Neurology, Amsterdam UMC, Amsterdam Neuroscience, Amsterdam, Netherlands
149. Department of Intensive Care, Amsterdam UMC, Amsterdam, Netherlands
150. Department of Infectious Diseases, Amsterdam UMC, Amsterdam, Netherlands
151. Department of Clinical Epidemiology, Biostatistics and Bioinformatics, Amsterdam UMC, Amsterdam, Netherlands
152. Experimental Immunology, Amsterdam UMC, Amsterdam, Netherlands
153. Department of Pulmonology, Amsterdam UMC, Amsterdam, Netherlands
154. Department of Pathology, Amsterdam UMC, Amsterdam, Netherlands
155. Department of Anesthesiology, Amsterdam UMC, Amsterdam, Netherlands.
156. Amsterdam UMC Biobank Core Facility, Amsterdam UMC, Amsterdam, Netherlands
157. Department of Radiology, Amsterdam UMC, Amsterdam, Netherlands
158. Department of Medical Microbiology, Amsterdam UMC, Amsterdam, Netherlands
159. Department of Clinical Chemistry, Amsterdam UMC, Amsterdam, Netherlands
160. Amsterdam UMC Biobank, Amsterdam UMC, Amsterdam, Netherlands
161. Core facility Genomics, Amsterdam UMC, Amsterdam, Netherlands
162. Ancestry, Lehi, UT, USA
163. University of Liege, GIGA-Institute, Liège, Belgium
164. CHC Mont-Légia, Liège, Liège, Belgium
165. CHU of Liege, BHUL (Liege biobank), Liège, Belgium
166. Analytic & Translational Genetics Unit, Massachusetts General Hospital, Boston, MA, USA
167. Stanley Center for Psychiatric Research & Program in Medical and Population Genetics, Broad Institute of MIT and Harvard, Cambridge, MA, USA

168. Centre de Génétique Humaine, Hôpital Erasme, Université Libre de Bruxelles, Brussels, Belgium
169. Service de Médecine Interne, Hôpital Erasme, Université Libre de Bruxelles, Brussels, Belgium
170. CHU of Liege, University of Liege, Liège, Belgium
171. CHU of Liege, Liège, Belgium
172. CHU of Liege, Liège, Belgium, Liège, Belgium
173. CHU of liege, Liège, Belgium
174. McGill Genome Centre and Department of Human Genetics, McGill University, Montréal, Québec, Canada
175. Department of Twin Research, King's College London, London, United Kingdom
176. Department of Epidemiology, Biostatistics and Occupational Health, McGill University, Montréal, Québec, Canada
177. Department of Emergency Medicine, McGill University, Montréal, Québec, Canada
178. Emergency Department, Jewish General Hospital, McGill University, Montréal, Québec, Canada
179. McGill AIDS Centre, Department of Microbiology and Immunology, Lady Davis Institute for Medical Research, Jewish General Hospital, McGill University, Montréal, Québec, Canada
180. McGill Centre for Viral Diseases, Lady Davis Institute, Department of Infectious Disease, Jewish General Hospital, Montréal, Québec, Canada
181. Research Centre of the Centre Hospitalier de l'Université de Montréal, Montréal, Canada
182. Department of Medicine and Research Centre of the Centre Hospitalier de l'Université de Montréal, Montréal, Canada
183. Department of Medicine, Université de Montréal, Montréal, Canada
184. Department of Medicine and Human Genetics, McGill University, Montréal, Québec, Canada
185. Department of Intensive Care and Research Centre of the Centre Hospitalier de l'Université de Montréal, Montréal, Canada
186. Division of Infectious Diseases and Research Centre of the Centre Hospitalier de l'Université de Montréal, Montréal, Canada
187. Division of Genetic Medicine, Department of Medicine, Vanderbilt University Medical Center
188. Vanderbilt Genetics Institute, Vanderbilt University Medical Center, Nashville, TN, USA
189. Institute of Human Genetics, University Hospital Bonn, Medical Faculty University of Bonn, 53127 Bonn, Germany
190. Institute of Genomic Statistics and Bioinformatics, University Hospital Bonn, Medical Faculty University of Bonn, Venusberg-Campus 1, 53127 Bonn, Germany
191. Department of Gastroenterology, Hepatology and Infectious Diseases, University Hospital Duesseldorf, Medical Faculty Heinrich Heine University, 40225 Duesseldorf, Germany
192. Institute of Human Genetics, Medical Faculty, RWTH Aachen University, Aachen, Germany
193. Clinic for Cardiology, Angiology and Internal Intensive Medicine, Medical Clinic I, RWTH Aachen University, Aachen, Germany
194. Department of Pneumology and Intensive Care Medicine, Faculty of Medicine, RWTH Aachen University, Aachen, Germany
195. Department of Pneumology, Hannover Medical School, Hannover, Germany
196. Department of Gastroenterology, Hepatology and Endocrinology, Hannover Medical School, Hannover, Germany
197. Hannover Unified Biobank, Hannover Medical School, Hannover, Germany
198. Department I of Internal Medicine, Faculty of Medicine and University Hospital of Cologne, University of Cologne, 50937 Cologne, Germany
199. Center for Molecular Medicine Cologne (CMMC), University of Cologne, 50931 Cologne, Germany
200. German Center for Infection Research (DZIF), Partner Site Bonn-Cologne, Cologne, Germany
201. Department I of Internal Medicine, Faculty of Medicine and University Hospital of Cologne, University of Cologne, 50937, Cologne, Germany

202. Cologne Center for Genomics (CCG), University of Cologne, Cologne, Germany
203. Department of Anesthesiology and Intensive Care Medicine, University Hospital Essen, University Duisburg-Essen, Essen, Germany
204. Department of Child and Adolescent Psychiatry, University Hospital Essen, University of Duisburg-Essen, Essen, Germany
205. Department of Infectious Diseases, University Hospital Essen, University Duisburg-Essen, Essen, Germany
206. Department of Pneumology, Allergology and Respiratory Medicine, University Hospital Saarland, Homburg/Saar, Germany
207. Center of Human and Molecular Biology, Department of Human Genetics, University Hospital Saarland, Homburg/Saar, Germany
208. Department of Genetics & Epigenetics, Saarland University, Saarbrücken, Germany
209. Eurac Research, Institute for Biomedicine (affiliated to the University of Lübeck), Bolzano, Italy
210. University of Colorado - Anschutz Medical Campus, Aurora, CO, USA
211. Department of Genetics and Development, Columbia University, Institute for Genomic Medicine,Columbia University, New York, NY, USA
212. Department of Medicine, Columbia University, Institute for Genomic Medicine, Columbia University, New York, NY, USA
213. Department of Biomedical Informatics, Columbia University, New York, NY, USA
214. Department of Pediatrics, Columbia University, New York, NY, USA
215. Department of Medicine, Columbia University, New York, NY, USA
216. Institute for Genomic Medicine, Columbia University, New York, NY, USA
217. Department of Biostatistics, Mailman School of Public Health, Columbia University, New York, NY, USA
218. Department of Pathology and Cell Biology, Columbia University, New York, NY, USA
219. Medical Research Institute, Kangbuk Samsung Hospital, Sungkyunkwan University School of Medicine, Seoul, Republic of Korea
220. Department of Clinical Research Design and Evaluation, SAIHST, Sungkyunkwan University, Seoul, Republic of Korea
221. Division of Gastroenterology, Department of Medicine, Kangbuk Samsung Hospital, Sungkyunkwan University, School of Medicine, Seoul, Republic of Korea
222. Department of Biochemistry, College of Medicine, Ewha Womans University, Seoul, Republic of Korea
223. Department of Internal Medicine, Seoul National University Hospital, Seoul National University College of Medicine, Seoul, Republic of Korea
224. Department of Periodontology, Section of Dentistry, Seoul National University Bundang Hospital, Seongnam, Republic of Korea
225. Department of Internal Medicine, Seoul National University Bundang Hospital, Seoul National University College of Medicine, Seongnam, Republic of Korea
226. Department of Physical & Rehabilitation Medicine, Kangbuk Samsung Hospital, Sungkyunkwan University School of Medicine, Seoul, Republic of Korea
227. Department of Clinical Research Design & Evaluation, SAIHST, Sungkyunkwan University, Seoul, Republic of Korea
228. Biomedical Institute for Convergence at SKKU, Sungkyunkwan University School of Medicine, Suwon, Republic of Korea
229. Department of Public Health Service, Seoul National University Bundang Hospital, Seongnam, Republic of Korea
230. Department of Rehabilitation Medicine, Seoul National University College of Medicine, Seoul, Republic of Korea

231. Korea Research Environment Open NETwork, Korea Institute of Science and Technology Information, Daejeon, Republic of Korea
232. Global Science Experimental Data Hub Center, Korea Institute of Science and Technology Information, Daejeon, Republic of Korea
233. Division of Infectious Diseases, Department of Medicine, Kangbuk Samsung Hospital, Sungkyunkwan University School of Medicine, Seoul, Republic of Korea
234. Center for Cohort studies, Kangbuk Samsung Hospital, Sungkyunkwan University School of Medicine, Seoul, Republic of Korea
235. Department of Occupational and Environmental Medicine, Sungkyunkwan University School of Medicine, Seoul, Republic of Korea
236. Department of Laboratory Medicine, Seoul National University Bundang Hospital, Seoul National University College of Medicine, Seongnam, Republic of Korea
237. Institute of Clinical Molecular Biology, Christian-Albrechts-University, Kiel, Germany
238. Novo Nordisk Foundation Center for Protein Research, Disease Systems Biology, Faculty of Health and Medical Sciences, University of Copenhagen, Copenhagen, Denmark
239. Institut de Biotecnologia i de Biomedicina, Universitat Autònoma de Barcelona, Bellaterra (Barcelona), Spain
240. ICREA, Barcelona, Spain
241. Research Group for Evolutionary Immunogenomics, Max Planck Institute for Evolutionary Biology, Plön, Germany
242. Research Unit for Evolutionary Immunogenomics, Department of Biology, University of Hamburg, Hamburg, Germany
243. Department of Gastroenterology, Hospital Universitario Ramón y Cajal, University of Alcalá, Instituto Ramón y Cajal de Investigación Sanitaria (IRYCIS), Madrid, Spain
244. Centro de Investigación Biomédica en Red en Enfermedades Hepáticas y Digestivas (CIBEREHD), Instituto de Salud Carlos III (ISCIII), Madrid, Spain
245. Vall d'Hebron Institut de Recerca (VHIR), Vall d'Hebron Hospital Universitari, Barcelona, Spain
246. Charite Universitätsmedizin Berlin, Berlin, Germany
247. Institute of Psychiatric Phenomics and Genomics, University Medical Center, University of Munich, Munich, Germany
248. Department of Psychiatry, University Medical Center, University of Munich, Munich, Germany
249. Hospital Universitario Clinico San Cecilio, Granada, Spain
250. Instituto de Investigación Ibs.Granada, Granada, Spain
251. Emergency Department, University Hospital Regensburg, Regensburg, Germany
252. Department for Infectious Diseases and Infection Control, University Hospital Regensburg, Regensburg, Germany
253. Medical University of Innsbruck, Department of Medicine and Christian Doppler Laboratory on Iron and Phosphate Biology, Innsbruck, Austria
254. Institute of Clinical Medicine, University of Oslo, Oslo, Norway
255. Department of Microbiology, Oslo University Hospital, Oslo, Norway
256. Hospital Clinic, University of Barcelona, and IDIBAPS, Barcelona, Spain
257. European Foundation for the Study of Chronic Liver Failure (EF-CLIF), Barcelona, Spain
258. University of Cologne, Medical Faculty and University Hospital Cologne, Department I of Internal Medicine, Cologne, Germany
259. University of Cologne, Cologne Excellence Cluster on Cellular Stress Responses in Aging-Associated Diseases (CECAD), Cologne, Germany
260. Center for Molecular Medicine Cologne (CMMC), University of Cologne, Cologne, Germany
261. Genomes for Life-GCAT lab. Germans Trias i Pujol Research Institute (IGTP), Badalona, Spain
262. IRCCS Humanitas Research Hospital, Rozzano, Milan, Italy

263. Department of Biomedical Sciences, Humanitas University, Pieve Emanuele, Milan, Italy
264. Institute of Transfusionsmedicine, University Hospital Schleswig-Holstein (UKSH), Germany
265. Klinik für Innere Medizin I, Universitätsklinikum Schleswig-Holstein, Campus Kiel, Germany
266. Zentrum für Humangenetik Regensburg, Regensburg, Germany
267. University Hospital Schleswig-Holstein (UKSH), Campus Kiel, Germany
268. Section for Gastroenterology, Department of Transplantation Medicine, Division for Cancer medicine, Surgery and Transplantation, Oslo University Hospital Rikshospitalet, Oslo, Norway
269. Research Institute for Internal Medicine, Division of Surgery, Inflammatory Diseases and Transplantation, Oslo University Hospital Rikshospitalet and University of Oslo, Oslo, Norway
270. Norwegian PSC Research Center, Department of Transplantation Medicine, Division of Surgery, Inflammatory Diseases and Transplantation, Oslo University Hospital Rikshospitalet, Oslo, Norway
271. Section for Gastroenterology, Department of Transplantation Medicine, Division for Cancer Medicine, Surgery and Transplantation, Oslo University Hospital Rikshospitalet, Oslo, Norway
272. Division of Rheumatology, Inflammation and Immunity, Brigham and Women's Hospital and Harvard Medical School, Boston, MA, USA
273. Division of Genetics, Department of Medicine, Brigham and Women's Hospital, Boston, MA, USA
274. Department of Biomedical Informatics, Harvard Medical School, Boston, MA, USA
275. Center for Data Sciences, Brigham and Women's Hospital, Boston, MA, USA
276. Randaberg Municipality, Norway
277. University of Stavanger, Faculty of health sciencies, Departement of quality and health technology, Stavanger, Norway
278. Norwegian Institute of Public Health, Division of Health Data and Digitalization, Department of Genetics and Bioinformatics (HDGB) Oslo, Norway
279. Centre for Genetics and Genomics Versus Arthritis, Centre for Musculoskeletal Research, Manchester Academic Health Science Centre, The University of Manchester, Manchester, UK
280. Department of Intensive Care, Hospital Universitario Ramón y Cajal, Instituto Ramón y Cajal de Investigación Sanitaria (IRYCIS), University of Alcalá, Madrid, Spain
281. Osakidetza Basque Health Service, Donostialdea Integrated Health Organisation, Clinical Biochemistry Department, San Sebastian, Spain
282. Research Center and Memory Clinic, Fundació ACE, Institut Català de Neurociències Aplicades, Universitat Internacional de Catalunya, Barcelona, Spain
283. Networking Research Center on Neurodegenerative Diseases (CIBERNED), Instituto de Salud Carlos III, Madrid, Spain
284. Department of Acute Medicine, Oslo University Hospital, Oslo, Norway
285. Fondazione IRCCS Ca' Granda Ospedale Maggiore Policlinico, Milan, Italy
286. European Reference Network on Hepatological Diseases (ERN RARE LIVER), San Gerardo Hospital, Monza, Italy
287. Division of Gastroenterology, Center for Autoimmune Liver Diseases, department of Medicne and Surgery, University of Milan Bicocca, Milan, Italy
288. German Center for Neurodegenerative Diseases (DZNE Bonn), Bonn, Germany
289. Division of Neurogenetics and Molecular Psychiatry, Department of Psychiatry and Psychotherapy, University of Cologne, Medical Faculty, Cologne, Germany
290. Department of Psychiatry and Glenn Biggs Institute for Alzheimer's and Neurodegenerative Diseases, San Antonio, TX, USA
291. Department of Neurodegenerative Diseases and Geriatric Psychiatry, University Hospital Bonn, Bonn, Germany
292. Liver Unit, Department of Internal Medicine, Hospital Universitari Vall d'Hebron, Vall d'Hebron Barcelona Hospital Campus, Barcelona, Spain

293. Department of Anesthesiology and Intensive Care, University Hospital of North Norway, Tromsø, Norway
294. Klinik für Innere Medizin I, Universitätsklinikum Schleswig-Holstein, Campus Kiel
295. Gastroenterology Unit, Fondazione IRCCS Casa Sollievo della Sofferenza, San Giovanni Rotondo, Italy
296. Dept. of Infectious diseases, Oslo University Hospital, Norway
297. Microbiology Department, Hospital Universitari Vall d'Hebron, Barcelona, Spain
298. Universitat Autònoma de Barcelona, Bellatera, Spain
299. Department of Respiratory Diseases, Hospital Universitario Ramón y Cajal, Instituto Ramón y Cajal de Investigación Sanitaria (IRYCIS), University of Alcalá, Centro de Investigación Biomédica en Red en Enfermedades Respiratorias (CIBERES), Madrid, Spain
300. Department of Respiratory Medicine and Allergology, University Hospital, Goethe University, Frankfurt am Main, Germany
301. Department of Infectious Diseases, Hospital Universitario Ramón y Cajal, Instituto Ramón y Cajal de Investigación Sanitaria (IRYCIS), University of Alcalá, Madrid, Spain
302. Technical University of Munich, School of Medicine, University Hospital rechts der Isar, Department of Internal Medicine II, Munich, Germany
303. Division of Clinical Infectious Diseases, Research Center Borstel, Borstel, Germany
304. German Center for Infection Research (DZIF) Clinical Tuberculosis Unit, Borstel, Germany
305. Respiratory Medicine & International Health, University of Lübeck, Lübeck, Germany
306. Osakidetza Basque Health Service, Basurto University Hospital, Respiratory Service, Bilbao, Spain
307. Department of Clinical and Molecular Medicine, Faculty of Medicine and Health Science, Norwegian University of Science and Technology, Trondheim, Norway
308. Clinic Ålesund Hospital, Dept. of Medicine, Møre & Romsdal Hospital Trust, Ålesund, Norway
309. Department of Anesthesiology, Hospital Universitario Ramón y Cajal, Instituto Ramón y Cajal de Investigación Sanitaria (IRYCIS), University of Alcalá, Madrid, Spain
310. Liver ICU, Hospital Clinic Barcelona, Barcelona, Spain Hospital Clinic, University of Barcelona, and IDIBAPS, Barcelona, Spain
311. Osakidetza Basque Health Service, Galdakao Hospital, Respiratory Service, Galdakao, Spain
312. IBMDR - E.O. Ospedali Galliera, Genova, Italy
313. Liver ICU, Hospital Clinic Barcelona, Barcelona, Spain
314. Biocruces Bizkaia Health Research Institute, Barakaldo, Spain
315. Histocompatibilidad y Biologia Molecular, Centro de Transfusion de Madrid, Madrid, Spain
316. University of Milan, Milan, Italy
317. Fondazione Grigioni per il Morbo di Parkinson, Milan, Italy
318. Department of Anesthesiology, Intensive Care Medicine and Pain Therapy, University Hospital Frankfurt, Frankfurt am Main, Germany
319. University of Cologne, Medical Faculty and University Hospital Cologne, German Center for Infection Research (DZIF), Partner Site Bonn-Cologne, Cologne, Germany
320. Department of Liver and Gastrointestinal Diseases, Biodonostia Health Research Institute - Donostia University Hospital, University of the Basque Country (UPV/EHU), CIBERehd, Ikerbasque, San Sebastian, Spain
321. Ikerbasque, Basque Foundation for Science, Bilbao, Spain
322. Department of Liver and Gastrointestinal Diseases, Biodonostia Health Research Institute – Donostia University Hospital – University of the Basque Country (UPV/EHU), San Sebastian, Spain
323. Infectious Diseases Service, Osakidetza, Biocruces Bizkaia Health Research Institute, Barakaldo, Spain
324. Medical Department, Drammen Hospital, Vestre Viken Hospital Trust, Norway
325. Research Center Borstel, BioMaterialBank Nord, Germany

326. German Center for Lung Research (DZL), Airway Research Center North (ARCN), Germany
327. Popgen 2.0 network (P2N), Kiel, Germany
328. Department of Liver and Gastrointestinal Diseases, Biodonostia Health Research Institute – Donostia University Hospital –, University of the Basque Country (UPV/EHU), San Sebastian, Spain
329. Department of Liver and Gastrointestinal Diseases, Biodonostia Health Research Institute - Donostia University Hospital, University of the Basque Country (UPV/EHU), CIBERehd, San Sebastian, Spain
330. Dept. of Infectious diseases, Oslo University Hospital, Oslo, Norway
331. Department of Clinical Science, University of Bergen, Bergen, Norway
332. Biodonostia Health Research Institute, Donostia University Hospital, San Sebastian, Spain
333. Germans Trias i Pujol Research Institute (IGTP), Badalona, Spain
334. Analytic & Translational Genetics Unit, Massachusetts General Hospital, Boston, MA, USA Stanley Center for Psychiatric Research & Program in Medical and Population Genetics, Broad Institute of MIT and Harvard, Cambridge, MA, USA
335. ISGlobal, Barcelona, Spain
336. CIBER Epidemiología y Salud Pública (CIBERESP), Madrid, Spain
337. Universitat Pompeu Fabra (UPF), Barcelona, Spain
338. IMIM (Hospital del Mar Medical Research Institute), Barcelona, Spain
339. Osakidetza Basque Health Service, Donostialdea Integrated Health Organization, San Sebastian, Spain
340. Department of Internal Medicine, Infectious Diseases, University Hospital Frankfurt & Goethe University Frankfurt, Frankfurt am Main, Germany
341. Humanitas Gavazzeni-Castelli, Bergamo, Italy
342. Department of Liver and Gastrointestinal Diseases, Biodonostia Health Research Institute, San Sebastian, Spain
343. Department of Liver and Gastrointestinal Diseases, Biodonostia Health Research Institute - Donostia University Hospital, Ikerbasque, San Sebastian, Spain
344. School of Biological Sciences, Monash University, Clayton, VIC, Australia
345. Munich Clinic Schwabing, Academic Teaching Hospital, Ludwig-Maximilians-University (LMU), Munich, Germany
346. Department of Anesthesiology, Hospital Universitario Ramón y Cajal, Instituto Ramón y Cajal de Investigación Sanitaria (IRYCIS), Madrid, Spain
347. NA
348. University of Cologne, Cologne Excellence Cluster on Cellular Stress Responses in Aging-Associated Diseases (CECAD), Cologne, Germany
349. Clinical Trials Centre Cologne, ZKS Köln, Cologne, Germany
350. University of Cologne, Medical Faculty and University Hospital Cologne, German Center for Infection Research (DZIF), Partner Site Bonn-Cologne, Cologne, Germany
351. Institute of Human Genetics, University of Bonn School of Medicine & University Hospital Bonn, Bonn, Germany
352. Institute of Clinical Molecular Biology, Christian-Albrechts-University of Kiel, Kiel, Germany
353. Institute of Immunology, Christian-Albrechts-University of Kiel & UKSH Schleswig-Holstein, Kiel, Germany
354. Institute of Immunology, Christian-Albrechts-University of Kiel, Kiel, Germany
355. Institute of Medical Virology, University Hospital Frankfurt, Goethe University, Frankfurt am Main, Germany
356. German Centre for Infection Research (DZIF), External Partner Site Frankfurt, Frankfurt am Main, Germany
357. Department of Neurology, Bezirksklinikum Regensburg, University of Regensburg, Regensburg, Germany

358. Institute of Human Genetics, University of Bonn, School of Medicine & University Hospital Bonn, Bonn, Germany
359. Charite Universitätsmedizin Berlin, Berlin Institute of Health, Berlin Germany
360. German Center for Infection Research (DZIF), Munich partner site, Munich, Germany
361. Department of Infectious Diseases, University Hospital of North Norway, Tromsø, Norway
362. Faculty of Health Sciences, UIT The Arctic University of Norway, Norway
363. Catalan Institute of Oncology (ICO), Barcelona, Spain
364. Bellvitge Biomedical Research Institute (IDIBELL), Barcelona, Spain
365. Universitat de Barcelona (UB), Barcelona, Spain
366. deCODE genetics, Reykjavik, Iceland
367. GENYO. Centre for Genomics and Oncological Research: Pfizer / University of Granada / Andalusian Regional Government, Parque Tecnológico de la Salud, Granada, Spain
368. Mucosal Immunology Lab. Unidad de Excelencia Instituto de Biomedicina y Genética Molecular de Valladolid (IBGM, Universidad de Valladolid-CSIC). Valladolid. Spain
369. Centro de Investigaciones Biomédicas en Red de Enfermedades Hepáticas y Digestivas (CIBERehd). Madrid, Spain
370. Valladolid University Hospital, Valladolid, Spain
371. Estonian Genome Centre, Institute of Genomics, University of Tartu, Tartu, Estonia
372. SYNLAB Estonia, University of Tartu, Tartu, Estonia
373. Kuressaare Hospital and University of Tartu, Tartu, Estonia
374. Kuressaare Hospital, Kuressaare, Estonia
375. Institute of Biomedicine and Translational Medicine, University of Tartu
376. Institute of Biomedicine and Translational Medicine, University of Tartu, Tartu, Estonia
377. West Tallinn Central Hospital, Tallinn, Estonia
378. University of Tartu, Tartu University Hospital, Tartu, Estonia
379. Estonian Health Insurance Fund, Tallinn, Estonia
380. Tartu University Hospital, Tartu, Estonia
381. FinnGen, Helsinki, Finland
382. Finnish Institute for Health and Welfare (THL), Helsinki, Finland
383. University of Helsinki, Faculty of Medicine, Clinical and Molecular Metabolism Research Program, Helsinki, Finland
384. Institute of Molecular and Clinical Ophthalmology Basel (IOB), Basel, Switzerland
385. Department of Ophthalmology, University of Basel, Basel, Switzerland
386. Infectious Diseases Service, Department of Medicine, University Hospital and University of Lausanne, Lausanne, Switzerland
387. Infectious Diseases Service, Department of Medicine, University Hospital, University of Lausanne, Lausanne, Switzerland
388. Centre for Primary Care and Public Health, University of Lausanne, Lausanne, Switzerland.
389. Division of Infectious Diseases and Hospital Epidemiology, Cantonal Hospital St Gallen, St Gallen, Switzerland.
390. Division of Intensive Care, Geneva University Hospitals and the University of Geneva Faculty of Medicine, Geneva, Switzerland.
391. Infectious Disease Service, Department of Internal Medicine, Geneva University Hospital, Geneva, Switzerland.
392. Clinique de Médecine et spécialités, Infectiologie, HFR-Fribourg, Fribourg, Switzerland
393. Infectious Diseases Division, University Hospital Centre of the canton of Vaud, hospital of Valais, Sion, Switzerland
394. Functional Host Genomics of Infectious Diseases, University Hospital and University of Lausanne, Lausanne, Switzerland

395. Registry COVID, University Hospital and University of Lausanne, Lausanne, Switzerland
396. Pneumonia prediction using lung ultrasound, University Hospital and University of Lausanne, Lausanne, Switzerland
397. Center for Primary Care and Public Health (Unisanté), University of Lausanne, Lausanne, Switzerland
398. Covid-19 Risk Prediction in Swiss ICUs-Trial, Division of Infectious Diseases and Hospital Epidemiology, Cantonal Hospital St Gallen, St Gallen, Switzerland.
399. GCAT-Genomes for Life, Germans Trias i Pujol Health Sciences Research Institute (IGTP), Crta. de Can Ruti, Cami de les Escoles s/n.08916 Badalona, Catalonia
400. Catalan Institute of Oncology,Bellvitge Biomedical Research Institute, Consortium for Biomedical Research in Epidemiology and Public Health and University of Barcelona, Barcelona, Spain
401. Barcelona Supercomputing Center - Centro Nacional de Supercomputación (BSC-CNS).Life & Medical Sciences *currently at Programs in Metabolism and Medical and Population Genetics, Broad Institute of MIT and Harvard, Cambridge, MA, USA and Diabetes Unit and Center for Genomic Medicine, Massachusetts General Hospital, Boston, MA, USA. Harvard Medical School, Boston, Massachusetts, USA
402. Barcelona Supercomputing Center - Centro Nacional de Supercomputación (BSC-CNS).Life & Medical Sciences, Barcelona, Spain
403. ICREA Research Professor at Barcelona Supercomputing Center - Centro Nacional de Supercomputación (BSC-CNS).Life & Medical Sciences, Barcelona, Spain
404. University of Siena, DIISM-SAILAB, Siena, Italy
405. Université Côte d’Azur, Inria, CNRS, I3S, Maasai
406. Medical Genetics, University of Siena, Italy
407. Med Biotech Hub and Competence Center, Department of Medical Biotechnologies, University of Siena, Siena, Italy
408. Division of Infectious Diseases and Immunology, Department of Medical Sciences and Infectious Diseases, Fondazione IRCCS Policlinico San Matteo, Pavia, Italy
409. Department of Internal Medicine and Therapeutics, University of Pavia, Italy
410. Department of Infectious and Tropical Diseases, University of Brescia and ASST Spedali Civili Hospital, Brescia, Italy
411. Chirurgia Vascolare, Ospedale Maggiore di Crema, Crema, Italy
412. III Infectious Diseases Unit, ASST-FBF-Sacco, Milan, Italy
413. Department of Biomedical and Clinical Sciences Luigi Sacco, University of Milan, Milan, Italy
414. Dept of Specialized and Internal Medicine, Tropical and Infectious Diseases Unit, Azienda Ospedaliera Universitaria Senese, Siena, Italy
415. Unit of Respiratory Diseases and Lung Transplantation, Department of Internal and Specialist Medicine, University of Siena, Siena, Italy
416. Dept of Emergency and Urgency, Medicine, Surgery and Neurosciences, Unit of Intensive Care Medicine, Siena University Hospital, Siena, Italy
417. Department of Medical, Surgical and Neuro Sciences and Radiological Sciences, Unit of Diagnostic Imaging, University of Siena, Siena, Italy
418. Rheumatology Unit, Department of Medicine, Surgery and Neurosciences, University of Siena, Policlinico Le Scotte, Siena, Italy
419. Department of Specialized and Internal Medicine, Infectious Diseases Unit, San Donato Hospital Arezzo, Arezzo, Italy
420. Dept of Emergency, Anesthesia Unit, San Donato Hospital, Arezzo, Italy
421. Department of Specialized and Internal Medicine, Pneumology Unit and UTIP, San Donato Hospital, Arezzo, Italy
422. Department of Emergency, Anesthesia Unit, Misericordia Hospital, Grosseto, Italy
423. Department of Specialized and Internal Medicine, Infectious Diseases Unit, Misericordia Hospital, Grosseto, Italy

424. Department of Preventive Medicine, Azienda USL Toscana Sud Est, Arezzo, Italy
425. Clinical Chemical Analysis Laboratory, Misericordia Hospital, Grosseto, Italy
426. Territorial Scientific Technician Department, Azienda USL Toscana Sud Est, Arezzo, Italy
427. Clinical Chemical Analysis Laboratory, San Donato Hospital, Arezzo, Italy
428. Department of Health Sciences, Clinic of Infectious Diseases, ASST Santi Paolo e Carlo, University of Milan, Milan, Italy
429. Department of Anesthesia and Intensive Care, University of Modena and Reggio Emilia, Modena, Italy
430. HIV/AIDS Department, National Institute for Infectious Diseases, IRCCS, Lazzaro Spallanzani, Rome, Italy
431. Infectious Diseases Clinic, Department of Medicine 2, Azienda Ospedaliera di Perugia and University of Perugia, Santa Maria Hospital, Perugia, Italy
432. Infectious Diseases Clinic, "Santa Maria" Hospital, University of Perugia, Perugia, Italy
433. Department of Infectious Diseases, Treviso Hospital, Local Health Unit 2 Marca Trevigiana, Treviso, Italy
434. Clinical Infectious Diseases, Mestre Hospital, Venezia, Italy
435. Infectious Diseases Clinic, ULSS1, Belluno, Italy
436. Medical Genetics and Laboratory of Medical Genetics Unit, A.O.R.N. "Antonio Cardarelli", Naples, Italy
437. Department of Molecular Medicine and Medical Biotechnology, University of Naples Federico II, Naples, Italy
438. CEINGE Biotecnologie Avanzate, Naples, Italy
439. IRCCS SDN, Naples, Italy
440. Unit of Respiratory Physiopathology, AORN dei Colli, Monaldi Hospital, Naples, Italy
441. Division of Medical Genetics, Fondazione IRCCS Casa Sollievo della Sofferenza Hospital, San Giovanni Rotondo, Italy
442. Department of Medical Sciences, Fondazione IRCCS Casa Sollievo della Sofferenza Hospital, San Giovanni Rotondo, Italy
443. Infectious Diseases Clinic, Policlinico San Martino Hospital, IRCCS for Cancer Research, Genova, Italy
444. Microbiology, Fondazione Policlinico Universitario Agostino Gemelli IRCCS, Catholic University of Medicine, Rome, Italy
445. Department of Laboratory Sciences and Infectious Diseases, Fondazione Policlinico Universitario A. Gemelli IRCCS, Rome, Italy
446. Department of Cardiovascular Diseases, University of Siena, Siena, Italy
447. Otolaryngology Unit, University of Siena, Italy
448. Department of Internal Medicine, ASST Valtellina e Alto Lario, Sondrio, Italy
449. First Aid Department, Luigi Curto Hospital, Polla, Salerno, Italy
450. U.O.C. Laboratorio di Genetica Umana, IRCCS Istituto G. Gaslini, Genova, Italy
451. Infectious Diseases Clinics, University of Modena and Reggio Emilia, Modena, Italy
452. Department of Respiratory Diseases, Azienda Ospedaliera di Cremona, Cremona, Italy
453. U.O.C. Medicina, ASST Nord Milano, Ospedale Bassini, Cinisello Balsamo (MI), Italy
454. Istituto Auxologico Italiano, IRCCS, Department of Cardiovascular, Neural and Metabolic Sciences, San Luca Hospital, Milan, Italy
455. Department of Medicine and Surgery, University of Milano-Bicocca, Milan, Italy
456. Istituto Auxologico Italiano, IRCCS, Center for Cardiac Arrhythmias of Genetic Origin, Milan, Italy
457. Istituto Auxologico Italiano, IRCCS, Laboratory of Cardiovascular Genetics, Milan, Italy
458. Unit of Infectious Diseases, ASST Papa Giovanni XXIII Hospital, Bergamo, Italy
459. Istituti Clinici Scientifici Maugeri IRCCS, Department of Cardiology, Institute of Montescano, Pavia, Italy

460. Istituti Clinici Scientifici Maugeri, IRCCS, Department of Cardiac Rehabilitation, Institute of Tradate (VA), Italy
461. Cardiac Rehabilitation Unit, Fondazione Salvatore Maugeri, IRCCS, Scientific Institute of Milan, Milan, Italy
462. IRCCS C. Mondino Foundation, Pavia, Italy
463. Medical Genetics Unit, Meyer Children's University Hospital, Florence, Italy
464. Department of Medicine, Pneumology Unit, Misericordia Hospital, Grosseto, Italy
465. Department of Preventive Medicine, Azienda USL Toscana Sud Est, Italy
466. Department of Anesthesia and Intensive Care Unit, ASST Fatebenefratelli Sacco, Luigi Sacco Hospital, Polo Universitario, University of Milan, Milan, Italy
467. Health Management, Azienda USL Toscana Sudest, Tuscany, Italy
468. Department of Mathematics, University of Pavia, Pavia, Italy
469. Independent Data Scientist, Milan, Italy
470. Scuola Normale Superiore, Pisa, Italy
471. CNR-Consiglio Nazionale delle Ricerche, Istituto di Biologia e Biotecnologia Agraria (IBBA), Milano, Italy
472. CNR-Consiglio Nazionale delle Ricerche, Istituto di Biologia e Biotecnologia Agraria (IBBA), Milano, Italy
473. Veos Digital, Milan, Italy
474. Med Biotech Hub and Competence Center, Department of Medical Biotechnologies, University of Siena,Siena, Italy Italy
475. Core Research Laboratory, ISPRO, Florence, Italy
476. Analytic & Translational Genetics Unit, Massachusetts General Hospital, Boston, MA, USA. Stanley Center for Psychiatric Research & Program in Medical and Population Genetics, Broad Institute of MIT and Harvard, Cambridge, MA, USA
477. Genetica Medica, Azienda Ospedaliero-Universitaria Senese, Siena, Italy
478. Division of Infectious Diseases and Immunology, Fondazione IRCCS Policlinico San Matteo, Pavia, Italy
479. Department of Molecular and Translational Medicine, University of Brescia, Italy
480. Clinical Chemistry Laboratory, Cytogenetics and Molecular Genetics Section, Diagnostic Department, ASST Spedali Civili di Brescia, Italy
481. Department of Medical and Surgical Sciences for Children and Adults, University of Modena and Reggio Emilia, Modena, Italy
482. Department of Molecular Medicine, University of Padova, Italy
483. Laboratory of Regulatory and Functional Genomics, Fondazione IRCCS Casa Sollievo della Sofferenza, San Giovanni Rotondo (Foggia), Italy
484. Clinical Trial Office, Fondazione IRCCS Casa Sollievo della Sofferenza Hospital, San Giovanni Rotondo, Italy
485. Department of Health Sciences, University of Genova, Genova, Italy
486. Study Coordinator Oncologia Medica e Ufficio Flussi Sondrio, Italy
487. Local Health Unit-Pharmaceutical Department of Grosseto, Toscana Sud Est Local Health Unit, Grosseto, Italy
488. Independent Scientist, Milan, Italy
489. Direzione Scientifica, Istituti Clinici Scientifici Maugeri IRCCS, Pavia, Italy
490. Fondazione per la ricerca Ospedale di Bergamo, Bergamo, Italy
491. Allelica Inc, New York, NY, USA
492. Dept of Specialized and Internal Medicine, Tropical and Infectious Diseases Unit, Azienda Ospedaliera Universitaria Senese, Siena, Italy

493. School of Basic and Medical Biosciences, Faculty of Life Sciences and Medicine, King's College London, London, United Kingdom
494. Medical and Population Genomics, Wellcome Sanger Institute, Hinxton, UK
495. Bradford Institute for Health Research, Bradford Teaching Hospitals National Health Service (NHS) Foundation Trust, Bradford, UK
496. Blizard Institute, 4 Newark Street, Queen Mary University of London
497. Institute of Population Health Sciences, 4 Newark Street, Queen Mary University of London, London, United Kingdom
498. Genes & Health, Blizard Institute, Queen Mary University of London, E1 2AT, London, United Kingdom
499. Institute of Population Health Sciences, 4 Newark Street, Queen Mary University of London
500. Department of Biostatistics, University of Michigan, Ann Arbor, MI, USA
501. Heart Institute (InCor)/Univ Sao Paulo Med Sch, São Paulo, Brazil
502. Genentech, San Fransisco, CA, USA
503. DNA Link, Seoul, Republic of Korea
504. Seoul National University Hospital Gangnam Center, Seoul, Republic of Korea
505. Division of Infectious Diseases, Department of Internal Medicine, Chungnam National University School of Medicine, Daejeon, Republic of Korea
506. Division of Infectious Disease, Department of Internal Medicine, Chungnam National Univsersity School of Medicine, Daejeon, Republic of Korea
507. Department of Internal Medicine, School of Medicine, Kyungpook National University, Daegu, Republic of Korea
508. Incheon Medical Center, Incheon, Republic of Korea
509. Department of Infectious disease, Keimyung university Dongsan hospital, Keimyung University School of Medicine, Daegu, Republic of Korea
510. Department of Internal Medicine, Pusan National University School of Medicine and Medical Research Institute, Pusan National University Hospital, Busan, Republic of Korea.
511. Myongji Hospital, Goyang, Republic of Korea
512. Gradate School of Public Health, Yonsei University, Seoul, Republic of Korea
513. Stanford University, Division of Cardiovascular Medicine, Stanford, USA
514. Stanford University, Departments of Medicine and Genetics, Stanford, USA
515. Stanford University, Division of Cardiovascular Medicine , Stanford, USA
516. Stanford University, Department of Biomedical Data Science, Stanford, USA
517. Stanford University, Departments of Genetics and Biomedical Data Science, Stanford, USA
518. Stanford University, Department of Pathology, Stanford, USA
519. Illumina, Inc, San Diego, CA, USA
520. Computational Biology, Drug Discovery Sciences, Takeda Pharmaceuticals, Boston, MA, USA
521. Department of Computational Biology, and Swiss Institute of Bioinformatics (SIB), University of Lausanne, Lausanne, Switzerland
522. Stanford University, Dept of Pathology, Stanford, USA
523. Royal Victoria Hospital, Belfast, NI
524. Chelsea & Westminster NHS Foundation Trust, London, UK
525. Northampton General Hospital NHS Trust, Northampton, UK
526. Wrexham Maelor Hospital, Wrexham, Wales
527. University College Dublin, St Vincent's University Hospital, Dublin, Ireland
528. University Hospitals Coventry & Warwickshire NHS Trust, Coventry, UK
529. Watford General Hospital, Watford, UK
530. NIHR Health Protection Research Unit, Institute of Infection, Veterinary and Ecological Sciences, Faculty of Health and Life Sciences, University of Liverpool, Liverpool, UK
531. Queen Alexandra Hospital (Hampshire), Portsmouth Hospital Trust, Hampshire, UK

532. Princess Royal Hospital, Brighton & Sussex Universities Hospitals NHS Trust, Brighton, UK
533. Bassettlaw Hospital, Doncaster and Bassetlaw, Nottinghamshire, UK
534. Darent Valley Hospital, Dartford & Gravesham NHS Trust, Kent, UK
535. High Containment Laboratories, University of Birmingham, Birmingham, UK
536. Queen Elizabeth the Queen Mother Hospital, Margate, East Kent Hospitals NHS Foundation Trust, Kent, UK
537. John Radcliffe Hospital, Oxford University Hospitals NHS Foundation Trust, Oxford, UK
538. Royal Albert Edward Infirmary (Wigan), Wrightington, Wigan and Leigh, Wigan, UK
539. Manchester Royal Infirmary, Manchester University Hospitals NHS Foundation Trust, Manchester, UK
540. Furness General Hospital, Morecambe Bay NHS Foundation Trust, Cumbria, UK
541. Castle Hill Hospital , Hull University Teaching Hospital Trust, Hull, UK
542. Hillingdon Hospital, Hillingdon Hospital, London, UK
543. St Thomas Hospital, Guys and St Thomas Foundation Trust, London, UK
544. University Hospitals Coventry and Warwickshire, Coventry, UK
545. St Michaels Hospital (Bristol), University Hospitals Bristol and Weston NHS Foundation Trust, Bristol, UK
546. Stepping Hill Hospital, Stockport NHS Foundation Trust, Manchester, UK
547. Royal Liverpool Hospital, Liverpool University Hospitals NHS Foundation Trust, Liverpool, UK
548. Bristol Royal Hospital (Children's), University Hospitals Bristol and Weston NHS Foundation Trust, Bristol, UK
549. Scarborough Hospital, York teaching Hospitals NHS Foundation Trust, York, UK
550. Liverpool Heart & Chest Hospital, Liverpool Heart & Chest NHS Foundation Trust, Liverpool, UK
551. James Paget University Hospital, James Paget University Hospitals NHS Foundation Trust, Norfolk, UK
552. The James Cook University Hospital, South Tees NHS Foundation Trust, Middlesborough, UK
553. Aberdeen Royal Infirmary, Grampian, Aberdeen, UK
554. University of Edinburgh, Edinburgh, UK
555. Royal Devon and Exeter Hospital, Royal Devon and Exeter NHS Foudation Trust, Exeter, UK
556. Worcestershire Royal Hospital, Worcestershire Acute Hospitals NHS Trust, Worcester, UK
557. Conquest Hospital, Hastings, East Sussex Healthcare NHS Trust, Sussex, UK
558. Dorset County Hospital, Dorset County Hospital NHS Foundation Trust, Dorset, UK
559. Royal Bournemouth General Hospital, University Hospitals Dorset NHS Foundation Trust, Bournemouth, UK
560. Harrogate Hospital, Harrogate and District NHS Foundation Trust, Harrogate, UK
561. Burnley General Teaching Hospital, East Lancashire Hospitals NHS Hospitals, Burnley, UK
562. Torbay Hospital, Torbay & South Devon NHS Foundation Trust, Torquay, UK
563. Royal Hallamshire Hospital, Sheffield Teaching Hospitals NHS Foundation Trust, Sheffield, UK
564. St Georges Hospital (Tooting), St Georges University Hospitals NHS Foundation Trust, London, UK
565. Blackpool Victoria Hospital, Blackpool Teaching Hospitals NHS Foundation Trust, Blackpool, UK
566. The Royal London Hospital, Barts Health NHS Trust, London, UK
567. Salford Royal NHS Foundation Trust, Salford Royal NHS Foundation Trust, Manchester, UK
568. University Hospital of North Durham, County Durham and Darlington Foundation Trust, Durham, UK
569. Norfolk & Norwich University Hospital , Norfolk and Norwich University Hospital NHS Foundation Trust, Norfolk, UK
570. Salisbury District Hospital, Salisbury NHS Foundation Trust, Salisbury, UK
571. St Thomas, Guys and St Thomas Foundation Trust, London, UK
572. Fairfield General Hospital, Pennine Acute Hospitals NHS Trust, Manchester, UK
573. Hereford County Hospital, Wye Valley NHS Trust, Hereford, UK

574. Southampton General Hospital, University Hospital Southampton NHS Foundation Trust, Southampton, UK
575. Northampton General Hospital, Northampton General Hospital NHS Trust, Northampton, UK
576. University Hospital of Wales, Cardiff and Vale University Health Board, Cardiff, UK
577. University of Bristol, Bristol, UK
578. Leighton Hospital, Mid Cheshire Hospitals NHS Foundation Trust, Cheshire, UK
579. Diana Princess of Wales Hospital (Grimsby), North Lincolnshire & Goole, Grimsby, UK
580. Manor Hospital, Walsall Healthcare NHS Trust, Walsall, UK
581. Addenbrookes Hospital, Cambridge University Hospital NHS Foundation Trust, Cambridge, UK
582. West Suffolk Hospital, West Suffolk Hospital NHS Foundation Trust, Suffolk, UK
583. Basingstoke and North Hampshire Hospital, Hampshire Hospitals NHS Foundation Trust, Hampshire, UK
584. North Cumbria Integrated Care NHS Foundation Trust, Cumbria, UK
585. Warwick Hospital, South Warwickshire NHS Foundation Trust, Warwick, UK
586. Birmingham Women and Children's Hospital , Birmingham Womens Childrens Hospital, Birmingham, UK
587. Nottingham City Hospital, Nottingham University Hospitals NHS Trust, Nottingham, UK
588. Glangwili Hospital Child Health Section, Hywel Dda University Health Board, Carmarthen, UK
589. Alder Hey Children's Hospital, Alder Hey Children's NHS Foundation Trust, Liverpool, UK
590. Bronglais General Hospital, Hywel Dda University Health Board, Aberystwyth, UK
591. Worthing Hospital, Western Sussex Hospitals NHS Foundation Trust, Sussex, UK
592. Rotheram District General Hospital, The Rotheram NHS Foundation Trust, Rotherham, UK
593. Royal Free Hospital, Royal Free London NHS Foundation Trust, London, UK
594. Homerton Hospital, Homerton University Hospital NHS Foundation Trust, London, UK
595. Airedale Hospital, Airedale NHS Foundation Trust, Keighley, UK
596. Basildon Hospital, Basildon and Thurrock University Hospitals NHS Foundation Trust, Essex, UK
597. The Christie NHS Foundation Trus, The Christie NHS Foundation Trust, Manchester, UK
598. Queen Elizabeth Hospital (Greenwich), Lewisham and Greenwich NHS Trust, London, UK
599. The Whittington Hospital, Whittington Health NHS Trust, London, UK
600. Sheffield Childrens Hospital , Sheffield Children's NHS Foundation Trust, Sheffield, UK
601. Royal United Hospital, Bath, Royal United Hospitals Bath NHS Foundation Trust, Bath, UK
602. Western General Hospital, Edinburgh, Lothian, UK
603. Mid and South Essex NHS Foundation Trust, Essex, UK
604. Hinchingbrooke Hospital, North West Anglia NHS Foundation Trust, Peterborough, UK
605. Royal Preston Hospital, Lancashire Teaching Hospitals NHS Foundation Trust, Preston, UK
606. University Hospital (Coventry) , University Hospitals Coventry and Warwickshire, Coventry, UK
607. The Walton Centre, The Walton Centre, Liverpool, UK
608. Hull Royal Infirmary, Hull University Teaching Hospital Trust, Hull, UK
609. Darlington Memorial Hospital, County Durham and Darlington Foundation Trust, Darlington, UK
610. Queen Elizabeth Hospital (Gateshead), Gateshead NHS Foundation Trust, Newcastle, UK
611. Warrington Hospital, Warrington & Halton Hospitals NHS Foundation Trust, Warrington, UK
612. University Hospitals Bristol and Weston NHS Foundation Trust, Bristol, UK
613. St Mary's Hospital (Isle of Wight), Isle of Wight NHS Trust, Isle of Wight, UK
614. The Maidstone Hospital, Maidstone & Tunbridge Wells NHS Trust, Maidstone, UK
615. Huddersfield Royal, Calderdale and Huddersfield NHS Foundation Trust, Huddersfield, UK
616. Royal Surrey County Hospital, Surrey, UK
617. Countess of Chester Hospital, Countess of Chester Hospital NHS Foundation Trust, Chester, UK
618. Frimley Park Hospital, Frimley Health Foundation Trust, Surrey, UK
619. Royal Hallamshire Hospital, Sheffield Teaching Hospitals NHS FoundationTrust, Sheffield, UK

620. Leeds General Infirmary, Leeds Teaching Hospitals, Leeds, UK
621. North Middlesex Hospital, North Middlesex University Hospital NHS Trust, London, UK
622. Arrowe Park Hospital, Wirral University Teaching Hospital NHS Foundation Trust, Wirral, UK
623. Great Ormond Street Hospital, Great Ormond Street Hospital for Children NHS Foundation Trust, London, UK
624. Royal Shrewsbury Hospital, Shrewsbury and Telford Hospital NHS Trust, Shropshire, UK
625. East Surrey Hospital (Redhill), Surrey & Sussex Healthcare, Surrey, UK
626. Burton Hospital, University Hospitals of Derby & Burton NHS Foundation Trust, Burtom, UK
627. Kent and Canterbury Hospital, East Kent Hospitals NHS Foundation Trust, Kent, UK
628. Weston Area General Trust, University Hospitals Bristol and Weston NHS Foundation Trust, Bristol, UK
629. Luton and Dunstable University Hospital, Bedford, UK
630. Glasgow Royal Infirmary, Greater Glasgow and Clyde, Glasgow, UK
631. Derbyshire Healthcare, Derbyshire Healthcare, Derby, UK
632. Macclesfield General Hospital, East Cheshire NHS Foundation Trust, Cheshire, UK
633. Chelsea and Westminster Hospital, Chelsea and Westminster NHS Trust, London, UK
634. Institute of Microbiology and Infection, University of Birmingham, Birmingham, UK
635. Prince Philip Hospital, Hwyel Dda University Health Board, Llanelli, Wales
636. George Eliot Hospital - Acute Services, George Eliot Hospital, Nuneaton, UK
637. Kettering General Hospital, Kettering General Hospital NHS Foundation Trust, Kettering, UK
638. Heartlands Hospital, Birmingham, Birmingham, UK
639. Russells Hall Hospital, The Dudley Group NHS Foundation Trust, Dudley, UK
640. Harefield Hospital , Royal Brompton & Harefield Trust, London, UK
641. Lister Hospital, East and North Hertfordshire NHS Trust, Stevenage, UK
642. Musgrove Park Hospital (Taunton & Somerset), Somerset NHS Foundation Trust, Somerset, UK
643. Queen's Hospital, Havering (Romford), Barking, Havering and Redbridge University Hospitals NHS Trust, London, UK
644. Southport & Formby District General Hospital, Southport and Ormskirk Hospital NHS Trust, Southport, UK
645. New Cross Hospital, The Royal Wolverhampton NHS Trust, Wolverhampton, UK
646. Kings College Hospital (Denmark Hill), London, UK
647. The Royal Victoria Infirmary, Newcastle Hospitals NHS Trust, Newcastle, UK
648. The Great Western Hospital, Great Western Hospitals NHS Foundation Trust, Swindon, UK
649. Ninewells Hospital, Tayside, Dundee, UK
650. Poole Hospital NHS Trust, Dorset, UK
651. Burton Hospital, University Hospitals of Derby & Burton NHS Foundation Trust, Derby, UK
652. William Harvey Hospital, Ashford, East Kent Hospitals NHS Foundation Trust, Kent, UK
653. Kings Mill Hospital, Sherwood Forest Hospitals NHS Foundation Trust, Nottinghamshire, UK
654. Liverpool Women's NHS Foundation Trust, Liverpool, UK
655. Dewsbury Hospital, Mid Yorkshire Hospitals NHS Trust, Dewsbury, UK
656. Northern Devon District Hospital, Northern Devon Healthcare NHS Trust, Devon, UK
657. Tameside General Hospital, Tameside and Glossop Integrated Care NHS Foundation Trust, Manchester, UK
658. Sandwell General Hospital, Sandwell And West Birmingham Hospitals NHS Trust, Birmingham, UK
659. Broomfield Hospital, Mid and South Essex University Hospitals Group, Essex, UK
660. Wycombe Hospital, Buckingham Healthcare NHS Trust, Buckinghamshire, UK
661. University Hospital of North Tees, North Tees and Hartlepool NHS Trust, Stockton-on-Tees, UK
662. Royal Manchester Children's Hospital, Manchester University Hospitals NHS Foundation Trust, Manchester, UK

663. Bedford Hospital, Bedford, UK
664. Colchester General Hospital, East Suffolk and North Essex Foundation Trust, Essex, UK
665. Queen Elizabeth Hospital (Birmingham) and Heartlands, University Hospital Birmingham NHS Foundation Trust, Birmingham, UK
666. Chesterfield Royal Hospital, Chesterfield Royal Hospital NHS Foundation Trust, Chesterfield, UK
667. Princess Alexandra Hospital, The Princess Alexandra Hospital NHS Trust, Essex, UK
668. Watford General Hospital, West Hertfordshire Hospitals NHS Trust, Watford, UK
669. Milton Keynes Hospital, Milton Keynes University Hospital NHS Foundation Trust, Milton Keynes, UK
670. Royal Bolton General Hospital, Bolton Foundation Trust, Bolton, UK
671. Royal Gwent (Newport), Aneurin Bevan University Health Board, Newport, UK
672. The Royal Marsden Hospital (London), The Royal Marsden NHS Foundation Trust, London, UK
673. Queen Victoria Hospital (East Grinstead), Queen Victoria Hospital NHS Foundation Trust, Sussex, UK
674. County Hospital (Stafford), University Hospitals Of North Midlands NHS Trust, Stafford, UK
675. Whiston Hospital, St Helen's & Knowlsey Hospitals NHS Trust, Prescot, UK
676. Croydon University Hospital, Croydon University Hospital, London, UK
677. Gloucester Royal, Gloucestershire Hospitals NHS Foundation Trust, Gloucester, UK
678. Medway Maritime Hospital, Medway Maritime NHS Trust, Kent, UK
679. Royal Papworth Hospital Everard, Royal Papworth Hospital NHS Foundation Trust, Cambridge, UK
680. Derriford (Plymouth), University Hospital Plymouth NHS Trust, Plymouth, UK
681. St Helier Hospital, Epsom and St Helier University Hospital NHS Trust, London, UK
682. Royal Berkshire Hospital, Royal Berkshire Foundation Trust, London, UK
683. Bradford Royal Infirmary, Bradford Teaching Hospitals NHS Foundation Trust, Bradford, UK
684. Northwick Park, London North West University Hospital Trust, London, UK
685. Ealing Hospital, London North West University Hospital Trust, London, UK
686. Royal Cornwall Hospital (Tresliske), Royal Cornwall NHS Trust, Cornwall, UK
687. Ashford Hospital, Ashford & St. Peter's Hospital, Surrey, UK
688. Leicester Royal Infirmary (Includes Glenfield Site), University Hospitals of Leicester, Leicester, UK
689. Grantham and District Hospital, United Lincolnshire Hospitals NHS Trust, Grantham, UK
690. University Hospital Aintree, Liverpool University Hospitals NHS Foundation Trust, Liverpool, UK
691. North Tyneside General Hospital, Northumbria Healthcare NHS Trust, Northumbria, UK
692. Queen Elizabeth Hospital (King's Lynn), Queen Elizabeth Hospital, King's Lynn, NHS Foundation Trust, Norfolk, UK
693. The Crick Institute, London, UK
694. Genomics England, London, UK
695. William Harvey Research Institute, Barts and the London School of Medicine and Dentistry, Queen Mary University of London, London EC1M 6BQ, UK.
696. Centre for Genomic and Experimental Medicine, Institute of Genetics and Molecular Medicine, University of Edinburgh, Western General Hospital, Crewe Road, Edinburgh, EH4 2XU, UK
697. Intensive Care National Audit & Research Centre, London, UK
698. Intensive Care Unit, Royal Infirmary of Edinburgh, 54 Little France Drive, Edinburgh, EH16 5SA, UK.
699. MRC Human Genetics Unit, Institute of Genetics and Molecular Medicine, University of Edinburgh, Western General Hospital, Crewe Road, Edinburgh, EH4 2XU, UK.
700. Wellcome Centre for Human Genetics, University of Oxford, Oxford, UK.
701. Genomics England, London, UK.
702. Roslin Institute, University of Edinburgh, Easter Bush, Edinburgh, EH25 9RG, UK.
703. Centre for Genomic and Experimental Medicine, Institute of Genetics and Molecular Medicine, University of Edinburgh, Western General Hospital, Crewe Road, Edinburgh, EH4 2XU, UK.
704. Intensive Care National Audit & Research Centre, London, UK.

705. Centre for Inflammation Research, The Queen's Medical Research Institute, University of Edinburgh, 47 Little France Crescent, Edinburgh, UK
706. Great Ormond Street Hospital for Children NHS Foundation Trust, London, UK.
707. Biostatistics Group, School of Life Sciences, Sun Yat-sen University, Guangzhou, China
708. Centre for Global Health Research, Usher Institute of Population Health Sciences and Informatics, Teviot Place, Edinburgh EH8 9AG, UK
709. Department of Medical Epidemiology and Biostatistics, Karolinska Institutet, Stockholm, Sweden
710. Institute for Molecular Bioscience, The University of Queensland, Brisbane, Australia
711. School of Life Sciences, Westlake University, Hangzhou, Zhejiang 310024, China
712. Westlake Laboratory of Life Sciences and Biomedicine, Hangzhou, Zhejiang 310024, China
713. Biostatistics Group, School of Life Sciences, Sun Yat-sen University, Guangzhou, China.
714. Centre for Medical Informatics, The Usher Institute, University of Edinburgh, Edinburgh, UK
715. Liverpool Clinical Trials Centre, University of Liverpool, Liverpool, UK
716. Centre for Health Informatics, Division of Informatics, Imaging and Data Science, School of Health Sciences, Faculty of Biology, Medicine and Health, University of Manchester, Manchester Academic Health Science Centre, Manchester, UK
717. MRC Human Genetics Unit, MRC Institute of Genetics and Molecular Medicine, University of Edinburgh, Edinburgh, UK
718. School of Informatics, University of Edinburgh, Edinburgh, UK
719. Royal Hospital for Children, Glasgow, UK
720. William Harvey Research Institute, Barts and the London School of Medicine and Dentistry, Queen Mary University of London, London EC1M 6BQ, UK
721. MRC-University of Glasgow Centre for Virus Research, Institute of Infection, Immunity and Inflammation, College of Medical, Veterinary and Life Sciences, University of Glasgow, Glasgow, UK
722. Centre for Tropical Medicine and Global Health, Nuffield Department of Medicine, University of Oxford, Old Road Campus, Roosevelt Drive, Oxford, OX3 7FZ, UK
723. Wellcome Centre for Human Genetics, University of Oxford, Oxford, UK
724. Department of Anaesthesia and Intensive Care, The Chinese University of Hong Kong, Prince of Wales Hospital, Hong Kong, China
725. Department of Critical Care Medicine, Queen's University and Kingston Health Sciences Centre, Kingston, ON, Canada
726. Wellcome-Wolfson Institute for Experimental Medicine, Queen's University Belfast, Belfast, Northern Ireland, UK
727. Department of Intensive Care Medicine, Royal Victoria Hospital, Belfast, Northern Ireland, UK
728. UCL Centre for Human Health and Performance, London, W1T 7HA, UK
729. Clinical Research Centre at St Vincent's University Hospital, University College Dublin, Dublin, Ireland
730. National Heart and Lung Institute, Imperial College London, London, UK
731. Imperial College Healthcare NHS Trust:London,London,UK
732. NIHR Health Protection Research Unit for Emerging and Zoonotic Infections, Institute of Infection, Veterinary and Ecological Sciences University of Liverpool, Liverpool, L69 7BE, UK
733. Respiratory Medicine, Alder Hey Children's Hospital, Institute in The Park, University of Liverpool, Alder Hey Children's Hospital, Liverpool, UK
734. Department of Intensive Care Medicine, Guy's and St. Thomas NHS Foundation Trust, London, UK
735. Department of Medicine, University of Cambridge, Cambridge, UK
736. Airedale General Hospital, Keighley, UK
737. Barts Health NHS Trust, London, UK
738. Basildon Hospital, Basildon, UK
739. BHRUT (Barking Havering) - Queens Hospital and King George Hospital, Essex, UK
740. Bradford Royal Infirmary, Bradford, UK

741. Bronglais General Hospital, Aberystwyth, UK
742. Broomfield Hospital, Chelmsford, UK
743. Calderdale Royal Hospital, Halifax, UK
744. Charing Cross Hospital, St Mary's Hospital and Hammersmith Hospital, London, UK
745. Barnet Hospital, London, UK
746. Birmingham Children's Hospital, Birmingham, UK
747. St John's Hospital Livingston, Livingston, UK
748. Aberdeen Royal Infirmary, Aberdeen, UK
749. Addenbrooke's Hospital, Cambridge, UK
750. Aintree University Hospital, Liverpool, UK
751. aintree University Hospital, Liverpool, UK
752. Arrowe Park Hospital, Wirral, UK
753. Ashford and St Peter's Hospital, Surrey, UK
754. Basingstoke and North Hampshire Hospital, Basingstoke, UK
755. Borders General Hospital, Melrose, UK
756. Chesterfield Royal Hospital Foundation Trust, Chesterfield, UK
757. Eastbourne District General Hospital, East Sussex, UK and Conquest Hospital, East Sussex, UK
758. Barnsley Hospital, Barnsley, UK
759. Blackpool Victoria Hospital, Blackpool, UK
760. East Surrey Hospital, Redhill, UK
761. Good Hope Hospital, Birmingham, UK
762. Hereford County Hospital, Hereford, UK
763. Hull Royal Infirmary, Hull, UK
764. Hull Royal Infirmary, Hull, Uk
765. Kent & Canterbury Hospital, Canterbury, UK
766. Manchester Royal Infirmary, Manchester, UK
767. Nottingham University Hospital, Nottingham, UK
768. Pilgrim Hospital, Lincoln, UK
769. Queen Elizabeth Hospital, Birmingham, UK
770. Salford Royal Hospital, Manchester, UK
771. Tameside General Hospital, Ashton Under Lyne, UK
772. The Tunbridge Wells Hospital and Maidstone Hospital, Kent, UK
773. The Royal Oldham Hospital, Manchester, UK
774. The Royal Papworth Hospital, Cambridge, UK
775. University College Hospital, London, UK
776. Withybush General Hospital, Pembrokeshire, Wales
777. Wythenshawe Hospital, Manchester, UK
778. Yeovil Hospital, Yeovil, UK
779. Cumberland Infirmary, Carlisle, UK
780. Darent Valley Hospital, Dartford, UK
781. Dumfries and Galloway Royal Infirmary, Dumfries, UK
782. Ealing Hospital, Southall, UK
783. Fairfield General Hospital, Bury, UK
784. George Eliot Hospital NHS Trust, Nuneaton, UK
785. Glan Clwyd Hospital, Bodelwyddan, UK
786. Glangwili General Hospital, Camarthen, UK
787. The Great Western Hospital, Swindon, UK
788. Guys and St Thomas' Hospital, London, UK
789. Harefield Hospital, London, UK

790. Harrogate and District NHS Foundation Trust, Harrogate, UK
791. Heartlands Hospital, Birmingham, UK
792. James Paget University Hospital NHS Trust, Great Yarmouth, UK
793. King's College Hospital, London, UK
794. King's Mill Hospital, Nottingham, UK
795. Kingston Hospital, Surrey, UK
796. Lincoln County Hospital, Lincoln, UK
797. Liverpool Heart and Chest Hospital, Liverpool, UK
798. Macclesfield District General Hospital, Macclesfield, UK
799. Medway Maritime Hospital, Gillingham, UK
800. Milton Keynes University Hospital, Milton Keynes, UK
801. Morriston Hospital, Swansea, UK
802. National Hospital for Neurology and Neurosurgery, London, UK
803. Norfolk and Norwich University hospital (NNUH), Norwich, UK
804. North Middlesex University Hospital NHS trust, London, UK
805. north Middlesex University Hospital NHS trust, London, UK
806. Northumbria Healthcare NHS Foundation Trust, North Shields, UK
807. Peterborough City Hospital, Peterborough, UK
808. Prince Charles Hospital, Merthyr Tydfil, UK
809. Royal Sussex County Hospital, Brighton, UK
810. Princess Royal Hospital, Haywards Heath, UK
811. Princess of Wales Hospital, Llantrisant, UK
812. Queen Alexandra Hospital, Portsmouth, UK
813. Queen Elizabeth Hospital, Woolwich, London, UK
814. Queen Elizabeth the Queen Mother Hospital, Margate, UK
815. Queen Victoria Hospital, West Sussex, UK
816. Queens Hospital Burton, Burton-On-Trent, UK
817. Raigmore Hospital, Inverness, UK
818. Rotherham General Hospital, Rotherham, UK
819. Royal Blackburn Teaching Hospital, Blackburn, UK
820. Royal Preston Hospital, Preston, UK
821. Royal Surrey County Hospital, Guildford, UK
822. Royal Albert Edward Infirmary, Wigan, UK
823. The Royal Alexandra Children's Hospital, Brighton, UK
824. Royal Alexandra Hospital, Paisley, UK
825. Royal Bolton Hospital, Bolton, UK
826. University Hospitals Dorset NHS Foundation Trust
827. Royal Brompton Hospital, London, UK
828. Imperial College London, London, UK
829. Royal Cornwall Hospital, Truro, UK
830. Royal Free Hospital, London, UK
831. Royal Glamorgan Hospital, Pontyclun, UK
832. Royal Gwent Hospital, Newport, UK
833. Royal Hallamshire Hospital and Northern General Hospital, Sheffield, UK
834. Royal Hampshire County Hospital, Hampshire, UK
835. Royal Manchester Children's Hospital, Manchester, UK
836. Royal Stoke University Hospital, Staffordshire, UK
837. Salisbury District Hospital, Salisbury, UK
838. Sandwell General Hospital, Birmingham, UK

839. Scarborough General Hospital, Scarborough, UK
840. Scunthorpe General Hospital, Scunthorpe, UK
841. Southmead Hospital, Bristol, UK
842. St George's Hospital, London, UK
843. St Mary's Hospital, Newport, UK
844. Stoke Mandeville Hospital, Buckinghamshire, UK
845. Sunderland Royal Hospital, Sunderland, UK
846. Alexandra Hospital, Redditch and Worcester Royal Hospital, Worcester, UK
847. The Christie NHS Foundation Trust, Manchester, UK
848. The Queen Elizabeth Hospital, King's Lynn, UK
849. The Royal Liverpool University Hospital, Liverpool, UK
850. The Royal Marsden NHS Foundation Trust, London, UK
851. Torbay Hospital, Torquay, UK
852. University Hospital Monklands, Airdrie, UK
853. University Hospital Lewisham, London, UK
854. University Hospital North Durham, Darlington, UK
855. University Hospital of North Tees, Stockton on Tees, UK
856. University Hospital of Wales, Cardiff, UK
857. University Hospital Wishaw, Wishaw, UK
858. Victoria Hospital, Kirkcaldy, UK
859. Warrington General Hospital, Warrington, UK
860. West Cumberland Hospital, Whitehaven, UK
861. Western Sussex Hospitals, West Sussex, UK
862. Whiston Hospital, Prescot, UK
863. York Hospital, York, UK
864. Ysbyty Gwynedd, Bangor, UK
865. Countess of Chester Hospital, Chester, UK
866. Croydon University Hospital, Croydon, UK
867. Diana Princess of Wales Hospital, Grimsby, UK
868. Dorset County Hospital, Dorchester, UK
869. Forth Valley Royal Hospital, Falkirk, UK
870. Furness General Hospital, Barrow-in-Furness, UK
871. Alder Hey Children's Hospital, Liverpool, UK
872. Derriford Hospital, Plymouth, UK
873. Glasgow Royal Infirmary, Glasgow, UK
874. Glenfield Hospital, Leicester, UK
875. Gloucestershire Royal Hospital, Gloucester, UK
876. Golden Jubilee National Hospital, Clydebank, UK
877. Great Ormond St Hospital and UCL Great Ormond St Institute of Child Health NIHR Biomedical Research Centre, London, UK
878. Homerton University Hospital Foundation NHS Trust, London UK
879. James Cook University Hospital, Middlesbrough, UK
880. John Radcliffe Hospital, Oxford, UK
881. Leicester Royal Infirmary, Leicester, UK
882. Lister Hospital, Stevenage, UK
883. New Cross Hospital, Wolverhampton, UK
884. Royal Victoria Infirmary, Newcastle Upon Tyne, UK
885. Ninewells Hospital, Dundee, UK
886. North Devon District Hospital, Barnstaple, UK

887. North Manchester General Hospital, Manchester, UK
888. Northwick Park Hospital, London, UK
889. Prince Philip Hospital, Lianelli, UK
890. Pinderfields General Hospital, Wakefield, UK
891. Poole Hospital, Poole, UK
892. Royal Shrewsbury Hospital, Shrewsbury, UK
893. Princess Royal Hospital , Telford , UK
894. Princess Royal Hospital, Telford, UK
895. Princess Royal Hospital Telford, UK
896. Princess Royal Hospital Shrewsbury and Royal Shrewsbury Hospital, Shrewsbury, UK
897. Princess Royal Hospital , Telford, UK
898. Princess Royal Hospital,Telford, UK
899. Queen Elizabeth Hospital Gateshead, Gateshead, UK
900. Queen Elizabeth University Hospital, Glasgow, UK
901. Royal Berkshire NHS Foundation Trust, Berkshire, UK
902. Royal Derby Hospital, Derby, UK
903. Royal Devon and Exeter Hospital, Exeter, UK
904. Royal Infirmary of Edinburgh, Edinburgh, UK
905. Royal Lancaster Infirmary, Lancaster, UK
906. Royal United Hospital, Bath, UK
907. Russell's Hall Hospital, Dudley, UK
908. Sheffield Children's Hospital, Sheffield, UK
909. Southampton General Hospital, Southampton, UK
910. Southend University Hospital, Westcliff-on-Sea, UK
911. Southport and Formby District General Hospital, Ormskirk, UK
912. St James's University Hospital and Leeds General Infirmary, Leeds, UK
913. Bristol Royal Infirmary, Bristol, UK
914. Stepping Hill Hospital, Stockport, UK
915. The Princess Alexandra Hospital, Harlow, UK
916. University Hospital Crosshouse, Kilmarnock, UK
917. University Hospital Hairmyres, East Kilbride, UK
918. Craigavon Area Hospital
919. Warwick Hospital, Warwick, UK
920. West Middlesex Hospital, Isleworth, UK
921. Western General Hospital, Edinburgh, UK
922. Whittington Hospital, London, UK
923. William Harvey Hospital, Ashford, UK
924. MRC-University of Glasgow Centre for Virus Research, Institute of Infection, Immunity and Inflammation, College of Medical, Veterinary and Life Sciences, University of Glasgow, Glasgow, UK.
925. Centre for Tropical Medicine and Global Health, Nuffield Department of Medicine, University of Oxford, Old Road Campus, Roosevelt Drive, Oxford, OX3 7FZ, UK.
926. Department of Anaesthesia and Intensive Care, The Chinese University of Hong Kong, Prince of Wales Hospital, Hong Kong, China.
927. Department of Critical Care Medicine, Queen's University and Kingston Health Sciences Centre, Kingston, ON, Canada.
928. UCL Centre for Human Health and Performance, London, W1T 7HA, UK.
929. Clinical Research Centre at St Vincent's University Hospital, University College Dublin, Dublin, Ireland.
930. Department of Intensive Care Medicine, Guy's and St. Thomas NHS Foundation Trust, London, UK.
931. Department of Medicine, University of Cambridge, Cambridge, UK.

932. Section of Molecular Virology, Imperial College London, London, UK
933. Antimicrobial Resistance and Hospital Acquired Infection Department, Public Health England, London, UK
934. Department of Infectious Disease, Imperial College London, London, UK
935. National Infection Service, Public Health England, London, UK
936. MRC-University of Glasgow Centre for Virus Research, 464 Bearsden Road, Glasgow, UK
937. Liverpool School of Tropical Medicine, Liverpool, UK
938. Institute of Infection and Global Health, University of Liverpool, Liverpool, UK
939. Centre for Tropical Medicine and Global Health, Nuffield Department of Medicine, University of Oxford, Old Road Campus, Roosevelt Drive, Oxford, UK
940. Virology Reference Department, National Infection Service, Public Health England, Colindale Avenue, London, UK
941. Department of Pharmacology, University of Liverpool, Liverpool, UK
942. Nuffield Department of Medicine, Peter Medawar Building for Pathogen Research, University of Oxford, UK
943. The Roslin Institute, University of Edinburgh, Edinburgh, UK
944. Nottingham University Hospitals NHS Trust, Nottingham, UK
945. Nuffield Department of Medicine, John Radcliffe Hospital, Oxford, UK
946. ISARIC Global Support Centre, Centre for Tropical Medicine and Global Health, Nuffield Department of Medicine, University of Oxford, Oxford, UK
947. Division of Infection and Immunity, University College London, UK
948. Institute of Infection, Veterinary and Ecological Sciences, University of Liverpool, Liverpool, UK
949. Centre for Clinical Infection and Diagnostics Research, Department of Infectious Diseases, School of Immunology and Microbial Sciences, King's College London, London, UK
950. Institute of Evolutionary Biology, University of Edinburgh, Edinburgh, UK
951. Department of Pediatrics and Virology, St Mary's Medical School Bldg, Imperial College London, London, UK
952. The Florey Institute for Host-Pathogen Interactions, Department of Infection, Immunity and Cardiovascular Disease, University of Sheffield, Sheffield, UK
953. Division of Structural Biology, The Wellcome Centre for Human Genetics, University of Oxford, Headington, Oxford, OX3 7BN, UK
954. Department of Medicine, University of Cambridge, Cambridge, Cambridgeshire, UK
955. Blood Borne Virus Unit, Virus Reference Department, National Infection Service, Public Health England, London, UK
956. Department of Infection, Immunity and Cardiovascular Disease, University of Sheffield, Sheffield, UK
957. Institute for Global Health, University College London, London, UK
958. Molecular and Clinical Cancer Medicine, Institute of Systems, Molecular and Integrative Biology, University of Liverpool, Liverpool, UK
959. Department of Child Life and Health, University of Edinburgh, Edinburgh, UK
960. Section of Biomolecular Medicine, Division of Systems Medicine, Department of Metabolism, Digestion and Reproduction, Sir Alexander Fleming Building, Exhibition Rd, London SW7 2AZ, UK
961. Department of Epidemiology and Biostatistics, School of Public Health, Faculty of Medicine, Imperial College London, 2 Norfolk St, W2 1PG, London, UK
962. National Phenome Centre, Department of Metabolism, Digestion and Reproduction, Imperial College London, London W12 0NN, United Kingdom
963. Department of Molecular and Clinical Cancer Medicine, University of Liverpool, Liverpool, UK
964. Institute of Translational Medicine, University of Liverpool, Liverpool, Merseyside, United Kingdom
965. Intensive Care Unit, Royal Infirmary Edinburgh, Edinburgh, UK
966. University of Liverpool, Liverpool, UK

967. University of Glasgow, Glasgow, UK
968. Edinburgh Clinical Research Facility, Western General Hospital, University of Edinburgh, EH4 2XU, UK.
969. NIHR Health Protection Research Unit for Emerging and Zoonotic Infections, Institute of Infection, Veterinary and Ecological Sciences University of Liverpool, Liverpool, L69 7BE, UK.
970. Department of Infectious Diseases, Leiden University Medical Center, Leiden, The Netherlands
971. Cambridge University Hospitals NHS Foundation Trust, Hills Road, Cambridge, CB2 0QQ, UK
972. Roslin Institute, University of Edinburgh, Easter Bush, Edinburgh, EH25 9RG, UK. Intensive Care Unit, Royal Infirmary of Edinburgh, 54 Little France Drive, Edinburgh, EH16 5SA, UK. MRC Human Genetics Unit, Institute of Genetics and Molecular Medicine, University of Edinburgh, Western General Hospital, Crewe Road, Edinburgh, EH4 2XU, UK
973. Roslin Institute, University of Edinburgh, Easter Bush, Edinburgh, EH25 9RG, UK. Department of Infectious Diseases, Leiden University Medical Center, Leiden, The Netherlands
974. Genotek Ltd., Moscow, Russia
975. Helix, San Mateo, CA, USA
976. Center for Genomic Medicine, Desert Research Institute, 2215 Raggio Pkwy, Reno, Nevada 89512, USA
977. Renown Health, 1155 Mill St., Reno, NV 89502, USA
978. 24Genetics, Boston, MA, USA
979. Hospital La Paz Institute for Health Research, Madrid, Spain
980. Division of Pulmonary Medicine, Department of Medicine, Keio University School of Medicine, Tokyo, Japan
981. Department of Statiatical Genetics, Osaka University Graduate School of Medicine, Suita 565-0871, Japan
982. Laboratory of Statistical Immunology, Immunology Frontier Research Center (WPI-IFReC), Osaka University, Suita 565-0871, Japan
983. Integrated Frontier Research for Medical Science Division, Institute for Open and Transdisciplinary Research Initiatives, Osaka University, Suita 565-0871, Japan
984. Division of Health Medical Intelligence, Human Genome Center, the Institute of Medical Science, the University of Tokyo, Tokyo, Japan
985. Laboratory of Viral Infection I, Department of Infection Control and Immunology, Ōmura Satoshi Memorial Institute & Graduate School of Infection Control Sciences, Kitasato University, Tokyo, Japan
986. Department of Surgery, Keio University School of Medicine, Tokyo, Japan.
987. Department of Organoid Medicine, Keio University School of Medicine, Tokyo, Japan.
988. Department of Infectious Diseases, Keio University School of Medicine, Tokyo, Japan.
989. Department of Respiratory Medicine and Clinical Immunology, Osaka University Graduate School of Medicine, Suita 565-0871, Japan
990. Department of Immunopathology, Immunology Frontier Research Center (WPI-IFReC), Osaka University, Suita 565-0871, Japan
991. Institute of Research, Tokyo Medical and Dental University, Tokyo, Japan
992. Department of Insured Medical Care Management, Tokyo Medical and Dental University Hospital of Medicine, Tokyo, Japan
993. Genome Medical Science Project (Toyama), National Center for Global Health and Medicine, Chiba, Japan
994. Division of Gastroenterology and Hepatology, Department of Medicine, Keio University School of Medicine, Tokyo, Japan.
995. M&D Data Science Center, Tokyo Medical and Dental University, Tokyo, Japan
996. Kyoto University, Department of Pathology and Tumor Biology Institute for the Advanced Study of Human Biology (WPI-ASHBi), Kyoto University, Kyoto, Japan

997. Department of Medicine, Center for Hematology and Regenerative Medicine, Karolinska Institute, Stockholm, Sweden
998. Department of Respiratory Medicine and Clinical Immunology, Osaka University Graduate School of Medicine, Suita 565-0871, Japan Department of Statiatical Genetics, Osaka University Graduate School of Medicine, Suita 565-0871, Japan
999. Department of Emergency and Critical Care Medicine, Keio University School of Medicine, Tokyo, Japan
1000. Department of Anesthesiology, Keio University School of Medicine, 35 Shinanomachi, Shinjuku-ku, Tokyo, Japan.
1001. Department of Laboratory Medicine, Keio University School of Medicine, Tokyo, Japan
1002. Division of Infection Control and Prevention, Osaka University Hospital, Suita 565-0871, Japan
1003. Department of Biomedical Ethics and Public Policy, Osaka University Graduate School of Medicine, Suita 565-0871, Japan
1004. Center for Genomic Medicine, Kyoto University Graduate School of Medicine, Kyoto 606-8507, Japan
1005. Department of Pulmonary Medicine, Faculty of Medicine, University of Tsukuba, Tsukuba 305-8575, Japan
1006. Department of Neurosurgery, Faculty of Medicine, the University of Tokyo, Tokyo 113-8655, Japan
1007. Laboratory of Immune Regulation, Department of Microbiology and Immunology, Osaka University Graduate School of Medicine, Suita 565-0871, Japan
1008. Department of Respiratory Medicine and Clinical Immunology, Osaka University Graduate School of Medicine, Suita 565-0871, Japan Laboratory of Immune Regulation, Department of Microbiology and Immunology, Osaka University Graduate School of Medicine, Suita 565-0871, Japan
1009. Medical Innovation Promotion Center, Tokyo Medical and Dental University, Tokyo, Japan
1010. Clinical Research Center, Tokyo Medical and Dental University Hospital of Medicine, Tokyo, Japan
1011. Department of Medical Informatics, Tokyo Medical and Dental University Hospital of Medicine, Tokyo, Japan
1012. Respiratory Medicine, Tokyo Medical and Dental University, Tokyo, Japan
1013. Clinical Laboratory, Tokyo Medical and Dental University Hospital of Medicine, Tokyo, Japan
1014. Kyoto University, Department of Pathology and Tumor Biology, Kyoto, Japan
1015. Department of Respiratory Medicine, Juntendo University Faculty of Medicine and Graduate School of Medicine, Tokyo, Japan
1016. Department of Emergency and Disaster Medicine, Juntendo University Faculty of Medicine and Graduate School of Medicine, Tokyo, Japan
1017. Department of Cardiovascular Biology and Medicine, Juntendo University Faculty of Medicine and Graduate School of Medicine, Tokyo, Japan
1018. Department of Respiratory Medicine, Tokyo Women's Medical University, Tokyo, Japan.
1019. Department of general Medicine, Tokyo Women's Medical University, Tokyo, Japan.
1020. Department of Respiratory Medicine, Saitama Cardiovascular and Respiratory Center, Saitama, Japan
1021. Kawasaki Municipal Ida Hospital, Kanagawa, Japan
1022. JCHO（Japan Community　Health care Organization) Saitama Medical Center, Internal Medicine, Saitama, Japan
1023. Saitama City Hospital, Saitama, Japan
1024. Division of Infection Control, Eiju General Hospital, Tokyo, Japan
1025. Department of Pulmonary Medicine, Eiju General Hospital, Tokyo, Japan
1026. Department of Respiratory Medicine, Osaka Saiseikai Nakatsu Hospital
1027. Department of Respiratory Medicine, Osaka Saiseikai Nakatsu Hospital, Osaka, Japan
1028. Department of Infection Control, Osaka Saiseikai Nakatsu Hospital, Osaka, Japan
1029. Department of Infectious Diseases, Tosei General Hospital, Aichi, Japan

1030. Fukujuji hospital, Kiyose, Japan
1031. Department of Emergency and Critical Care Medicine, Tokyo Women's Medical University Medical Center East, Tokyo, Japan
1032. Department of Medicine, Tokyo Women's Medical University Medical Center East, Tokyo, Japan
1033. Department of Pediatrics, Tokyo Women's Medical University Medical Center East, Tokyo, Japan
1034. Japan Community Health care Organization kanazawa Hospital, Kanazawa, Japan
1035. Division of Pulmonary Medicine, Department of Internal Medicine, Federation of National Public Service Personnel Mutual Aid Associations, Tachikawa Hospital, Tachikawa, Japan
1036. Department of Respiratory Medicine Japan Organization of Occupational Health and Safety KANTO ROSAI HOSPITAL, Kawasaki, Japan
1037. Department of Generai Internal Medicine Japan Organization of Occupational Health and Safety KANTO ROSAI HOSPITAL, Kawasaki, Japan
1038. Departmet of Emergency and Critical Care Medicine Kansai Medical Univrsity General Medical Center, Kirakata, Japan
1039. Department of Respiratory Medicine, Kitasato University Kitasato Institute Hospital, Tokyo, Japan
1040. Ishikawa Prefectural Central Hospital, Kanazawa, Japan
1041. Internal Medicine, Sano Kosei General Hospital, Sano Japan
1042. Saiseikai Yokohamashi Nanbu Hospital, Yokohama, Japan
1043. Kanagawa Cardiovascular and Respiratory Center, Yokohama, Japan
1044. Saiseikai Utsunomiya Hospital,Utsunomiya, Japan
1045. KKR Sapporo Medical Center, Department of Respiratory Medicine, Sapporo, Japan
1046. Internal Medicine, Internal Medicine Center, Showa University Koto Toyosu Hospital, Tokyo, Japan
1047. Department of Respiratory Medicine, Toyohashi Municipal Hospital, Toyohashi, Japan
1048. Keiyu Hospital, Yokohama, Japan
1049. National Hospital Organization Hokkaido Medical Center Department of Rheumatology, Sapporo, Japan
1050. Department of Respiratory Medicine, National Hospital Organization Tokyo Medical Center, Tokyo, Japan
1051. Department of Allergy, National Hospital Organization Tokyo Medical Center, Tokyo, Japan
1052. Department of General Internal Medicine and Infectious Diseases, National Hospital Organization Tokyo Medical Center, Tokyo, Japan
1053. Japanese Red Cross Musashino Hospital, Musashino, Japan
1054. Department of Respiratory Medicine, Tohoku University Graduate School of Medicine, Sendai, Japan
1055. Nihon University School of Medicine, Department of Internal Medicine, Division of Respiratory Medicine, Tokyo, Japan
1056. Department of Emergency and Critical Care Medicine, St.Marianna University School of Medicine, Kawasaki, Japan
1057. Division of General Internal Medicine, Department of Internal Medicine, St. Marianna University School of Medicine, Kawasaki, Japan
1058. National Hospital Organization Kanazawa Medical Center, Kanazawa, Japan
1059. Division of Infectious Diseases and Respiratory Medicine, Department of Internal Medicine, National Defense Medical College
1060. Department of Emergency and Critical Care Medicine, Faculty of Medicine, Fukuoka University, Fukuoka, Japan
1061. Department of Infection Control, Fukuoka University Hospital, Fukuoka, Japan
1062. Tokyo Saiseikai Central Hospital, Tokyo, Japan
1063. Fukuoka Tokushukai Hospital Department of internal medicine, Kasuga, Japan
1064. Department of Infestious Disease and Clinical Research Institute, National Hospital Organization Kyushu Medical Center, Fukuoka, Japan

1065. Department of Respirology, National Hospital Organization Kyushu Medical Center, Division of Respirology, Rheumatology, and Neurology, Department of Internal Medicine Kurume University School of Medicine, Fukuoka, Japan
1066. Department of Infestious Disease, National Hospital Organization Kyushu Medical Center, Fukuoka, Japan
1067. matsumoto city hostpital, Matsumoto, Japan
1068. Uji-TOKUSHUKAI MEDICAL CENTER, Uji, Japan
1069. Department of Respiratory Medicine, Nagoya University Graduate School of Medicine, Nagoya, Japan
1070. Fujisawa city hospital, department of respiratory medicine, Fujisawa,Japan
1071. Sapporo City General Hospital, Sapporo, Japan
1072. Department of Emergency and Critical Care Medicine, Chiba University Graduate School of Medicine, Chiba, Japan
1073. Division of Respiratory Medicine, Social Welfare Organization Saiseikai Imperial Gift Foundation, Inc., Saiseikai Kumamoto Hospital, Kumamoto, Japan
1074. Department of Anesthesiology and Intensive Care Medicine, Kyoto Prefectural University of Medicine, Kyoto, Japan
1075. Ome Municipal General Hospital, Ome, Japan
1076. Hanwa Daini Hospital, Osaka, Japan
1077. Department of Respiratory Internal Medicine, St. Marianna University School of Medicine, Yokohama-City Seibu Hospital, Yokohama, Japan.
1078. Division of Hematology, Department of Internal Medicine, St. Marianna University Yokohama-City Seibu Hospital, Yokohama, Japan.
1079. Division of Pulmonary Medicine, Department of Medicine, Tokai University School of Medicine, Tokai University School of Medicine, Tokyo, Japan
1080. Division of Pulmonary Medicine, Department of medicine, Tokai University School of Medicine, Tokyo, Japan
1081. National Hospital Organization Kumamoto Medical Center, Kumamoto, Japan
1082. Department of Respiratory Medicine, Tokyo Medical University Hospital, Tokyo, Japan
1083. Department of Respiratory Medicine, Japanese Red Cross Medical Center, Tokyo, Japan
1084. JA Toride medical hospital, Toride, Japan
1085. Japan Organization of Occupational Health and Safety Okayama Rosai Hospital, Okayama, Japan
1086. Gifu University School of Medicine Graduate School of Medicine, Emergency and Disaster Medicine, Gifu, Japan
1087. Niigata University, Niigata, Japan
1088. National Hospital Organization Kyoto Medical Center, Kyoto, Japan
1089. Research Institute for Diseases of the Chest, Graduate School of Medical Sciences, Kyushu University, Fukuoka, Japan.
1090. Department of Medicine and Biosystemic Science, Kyushu University Graduate School of Medical Sciences, Fukuoka, Japan.
1091. Department of Emergency and Critical Care Medicine, Tsukuba University, Tsukuba, Japan
1092. Department of Nephrology, Faculty of Medicine, University of Tsukuba, Tsukuba, Japan
1093. Department of Hematology, Faculty of Medicine, University of Tsukuba, Tsukuba, Japan
1094. National Hospital Organization Tokyo Hospital, Tokyo, Japan
1095. Fujioka General Hospital, Fujioka, Japan
1096. Division of Respiratory Medicine and Allergology, Department of Medicine, School of Medicine, Showa University, Tokyo, Japan
1097. Department of Pulmonary Medicine, Fukushima Medical University, Fukushima, Japan
1098. Kansai Electric Power Hospital, Osaka, Japan

1099. Kumamoto City Hospital, Kumamoto, Japan
1100. Department of Emergency and Critical Care Medicine, Tokyo Metropolitan Police Hospital, Tokyo, Japan
1101. Department of Respiratory Medicine, International University of Health and Welfare Shioya Hospital, Narita, Japan
1102. Department of Clinical Laboratory, International University of Health and Welfare Shioya Hospital, Narita, Japan
1103. National Hospital Organization Saitama Hospital, Saitama, Japan
1104. Department of Respiratory Medicine, Gunma University Graduate School of Medicine, Maebashi, Japan
1105. Dept. of Orthopedic Surgery, Tokyo Medical University, Ibaraki Medical Center, Tokyo, Japan
1106. Department of Internal Medicine, Kiryu Kosei General Hospital, Kiryu, Japan
1107. Daini Osaka Police Hospital, Osaka, Japan
1108. Department of Genetics, University Medical Centre Groningen, University of Groningen, Groningen, Netherlands
1109. Department of Epidemiology, University Medical Centre Groningen, University of Groningen, Groningen, Netherlands
1110. Department of Genetics, University Medical Centre Groningen, University of Groningen / Department of Genetics, University Medical Centre Utrecht, P.O. Box 85500, 3508 GA, Utrecht, The Netherlands
1111. Department of Epidemiology, University of Groningen, University Medical Center Groningen, Groningen, The Netherlands
1112. University of Groningen, University Medical Center Groningen, Department of Genetics, Groningen, The Netherlands
1113. UMCG Department of Genetics, UMCG Department of Psychiatry, Groningen, Netherlands
1114. Centre for Heart Lung Innovation, University of British Columbia, Vancouver, BC, Canada
1115. Division of Respiratory Medicine, Faculty of Medicine, University of British Columbia, Vancouver, BC, Canada
1116. Institut universitaire de cardiologie et de pneumologie de Québec – Université Laval, Quebec City, Canada
1117. Department of Genetics and Genomic Sciences, Icahn School of Medicine at Mount Sinai, , New York, NY, USA
1118. University of Washington, Global Health, Seattle, WA, USA
1119. Gossamer Bio 3013 Science Park Road, San Diego, CA, USA
1120. University of Groningen, University Medical Centre Groningen, Department of Pathology and Medical Biology Groningen, The Netherlands
1121. University of Groningen, University Medical Centre Groningen, GRIAC Research Institute, Groningen, The Netherlands
1122. University of Groningen, University Medical Center Groningen, Department of Pulmonary Diseases, Groningen, The Netherlands
1123. Center for Genomic Medicine, Massachusetts General Hospital, Boston, MA, USA
1124. Programs in Metabolism and Medical and Population Genetics, Broad Institute of MIT and Harvard, Cambridge, MA, USA
1125. Diabetes Unit and Center for Genomic Medicine, Massachusetts General Hospital, Boston, MA, USA. Harvard Medical School, Boston, Massachusetts, USA
1126. Channing Division of Network Medicine, Department of Medicine, Brigham and Women's Hospital, Boston, MA, USA
1127. Brigham and Women’s Hospital, Boston, MA, USA
1128. Psychiatric and Neurodevelopmental Genetics Unit, Center for Genomic Medicine, Massachusetts General Hospital, Boston, MA

1129. Department of Neurology, Massachusetts General Hospital, Boston, MA, USA
1130. Division of General Internal Medicine, Massachusetts General Hospital and Dpeartment of Medicine, Harvard Medical School and Program in Medical and Population Genetics, Broad Institute, Boston, MA, USA
1131. Division of Genetics, Department of Medicine, Brigham and Women's Hospital, Broad Institute of MIT and Harvard, Harvard Medical School, Boston, MA, USA
1132. Department of Human Genetics, University of Michigan, Ann Arbor, MI, USA
1133. Mount Sinai Clinical Intelligence Center, Department of Genetics and Genomic Sciences, Icahn School of Medicine at Mount Sinai, New York, NY, 10029, USA
1134. Department of Genetics and Genomic Sciences, Icahn School of Medicine at Mount Sinai, New York, NY, 10029, USA
1135. Sema4, a Mount Sinai venture, Stamford CT, 06902, USA
1136. Seaver Autism Center for Research and Treatment, Department of Psychiatry, Icahn School of Medicine at Mount Sinai, New York, NY 10029, USA
1137. Mount Sinai Clnical Intelligence Center, Charles Bronfman Institute for Personalized Medicine
1138. Department of Genetics & Genomic Sciences, Icahn School of Medicine at Mount Sinai, New York, NY, 10029, USA
1139. Mount Sinai Clinical Intelligence Center, Department of Genetics and Genomic Sciences, Icahn School of Medicine at Mount Sinai, New York, NY 10029, USA
1140. Icahn Institute of Data Science and Genomics Technology, New York, NY 10029, USA
1141. Mount Sinai Clinical Intelligence Center, New York, NY 10029, USA
1142. Department of Genetics and Genomic Sciences, Icahn School of Medicine at Mount Sinai, New York, NY, USA
1143. Charles Bronfman Institute for Personalized Medicine, Icahn School of Medicine at Mount Sinai, New York, NY, 10029, USA
1144. Institute for Genomic Health, Icahn School of Medicine at Mount Sinai, New York, NY, USA
1145. The Charles Bronfman Institute for Personalized Medicine, Icahn School of Medicine at Mount Sinai, New York, NY 10029, USA
1146. The Mindich Child Health and Development Institute, Icahn School of Medicine at Mount Sinai, New York, NY 10029, USA
1147. Pamela Sklar Division of Psychiatric Genomics, Department of Psychiatry, Department of Genetic and Genomic Sciences
1148. Pamela Sklar Division of Psychiatric Genomics, Department of Psychiatry, Department of Genetic and Genomic Sciences, Icahn School of Medicine at Mount Sinai, New York, NY 10029, USA
1149. Seaver Autism Center for Research and Treatment, Department of Psychiatry
1150. Mount Sinai Clinical Intelligence Center, Department of Psychiatry, Department of Genetic and Genomic Sciences, Icahn School of Medicine at Mount Sinai, NY, 10029, USA
1151. Department of Environmental Medicine and Public Health, Icahn School of Medicine at Mount Sinai, New York, NY 10029, USA
1152. Mount Sinai Clinical Intelligence Center
1153. The Hasso Plattner Institute of Digital Health at Mount Sinai
1154. BioMe Phenomics Center, Icahn School of Medicine at Mount Sinai, New York, NY 10029, USA
1155. Department of Medicine, Icahn School of Medicine at Mount Sinai, New York, NY 10029, USA
1156. Pamela Sklar Division of Psychiatric Genomics, Seaver Autism Center for Research and Treatment, Department of Psychiatry, Department of Genetic and Genomic Sciences, Icahn School of Medicine at Mount Sinai, New York, NY 10029, USA
1157. Regeneron Genetics Center, Tarrytown, NY, USA
1158. Phenomic Analytics & Clinical Data Core, Geisinger Health System, Danville, PA, USA
1159. Department of Population Health Sciences, Geisinger Health System, Danville, PA, USA

1160. Department of Molecular and Functional Genomics, Geisinger Health System, Danville, PA, USA
1161. Vrije Universiteit Amsterdam, Amsterdam, UK
1162. Department of Genetics, University of Pennsylvania Perelman School of Medicine, Philadelphia, PA, USA
1163. Department of Biomedical Data Science, Stanford University, Stanford, CA, USA
1164. Institute G. Gaslini, University of Genova, Italy
1165. University of Genova, IRCCS Gaslini, Genova, Italy
1166. Department of Psychiatry, University of North Carolina at Chapel Hill, Chapel Hill, USA
1167. Department of Nutrition, University of North Carolina at Chapel Hill, Chapel Hill, USA
1168. Institute of Neuroscience and Physiology, University of Gothenburg, Gothenburg, Sweden
1169. Department of Medical Sciences, University of Turin, 10126, Turin, Italy
1170. Department of Clinical and Biological Sciences, University of Turin, 10149, Orbassano, Italy
1171. IRCCS Gaslini, Genova, Italy
1172. University of Genova, Genova, Italy
1173. Hopital Mont-Godinne, Yvoir, Belgium
1174. Department of Molecular Medicine, University of Pavia, 27100 Pavia, Italy
1175. Department of Public Health and Pediatric sciences, University of Turin, 10126, Turin, Italy
1176. Qatar Biobank for Medical Research, Qatar Foundation Research, Development and Innovation, Qatar Foundation, Doha, Qatar
1177. Latvian Biomedical Research and Study centre, Ratsupites str. 1k-1, Riga LV-1067, Latvia
1178. Department of Neuroscience, Karolinska Institutet, Stockholm, Sweden
1179. Max Planck Institute for Evolutionary Anthropology, Leipzig, Germany
1180. Anaesthesiology and Intensive Care Medicine, Department of Surgical Sciences, Uppsala University, Uppsala, Sweden
1181. Integrative Physiology, Department of Medical Cell Biology, Uppsala University, Uppsala, Sweden
1182. Hedenstierna Laboratory, CIRRUS, Anaesthesiology and Intensive Care Medicine, Department of Surgical Sciences, Uppsala University, Uppsala, Sweden
1183. Department of Computer Science, School of Engineering, UCLA, Los Angeles, CA, USA
1184. University of California Los Angeles, Los Angeles, CA, USA
1185. Department of Psychiatry and Biobehavioral Sciences, David Geffen School of Medicine at UCLA, Los Angeles, CA, USA
1186. Division of Immunology, Allergy, and Rheumatology, Department of Pediatrics, Department of Microbiology, Immunology, and Molecular Genetics
1187. UCLA, Los Angeles, CA, USA
1188. Departments of Neurology, Psychiatry and Human Genetics, Center for Autism Research and Treatment, Institute for Precision Health, Los Angeles, CA, USA
1189. Departments of Computational Medicine
1190. Pathology and Laboratory Medicine, Human Genetics, David Geffen School of Medicine at UCLA David Geffen School of Medicine at UCLA, Los Angeles, CA, USA
1191. Bioinformatics IDP, UCLA, Los Angeles, CA, USA
1192. Department of Neurology, David Geffen School of Medicine at UCLA, Los Angeles, CA, USA
1193. Departments of Human Genetics and Urology, David Geffen School of Medicine at UCLA, Los Angeles, CA, USA
1194. Genomics England
1195. Queen Mary University, London, United Kingdom
1196. UCL Great Ormond Street Institute of Child Health, London, United Kingdom
1197. University of Cambridge, London, United Kingdom
1198. Department of Human Genetics, McGill University, Montréal, Québec, Canada. Lady Davis Institute, Jewish General Hospital, McGill University, Montréal, Québec, Canada. Kyoto-McGill International

Collaborative School in Genomic Medicine, Graduate School of Medicine, Kyoto University, Kyoto, Japan. Research Fellow, Japan Society for the Promotion of Science
1199. Big Data Institute, Nuffield Department of Population Health, University of Oxford, Li Ka Shing Centre for Health Information and Discovery, Old Road Campus, Oxford, OX3 7LF, United Kingdom
1200. Genomics PLC, King Charles House, Park End Street, Oxford, OX1 1JD, United Kingdom
1201. Nuffield Department of Medicine, Experimental Medicine Division, University of Oxford, John Radcliffe Hospital, Oxford, OX3 9DU, United Kingdom
1202. Public Health England, Field Service, Addenbrooke's Hospital, Cambridge, CB2 0QQ, United Kingdom
1203. Public Health England, Data and Analytical Services, National Infection Service, London, NW9 5EQ, United Kingdom
1204. Program in Bioinformatics and Integrative Genomics, Harvard Medical School, Boston, MA, USA
1205. Program in Biological and Biomedical Sciences, Harvard Medical School, Boston, MA, USA
1206. Wellcome Centre for Human Genetics, University of Oxford, Roosevelt Drive, Oxford, OX3 7BN, United Kingdom
1207. Department of Clinical Research and Leadership, George Washington University, Washington, DC, USA
1208. Department of Human Genetics, The Wellcome Sanger Institute, Wellcome Genome Campus, Hinxton, Cambridge, CB10 1HH, UK
1209. The National Institute for Health Research Blood and Transplant Unit in Donor Health and Genomics, University of Cambridge, Strangeways Research Laboratory, Wort's Causeway, Cambridge, CB1 8RN, UK
1210. Department of Haematology, University of Cambridge, Cambridge Biomedical Campus, Long Road, Cambridge, CB2 0PT, UK
1211. British Heart Foundation Cardiovascular Epidemiology Unit, Department of Public Health and Primary Care, University of Cambridge, Cambridge, UK
1212. British Heart Foundation Centre of Research Excellence, University of Cambridge, Cambridge, UK
1213. National Institute for Health Research Blood and Transplant Research Unit in Donor Health and Genomics, University of Cambridge, Cambridge, UK
1214. Health Data Research UK Cambridge, Wellcome Genome Campus and University of Cambridge, Cambridge, UK
1215. Department of Human Genetics, Wellcome Sanger Institute, Hinxton, UK
1216. Department of Epidemiology, Emory University Rollins School of Public Health
1217. Atlanta CA Health Care System, North Druid Hills, GA, USA
1218. Center for Population Genomics, MAVERIC, VA Boston Healthcare System, Boston, MA, USA
1219. MAVERIC, VA Boston Healthcare System, Boston, MA, USA
1220. Stanford University
1221. Palo Alto VA Healthcare System, Stanford, CA, USA
1222. Department of Biostatistics, Boston Univeristy School of Public Health
1223. Department of Haematology, Central Hospital of Bolzano (SABES-ASDAA), Bolzano, Italy
1224. Laboratory of Clinical Pathology, Hospital of Bressanone (SABES-ASDAA), Bressanone, Italy
